# Navigating the Landscape of Cycloartanyl Cations: Synthesis of Fortunefuroic Acid I, Parkeol, 25,26,27-Trinor-3α-hydroxy-17,13-friedolanosta-8,12-dien-23-one, and Spirochensilide A

**DOI:** 10.1021/jacs.5c22292

**Published:** 2026-03-26

**Authors:** Manuel Kizakis, Marvin Treger, Gerald Dräger, Carolin König, Philipp Heretsch

**Affiliations:** † Institute of Organic Chemistry, 26555 Leibniz Universität Hannover, Schneiderberg 1B, Hannover 30167, Germany; ‡ Institute of Physical Chemistry and Electrochemistry, Leibniz Universität Hannover, Callinstraße 3A, Hannover 30167, Germany

## Abstract

For the semisynthesis of plant-derived triterpenoid natural products, access to cycloartenol or a derivative is mandatory. We here describe a robust access from the renewable feedstock γ-oryzanol and isolation by differentiable reactivity. Using either radical-polar crossover or polar modes of activation, we then selectively access different carbenium ions and elucidate their fate in rearrangements. As a result, a chemical toolbox for the modular and stepwise skeletal rearrangement to access natural products with increasing levels of divergence from the parent cycloartane is reported. Using this toolbox, parkeol-, 17,13-friedocycloartane-, 17,13-friedoparkeol-, as well as 17,13- and 17,14-friedolanostane systems are selectively obtained and transformed via short routes to the plant metabolites parkeol (**3**), fortunefuroic acid I (**4**), 25,26,27-trinor-3α-hydroxy-17,13-friedolanosta-8,12-dien-23-one (“sibiricanone”, **5**), and spirochensilide A (**6**). Probing the innate reactivity of cycloartanyl cations and corroborating the experimental results with DFT calculations sheds light on the potential biogenesis of these and related plant metabolites and, thus, can account for the occurrence of 17,14-friedolanostanes in plants.

## Graphical abstract

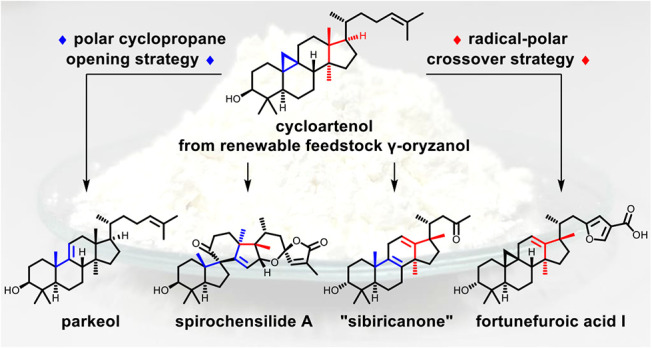

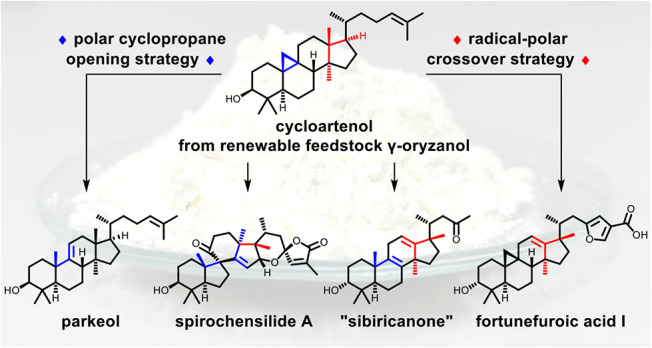

## Introduction

Steroidal natural products have evolved in different kingdoms of life. In animals and fungi, lanosterol is obtained enzymatically from an oxidosqualene precursor and serves as a biosynthetic hub to access cholesterol and its derivatives. In plants, though, mainly cycloartenol (**1**, Scheme 1) is accessed from oxidosqualene, with phytosterols being its biosynthetic derivatives. While many other specific oxidosqualene cyclases exist, in prokaryotes such as *Gemmata obscuriglobus*, mainly parkeol (**3**) is formed.^1^

**Scheme 1:**
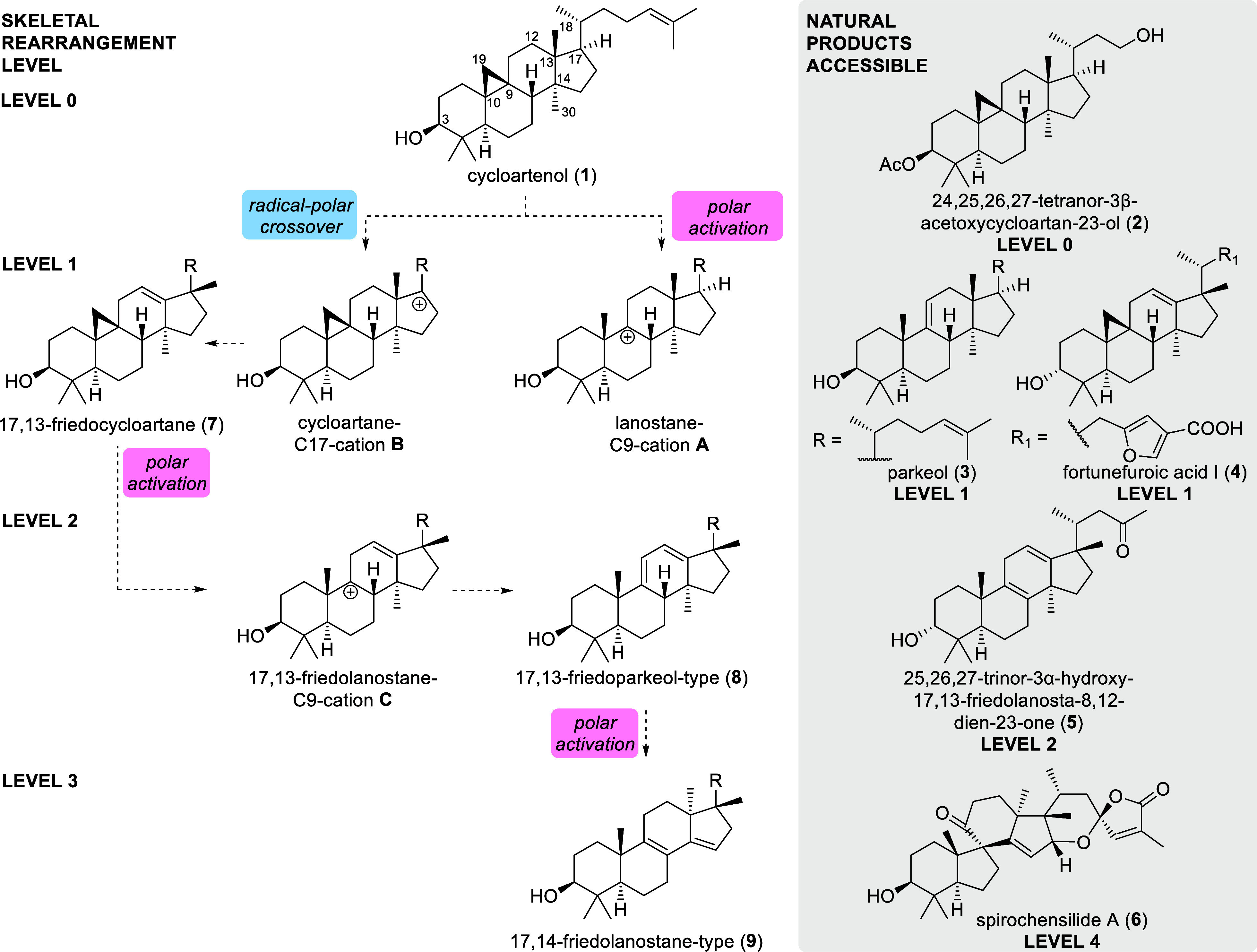
C9 and C17-Centered Carbenium Ions of Cycloartenol (**1**) and Their Potential Rearrangement to Parkeol (**3**), Fortunefuroic Acid I (**4**), or 25,26,27-Trinor-3α-hydroxy-17,13-friedolanosta-8,12-dien-23-one (**5**), as well as a Possible Connection to Spirochensilide A (**6**)

In the past years, our group^2^ as well as others^3^ have spent considerable effort in employing lanosterol in the semisynthesis of complex, rearranged triterpenoid natural products, and, thus, showcased the value of strategies starting from this abundant and renewable feedstock. Employing a radical-polar crossover synthetic strategy, we have recently accessed spirochensilide A (**6**) and B, two rare examples of plant-derived lanostane-type triterpenoids with an ambiguous biogenesis.^2^

We now extended our focus on triterpenoids of the cycloartane-type to access cationic intermediates and elucidate their propensity to undergo methanide and hydride shifts. These conversions would enable and streamline synthetic access to a variety of phytosterols and related triterpenoids. To assess the level of divergence from the parent cycloartane skeleton (which we defined as “level 0”), we deemed each C–C bond manipulation an increase by one level, meaning, e.g., either a methanide shift or a C–C bond scission, e.g., of the cyclopropane ring, would increase the skeletal divergence to “level 1”, and a series of these manipulations would lead to compounds of, e.g., level 3, as later obtained and described in this study.

Thus, C17 carbenium ions as **B** (potentially accessible by radical-polar crossover logic, for more details *vide infra*) could rearrange through a methanide shift of the 18-methyl group to give 17,13-friedosystem **7** (for atom numbering, see structure **1**) which could present an opportunity for the synthesis of fortunefuroic acid I (**4**).^4^ Isolated from the conifer species *Keteleeria fortunei* and possessing dual ATP-citrate lyase and acetyl-CoA carboxylase 1 inhibitory effects in the low micromolar range, **4** constitutes the second ever reported 17,13-friedocycloartane with the only other example being (22*Z*,24*E*)-3α-hydroxy-17,13-friedocycloarta-12,22,24-trien-26-oic acid (not shown), the latter being isolated from the bark of *Guttiferae benthami*.^5^ Despite their unique 17,13-friedostructures, a 3-furoic acid moiety constituting ring E, presents another structural feature of fortunefuroic acid I (**4**), while in (22*Z*,24*E*)-3α-hydroxy-17,13-friedocycloarta-12,22,24-trien-26-oic acid, a (*E*,*Z*)-pentadienoic acid is present, instead.

Chemically as well as biogenetically, and as shown in Scheme 2, we assume a 23-hydroxy-cycloartenol as **10** to undergo alkoxy radical formation to intermediate **D** and, through a 1,5-H atom transfer, to furnish C17-centered radical **E**, which, in turn, undergoes a single electron oxidation (radical-polar crossover) to give carbenium ion **F**. The latter, in a Wagner–Meerwein-type methanide shift, rearranges to 17,13-friedosystem **G**, which, after proton elimination and oxidative furan formation, and through the intermediacy of **11**, eventually yields fortunefuroic acid I (**4**). From a chemical point of view, the mild nature of radical-initiated carbenium ion generation could allow for the cyclopropyl motif to be preserved, while polar, especially Lewis^6^ (*vide infra*) or Brønsted-acidic conditions^7^ may lead to opening of the cyclopropane ring.

**Scheme 2:**
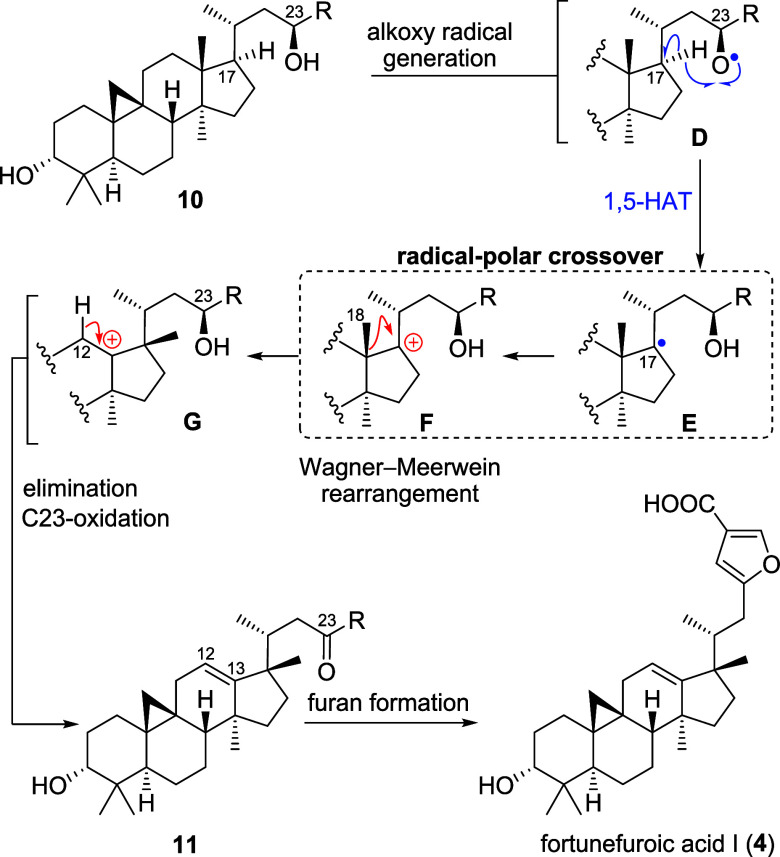
Biosynthetic Proposal for Fortunefuroic Acid I (**4**) Employing Radical-Polar Crossover Logic

On the other hand, activation of the cyclopropane ring itself through scission of the C9–C19 bond forms C9 carbenium ions as **A** or **C** (Scheme 1) and presents a route to access parkeol (**3**) and related skeletons from a cycloartane precursor.

By executing these reaction manifolds sequentially, i.e., a methanide shift and cyclopropane opening, 17,13-friedoparkeol systems as **8** could become available and enable the semisynthesis of a large variety of plant-derived triterpenoids.

Eventually, it remains to be elucidated, if a second methanide shift, i.e., of the C30 methyl group can be initiated from any of the 17,13-friedosystems to allow for an access to 17,14-friedolanostanes as **9**, chemically bridging the gap between the cycloartane and the 17,14-friedolanostane series in plants and deliver a chemically corroborated explanation for the occurrence of the latter, even though lanosterol itself is typically not produced in the plant kingdom.

## Results and Discussion

### Access to Cycloartenol Derivatives

To initiate our studies, we faced the demand for quantities of cycloartenol (**1**) or a close derivative with acceptable purity. Unfortunately, the availability of cycloartenol is severely limited to analytical standards at high price, and no vendor of cycloartenol, even at technical grade purity, could be identified.

Before any further synthetic studies could be initiated, we, thus, had to solve the supply problem, and soon realized, that rather a derivative of cycloartenol could serve as such, with the ferulic ester **12** (Scheme 3A) being especially well-suited due to its abundance in γ-oryzanol, the fat fraction from rice bran oil.^8^ As a renewable feedstock, γ-oryzanol is used, e.g., in organic cosmetics as a sun blocker, and can be purchased in kg-quantities at a cost of around $0.10 per gram. Among other minor constituents, 24-methylenecycloartanyl ferulate (**14**) as well as campesteryl ferulate (**13**) are the main impurities.^9^ It was previously known, that a series of recrystallizations from boiling ethyl acetate suffices to deliver material with a purity above 90%, and indeed, in our hands, this rather tedious procedure of eight consecutive recrystallizations was successful in delivering **12** which could then be saponified (see Scheme 3B) to give **1**.^10^ Given its time- and resource-demanding character, as well as the rather low isolated yield (app. 19% with respect to 40 wt % content of **12** in γ-oryzanol), this access was deemed not suitable for large-scale preparations, though.

**Scheme 3:**
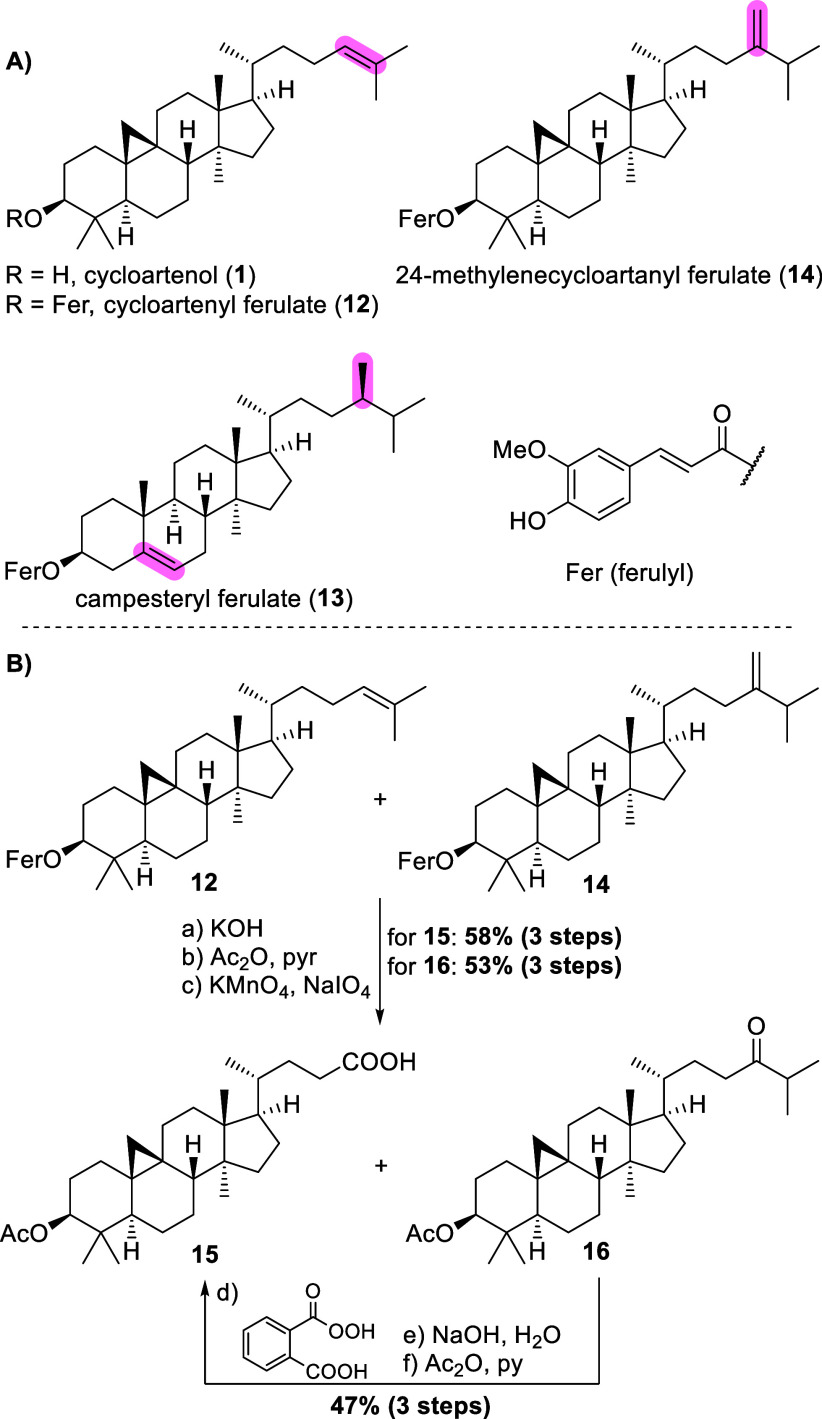
A) Structures of Cycloartenol (**1**), Cycloartenyl Ferulate (12), Campesteryl Ferulate (**13**), and 24-Methylenecycloartanyl Ferulate (**14**); B) Access to Trinor-cycloartanoic Acid **15** from γ-Oryzanol

In order to increase efficiency, we aimed to implement using γ-oryzanol directly as a starting material and to remove impurities through differentiable reactivity in the first steps of our route toward fortunefuroic acid I (**4**) and other natural products.

Saponification followed by acetylation of crude γ-oryzanol gave, among desired cycloartenyl acetate, the acetates of both impurities, 24-methylenecycloartanol and campesterol (not shown) as an inseparable mixture. Oxidative scission of the double bond in cycloartenyl acetate employing Lemieux–Von Rudloff conditions led to carboxylic acid **15**, while campesteryl acetate remained unchanged and 24-methylenecycloartanyl acetate was transformed into the corresponding 24-oxo derivative **16**. Simple extraction and chromatography could then be employed to access pure **15** in 58% yield over the three steps (with respect to a cycloartenyl ferulate content in γ-oryzanol of app. 40%). To further increase the yield of **15**, the 24-oxo derivative **16** (obtained in 53% overall yield from γ-oryzanol with a content of 24-methylenecycloartanyl ferulate of app. 40%) could also be salvaged through highly regioselective Bayer–Villiger oxidation using monoperoxyphthalic acid, followed by global saponification and reacetylation (47% over these 3 steps from pure **16**).

### Synthesis of Fortunefuroic Acid I (Level 1)

With decagram quantities of **15** in hand, we then aimed to implement the radical-polar crossover process to migrate the 18-methyl group. Thus, decarboxylative elimination following Ritter’s protocol^11^ and hydroboration/oxidation of the so-obtained terminal olefin was used to reach the alkoxy radical precursor **2** in 54% yield over the two steps (see Scheme 4). Notably, 24,25,26,27-tetranor-3β-acetoxycycloartan-23-ol (**2**) is a natural product isolated from the wormwood species *Artemisia lagocephala* and represents a “level 0” derivative of cycloartenol according to our system.^12^

**Scheme 4:**
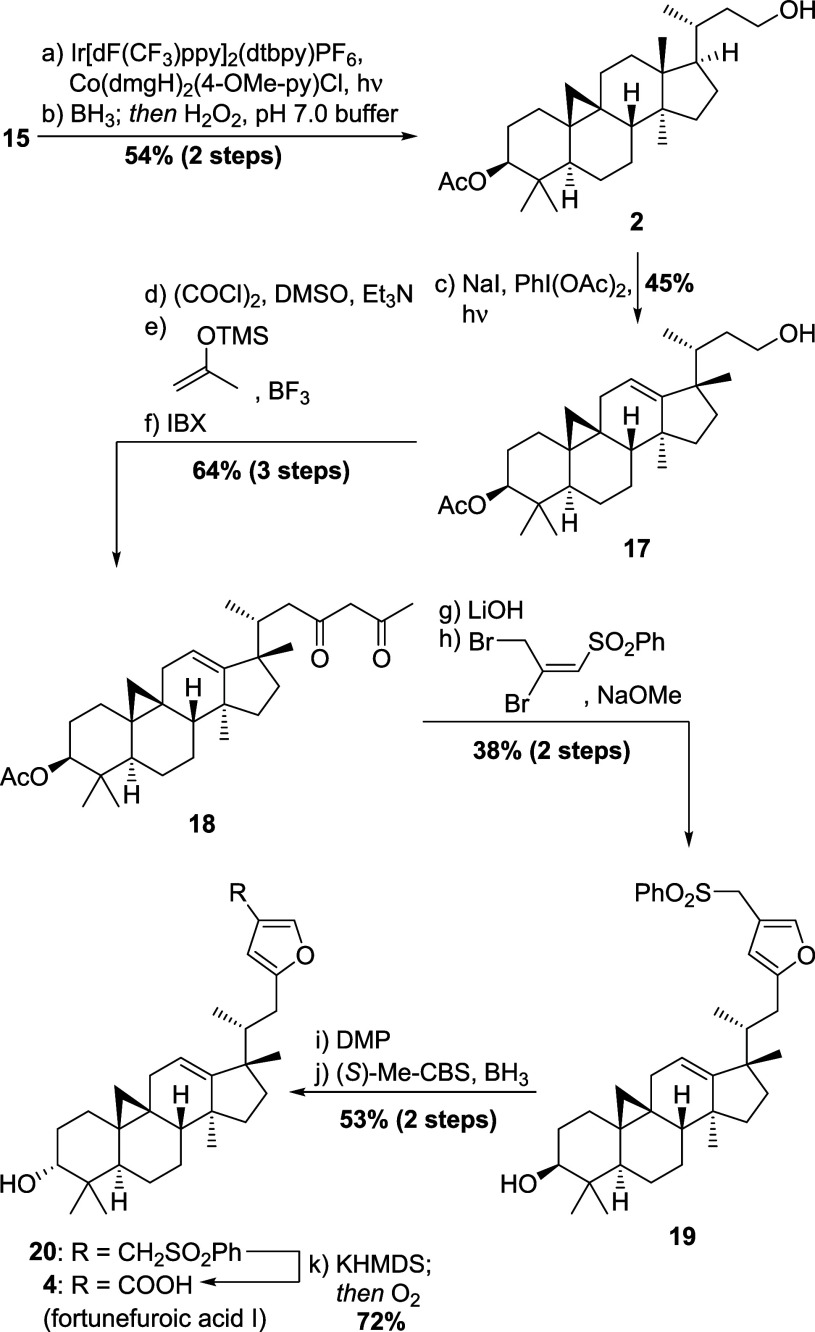
Chemical Synthesis of Fortunefuroic Acid I (**4**) from Precursor **15**

Applying Suárez-type conditions to the latter, a methyl-shifted cycloartane was obtained in 45% isolated yield. 2D NMR analysis could confirm its structure as 17,13-friedocycloartane **17** with a Δ^12^ bond, confirming our rationale as previously outlined (*vide supra*). This methodology was found advantageous and preferable for this purpose for several reasons. It proceeds without the need to install an auxiliary group, as in Deng’s work,^3b^ where a three-step procedure to install a trifluoromethyl ketone as side chain, which is then transformed into a tethered TFDO derivative to allow for C17 oxidation, after which degradation of an intermediary lactol and three more steps for activation of the so-obtained 17-hydroxy group as a leaving group are needed to enable methanide migration. In comparison, our method is traceless (does not require the installation of an auxiliary group or its later removal) and single-stepped (remote activation and methanide shift occur in one flask under the same conditions). Besides these time and resource economic reasons, also the substitution and oxidation state at C23 being either 23-hydroxy or 23-oxo in a wider panel of triterpenoid natural products (or further processed to, e.g., furan-*O* as in fortunefuroic acid I, or dioxadispiro-*O* as in the spirochensilides) provides a hint at the possibility of nature accessing a similar 23-alkoxy radical species for the same purpose of methanide migration, potentially from a 23-hydroperoxy species obtained through a Schenck ene reaction. Our results may, thus, also provide chemical evidence for the greater biosynthetic picture of these and related 17,13- and 17,14-friedotriterpenoid natural products.

Since Wager–Meerwein shifts in triterpenoid biosynthesis are known to be energetically “uphill”,^13^ the termination of the desired methanide shift by an elimination instead of the 30-methyl group to shift into the 13-position and form a 17,14-friedocycloartane is in agreement with this realization and corroborates our earlier^2^ as well as other groups^3b,6^ findings.

From intermediate **17**, what remained to be done was the inversion of stereoconfiguration of the 3β-hydroxy group as well as the installation of the 3-furoic acid moiety in the side chain. Thus, oxidation of the primary alcohol in **17** followed by Mukaiyama aldol reaction employing the silylenol ether of acetone, followed by a second oxidation, delivered 1,3-dicarbonyl compound **18**. Only with 2-iodoxybenzoic acid (IBX) for the second oxidation,^14^ appreciable amounts of diketone could be obtained (64% yield over 3 steps). Removal of the 3-acetate was performed at this stage, since the use of NaOMe in the upcoming furan formation was expected to produce conflicting reactivity. Toward this end, the use of 2,3-dibromo-1-(phenylsulfonyl)-1-propene (DBP) was deemed the most straightforward option to install the E-ring and give **19**,^15^ albeit in a somewhat lower yield of 40% (for optimization, see the Supporting Information). Oxidation of the 3-hydroxy moiety, followed by reduction employing (*S*)-Me-CBS [(*S*)-2-methyl-Corey–Bakshi–Shibata-oxazaborolidine] and borane^16^ then gave rise to the 3α-alcohol **20** (d.r. = 5/1, 3α-/3β-), while other methods either led to no marked diastereocontrol or low conversion (for details, see the Supporting Information). To conclude the first synthesis of fortunefuroic acid I (**4**), the phenylsulfone was converted to the required carboxylic acid through deprotonation and treatment with oxygen.^17^ Thus, in a total of 14 steps and 1.3% overall yield from γ-oryzanol a “level 1” natural product could be obtained. Building on this result, we now aimed at accessing higher level derivatives of cycloartenol, and thus, started exploring the activation the cyclopropane ring.

### Selective Activation of the Cyclopropane Ring and Synthesis of Parkeol (**3**, Level 1)

Following our original plan, we now probed the propensity of cycloartenol- and 17,13-friedocycloartenol skeletons to undergo selective cyclopropane opening and elucidated the fate of the so-obtained C9 carbenium ions.

As shown in Scheme 5, we first subjected pure cycloartenol (**1**) obtained through repeated recrystallization from γ-oryzanol and saponification (*vide supra*) to standard Lewis-acidic treatment employing BF_3_·OEt_2_ and successfully obtained parkeol (**3**) as the main product in 43% yield. Surprisingly, parkeol was previously only obtained as a minor, neither isolated nor purified, byproduct in attempts to mimic triterpenoid biosynthesis,^7c^ through rather tedious multistep processes,^7a,7b^ or as a (by-)­product of metabolic engineering from, e.g., *Oryza sativa* L.^18^ We here have achieved a one-step access to quantities of this evolutionarily important natural product and provide full characterization data in the Supporting Information.

**Scheme 5:**
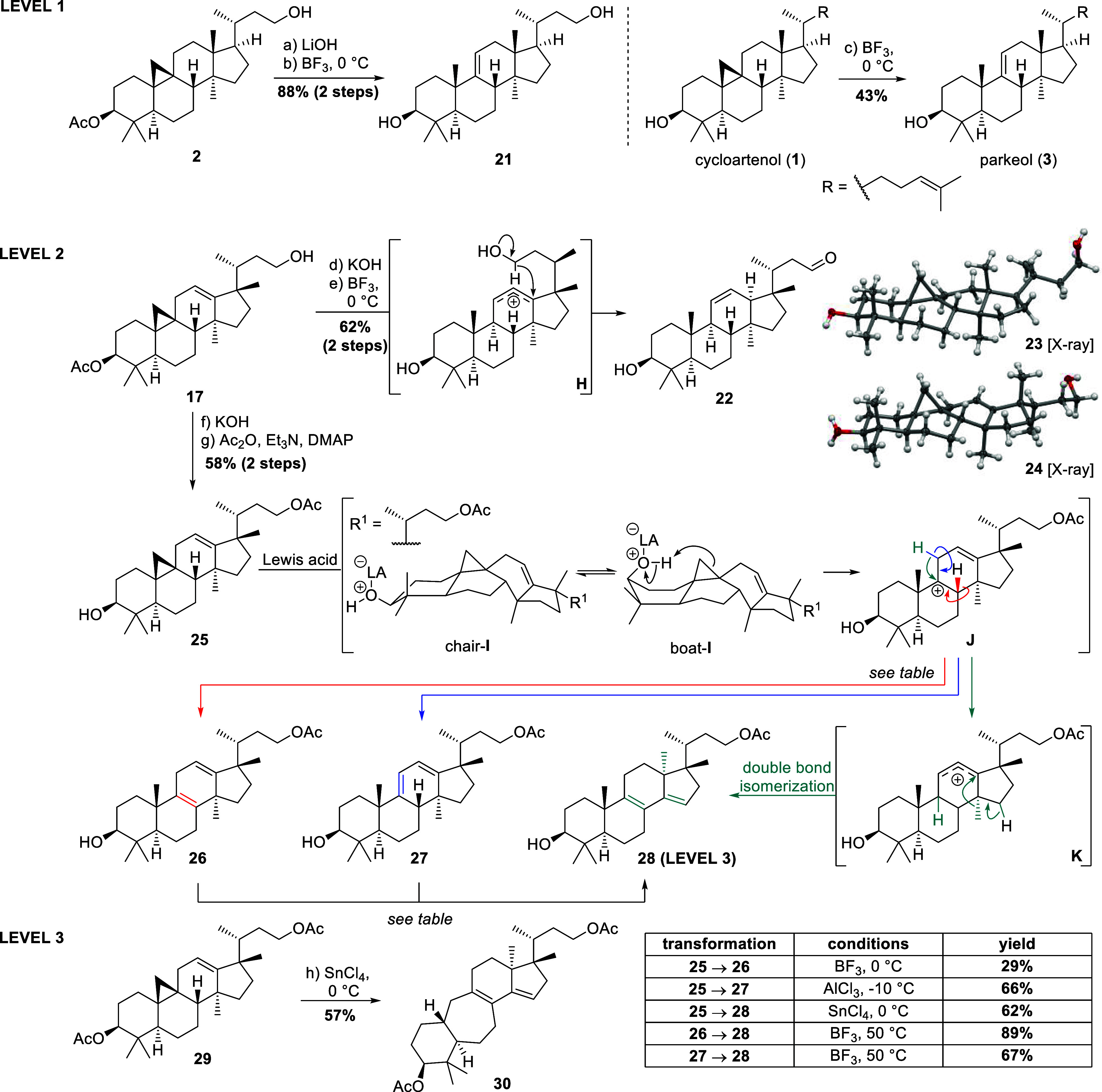
Selective Polar Activation of Cycloartane Derivatives to Access Parkeol Systems, 17,13-, 17,14-Friedolanostanes, and a 9,10-Seco-17,14-friedolanostane, Mechanistic Proposal for Their Formation, and Representation of Single-Crystal X-ray Diffraction Analysis of Cycloartane **23** and 17,13-Friedocycloartene **24** (for Details, see the Supporting Information)

Also, and only when cycloartane-derived **2** was deacetylated, i.e., possessed a free 3-hydroxy group, treatment with BF_3_·OEt_2_ gave related parkeol system **21** in 88% yield.^19^ When employing the acetylated systems instead, complex and inseparable mixtures were obtained with no clear main or even major product to be isolated.

The necessity of a free 3-hydroxy moiety for a selective opening of the cyclopropane ring may be the result of several factors. For once, the coordination of Lewis-acids to alcohols markedly increases their Brønsted-acidity by rendering the oxygen atom electron deficient (see chair-**I**). Thus, protonation of the cyclopropane ring becomes more facile. Second, the 3-hydroxy moiety can direct protonation to the C19 of the cyclopropane ring. This directing effect should be in effect, when the A ring adopts a boat conformation as in boat-**I**. We will elaborate on the thermodynamics of this equilibrium in the next section, where DFT calculations are reported.

We then shifted our attention to 17,13-friedosubstrates to access “level 2” compounds. Thus, subjecting 17,13-friedocycloartane **24** (obtained by saponification of **17**) to the exact same conditions, no parkeol system, but rather aldehyde **22** was obtained in good yield, and presumably as the result of the intermediacy of allylic cation **H**, the latter engaging in a 1,5-hydride shift, facilitated by oxocarbenium stabilization.

Interestingly, while experimentally the cycloartane and the 17,13-friedocycloartene derivatives show markedly different behavior toward treatment with Lewis-acid, the structural differences in the respective single-crystal X-ray diffraction analysis of diol **23** and diol **24** (the latter being the friedosystem) are only subtle (see Scheme 5).

To suppress the formation of an aldehyde as obtained in the previous experiment, the 23-hydroxy moiety in **24** was acetylated to give acetate **25**. When treated with BF_3_·OEt_2_, a separable mixture of products was obtained and structurally elucidated as 1,4-cyclohexadiene **26**, 1,3-cyclohexadiene **27**, and 17,14-friedosystem **28**, the former two being “level 2” derivatives, the latter a “level 3” derivative of cycloartenol.

The occurrence of the former two (1,4- and 1,3-cyclohexadiene products **26** and **27**) can be attributed to the intermediacy of allylic cation **J**, with either the elimination of H-8 (red path in Scheme 5 to **26**) or H-11 (blue path in Scheme 5 to **27**) to give rise to the observed products.

The formation of 17,14-friedosystem **28**, on the other hand, may also be attributed to carbenium ion **J**, where a mechanistic bifurcation is observed following the green path in Scheme 5. Thus, instead of direct proton elimination, H-11 shifts as a hydride into C9, giving rise to allylic carbenium ion **K**. The latter engages in a methanide shift from C13 to C14, while the double bond, under the acidic conditions, undergoes isomerization into the tetrasubstituted, conjugate 8,9-position.

To obtain an experimental estimate about the underlying thermodynamics of the process, both, pure 1,4-cyclohexadiene **26** as well as 1,3-cyclohexadiene **27** were then shown to convert into 17,14-friedosystem **28** under similar Lewis-acidic conditions, employing BF_3_·OEt_2_, but at slightly elevated temperatures (50 °C instead of 0 °C), thus, suggesting either the accessibility of allylic carbenium ion **K** from the former, or a distinct, but similar mode of rearrangement. We will come back to the energies involved in this process in the next section.

To make the whole process synthetically useful, a more selective access to the three entities **26**, **27**, and **28** was needed. The type of Lewis acid and a careful control of the reaction conditions were found crucial toward this goal (for detailed optimization studies, see the Supporting Information). As a result of these efforts, the 1,4-cyclohexadiene **26** was mainly obtained using BF_3_·OEt_2_ and shorter reaction times (29% yield), while the 1,3-cyclohexadiene **27** was the main product (66% yield) of treatment with AlCl_3_. Especially noteworthy is the direct selective formation of 17,14-friedolanostane **28** using SnCl_4_ as a Lewis acid in 62% yield.

To corroborate the importance of the 3-hydroxy group’s directing effect on the selective opening of the cyclopropane between C9 and C19, we ran another control experiment: When subjecting 17,13-friedosystem **29**, with both hydroxy groups acetylated (obtained as a byproduct in the preparation of **25** from **17**), to Lewis-acid treatment, a different ring opening product **30** was isolated and fully characterized (SnCl_4_ provided the highest yield of 57%). In **30**, the cyclopropane ring is cut between C9 and C10 resulting in a B-ring expansion to a seven-membered ring while also a second methanide shift has occurred. The obtained 17,14-friedo-B-*homo*-system is a common motif in natural products as solanoeclepin A^20^ and related hatch-stimulating factors for potato cyst nematodes.

### DFT Calculations

To further substantiate our experiments, we performed DFT (B3LYP/def2-TZVP) and coupled cluster (DLPNO-CCSD­(T)/def2-TZVP) calculations to obtain the free enthalpies of chair-**25**, and boat-**25,** chair-**I**, and boat-**I**, as well as **26**–**28** (for more details, see Figure 1 and the Supporting Information).^21^ The Gibbs reaction energies of the formation of the products **26**, **27**, and **28** were calculated to be between −33.11 and −48.11 kJ mol^–1^. While the smallest reaction Gibbs energy was computed for product **28**, product **27** showed the highest value. The small differences in the reaction Gibbs energies underline the initial observation that a mixture of products was obtained. Furthermore, we calculated the smallest energy for the formation of product **28** which corresponds to the experimental observation that product **26** and **27** convert into product **28**. Finally, the conversions of chair-**25** to boat-**25**, as well as chair-**I** to boat-**I** were examined, revealing a difference in Gibbs energy of about 15 kJ mol^–1^ in the presence of a Lewis acid (compound **I**). In the absence of a Lewis acid (compound **25**), this conversion is associated with a change in Gibbs energy that is about 5 kJ mol^–1^ higher. This demonstrates that the A ring can adopt a boat conformation at ambient temperatures and the presence of a Lewis acid makes this conversion energetically more favorable.

**Figure 1:**
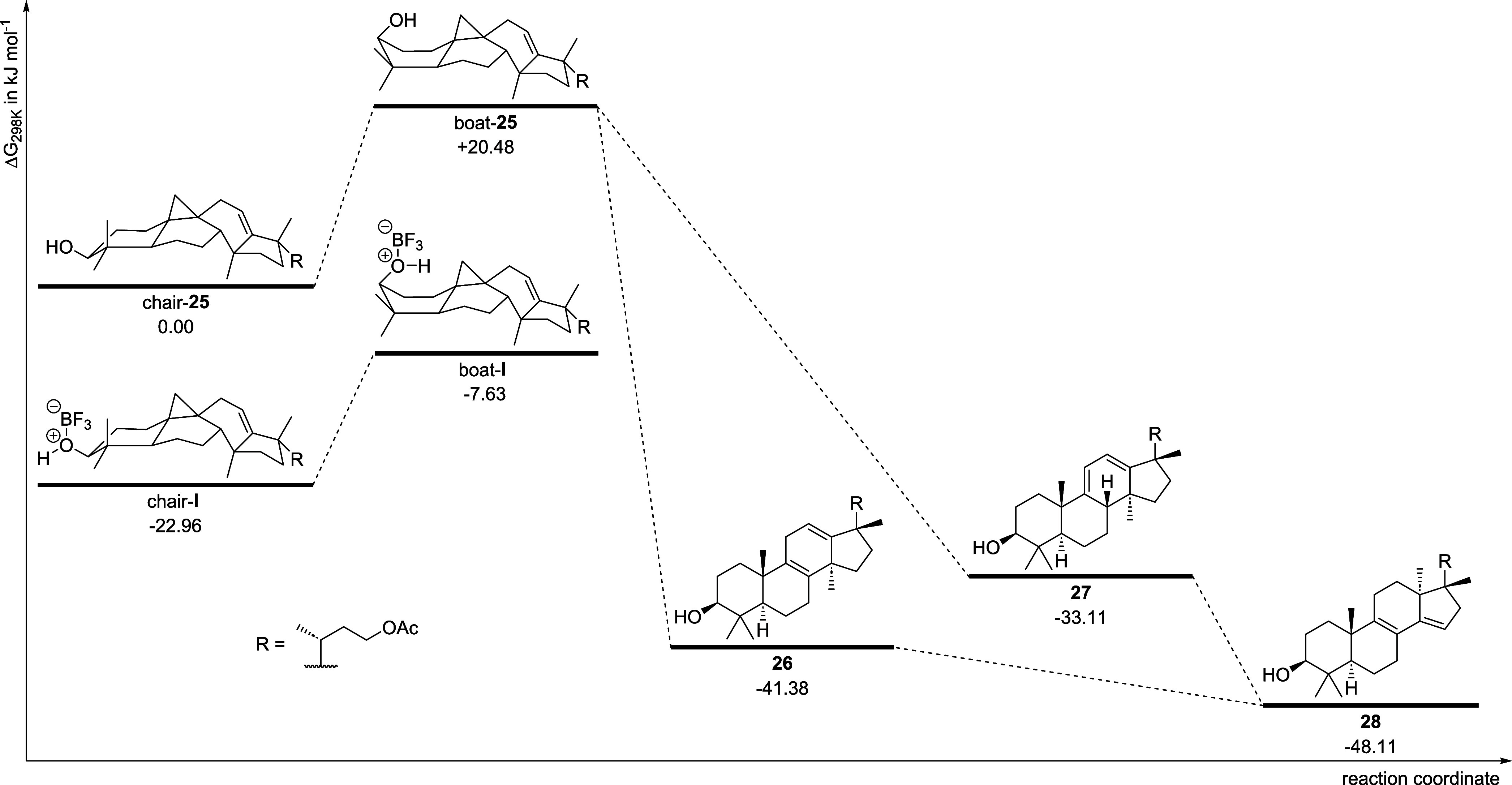
Energy profile of the possible reaction pathway. The differences in Gibbs energy (DLPNO-CCSD­(T)/def2-TZVP) are calculated relative to compound **25** in chair conformation. The differences for the BF_3_ adducts are calculated relative to the sum of the Gibbs energy of **25** in chair conformation and an isolated BF_3_ molecule.

### Synthesis of “Sibiricanone” **5** (Level 2)

Employing rearrangement product **26**, we achieved a short synthesis of yet unnamed 25,26,27-trinor-3α-hydroxy-17,13-friedolanosta-8,12-dien-23-one (“sibiricanone”, **5**), isolated from the needles of *Abies sibirica* in 1996.^22^ Thus, and as shown in Scheme 6, saponification and oxidation under Swern conditions was employed to provide the 3-oxo-aldehyde, which was selectively methylenated under Wittig-conditions to give terminal alkene **31**. Diastereoselective reduction of the remaining 3-oxo-function using (*S*)-Me-CBS and borane,^16^ followed by Wacker oxidation of the terminal olefin to the methyl ketone gave the natural product **5** in 14 steps.

**Scheme 6:**
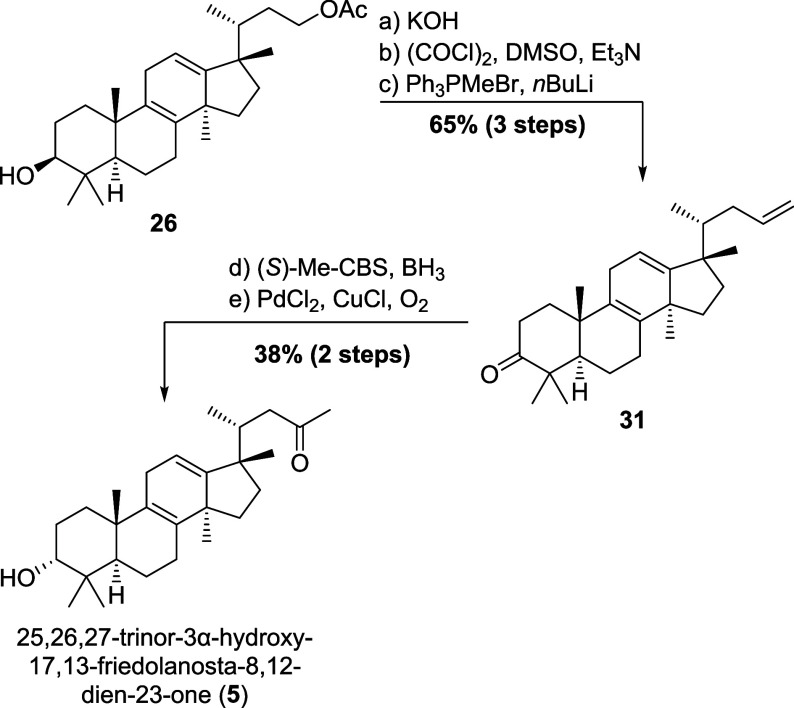
Chemical Synthesis of 25,26,27-Trinor-3α-hydroxy-17,13-friedolanosta-8,12-dien-23-one (“Sibiricanone”, **5**) from Advanced Precursor **26**

### Formal Synthesis of Spirochensilide A (Level 3 and Beyond)

To conclude this study and access a natural product from a level 3 intermediate, a transposition of the acetyl group to the 3-position in **28** (see Scheme 7) yielded 17,14-friedoalcohol **32**, a reported intermediate in our previously published synthesis of the spirochensilides.^2^ Treatment of this level 3 intermediate with *N*-iodosuccinimide and AgNO_3_ in a mixture of hexafluoroisopropanol and water initiated a Meinwald rearrangement to forged all-carbon spirocycle **33** and formally elevated the divergence to level 4. From there, another seven steps (10% total yield) were required to arrive at spirochensilide A (**6**). The above findings, thus, constitute a formal synthesis of spirochensilide A (**6**) from cycloartenol (**1**) instead of previously employed lanosterol. Importantly, the particular rearrangement as well as the propensity of related compounds as **26** and **27** to undergo similar rearrangements to the same 17,14-lanostane skeleton offers new insight into the potential biosynthesis of these fir metabolites from a cycloartane instead of a lanostane precursor, the latter being a very rare intermediate in plant metabolism.^23^ Indeed, the selective opening of the cyclopropane ring between C9 and C19 in cycloartenol and its derivates, thus, constitutes a general access into the lanostane series and can explain the occurrence of such metabolites in plants.

**Scheme 7:**
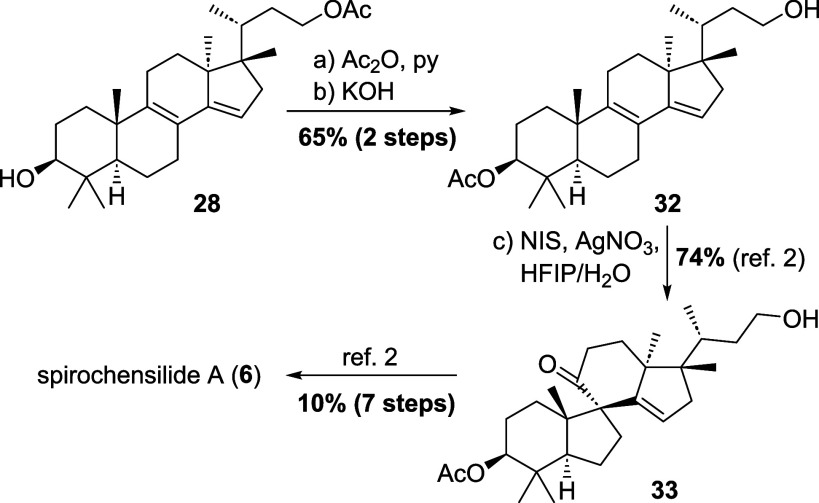
Formal Synthesis of Spirochensilide A (**6**) from Cycloartenol-derived Precursor **28**

## Conclusion

We have demonstrated the value of combining radical-polar as well as polar activation modes for the modular, selective rearrangement of the cycloartane framework to gain access to triterpenoid natural products of increasing divergence from the parent compound. Therefore, we have devised conditions to isolate cycloartane derivatives from the renewable feedstock γ-oryzanol and transform them selectively into parkeol-, 17,13-friedocycloartane-, 17,13-friedoparkeol-, as well as 17,14-friedolanostane systems and employ these in the synthesis of the plant metabolites parkeol (**3**), fortunefuroic acid I (**4**), and 25,26,27-trinor-3α-hydroxy-17,13-friedolanosta-8,12-dien-23-one (“sibiricanone”, **5**). Furthermore, the possibility to cross from the 17,13-friedocycloartane into the 17,14-friedolanostane series presents a chemically corroborated biosynthetic possibility to explain the occurrence of rearranged lanostanes such as spirochensilide A (**6**) in plants and concludes a formal synthesis from cycloartenol (**1**) instead of previously reported lanosterol. Employing DFT calculations, further insights into the structural requirements for these rearrangements as well as innate reactivity of the species obtained could be gathered. Future work will access other carbenium ion intermediates to further navigate the cation landscape of plant-derived triterpenoids and broaden our chemical and theoretical understanding of the underlying biogenesis.

## Supplementary Material



## Notes

General methods; detailed experimental studies; experimental procedures and spectral data; comparison of synthetic and natural fortunefuroic acid I (**4**), parkeol (**3**), 25,26,27-trinor-3α-hydroxy-17,13-friedolanosta-8,12-dien-23-one (**5**), and 4,4,14α-trimethyl-23-hydroxy-9β,19-cyclo-24-nor-5α-cholan-3β-yl acetate (**2**); ^1^H and ^13^C NMR spectra; and references (PDF)
