# Supplementary material for: Navigating the Landscape of Cycloartanyl Cations: Synthesis of Fortunefuroic Acid I, Parkeol, 25,26,27-Trinor-3α-hydroxy-17,13-friedolanosta-8,12-dien-23-one, and Spirochensilide A
Source: J Am Chem Soc. 2026 Mar 26;148(13):13912–20. doi: 10.1021/jacs.5c22292 (PMC13067352; doi:10.1021/jacs.5c22292)
Supplement: Supplementary file 1 [file ja5c22292_si_001.pdf]

Navigating the Landscape of Cycloartanyl Cations: Synthesis of Fortunefuroic Acid I, Parkeol, 25,26,27-Trinor-3α-hydroxy-17,13-friedolanosta-8,12-dien-23-one, and Spirochensilide A

Manuel Kizakis[a], Marvin Treger[b], Gerald Dräger[a],
Carolin König[b], Philipp Heretsch[a,*]

[a] Institute of Organic Chemistry, Leibniz Universität Hannover, Schneider-berg 1B, 30167 Hannover, Germany
[b] Institute of Physical Chemistry and Electrochemistry, Leibniz Universität Hannover, Callinstraße 3A, 30167 Hannover, Germany

*Correspondence to: philipp.heretsch@oci.uni-hannover.de

Supporting Information

Table of Contents

S1 Synthetic Route to Fortunefuroic Acid I (**4**) ... 3

S2 Synthetic Route to 25,26,27-Trinor-3α-hydroxy-17,13-friedolanostan-8,12-dien-23-one (**5**) ... 4

S3 Lewis acid induced cyclopropane opening reactions, synthesis of Parkeol (**3**) and formal synthesis of Spirochensilide A (**6**). ... 5

S4 General methods ... 6

S5 Experimental Procedures and Characterization Data ... 8

S6 X-ray crystallographic data ... 57

S7 Optimization of the furan formation to **19** ... 60

S8 Optimization of the reduction to α-alcohol in position 3 ... 61

S9 Cyclopropane Opening Experiments on Acetate **25**. ... 62

S10 DFT calculations ... 65

S11 NMR Comparisons ... 67

S12 Recrystallizations of γ-Oryzanol ... 75

S13 References ... 78

S14 NMR-spectra ... 80

S1 Synthetic Route to Fortunefuroic Acid I (4).

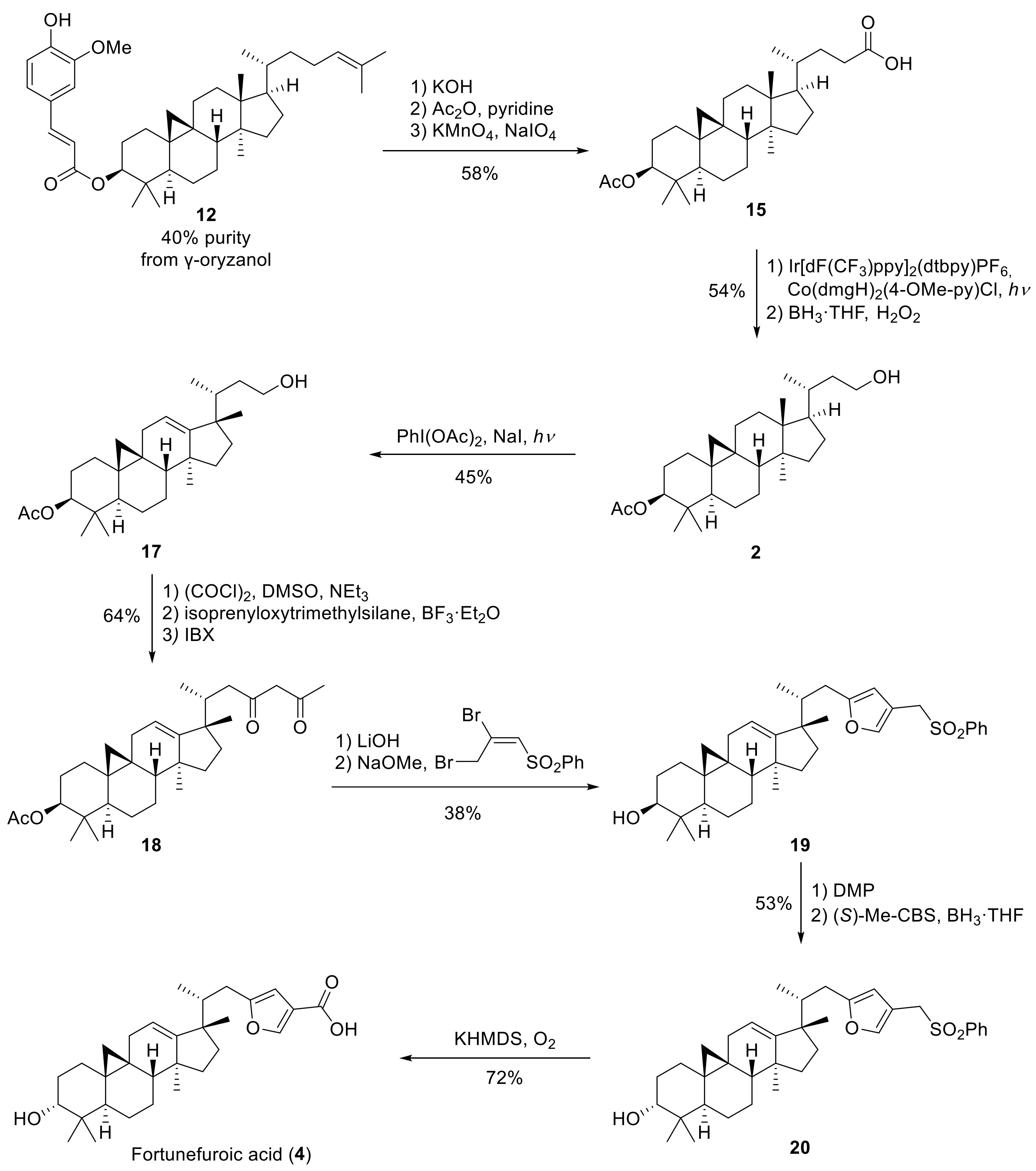

S2 Synthetic Route to 25,26,27-Trinor-3α-hydroxy-17,13-friedolanostan-8,12-dien-23-one (5).

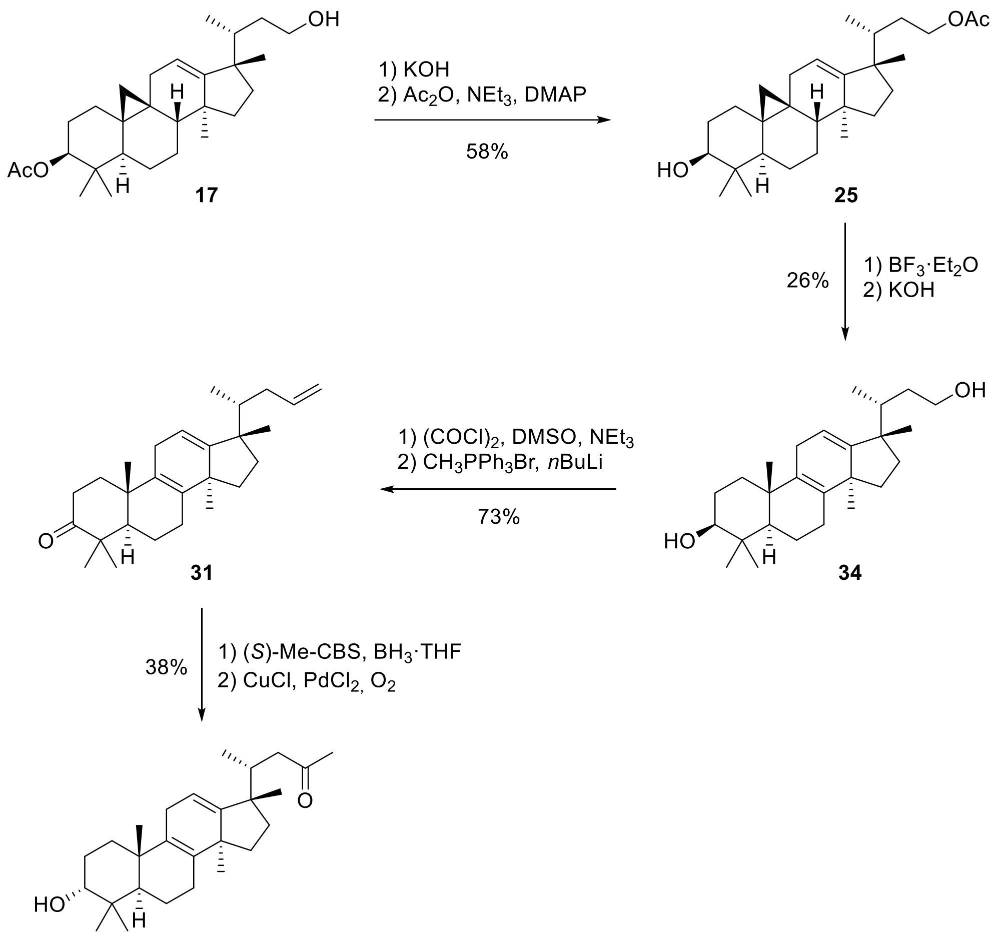

25,26,27-Trinor-3α-hydroxy-17,13-friedolanostan-8,12-dien-23-one, "Sibiricanone" (**5**)

S3 Lewis acid induced cyclopropane opening reactions, synthesis of Parkeol (3) and formal synthesis of Spirochensilide A (6).

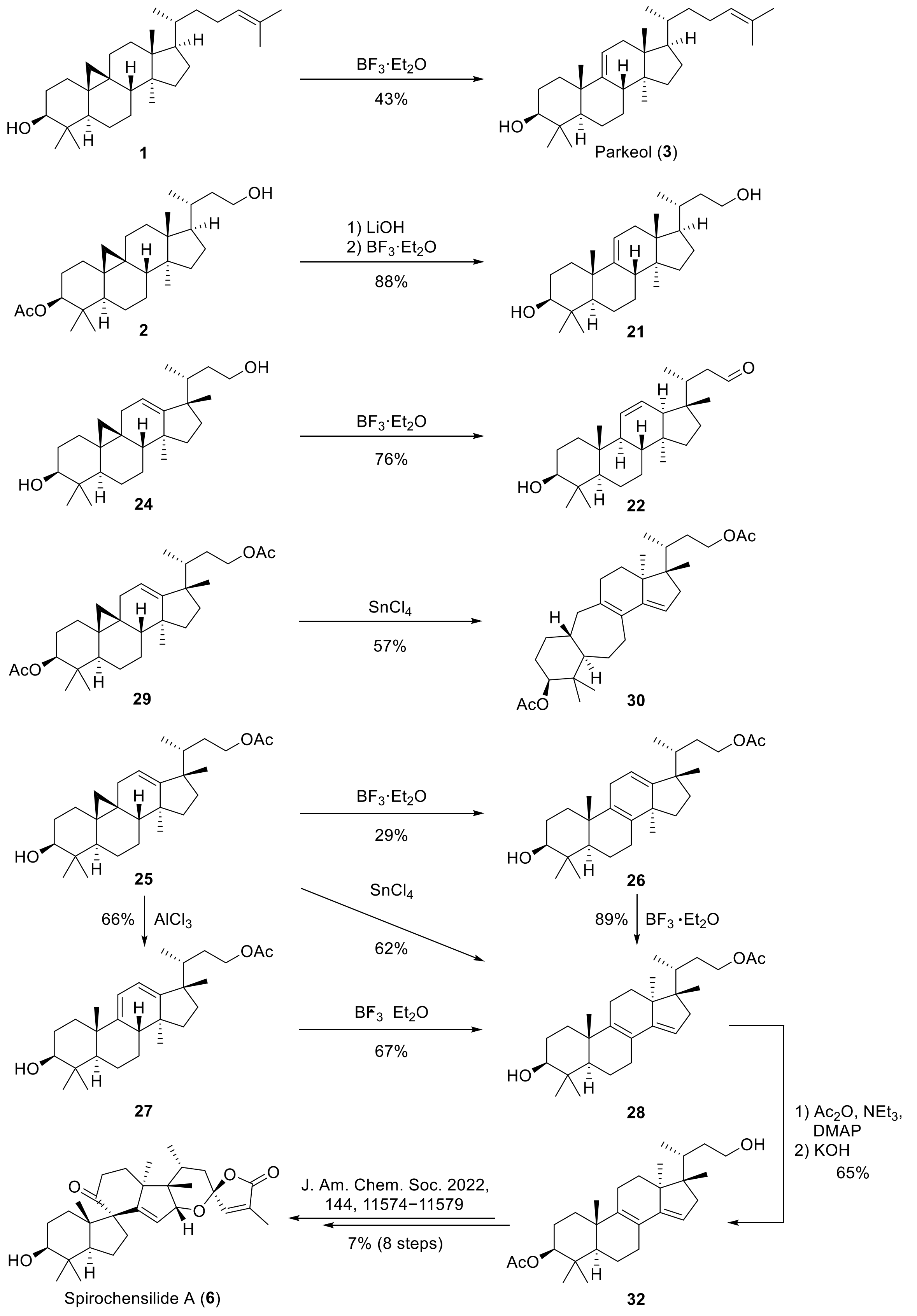

S4 General methods.

All reactions sensitive to moisture and/or air were carried out using heat gun-dried glassware, under an atmosphere of argon, and anhydrous solvents. Anhydrous dichloromethane, toluene, tetrahydrofuran, and diethyl ether were taken from a M. Braun GmbH MB SPS-800 solvent purification system and were stored over 4 Å molecular sieves. Petroleum ether and *n*hexane were purified by distillation. NaI was dried overnight under high vacuum (10^{-3} mbar) at 90 °C and stored under argon. γ-Oryzanol was acquired from Dragonspice Naturwaren. All other solvents (HPLC quality) and commercially available reagents were used without further purification unless otherwise stated.

Concentration under reduced pressure was performed by rotary evaporation at 45 °C and appropriate pressure, followed by exposure to high vacuum (10^{-3} mbar) at 25 °C.

Melting Point: Melting points were measured on a Büchi B-545.

NMR: NMR spectra were recorded on either a Jeol ECX400 (400 MHz), a Bruker Ultrashield 400 (400 MHz), a Jeol ECP500 (500 MHz), a Bruker AVANCE III 500 (500 MHz) or a Bruker Ascend 600 (600 MHz, with CryoProbe) spectrometer. Chemical shifts δ are reported in parts per million (ppm) using residual undeuterated solvent ($CDCl_3$: δ_H = 7.26 ppm, δ_C = 77.16 ppm unless otherwise stated) as an internal reference at 298 K. The given multiplicities are phenomenological; thus, the actual appearance of the signals is stated and not the theoretically expected one. The following abbreviations are used to designate multiplicities: s = singlet, d = doublet, t = triplet, q = quartet, p = pentet, br = broad, and combinations thereof. In case no multiplicity could be identified, the chemical shift range of the signal is given (m = multiplet).

IR spectroscopy: Infrared (IR) spectra were measured on a Jasco FT/IR-4100 Type A spectrometer with a TGS detector or a SHIMADZU FT-IR Affinity-1S spectrometer. Wavenumbers $\tilde{\nu}$ are given in cm^{-1} and intensities are as follows: s = strong, m = medium, w = weak, b = broad.

Mass spectrometry: High-resolution mass spectra (HRMS) were recorded using an Agilent 6210 ESI-TOF or a Waters QTof Premier (with an Acquity UPLC system) spectrometer.

Optical rotation: Optical rotations were measured on a JASCO P-2000 polarimeter at 589 nm using 100 mm cells and the solvent and concentration (g/100 mL) indicated.

Chromatography: Reactions were monitored by thin-layer chromatography (TLC) carried out on Merck Silica Gel 60 F_{254}-plates and visualized by fluorescence quenching under UV-light or staining with an aqueous solution of cerium sulphate and phosphomolybdic acid and heat. Column chromatographic purifications were performed with Macherey-Nagel Silica Gel 60 M (40–60 µm). Silica impregnated with $AgNO_3$ was prepared according to [1]. Preparative TLC was performed on 20x20 cm Merck Silica Gel 60 F_{254}-glass plates.

S5 Experimental Procedures and Characterization Data.

Isolation of cycloartenyl ferulate (**12**)[2]

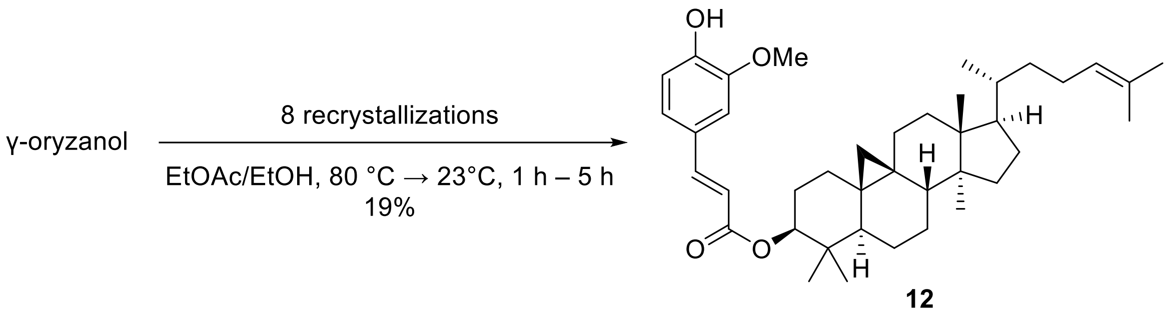

To obtain cycloartenyl ferulate **12** from γ-oryzanol (containing approx. 40% by weight), a total of eight recrystallizations were carried out, in each the solid was dissolved at boiling temperature (80 °C app., for 10 min), and then allowed to crystallize by cooling slowly to 23 °C for the time indicated below, after which filtration through fritted glas was carried out. The crystals so-obtained were further fried under reduced pressure.

1st recrystallization: γ-Oryzanol (50.0 g) was dissolved in EtOAc:EtOH (3:1, 70 mL) and allowed to crystallize over 1 h (30.1 g, CAF:mCAF = 100:81).

2nd crystallization: The colorless solid was dissolved in EtOAc:EtOH (3:1, 40 mL) and allowed to crystallize over 1 h (23.7 g, CAF:mCAF = 100:71).

3rd recrystallization: The colorless crystals were dissolved in EtOAc (40 mL) and allowed to crystallize over 5 h (14.3 g, CAF:mCAF = 100:62).

4th recrystallization: The colorless solid was dissolved in EtOAc (30 mL) and allowed to crystallize over 5 h (11.2 g, CAF:mCAF = 100:30).

5th recrystallization: The colorless crystals were dissolved in EtOAc (25 mL) and allowed to crystallize over 5 h (8.68 g, CAF:mCAF = 100:20).

6th recrystallization: The colorless crystals were dissolved in EtOAc (23 mL) and allowed to crystallize over 5 h (7.26 g, CAF:mCAF = 100:14).

7th recrystallization: The colorless crystals were dissolved in EtOAc (20 mL) and allowed to crystallize over 5 h (5.89 g, CAF:mCAF = 100:10).

8th recrystallization: The colorless crystals were dissolved in EtOAc (18 mL) and allowed to crystallize over 5 h to obtain cycloartenyl ferulate **12** as colorless crystals (4.06 g, 6.73 mmol, 19%, CAF:mCAF = 100:6).

TLC: R_f = 0.47 (*n*hexane/EtOAc = 7:3, *v*/*v*).

^{1}H-NMR: (400 MHz, $CDCl_3$) δ [ppm] = 7.59 (d, *J* = 8.1 Hz, 1H), 7.09–7.04 (m, 2H), 6.91 (d, *J* = 8.1 Hz, 1H), 6.30 (d, *J* = 15.9 Hz, 1H), 5.84 (s, 1H), 5.10 (tp, *J* = 7.1 Hz, 1.4 Hz, 1H), 4.74–4.69 (m, 1H), 3.94 (s, 3H), 2.07–1.97 (m, 2H), 1.93–1.80 (m, 3H), 1.69–1.65 (m, 5H), 1.63–1.51 (m, 9H), 1.47–1.26 (m, 8H), 1.17–1.01 (m, 3H), 0.97 (s, 3H), 0,97 (s, 3H), 0.91 (s, 3H), 0.90 (s, 3H), 0.88 (s, 3H), 0.60 (d, *J* = 4.2 Hz, 1H), 0.37 (d, *J* = 4.2 Hz, 1H).

^{13}C-NMR: (101 MHz, $CDCl_3$) δ [ppm] = 167.2, 147.9, 146.9, 144.4, 131.1, 127.3, 125.4, 123.2, 116.5, 114.8, 109.4, 80.7, 56.1, 52.4, 49.0, 48.0, 47.4, 45.4, 39.9, 36.5, 36.0, 35.7, 33.0, 31.8, 29.9, 28.3, 27.1, 26.7, 26.1, 26.0, 25.9, 25.6, 25.1, 21.1, 20.3, 19.4, 18.4, 18.1, 17.8, 15.5.

HRMS: (ESI-TOF); m/z calculated for $C_{40}H_{57}O_4^-$ $[M-H^+]^-$: 601.4262, found: 601.4261.

The spectroscopic data are in agreement with those reported in [2].

Synthesis of cycloartenol (**1**)[2]

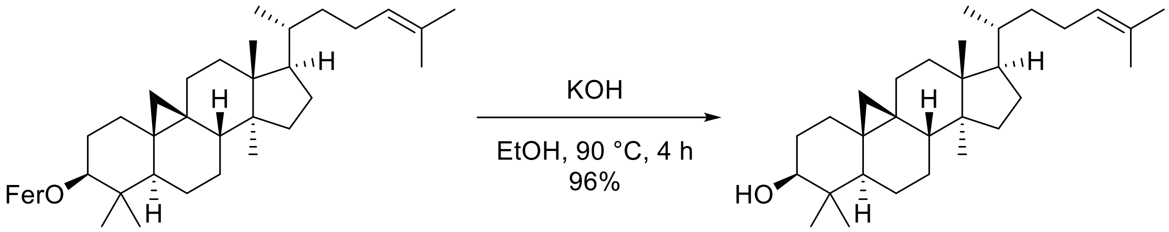

12 **1**

To a solution of cycloartenyl ferulate **12** (1.41 g, 2.35 mmol, 1.0 eq.) in EtOH (40 mL) was added KOH (10 M, aq., 4.42 mL) at 23 °C. After stirring at 90 °C for 4 h the solvent was removed under reduced pressure and the resulting solid was dissolved in CH_2Cl_2 (40 mL). The organic phase was successively washed with sat. aq. $NaHCO_3$ (20 mL) and brine (20 mL), dried over Na_2SO_4, filtered, and the solvent was removed under reduced pressure. The crude product was purified by column chromatography (SiO_2, *n*hexane/EtOAc = 4:1, *v*/*v*) to obtain cycloartenol (**1**) as a colorless solid (960 mg, 2.25 mmol, 96%).

TLC: R_f = 0.63 (*n*hexane/EtOAc = 4:1, *v*/*v*).

^{1}H-NMR: (400 MHz, $CDCl_3$) δ [ppm] = 5.10 (t, *J* = 6.9 Hz, 1H), 3.28 (dd, *J* = 11.2, 4.3 Hz, 1H), 1.08–1.95 (m, 2H), 1.93–1.81 (m, 2H), 1.75 (m, 1H), 1.68 (s, 3H), 1.63–1.54 (m, 8H), 1.53–1.33 (m, 5H), 1.32–1.22 (m, 6H), 1.15–1.01 (m, 3H), 0.96 (s, 3H), 0.96 (s, 3H), 0.89 (s, 3H), 0.87 (s, 3H), 0.81 (s, 3H), 0.79–0.73 (m, 1H), 0.55 (d, *J* = 4.2 Hz, 1H), 0.33 (d, *J* = 4.2 Hz, 1H).

^{13}C-NMR: (101 MHz, $CDCl_3$) δ [ppm] = 131.0, 125.4, 79.0, 52.4, 48.9, 48.1, 47.3, 45.4, 40.6, 36.5, 36.0, 35.7, 33.0, 32.1, 30.5, 30.0, 28.3, 26.6, 26.2, 26.2, 25.9, 25.6, 25.1, 21.3, 20.1, 19.5, 18.4, 18.2, 17.8, 14.1.

The spectroscopic data are in agreement with those reported in [2].

Synthesis of 3-*O*-acetylcycloartenol (**35**)[2]

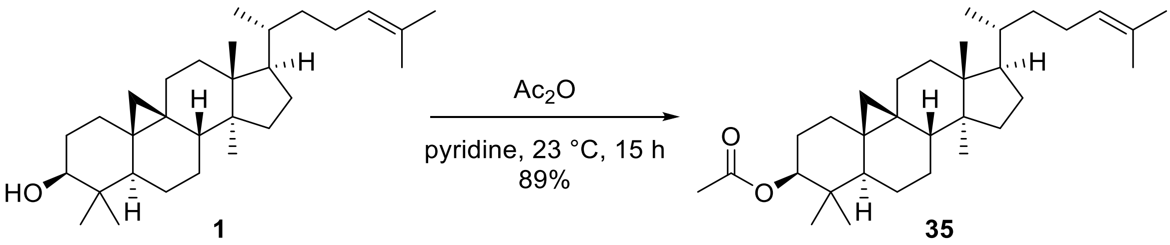

Cycloartenol (**1**) (951 mg, 2.23 mmol, 1.0 eq.) was dissolved in pyridine (10 mL) and acetic anhydride (10 mL) was added at which point the mixture was stirred for 15 h at 23 °C. The solvent was then removed under reduced pressure and the resulting colourless solid was redissolved in CH_2Cl_2 (60 mL). The organic phase was washed with brine (20 mL), dried over Na_2SO_4, filtered, and the solvent was removed under reduced pressure. The crude product was purified by column chromatography (SiO_2, *n*hexane/EtOAc = 9:1, *v*/*v*) to obtain acetate **35** as a colorless solid (934 mg, 1.99 mmol, 89%).

TLC: R_f = 0.69 (*n*hexane/EtOAc = 9:1, *v*/*v*).

^{1}H-NMR: (400 MHz, $CDCl_3$) δ [ppm] = 5.13–5.07 (m, 1H), 4.57 (dd, *J* = 11.3, 4.4 Hz, 1H), 2.05 (s, 3H), 2.05–2.01 (m, 1H), 1.99–1.82 (m, 3H), 1.79–1.72 (m, 1H), 1.68 (s, 3H), 1.65–1.51 (m, 10H), 1.49–1.36 (m, 3H), 1.34–1.23 (m, 5H), 1.16–1.00 (m, 3H), 0.96 (s, 3H), 0.89 (s, 3H), 0.89 (s, 3H), 0.87 (s, 3H), 0.85 (s, 3H), 0.79 (dd, *J* = 12.6, 2.6 Hz, 1H), 0.57 (d, *J* = 4.3 Hz, 1H), 0.34 (d, *J* = 4.3 Hz, 1H).

^{13}C-NMR: (101 MHz, $CDCl_3$) δ [ppm] = 171.1, 131.1, 125.4, 80.8, 52.4, 48.9, 48.0, 47.3, 45.4, 39.6, 36.5, 36.0, 35.7, 33.0, 31.7, 29.9, 28.3, 26.9, 26.6, 26.1, 26.0, 25.9, 25.6, 25.1, 21.5, 21.1, 20.3, 19.4, 18.4, 18.1, 17.8, 15.3.

The spectroscopic data are in agreement with those reported in [2].

Synthesis of 4,4,14α-trimethyl-3β-acetoxy-9β,19-cyclo-5α-cholan-24-oic acid (**15**)

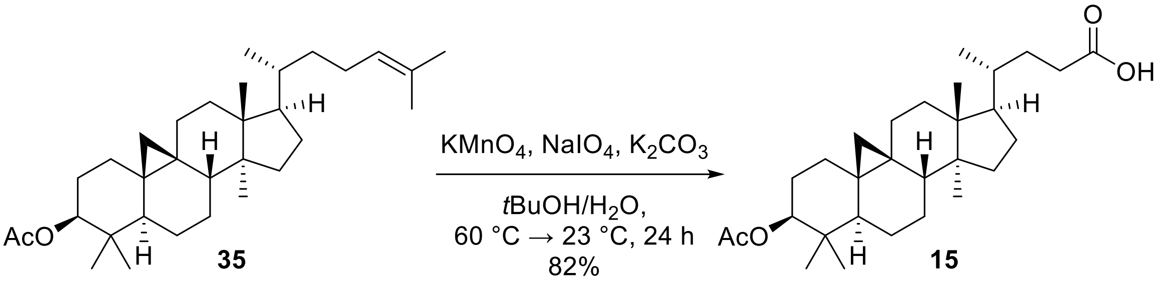

3-OAc-Cycloartenol **35** (930 mg, 1.98 mmol, 1.0 eq.) was dissolved in *t*BuOH (42 mL), K_2CO_3 (823 mg, 5.95 mmol, 3.0 eq.) was added and the solution was heated to 60 °C. At this temperature, a suspension of $KMnO_4$ (125 mg, 794 µmol, 0.4 eq.) and $NaIO_4$ (2.04 g, 9.52 mmol, 4.8 eq.) in water (25 mL) was added dropwise, and the reaction solution was stirred for 2 h at 60 °C and then for 22 h at 23 °C. After this time, the reaction was quenched with aq. $NaHSO_3$ (10wt%, 50 mL), and *t*BuOH was removed under reduced pressure. The pH of the remaining aqueous solution was adjusted to pH = 2 with aq. HCl (1 M) and the solution was extracted with CH_2Cl_2 (5 x 50 mL). The combined organic layers were dried over Na_2SO_4, filtered, and the solvent was removed under reduced pressure. The crude product was purified by column chromatography (SiO_2, *n*hexane/EtOAc 7:3 → 1:1, *v*/*v*) to afford acid **15** as a colorless solid (749 mg, 1.64 mmol, 82%).

m.p.: 179–180 °C (EtOAc).

TLC: R_f = 0.60 (*n*hexane/EtOAc = 1:1, *v*/*v*).

^{1}H-NMR: (400 MHz, $CDCl_3$) δ [ppm] = 4.57 (dd, *J* = 11.1, 5.3 Hz, 1H), 2.43 (ddd, *J* = 15.5, 10.1, 5.2 Hz, 1H), 2.28 (ddd, *J* = 15.9, 9.6, 6.5 Hz, 1H), 2.05 (s, 3H), 2.02–1.72 (m, 4H), 1.67–1.48 (m, 7H), 1.45–1.22 (8H), 1.17–1.03 (m, 2H), 0.96 (s, 3H), 0.90 (s, 3H), 0.89 (s, 3H), 0.88 (s, 3H), 0.85 (s, 3H), 0.79 (dd, *J* = 12.6 Hz, 2.6 Hz, 1H), 0.58 (d, *J* = 4.2 Hz, 1H), 0.34 (d, *J* = 4.2 Hz, 1H).

^{13}C-NMR: (101 MHz, $CDCl_3$) δ [ppm] = 179.1, 171.2, 80.8, 52.2, 49.0, 48.0, 47.3, 45.5, 39.6, 35.8, 35.6, 33.0, 31.7, 31.2, 31.1, 29.9, 28.2, 26.9, 26.6, 26.1, 25.9, 25.6, 21.5, 21.1, 20.2, 19.4, 18.2, 18.0, 15.3.

IR: $\tilde{\nu}$ [cm^{-1}] = 3300 (b), 2934 (w), 1742 (s), 1697 (m), 1375 (m), 1281 (s), 1246 (w), 1148 (w), 1136 (w), 1042 (w), 1032 (w), 968 (w).

HRMS: (ESI-TOF); *m*/*z* calculated for $C_{29}H_{45}O_4^-$ $[M-H^+]^-$: 457.3323,
found: 457.3329.

Opt. act.: $[\alpha]_D^{20}$ = +53.5 (*c* = 0.60, $CHCl_3$).

Synthesis of 4,4,14α-trimethyl-3β-acetoxy-9β,19-cyclo-5α-cholan-24-oic acid (**15**) and 3-*O*-acetylcycloartan-24-one (**16**) starting from γ-oryzanol

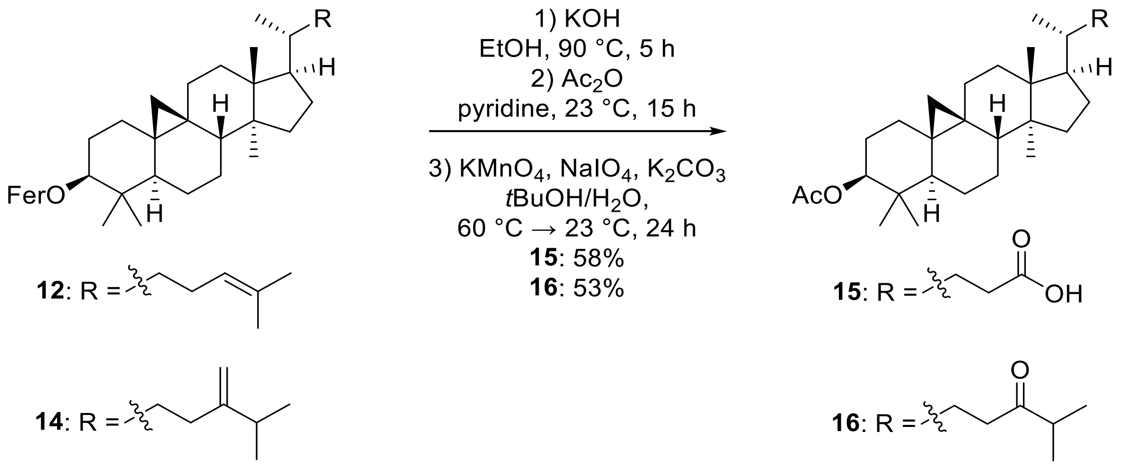

γ-Oryzanol (30.0 g), containing cycloartenyl ferulate (**12**) (40% by weight, 19.9 mmol, 1.0 eq.) and 24-methylenecycloartanyl ferulate (**14**) (40% by weight, 19.5 mmol, 1.0 eq.), was dissolved in EtOAc:EtOH (3:1, 40 mL) at 80 °C and allowed to crystallize over 1 h. The crystals (24.5 g) were then suspended in EtOH (690 mL) and aq. KOH (10 M, 76.6 mL) was added at 23 °C. The resulting solution was stirred for 5 h at 90 °C. Then the solvent was removed under reduced pressure and the resulting solid was redissolved in CH_2Cl_2 (500 mL). The organic phase was washed successively with sat. aq. $NaHCO_3$ (100 mL) and brine (100 mL), dried over Na_2SO_4, filtered, and the solvent was removed under reduced pressure. The crude product (16.7 g) was dissolved in pyridine (170 mL), acetic anhydride (170 mL) was added, and the solution was stirred for 15 h at 23 °C. After this time, all volatiles were removed under reduced pressure and the colorless solid was redissolved in CH_2Cl_2 (200 mL). The organic phase was washed with brine (80 mL), dried over Na_2SO_4, filtered, and the solvent was removed under reduced pressure.

The resulting solid (18.0 g) was dissolved in *t*BuOH (820 mL), K_2CO_3 (15.9 g, 115 mmol, 5.8 eq.) was added and the resulting suspension was heated to 60 °C. Subsequently, a suspension of $KMnO_4$ (2.43 g, 15.4 mmol, 0.8 eq.) and $NaIO_4$ (38.3 g, 9.52 mmol, 9.0 eq.) in water (475 mL) was added carefully, after which the reaction was stirred for 2 h at 60 °C and then for 22 h at 23 °C. The reaction was quenched with aq. $NaHSO_3$ (10wt%, 800 mL) and *t*BuOH was removed under reduced pressure. The pH of the remaining aqueous solution was adjusted to pH = 2 with aqueous HCl (1 M) and the solution was extracted with CH_2Cl_2 (5 x 500 mL). The combined organic phase was dried over Na_2SO_4, filtered, and the solvent

was removed under reduced pressure. The crude product was purified by column chromatography (SiO_2, *n*hexane/EtOAc 19:1 → 1:1, *v*/*v*). Acid **15** as a yellow solid (6.37 g) was washed by suspending it in EtOAc (20 mL) for 10 min in an ultrasonic bath and filtered through fritted glass to afford a colorless solid (5.26 g, 11.5 mmol, 58% over three steps). Ketone **16** was obtained as a colorless solid (5.05 g, 10.4 mmol, 53% over three steps).

All analytical data of acid **15** was identical to the data from the previous procedure.

ketone **16**

**TLC:**	R_f = 0.61 (heptane/EtOAc = 4:1, *v*/*v*).
**^{1}H-NMR:**	(400 MHz, $CDCl_3$) δ [ppm] = 4.53 (dd, *J* = 11.2, 4.6 Hz, 1H), 2.58 (hept, *J* = 6.9 Hz, 1H), 2.45 (ddd, *J* = 16.6, 10.0, 5.2 Hz, 1H), 2.34 (ddd, *J* = 16.3, 9.6, 6.1 Hz, 1H), 2.01 (s, 3H), 2.00–1.83 (m, 2H), 1.78–1.68 (m, 2H), 1.62–1.45 (m, 7H), 1.38–1.18 (m, 8H), 1.12–1.07 (m, 1H), 1.05 (d, *J* = 6.9 Hz, 6H), 0.92 (s, 3H), 0.86 (s, 3H), 0.85 (s, 3H), 0.82 (d, *J* = 6.4 Hz, 3H), 0.81 (s, 3H), 0.76 (dd, *J* = 12.6, 2.6 Hz, 1H), 0.54 (d, *J* = 4.2 Hz, 1H), 0.30 (d, *J* = 4.2 Hz, 1H).
**^{13}C-NMR:**	(101 MHz, $CDCl_3$) δ [ppm] = 215.4, 170.9, 80.7, 52.3, 48.9, 47.9, 47.2, 45.3, 40.9, 40.9, 39.5, 37.6, 35.8, 35.5, 32.9, 31.7, 30.2, 29.8, 28.1, 26.9, 26.5, 26.0, 25.9, 25.5, 21.4, 21.0, 20.1, 19.3, 18.5, 18.4, 18.2, 18.1, 15.2.
**HRMS:**	(ESI-TOF); *m*/*z* calculated for $C_{32}H_{52}O_3Na^+$ $[M+Na]^+$: 507.3809, found: 507.3813.

The spectroscopic data are in agreement with those reported in [3].

Synthesis of 4,4,14α-trimethyl-3β-hydroxy-9β,19-cyclo-5α-cholan-24-oic acid (**36**)

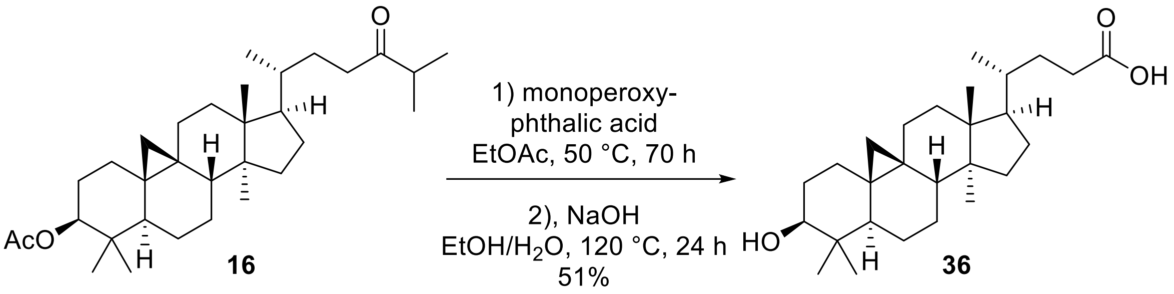

Na_2CO_3 (16.4 g, 155 mmol, 15 eq.) was dissolved in H_2O (77 mL) and H_2O_2 (30%, 19.5 mL, 186 mmol, 18 eq.) was added to the solution at 0 °C. Phthalic anhydride (22.9 g, 155 mmol, 15 eq.) was then added in one portion at -5 °C and the reaction mixture was stirred for 40 min at this temperature. The suspension was diluted with EtOAc (110 mL) and acidified with H_2SO_4 (95%, 9.3 mL). The organic phase was separated and the aqueous phase was extracted with EtOAc (2 x 50 mL). The combined organic phase was washed with $(NH_4)_2SO_4$, dried over $MgSO_4$, and filtered.[4] Ketone **16** (5.00 g, 10.3 mmol, 1.0 eq.) was added to the solution at 23 °C and the reaction mixture was stirred for 70 h at 45 °C. After this time, the reaction was quenched with sat. aq. $Na_2S_2O_3$ (50 mL), the organic phase was separated, and the aqueous phase was extracted with EtOAc (3 x 100 mL). The combined organic phases were was washed consecutively with sat. aq. Na_2CO_3 (2 x 50 mL) and NaOH (0.5 M, 2 x 50 mL), dried over Na_2SO_4, filtered, and the solvent was removed under reduced pressure.
The crude product (5.23 g) was dissolved in EtOH (177 mL), KOH (20 M in H_2O, 20 mL) was added, and the solution was stirred for 24 h at 100 °C. After this time, all volatiles were removed under reduced pressure and the yellowish solid was redissolved in EtOAc (200 mL) and H_2O (100 mL). The pH was adjusted to pH = 1 with aqueous HCl (1 M) and the solution was extracted with EtOAc (5 x 100 mL). The combined organic phase was dried over Na_2SO_4, filtered, and the solvent was removed under reduced pressure. The crude product was purified by column chromatography (SiO_2, heptane/EtOAc 1:1, *v*/*v*) to afford acid **36** as a colorless solid (2.23 g, 5.23 mmol, 51%).

m.p.: 224–225 °C (EtOAc).

TLC: R_f = 0.38 (heptane/EtOAc = 1:1, *v*/*v*).

**^{1}H-NMR:**	(400 MHz, $CDCl_3$) δ [ppm] = 3.29 (dd, J = 3.29, 1H), 2.43 (ddd, J = 15.4, 10.1, 5.2 Hz, 1H), 2.28 (ddd, J = 15.8, 9.6, 6.5 Hz, 1H), 2.02–1.73 (m, 4H), 1.64–1.54 (m, 6H), 1.50 (dd, J = 12.3, 4.7 Hz, 1H), 1.45–1.24 (m, 8H), 1.15–1.04 (m, 2H), 0.97 (s, 3H), 0.97 (s, 3H), 0.89 (s, 3H), 0.89 (d, J = 5.7 Hz, 3H), 0.81 (s, 3H), 0.80–0.76 (m, 1H), 0.55 (d, J = 4.2 Hz, 1H), 0.33 (d, J = 4.2 Hz, 1H).
**^{13}C-NMR:**	(101 MHz, $CDCl_3$) δ [ppm] = 179.0, 79.0, 52.2, 49.0, 48.1, 47.2, 45.5, 40.6, 35.8, 35.7, 33.0, 32.1, 31.2, 31.1, 30.5, 30.0, 28.2, 26.6, 26.2, 26.1, 25.6, 21.3, 20.1, 19.5, 18.2, 18.0, 14.2.
**IR:**	$\tilde{\nu}$ [cm^{-1}] = 3416 (br), 2959 (m), 2930 (s), 2862 (w), 1711 (s), 1381 (w), 1368 (w), 1269 (w), 1179 (m), 1103 (w), 1096 (w), 1047 (m), 1024 (m), 1009 (w), 993 (m), 907 (w), 665 (w), 569 (w).
**HRMS:**	(ESI-TOF); m/z calculated for $C_{27}H_{43}O_3^-$ [M-H$^+$]$^-$: 415.3218, found: 415.3219.
**Opt. act.:**	$[\alpha]_D^{20}$ = +45.4 (c = 0.59, pyridine).

Synthesis of 4,4,14α-trimethyl-3β-acetoxy-9β,19-cyclo-5α-cholan-24-oic acid (**15**)

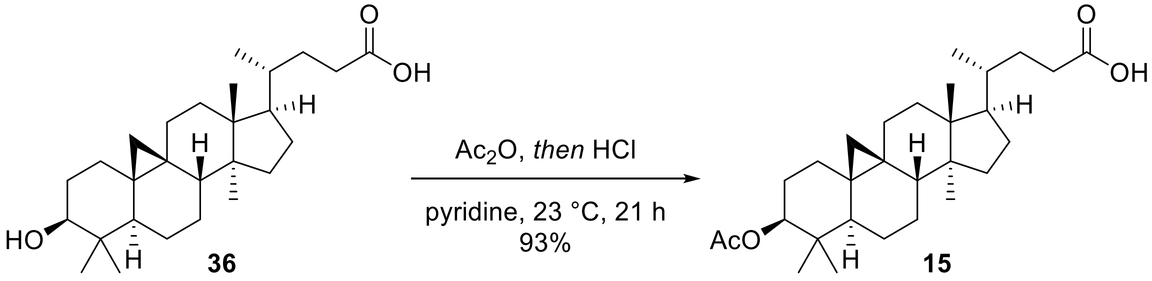

Alcohol **36** (2.23 g, 5.35 mmol, 1.0 eq.) was dissolved in pyridine (43 mL) and acetic anhydride (43 mL) was added at which point the mixture was stirred for 18 h at 23 °C. Then HCl (1 M, 40 mL) was added and the mixture was stirred for 3 h at 23 °C. The solvent was then removed under reduced pressure and the resulting colorless solid was redissolved in EtOAc (100 mL). The organic phase was washed with HCl (1 M, 40 mL), sat. aq. $NaHCO_3$ (40 mL) and brine (20 mL), dried over Na_2SO_4, filtered, and the solvent was removed under reduced

pressure. The crude product was purified by column chromatography (SiO_2, heptane/EtOAc 1:1, *v*/*v*) to obtain acid **15** as a colorless solid (2.33 g, 4.97 mmol, 93%).

All analytical data of acid **15** was identical to the data from the previous procedure.

Synthesis of 4,4,14α-trimethyl-9β,19-cyclo-24-nor-5α-chol-22-en-3β-yl acetate (**37**)

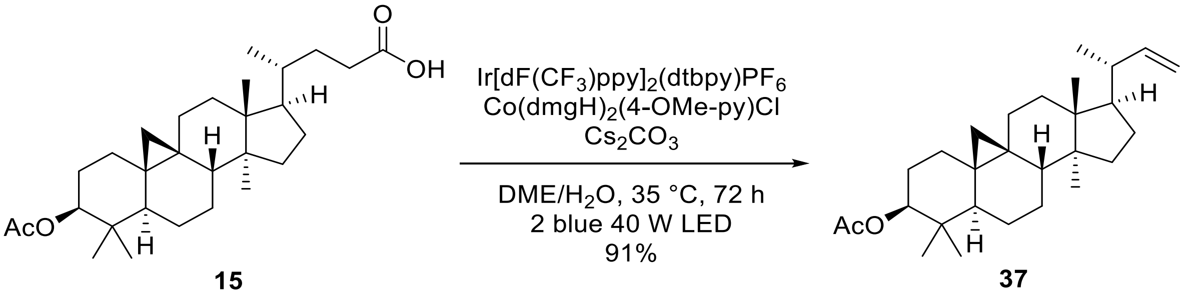

Acid **15** (2.19 g, 4.77 mmol, 1.0 eq.), [4,4′-*bis*(1,1-dimethylethyl)-2,2′-bipyridine-*N*1, *N*1′]*bis*[3,5-difluoro-2-[5-(trifluoromethyl)-2-pyridinyl-*N*]phenyl-C]iridium(III) hexafluorophosphate (53.5 mg, 7.7 µmol, 1 mol%), chloro(4-methoxypyridine)bis(dimethylglyoximato)cobalt(III) (103 mg, 238 µmol, 5 mol%) and Cs_2CO_3 (307 mg, 942 µmol, 0.2 eq.) were placed in a Schlenk tube under argon and the mixture was suspended in a degassed solution of dimethoxyethane (48 mL) and H_2O (2.6 mL). The suspension was stirred for 72 h under irradiation with blue light (two 40 W Kessil LED lamps, placed at a 2 cm distance to the reaction flask, cooled with a 120 mm fan) at approx. 35 °C. After this time, the reaction solution was transferred to a round-bottomed flask, the organic solvent was removed under reduced pressure, and the so-obtained crude (as aqueous mixture) was redissolved in CH_2Cl_2 (50 mL). The organic phase was separated and the aqueous phase was extracted with CH_2Cl_2 (2 x 20 mL). The combined organic phases were dried over Na_2SO_4, filtered, and the solvent was removed under reduced pressure. The crude product was purified by column chromatography (SiO_2, *n*hexane/EtOAc 19:1, *v*/*v*) to afford alkene **37** as colorless crystals (1.79 g, 4.35 mmol, 91%).

m.p.: 150–151 °C (EtOAc).

TLC: R_f = 0.55 (*n*hexane/EtOAc = 19:1, *v*/*v*).

^{1}H-NMR: (400 MHz, $CDCl_3$) δ [ppm] = 5.68 (ddd, J = 17.1, 10.2, 8.3 Hz, 1H), 4.92 (ddd, J = 17.1, 2.0, 0.9 Hz, 1H), 4.82 (dd, J = 10.2, 2.0 Hz, 1H), 4.57 (dd, J = 11.3, 4.4 Hz, 1H), 2.12–1.95 (m, 2H), 2.05 (s, 3H), 1.80–1.71 (m, 2H), 1.67–1.58 (m, 4H), 1.57–1.50 (m, 3H), 1.39 (dd, J = 12.4, 4.4 Hz, 1H), 1.36–1.24 (m, 5H), 1.17–1.06 (m, 2H), 0.99 (s, 3H), 0.98 (s, 3H), 0.90 (s, 3H), 0.89 (s, 3H), 0.85 (s, 3H), 0.80 (dd, J = 12.6, 2.7 Hz, 1H), 0.58 (d, J = 4.3 Hz, 1H), 0.34 (d, J = 4.3 Hz, 1H).

^{13}C-NMR: (101 MHz, $CDCl_3$) δ [ppm] = 171.2, 145.7, 111.7, 80.8, 51.8, 49.1, 48.0, 47.3, 45.4, 41.8, 39.6, 35.7, 32.9, 31.8, 30.0, 28.5, 26.9, 26.6, 26.1, 26.0, 25.6, 21.5, 21.0, 20.3, 19.8, 19.4, 18.3, 15.3.

IR: $\tilde{\nu}$ [cm^{-1}] = 2955 (m), 2936 (m), 2866 (w), 1728 (s), 1371 (m), 1246 (s), 1223 (w), 1207 (w), 1098 (w), 1047 (w), 1026 (m), 997 (w), 980 (m), 970 (w), 955 (w), 914 (m).

HRMS: (ESI-TOF); m/z calculated for $C_{28}H_{44}O_2Na^+$ $[M+Na]^+$: 435.3234, found: 435.3228.

Opt. act.: $[\alpha]_D^{20}$ = +43.6 (c = 0.53, $CHCl_3$).

Synthesis of 4,4,14α-trimethyl-23-hydroxy-9β,19-cyclo-24-nor-5α-cholan-3β-yl acetate (**2**)

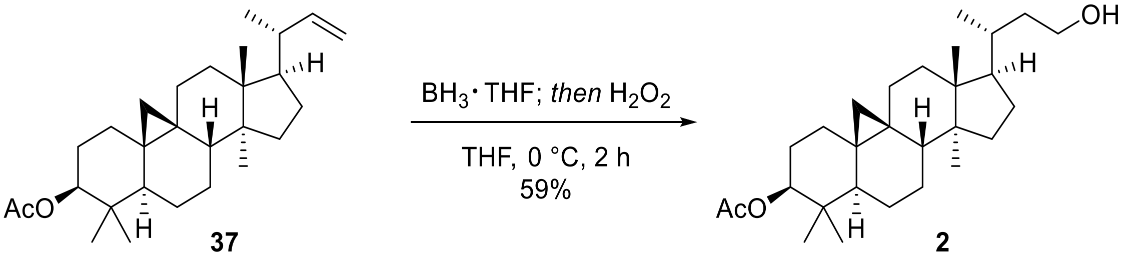

The reaction was carried out in parallel, employing multiple batches which were combined after dilution with H_2O.

Terminal alkene **37** (500 mg, 1.21 mmol, 1.0 eq.) was dissolved in THF (8.7 mL) and BH_3·THF (1.0 M, 4.24 mL, 4.24 mmol, 3.5 eq.) was added dropwise at 0 °C. The reaction mixture was stirred for 2 h at 0 °C, when H_2O_2 (35wt%, 3.70 mL) in phosphate buffer (pH = 7.0, 6.10 mL) was slowly added dropwise at 0 °C and stirring was continued for

30 min at 23 °C. The resulting solution was diluted with H_2O (10 mL) and extracted with CH_2Cl_2 (4 x 30 mL). The combined organic phases were dried over Na_2SO_4, filtered, and the solvent was removed under reduced pressure. The crude product was purified by column chromatography (SiO_2, *n*hexane/EtOAc 9:1, *v*/*v*) to afford the alcohol **2** as colorless crystals (310 mg, 720 mmol, 59%).

m.p.: 108–109 °C (EtOAc).

TLC: R_f = 0.48 (*n*hexane/EtOAc = 7:3, *v*/*v*).

^{1}H-NMR: (400 MHz, $CDCl_3$) δ [ppm] = 4.56 (dd, *J* = 10.8, 5.0 Hz, 1H), 3.73 (ddd, *J* = 10.4, 8.1, 4.7 Hz, 1H), 3.65 (dt, 10.4, 7.5 Hz, 1H), 2.05 (s, 3H), 2.03–1.88 (m, 2H), 1.80–1.71 (m, 2H), 1.65–1.58 (m, 5H), 1.57–1.49 (m, 4H), 1.39 (dd, *J* = 12.4, 4.4 Hz, 1H), 1.35–1.21 (m, 6H), 1.16–1.05 (m, 2H), 0.97 (s, 3H), 0.91 (s, 3H), 0.90 (d, *J* = 6.3 Hz, 3H), 0.89 (s, 3H), 0.84 (s, 3H), 0.79 (dd, *J* = 12.6, 2.6 Hz, 1H), 0.58 (d, *J* = 4.3 Hz, 1H), 0.34 (d, *J* = 4.3 Hz, 1H).

^{13}C-NMR: (101 MHz, $CDCl_3$) δ [ppm] = 171.0, 80.7, 61.0, 52.6, 48.8, 47.8, 47.1, 45.3, 39.4, 39.3, 35.5, 33.1, 32.8, 31.6, 29.7, 28.2, 26.8, 26.4, 25.9, 25.8, 25.4, 21.3, 20.9, 20.1, 19.3, 18.5, 17.9, 15.1.

IR: $\tilde{\nu}$ [cm^{-1}] = 3271 (b), 2961 (w), 2938 (m), 2866 (w), 1726 (s), 1371 (m), 1256 (s), 1242 (s), 1042 (m), 1024 (m), 997 (w), 970 (m).

HRMS: (ESI-TOF); m/z calculated for $C_{28}H_{46}O_3Na^+$ $[M+Na]^+$: 453.3339, found: 453.3331.

Opt. act.: $[\alpha]_D^{20}$ = +49.0 (*c* = 0.57, $CHCl_3$).

The spectroscopic data are in agreement with those reported in [5].

Synthesis of 4,4,14α-trimethyl-23-hydroxy-17,13-friedo-9β,19-cyclo-24-nor-5α-chol-12-en-3β-yl acetate (**17**)

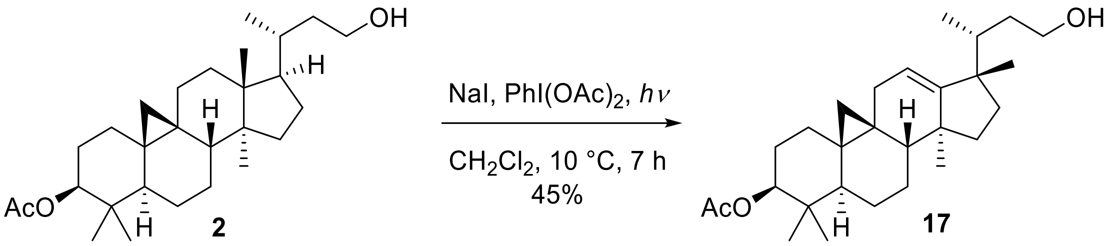

The reaction was carried out in parallel, employing multiple batches which were combined after the addition of sat. aq. $Na_2S_2O_3$.

Alcohol **2** (70.0 mg, 163 µmol, 1.0 eq.) was placed in a flame-dried 10 mL Schlenk tube, dissolved in CH_2Cl_2 (3.7 mL), and (diacetoxyiodo)benzene (209 mg, 650 µmol, 4.0 eq.) and NaI (97.5 mg, 650 µmol, 4.0 eq.) were added successively. The purple reaction mixture was stirred for 7 h at 10 °C under irradiation with a 45 W compact fluorescent lamp at which time the reaction was quenched with sat. aq. $Na_2S_2O_3$ (3.9 mL). The mixture was then transferred to a round-bottomed flask containing EtOAc (5 mL) and stirred for 30 min at 23 °C. The organic phase was separated and the aqueous phase was extracted with EtOAc (3 x 10 mL). The combined organic phases were dried over Na_2SO_4, filtered, and the solvent was removed under reduced pressure. The crude product was purified by column chromatography (1st SiO_2, heptane/EtOAc 8:2, *v*/*v*, 2nd SiO_2 impregnated with $AgNO_3$ (20%), CH_2Cl_2/MeOH 500:1, *v*/*v*)) to afford alkene **17** as a colorless oil (31.5 mg, 73.5 µmol, 45%).

TLC: R_f = 0.44 (*n*hexane/EtOAc = 7:3, *v*/*v*).
impregnated with $AgNO_3$ (25%): R_f = 0.23 (CH_2Cl_2/MeOH = 100:2, *v*/*v*).

^{1}H-NMR: (400 MHz, $CDCl_3$) δ [ppm] = 5.40 (dd, *J* = 7.7, 2.6 Hz, 1H), 4.59 (dd, *J* = 11.2, 4.2 Hz, 1H), 3.75 (ddd, *J* = 10.3, 8.2, 4.7 Hz, 1H), 3.62 (dt, *J* = 10.3, 7.6 Hz, 1H), 2.42 (dd, *J* = 15.7, 2.6 Hz, 1H), 2.05 (s, 3H), 1.90 (dtd, *J* = 13.3, 8.1, 2.5 Hz, 1H), 1.80–1.72 (m, 2H), 1.70–1.60 (m, 3H), 1.57–1.48 (m, 5H), 1.41–1.34 (m, 3H), 1.30 (dt, *J* = 12.2, 2.6 Hz, 1H), 1.22–1.14 (m, 2H), 1.13–1.06 (m, 1H), 1.01 (s, 3H), 0.89 (s, 3H), 0.87 (s, 3H), 0.87 (s, 3H), 0.86 (s, 3H), 0.85–0.82 (m, 1H), 0.53 (d, *J* = 4.4 Hz, 1H), 0.18 (d, *J* = 4.4 Hz, 1H).

^{13}C-NMR: (101 MHz, $CDCl_3$) δ [ppm] = 171.1, 159.8, 115.3, 80.7, 62.4, 50.3, 49.5, 47.3, 46.7, 39.3, 38.0, 35.5, 35.2, 32.7, 31.7, 31.2, 28.0, 26.7, 26.5, 26.0, 25.7, 25.7, 22.1, 21.5, 19.8, 17.5, 15.3, 15.1.

IR: $\tilde{\nu}$ [cm^{-1}] = 3503 (b), 2941 (m), 2868 (w), 1730 (m), 1709 (s), 1456 (w), 1375 (m), 1263 (s), 1244 (s), 1059 (m), 1032 (s), 997 (w), 974 (m), 731 (w).

HRMS: (ESI-TOF); *m/z* calculated for $C_{28}H_{44}O_3Na^+$ $[M+Na]^+$: 451.3183, found: 451.3190.

Opt. act.: $[\alpha]_D^{20}$ = +39.7 (c = 0.95, $CHCl_3$).

Synthesis of 4,4,14α-trimethyl-23-oxo-17,13-friedo-9β,19-cyclo-24-nor-5α-chol-12-en-3β-yl acetate (**38**)

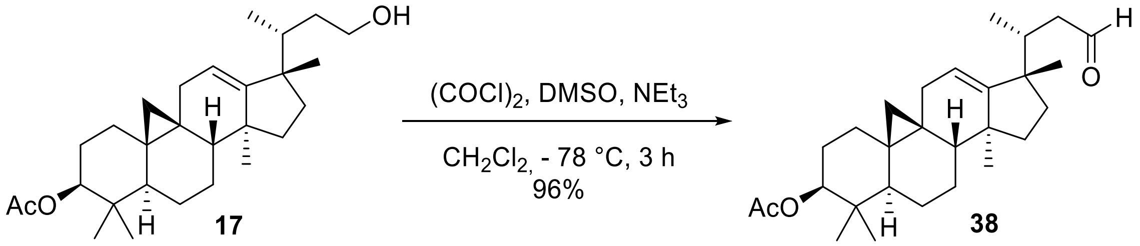

Oxalyl chloride (320 µL, 3.55 mmol, 5.0 eq.) was dissolved in CH_2Cl_2 (12 mL), DMSO (705 µL, 9.93 mmol, 14 eq.) was added at -78 °C, and the resulting solution was stirred for 20 min at -78 °C. Alcohol **17** (304 mg, 709 µmol, 1.0 eq.) in CH_2Cl_2 (4.0 mL) was then added dropwise and the resulting solution was stirred for 90 min at -78 °C. After this time, NEt_3 (3.5 mL) was added and the reaction solution was allowed to warm to 23 °C over 1 h. The reaction mixture was then diluted with heptane/toluene (3:1, *v*/*v*, 20 mL) and filtered through Celite®. The solvent of the filtrate was removed under reduced pressure and the so-obtained crude was dispersed in EtOAc (10 mL). The mixture was filtered again through Celite®, the solvent of the filtrate was removed under reduced pressure, and the crude was redissolved in heptane/toluene (3:1, 10 mL). The slightly cloudy solution was filtered a third time through Celite® and solvent of the filtrate was removed under reduced pressure to obtain

crude aldehyde **38** as a yellowish oil (291 mg, 682 µmol, 96%) which was used in the next reaction without further purification.

**TLC:**	R_f = 0.31 (*n*hexane/EtOAc = 9:1, *v*/*v*).
**¹H-NMR:**	(400 MHz, $CDCl_3$) δ [ppm] = 9.77 (dd, *J* = 3.3, 1.1 Hz, 1H), 5.46 (dd, *J* = 7.7, 2.6 Hz, 1H), 4.59 (dd, *J* = 11.2, 4.2 Hz, 1H), 2.68–2.62 (m, 1H), 2.42 (dd, *J* = 15.6, 2.7 Hz, 1H), 2.27–2.19 (m, 1H), 2.14 (ddd, *J* = 15.7, 10.4, 3.3 Hz, 1H), 2.06 (s, 3H), 1.81–1.73 (m, 2H), 1.68–1.63 (m, 1H), 1.58–1.48 (m, 4H), 1.41–1.35 (m, 2H), 1.29–1.22 (m, 4H), 1.14–1.08 (m, 1H), 1.01 (s, 3H), 0.91 (s, 3H), 0.89 (s, 3H), 0.88 (s, 3H), 0.87 (s, 3H), 0.86–0.83 (m, 1H), 0.53 (d, *J* = 4.4 Hz, 1H), 0.19 (d, *J* = 4.4 Hz, 1H).
**¹³C-NMR:**	(101 MHz, $CDCl_3$) δ [ppm] = 203.7, 171.1, 159.1, 116.2, 80.7, 50.1, 49.0, 47.4, 47.3, 46.6, 39.4, 37.7, 33.8, 33.1, 31.7, 31.2, 28.0, 26.7, 26.0, 26.0, 25.7, 25.7, 22.0, 21.5, 19.8, 17.8, 16.0, 15.4.
**IR:**	$\tilde{\nu}$ [cm^{-1}] = 2957 (w), 2934 (m), 1730 (s), 1458 (w), 1369 (m), 1242 (s), 1206 (w), 1099 (w), 1024 (m), 995 (w), 972 (m), 916 (w), 733 (m).
**HRMS:**	(ESI-TOF); *m*/*z* calculated for $C_{28}H_{42}O_3Na^+$ [M+Na]$^+$: 449.3026, found: 449.3032.
**Opt. act.:**	$[\alpha]_D^{20}$ = −16.7 (*c* = 0.60, $CHCl_3$).

Synthesis of 23-hydroxy-25-oxo-17,13-friedo-9β,19-cyclo-27-nor-5α-lanost-12-en-3β-yl acetate (**39**)

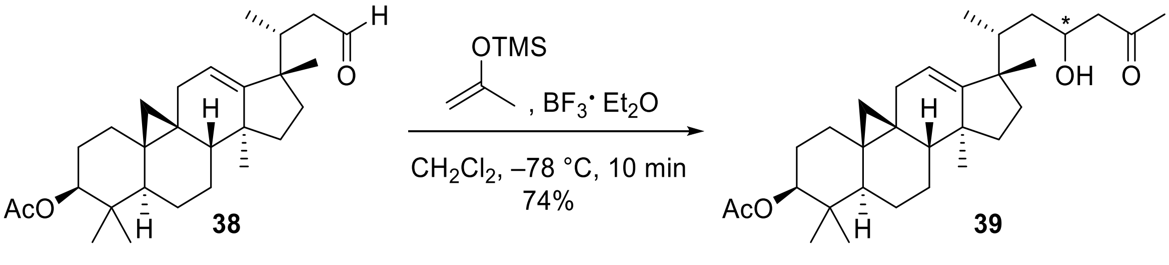

38 **39**

Aldehyde **38** (206 mg, 483 µmol, 1.0 eq.) was dissolved in CH_2Cl_2 (9.5 mL) and *iso*propenyloxytrimethylsilane (126 µL, 966 µmol, 2.0 eq.) was added. The solution was cooled to

-78 °C and $BF_3 \cdot Et_2O$ (0.1 M in CH_2Cl_2, 5.79 mL, 579 µmol, 1.2 eq.) was added dropwise. After stirring for 10 min at -78 °C, the reaction was quenched with water (10 mL), the solution was warmed to 23 °C and extracted with CH_2Cl_2 (3 x 30 mL). The combined organic phases were dried over Na_2SO_4, filtered, and the solvent was removed under reduced pressure. The crude product was purified by column chromatography (SiO_2, *n*hexane/EtOAc 9:1 → 4:1, *v*/*v*) to afford the β-hydroxyketone **39** as colorless crystals (174 mg, 359 µmol, 74%, *dr* = 1:1.33).

TLC: R_f = 0.17 (*n*hexane/EtOAc = 4:1, *v*/*v*).

^{1}H-NMR major epimer: (400 MHz, $CDCl_3$) δ [ppm] = 5.40 (td, *J* = 8.5, 2.6 Hz, 1H), 4.59 (dd, *J* = 11.1, 4.2 Hz, 1H), 4.12–4.07 (m, 1H), 2.71 (dd, *J* = 17.8, 2.3 Hz, 1H), 2.46 (dd, *J* = 17.6, 9.2 Hz, 1H), 2.44–2.38 (m, 1H), 2.19 (s, 3H), 2.06 (s, 3H), 1.80–1.60 (m, 5H), 1.58–1.51 (m, 3H), 1.51-1.50 (m, 1H), 1.50–1.45 (m, 2H), 1.41–1.32(m, 2H), 1.31–1.30 (m, 1H), 1.30–1.25 (m, 3H), 1.22–1.07 (m, 2H), 1.00 (s, 3H), 0.89 (s, 3H), 0.87 (s, 3H), 0.87 (s, 3H), 0.86 (d, *J* = 4.1 Hz, 3H), 0.56–0.51 (m, 1H), 0.18 (d, *J* = 4.4 Hz, 1H).

minor epimer: (400 MHz, $CDCl_3$) δ [ppm] = 5.40 (td, *J* = 8.5, 2.6 Hz, 1H), 4.59 (dd, *J* = 11.1, 4.2 Hz, 1H), 4.17–4.09 (m, 1H), 2.59* (d, *J* = 5.9 Hz, 2H), 2.44–2.38 (m, 1H), 2.17 (s, 3H), 2.06 (s, 3H), 1.93–1.87 (m, 1H), 1.80–1.60 (m, 5H), 1.58–1.51 (m, 3H), 1.50–1.45 (m, 2H), 1.41–1.32 (m, 2H), 1.30–1.25 (m, 3H), 1.22–1.07 (m, 2H), 1.00 (s, 3H), 0.96–0.93 (m, 1H), 0.90 (s, 3H), 0.89 (s, 3H), 0.88 (s, 3H), 0.86 (d, *J* = 4.1 Hz, 3H), 0.56–0.51 (m, 1H), 0.18 (d, *J* = 4.4 Hz, 1H).

^{13}C-NMR (101 MHz, $CDCl_3$) δ [ppm] = 210.4, 210.1*, 171.1, 171.1*, 159.8*, 159.7, 115.4, 115.3*, 80.7*, 80.7, 67.5, 65.7*, 51.3*, 50.4, 50.4*, 49.6* 49.6, 49.4, 49.3*, 47.4, 47.3*, 46.8, 46.7*, 39.4, 39.4*, 38.9, 38.8*, 38.0, 38.0*, 36.4, 34.5*, 32.7*, 32.6, 31.7*, 31.7, 31.2, 31.2*, 31.0, 30.9*, 28.0*, 28.0, 26.7*, 26.7, 26.6*, 26.0, 26.0*, 25.8*, 25.7, 25.7*, 22.1*, 22.1, 21.5*, 21.5, 19.8*, 19.8, 17.5, 17.3*, 17.3, 15.8, 15.4, 15.4*, 14.9. *Signals of minor epimer are marked with an *.*

HRMS (ESI-TOF); *m*/*z* calculated for $C_{31}H_{48}O_4Na^+$ $[M+Na]^+$: 507.3445,

found: 507.3455.

Synthesis of 23,25-dioxo-17,13-friedo-9β,19-cyclo-27-nor-5α-lanost-12-en-3β-yl acetate (**18**)

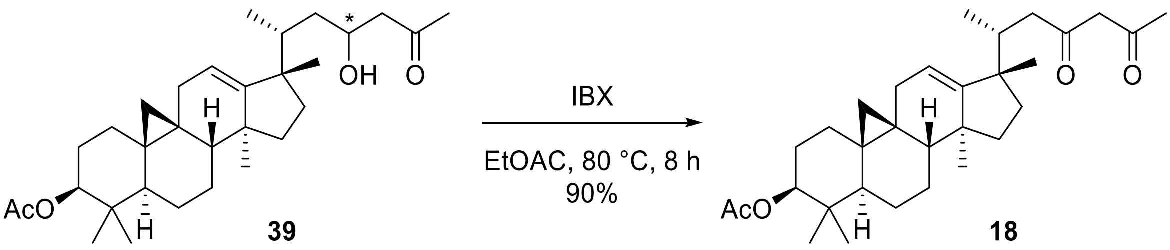

β-Hydroxyketone **39** (154 mg, 318 µmol, 1.0 eq.) was dissolved in EtOAc (2.3 mL), 2-iodoxybenzoic acid (281 mg, 954 µmol, 3.0 eq.) was added and the suspension was stirred for 8 h at 80 °C. The suspension was then allowed to cool to 23 °C, filtered twice through plugs of silica, washed with EtOAc (2 x 5 mL) and the solvent was removed under reduced pressure. Diketone **18** was obtained as a yellow oil which was used in the next reaction without further purification (154 mg, 286 µmol, 90%; yield corrected based on app. 10wt% impurity).

m.p.: 123–124 °C (EtOAc).

TLC: R_f = 0.39 (*n*hexane/EtOAc = 9:1, *v*/*v*).

^{1}H-NMR: Enol form: (400 MHz, $CDCl_3$) δ [ppm] = 15.58 (s, 1H), 5.49 (s, 1H), 5.45 (dd, *J* = 7.7, 2.6 Hz, 1H), 4.59 (dd, *J* = 11.1, 4.2 Hz, 1H), 2.62 (dd, *J* = 13.9, 3.0 Hz, 1H), 2.42 (dd, *J* = 15.5, 2.6 Hz, 1H), 2.24 (s, 1H), 2.09 (m, 1H), 2.06 (s, 3H), 2.05 (s, 3H), 1.83–1.74 (m, 2H), 1.66–1.50 (m, 7H), 1.37–1.24 (m, 5H), 1.02 (s, 3H), 0.89 (s, 3H), 0.89 (s, 3H), 0.88 (s, 3H), 0.86 (s, 3H), 0.85 (m, 1H), 0.53 (d, *J* = 4.4 Hz, 1H), 0.19 (d, *J* = 4.4 Hz, 1H).

^{13}C-NMR: Enol form: (101 MHz, $CDCl_3$) δ [ppm] = 195.2, 191.2, 171.1, 159.2, 116.0, 100.8, 80.7, 50.1, 49.3, 47.4, 46.6, 41.6, 39.4, 37.7, 36.7, 33.1,

31.2, 29.9, 28.0, 26.7, 26.0, 25.9, 25.7, 25.7, 25.1, 22.0, 21.5, 19.8, 17.8, 15.4, 15.2.

IR: $\tilde{\nu}$ [cm^{-1}] = 2951 (w), 2928 (m), 1722 (s), 1609 (b), 1458 (w), 1445 (w), 1373 (m), 1246 (s), 1020 (m), 997 (w), 982 (w), 972 (m).

HRMS: (ESI-TOF); *m/z* calculated for $C_{31}H_{46}O_4Na^+$ $[M+Na]^+$: 505.3288, found: 505.3284.

Opt. act.: $[\alpha]_D^{20}$ = +24.4 (c = 0.44, $CHCl_3$).

Synthesis of 23,25-dioxo-17,13-friedo-9β,19-cyclo-27-nor-5α-lanost-12-en-3β-ol (**40**)

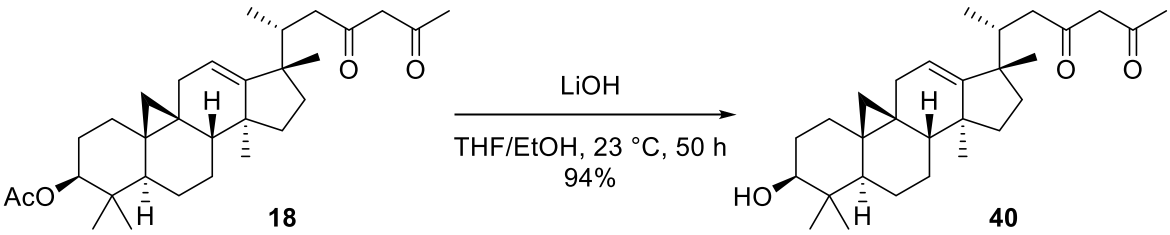

1,3-Diketone **18** (90% purity, 154 mg, 286 μmol, 1.0 eq.) was dissolved in THF/EtOH (3:2, 8 mL), LiOH (36.3 mg, 1.44 mmol, 5.0 eq.) was added at 23 °C and the resulting solution was stirred for 50 h at 23 °C. The reaction mixture was then diluted with phosphate buffer (pH = 7, 5 mL) and extracted with EtOAc (3 x 5 mL). The combined organic phases were dried over Na_2SO_4, filtered, and the solvent was removed under reduced pressure. Diketone **40** was obtained as a yellow oil which was used in the next reaction without further purification (141 mg, 272 μmol, 94%; yield corrected based on app. 15wt% impurity).

TLC: R_f = 0.36 (*n*hexane/EtOAc = 4:1, *v*/*v*).

^{1}H-NMR: Enol form: (500 MHz, $CDCl_3$) δ [ppm] = 15.58 (s, 1H), 5.49 (s, 1H), 5.45 (dd, J = 7.7, 2.6 Hz, 1H), 3.31 (dd, J = 11.2, 4.2 Hz, 1H), 2.62 (dd, J = 13.9, 3.1 Hz, 1H), 2.41 (dd, J = 15.6, 2.6 Hz, 1H), 2.33 (m, 1H), 2.24 (s, 1H), 2.09 (m, 1H), 2.05 (s, 3H), 1.84 (dd, J = 13.9, 11.2 Hz, 1H), 1.77 (m, 1H), 1.66–1.59 (m, 2H), 1.57–1.52 (m, 2H), 1.50 (d, J = 7.7 Hz, 1H), 1.40–1.35 (m, 2H), 1.33–1.28 (m, 4H), 1.20–1.16 (m, 1H), 1.02 (s, 3H),

	1.00 (s, 3H), 0.89 (s, 3H), 0.86 (s, 3H), 0.85 (m, 1H), 0.81 (s, 3H), 0.52 (d, $J = 4.4$ Hz, 1H), 0.17 (d, $J = 4.4$ Hz, 1H).
**^{13}C-NMR:**	(126 MHz, $CDCl_3$) δ [ppm] = 195.2, 191.2, 159.2, 116.1, 100.8, 78.9, 50.1, 49.3, 47.3, 46,6, 41.6, 40.4, 37.7, 36.6, 33.1, 31.7, 31.5, 30.2, 29.9, 26.2, 25.9, 25.7, 25.1, 22.8, 22.0, 20.0, 17.9, 15.2, 14.2.
**HRMS:**	(ESI-TOF); *m/z* calculated for $C_{29}H_{44}O_3Na^+$ $[M+Na]^+$: 463.3183, found: 463.3199.
**Opt. act.:**	$[\alpha]_D^{20}$ = +19.5 (c = 0.25, $CHCl_3$).

Synthesis of 4,4,14α-trimethyl-22-(4-((phenylsulfonyl)methyl)furan-2-yl)-17,13-friedo-9β,19-cyclo-23,24-nor-5α-chol-12-en-3β-ol (**19**)

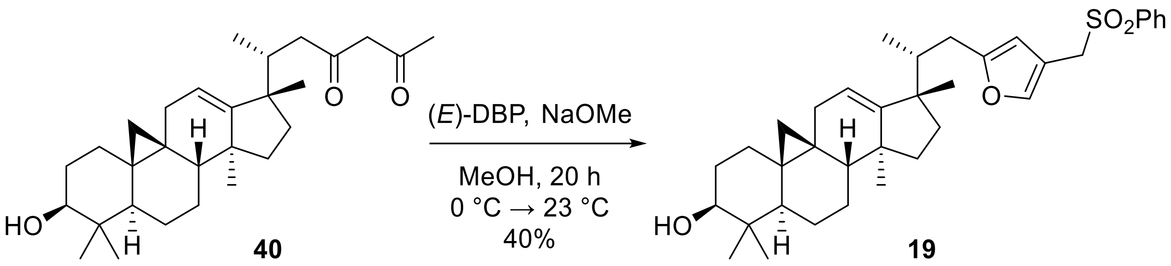

The reaction was carried out in parallel, employing multiple batches which were combined after quenching with sat. aq. NH_4Cl.

1,3-Diketone **40** (85% purity, 40.0 mg, 77.2 µmol, 1.0 eq.) was dissolved in MeOH/THF (4:1, 460 µL), the solution was cooled to 0 °C, and NaOMe (0.5 M in MeOH, 185 µL, 92.6 µmol, 1.2 eq.) was added dropwise. The resulting solution was allowed to warm to 23 °C and stirred there for 20 min, then cooled again to 0 °C when (*E*)-2,3-dibromo-1-(phenylsulfonyl)-1-propene (**41**) (26.2 mg, 77.2 µmol, 1.0 eq.) was added in one portion. The reaction solution was stirred for 2 h at 23 °C, then cooled again to 0 °C, when a second portion of NaOMe (0.5 M in MeOH, 216 µL, 108 µmol, 1.4 eq.) was added dropwise. After warming to 23 °C, stirring was continued for another 18 h. After this time, the reaction was quenched by adding sat. aq. NH_4Cl (1 mL), MeOH was removed under reduced pressure, and the aqueous phase was extracted with CH_2Cl_2 (4 x 5 mL). The combined organic phases were dried over Na_2SO_4, filtered, and all volatiles were removed under reduced pressure.

The so-obtained crude product was purified by column chromatography (SiO_2, CH_2Cl_2/MeOH 450:1, *v*/*v*) to afford furan **19** as colorless oil (17.9 mg, 31.0 µmol, 40%).

TLC: R_f = 0.61 (PE/EtOAc = 1:1, *v*/*v*).

^{1}H-NMR: (400 MHz, $CDCl_3$) δ [ppm] = 7.74 (d, *J* = 7.4 Hz, 2H), 7.62 (t, *J* = 7.5 Hz, 1H), 7.48 (t, *J* = 7.7 Hz, 2H), 7.05 (s, 1H), 5.88 (s, 1H), 5.44 (dd, *J* = 7.8, 2.6 Hz, 1H), 4.13 (s, 2H), 3.31 (dd, *J* = 10.9, 4.3 Hz, 1H), 2.87 (dd, *J* = 14.7, 2.7 Hz, 1H), 2.44 (dd, *J* = 16.5, 2.4 Hz, 1H), 2.11 (m, 1H), 1.90 (m, 1H), 1.79–1.73 (m, 2H), 1.69–1.62 (m, 2H), 1.60–1.57 (m, 3H), 1.54–1.57 (m, 3H), 1.38 (dd, *J* = 12.2, 5.5 Hz, 1H), 1.33–1.29 (m, 2H), 1.20 (m, 1H), 1.11 (d, *J* = 10.9 Hz, 1H), 1.05 (s, 3H), 1.00 (s, 3H), 0.88 (s, 3H), 0.85 (m, 1H), 0.82 (s, 3H), 0.76 (d, *J* = 6.7 Hz, 3H), 0.52 (d, *J* = 4.4 Hz, 1H), 0.18 (d, *J* = 4.4 Hz, 1H).

^{13}C-NMR: (101 MHz, $CDCl_3$) δ [ppm] = 159.5, 157.7, 141.1, 138.0, 133.8, 129.0, 128.8, 115.8, 113.2, 107.7, 78.9, 53.9, 50.2, 49.2, 47.3, 46.6, 40.4, 38.4, 37.8, 32.9, 31.7, 31.6, 31.0, 30.2, 28.0, 26.2, 25.9, 25.9, 25.7, 22.0, 20.0, 17.9, 15.1, 14.2.

IR: $\tilde{\nu}$ [cm^{-1}] = 3435 (b), 2920 (s), 2849 (m), 1717 (w), 1447 (w), 1308 (w), 1246 (w), 1155 (s), 1086 (m), 1020 (m), 810 (w), 754 (w), 687 (w), 607 (w), 548 (m), 527 (s).

HRMS: (ESI-TOF); *m/z* calculated for $C_{36}H_{49}O_4S^+$ $[M+H]^+$: 577.3346, found: 577.3356.

Opt. act.: $[\alpha]_D^{20}$ = + 6.17 (*c* = 0.47, $CHCl_3$).

Synthesis of 4,4,14α-trimethyl-22-(4-((phenylsulfonyl)methyl)furan-2-yl)-17,13-friedo-9β,19-cyclo-23,24-nor-5α-chol-12-en-3-one (**42**)

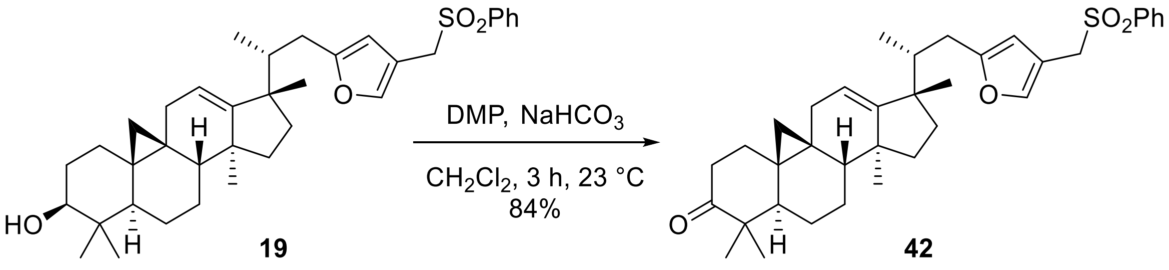

Alcohol **19** (41.2 mg, 71.4 μmol, 1.0 eq.) was dissolved in CH_2Cl_2 (700 μL), $NaHCO_3$ (21.0 mg, 250 μmol, 3.5 eq.) and Dess–Martin periodinane (95%, 41.5 mg, 92.9 μmol, 1.3 eq.) were added successively at 23 °C and the reaction mixture was stirred at this temperature for 2 h. The reaction was quenched with sat. aq. $Na_2S_2O_3$ (500 μL) and stirred for a further 30 min. The mixture was then extracted with CH_2Cl_2 (4 x 10 mL), the combined organic phases were washed with sat. aq. $NaHCO_3$ (10 mL), dried over Na_2SO_4, filtered, and the solvent was removed under reduced pressure. The crude product was purified by column chromatography (SiO_2, CH_2Cl_2/MeOH 500:1, *v*/*v*) to afford ketone **42** as a colorless oil (34.4 mg, 59.8 μmol, 84%).

TLC: R_f = 0.52 (PE/EtOAc = 7:3, *v*/*v*).

^{1}H-NMR: (600 MHz, $CDCl_3$) δ [ppm] = 7.74 (dd, *J* = 8.3, 1.3 Hz, 2H), 7.62 (ddt, *J* = 8.7, 7.2, 1.3 Hz, 1H), 7.48 (dd, *J* = 8.3, 7.4 Hz, 2H), 7.05 (d, *J* = 1.0 Hz, 1H), 5.89 (s, 1H), 5.46 (dd, *J* = 7.7, 2.6 Hz, 1H), 4,13 (d, *J* = 0.8 Hz, 2H), 2.87 (dd, *J* = 14.8, 3.1 Hz, 1H), 2.75 (td, *J* = 13.9, 6.4 Hz, 1H), 2.48 (dd, *J* = 15.5, 2.7 Hz, 1H), 2.31 (ddd, *J* = 13.8, 4.3, 2.6 Hz, 1H), 2.15 (dd, *J* = 14.7, 11.3 Hz, 1H), 1.97-1.88 (m, 2H), 1.79 (dt, *J* = 12.4, 8.2 Hz, 1H), 1.69 (dd, *J* = 12.1, 4.0 Hz, 1H), 1.63–1.50 (m, 6H), 1.46 (dd, *J* = 12.4, 5.8 Hz, 1H), 1.25–1.13 (m, 2H), 1.11 (s, 3H), 1.07 (s, 3H), 1.07 (s, 3H), 1.02–1.00 (m, 1H), 0.89 (s, 3H), 0.76 (d, *J* = 6.7 Hz, 3H), 0.74 (d, *J* = 4.7 Hz, 1H), 0.43 (d, *J* = 4.5 Hz, 1H).

^{13}C-NMR: (151 MHz, $CDCl_3$) δ [ppm] = 216.7, 159.8, 157.6, 141.1, 138.0, 133.8, 129.0, 128.8, 115.5, 113.2, 107.8, 53.9, 50.2, 50.2, 49.3, 48.7, 46.5, 38.4,

37.9, 37.4, 33.1, 32.9, 31.8, 31.0, 27.7, 26.2, 26.0, 25.7, 23.1, 22.5, 21.1, 20.4, 17.8, 15.1.

IR: $\tilde{\nu}$ [cm^{-1}] = 2935 (m), 2924 (s), 2853 (w), 1705 (s), 1458 (w), 1319 (w), 1261 (m), 1155 (s), 1112 (s), 1101 (s), 1020 (m), 806 (m), 689 (w), 549 (w), 549 (w), 529 (w).

HRMS: (ESI-TOF); *m/z* calculated for $C_{36}H_{46}O_4SNa^+$ $[M+Na]^+$: 597.3009, found: 597.3037.

Opt. act.: $[\alpha]_D^{20}$ = −1.23 (c = 0.33, $CHCl_3$).

Synthesis of 4,4,14α-trimethyl-22-(4-((phenylsulfonyl)methyl)furan-2-yl)-17,13-friedo-9β,19-cyclo-23,24-nor-5α-chol-12-en-3α-ol (**20**)

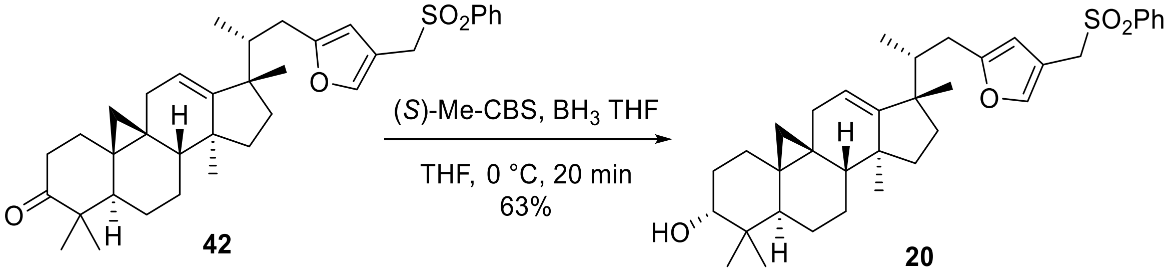

(S)-2-methyl-CBS-oxazaborolidine (31.9 mg, 109 µmol, 2.0 eq.) was dissolved in THF (1 mL), BH_3·THF (1 M, 98.3 µL, 98.3 µmol, 1.8 eq.) was slowly added dropwise at 0 °C and the mixture was stirred at 0 °C for 15 min. Ketone **42** (31.4 mg, 54.6 µmol, 1.0 eq.) in THF (1 mL) was then added dropwise at 0 °C and the resulting solution was stirred for 20 min at this temperature. The reaction was quenched by adding MeOH (2.0 mL) and all volatiles were removed under reduced pressure. The crude product was purified by column chromatography (SiO_2, CH_2Cl_2/ MeOH 450:1, *v*/*v*) to afford alcohol **20** (19.9 mg, 34.5 µmol, 63%) as colourless oil.

TLC: R_f = 0.69 (PE/EtOAc = 1:1, *v*/*v*).

^{1}H-NMR: (600 MHz, $CDCl_3$) δ [ppm] = 7.74 (dd, J = 8.4, 1.3 Hz, 2H), 7.61 (tt, J = 7.6, 1.2 Hz, 1H), 7.48 (t, J = 7.5 Hz, 2H), 7.05 (d, J = 1.0 Hz, 1H), 5.87

(d, J = 0.9 Hz, 1H), 5.44 (dd, J = 7.7, 2.6 Hz, 1H), 4.13 (d, J = 0.8 Hz, 2H), 3.49 (t, J = 2.3 Hz, 1H), 2.87 (dd, J = 14.9, 3.0 Hz, 1H), 2.43 (dd, J = 15.6, 2.6 Hz, 1H), 2.14 (dd, J = 14.7, 11.3 Hz, 1H), 1.95 (d, J = 8.8 Hz, 2H), 1.90 (ddt, J = 15.0, 8.2, 2.9 Hz, 1H), 1.81–1.74 (m, 2H), 1.66 (dt, J = 10.3, 3.2 Hz, 1H), 1.55–1.51 (m, 5H), 1.46 (dq, J = 12.9, 3.8 Hz, 1H), 1.40 (dd, J = 12.4, 5.9 Hz, 1H), 1.22–1.11 (m, 2H), 1.07 (dd, J = 8.9, 2.6 Hz, 1H), 1.05 (s, 3H), 0.98 (s, 3H), 0.89 (s, 3H), 0.88 (s, 3H), 0.84 (qd, J = 12.8, 2.2 Hz, 1H), 0.75 (d, J = 6.7 Hz, 3H), 0.49 (d, J = 4.3 Hz, 1H), 0.19 (d, J = 4.3 Hz, 1H).

^{13}C-NMR: (151 MHz, $CDCl_3$) δ [ppm] = 159.5, 157.7, 141.1, 137.9, 133.8, 129.0, 128.8, 115.8, 113.2, 107.7, 77.2, 53.9, 50.4, 49.2, 46.7, 41.3, 39.4, 38.5, 37.8, 32.9, 31.6, 31.0, 28.4, 27.9, 27.0, 26.5, 26.2, 26.0, 25.7, 21.8, 21.5, 20.0, 17.8, 15.1.

HRMS: (ESI-TOF); *m/z* calculated for $C_{36}H_{48}O_4SNa^+$ $[M+Na]^+$: 599.3166, found: 599.3148.

Synthesis of fortunefuroic acid I (**4**)

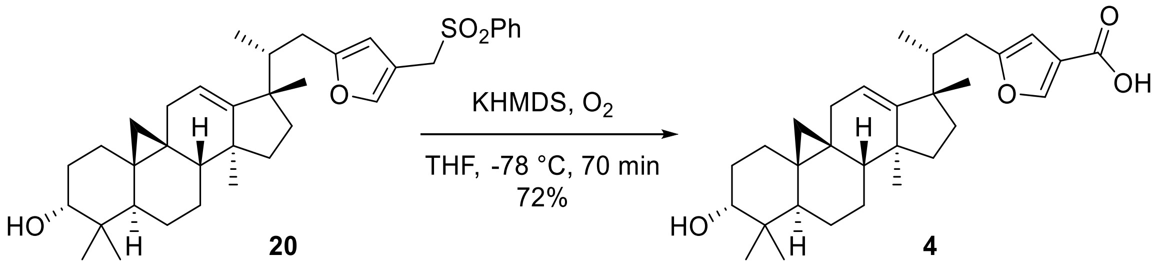

Sulfone **20** (17.3 mg, 30.0 µmol, 1.0 eq.) was dissolved in THF (350 µL) and KHMDS (0.5 M in toluene, 180 µL, 90.0 µmol, 3.0 eq.) was slowly added dropwise at -78 °C. After stirring for 25 min at this temperature, dry oxygen was sparged through the stirred mixture at -78 °C for 45 min using a 22-gauge needle. The solution was allowed to warm to 23 °C, where it was diluted with CH_2Cl_2 (1 mL) and quenched by adding a mixture of sat. aq. $NaHCO_3$ and sat. aq. Na_2CO_3 (1:1, *v*/*v*, 1 mL). The organic phase was separated, the aqueous phase was adjusted to pH = 2 with aq. HCl (1 M) and extracted with CH_2Cl_2 (4 x 1 mL). The combined

organic phases were dried over Na_2SO_4, filtered, and the solvent was removed under reduced pressure. The crude product was purified by column chromatography (SiO_2, heptane/EtOAc/AcOH 80:20:1, *v*/*v*) to afford fortunefuroic acid I (**4**) as a colorless amorphous solid (10.1 mg, 21.6 µmol, 72%).

TLC: R_f = 0.52 (heptane/EtOAc/AcOH = 50:50:1, *v*/*v*).

^{1}H-NMR: (600 MHz, $CDCl_3$) δ [ppm] = 7.96 (s, 1H), 6.36 (s, 1H), 3.5 (dd, *J* = 2.7, 2.7 Hz, 1H), 2.97 (dd, *J* = 14.9, 2.9 Hz, 1H), 2.45 (dd, *J* = 15.7, 2.6 Hz, 1H), 2.25 (dd, *J* = 14.8, 11.3 Hz, 1H), 1.99 (br dd, *J* = 12.3, 9.1 Hz, 1H), 1.97 (m, 1H), 1.96 (br ddd, *J* = 10.1, 2.8, 2.8 Hz, 1H), 1.81 (br dd, *J* = 12.5, 3.4 Hz, 1H), 1.81 (dd, *J* = 11.5, 3.5 Hz, 1H), 1.67 (ddd, *J* = 10.2, 3.1, 3.1 Hz, 1H), 1.55 (br dd, *J* = 11.3, 5.5 Hz, 1H), 1.55 (m, 1H), 1.54 (dd, *J* = 13.4, 8.2 Hz, 1H), 1.52 (m, 1H), 1.47 (dt, *J* = 12.9, 3.6 Hz, 1H), 1.41 (dd, *J* = 12.3, 6.0 Hz, 1H), 1.23 (ddd, *J* = 11.4, 6.2, 5.4 Hz, 1H), 1.16 (qd, *J* = 12.9, 2.6 Hz, 1H), 1.08 (s, 3H), 1.08 (br dd, *J* = 9.2, 7.8 Hz, 1H), 1.00 (s, 3H), 0.91 (s, 3H), 0.90 (s, 3H), 0.84 (d, *J* = 6.7 Hz, 3H), 0.84 (m, 1H), 0.50 (d, *J* = 4.3 Hz, 1H), 0.20 (d, *J* = 4.3 Hz, 1H).

^{13}C-NMR: (151 MHz, $CDCl_3$) δ [ppm] = 164.5, 159.3, 158.5, 147.2, 118.3, 115.8, 105.7, 77.1, 50.2, 49.1, 46.5, 41.1, 39.3, 38.2, 37.6, 32.9, 31.4, 30.8, 28.3, 27.8, 26.8, 26.3, 26.1, 25.6, 25.5, 21.7, 21.4, 19.8, 17.8, 14.9.

IR: $\tilde{\nu}$ [cm^{-1}] = 3356 (br), 3192 (br), 2922 (s), 2851 (s), 1697 (m), 1549 (w), 1467 (m), 1377 (m), 1261 (m), 1206 (m), 1136 (m), 1067 (m).

HRMS: (ESI-TOF); *m*/*z* calculated for $C_{30}H_{41}O_4^-$ $[M–H^+]^-$: 465.3010,
found: 465.2998.

The spectroscopic data are in agreement with those reported in [6].

Synthesis of 2,3-dibromo-1-(phenylsulfonyl)-1-propene (DBP, **41**)[7][8]

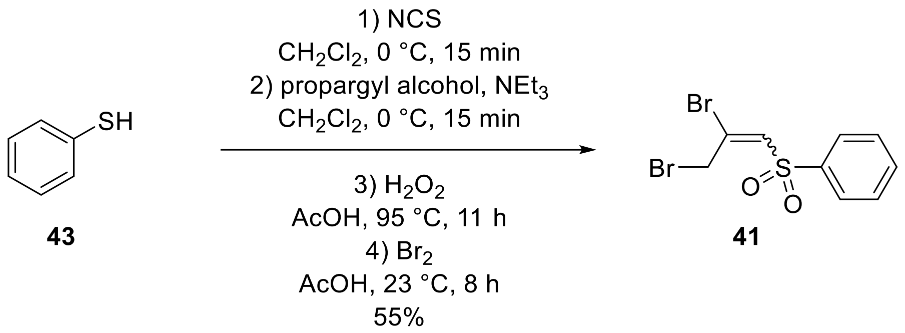

N-Chlorosuccinimide (782 mg, 5.85 mmol, 1.25 eq.) was dissolved in CH_2Cl_2 (17 mL), the solution was cooled to 0 °C, and thiophenol (**43**) (478 µL, 4.68 mmol, 1.0 eq.) was added dropwise. After stirring for 15 min at this temperature, the solvent was removed at 40 °C and 500 mbar using a rotary evaporator and the crude product was redissolved in *n*hexane (4 mL). The solution was filtered through Celite® and rinsed with *n*hexane (2 x 1 mL). The solvent was removed at 40 °C and 200 mbar and the resulting phenylsulfenyl chloride was used immediately in the next step.

To a solution of propargyl alcohol (270 µL, 4.57 mmol, 1.0 eq.) and NEt_3 (775 µL, 5.59 mmol, 1.23 eq.) in CH_2Cl_2 (18 mL), was added a solution of phenylsulfenyl chloride (668 mg, 4.62 mmol, 1.0 eq.) in CH_2Cl_2 (1.3 mL) dropwise at 0 °C. The resulting reaction mixture was stirred for 3 h at 23 °C. The solution was then washed with H_2O (3 x 5 mL), dried over Na_2SO_4, filtered, and the solvent was removed under reduced pressure.

The crude product was redissolved in acetic acid (2.6 mL), the solution was heated to 90 °C and H_2O_2 (w = 35%, 1.03 mL) was added dropwise while stirring. The reaction solution was stirred for 11 h at 95 °C, cooled to 23 °C, then diluted with H_2O (5 mL) and extracted with CH_2Cl_2 (4 x 10 mL). The combined organic phases were washed with H_2O (10 mL) and sat. aq. $NaHCO_3$ (10 mL), dried over Na_2SO_4, filtered, and the solvent removed under reduced pressure.

The crude product (707 mg) was dissolved in acetic acid (2.8 mL), bromine (223 µL, 4.35 mmol, 1.0 eq.) was added dropwise at 23 °C over 30 min and the solution was stirred for 8 h at 23 °C. The reaction solution was then diluted with H_2O (10 mL) and extracted with CH_2Cl_2 (4 x 10 mL). The combined organic phases were washed successively with sat. aq. $Na_2S_2O_3$ (2 x 10 mL), H_2O (10 mL) and sat. aq. $NaHCO_3$ (10 mL), dried over Na_2SO_4, filtered, and the solvent was removed under reduced pressure. The crude product was

purified by column chromatography (SiO_2, *n*hexane/EtOAc 4:1, *v*/*v*) to obtain DBP (**41**) as (*E*)-isomer (581 mg, 1.71 mmol, 37% over four steps) and (*Z*)-isomer (274 mg, 806 µmol, 18% over four steps).

(*E*)-Isomer

TLC: R_f = 0.61 (*n*hexane/EtOAc = 4:1, *v*/*v*).

^{1}H-NMR: (400 MHz, $CDCl_3$) δ [ppm] = 7.99–7.95 (m, 2H), 7.71 (tt, *J* = 7.4 Hz, 1.3 Hz, 1H), 7.63–7.58 (m, 2H), 6.77 (s, 1H), 4.92 (s, 2H).

^{13}C-NMR: (101 MHz, $CDCl_3$) δ [ppm] = 139.8, 137.8, 134.5, 133.9, 129.8, 128.0, 29.9.

HRMS: (ESI-TOF); *m*/*z* calculated for $C_9H_8O_2SBr_2Na^+$ $[M+Na]^+$: 360.8504 found: 360.8505.

(*Z*)-Isomer

TLC: R_f = 0.38 (*n*hexane/EtOAc = 4:1, *v*/*v*).

^{1}H-NMR: (400 MHz, $CDCl_3$) δ [ppm] = 7.96 (dd, *J* = 8.4, 1.3 Hz, 2H), 7.65 (t, *J* = 7.5 Hz, 1H), 7.55 (t, *J* = 7.8 Hz, 2H), 7.32 (s, 1H), 4.22 (d, *J* = 1.2 Hz, 2H).

^{13}C-NMR: (101 MHz, $CDCl_3$) δ [ppm] = 139.7, 134.2, 134.1, 133.9, 129.3, 128.2, 35.8.

HRMS: (ESI-TOF); *m*/*z* calculated for $C_9H_8O_2SBr_2Na^+$ $[M+Na]^+$: 360.8504 found: 360.8505.

The spectroscopic data are in agreement with those reported in [8].

Synthesis of parkeol (**3**)

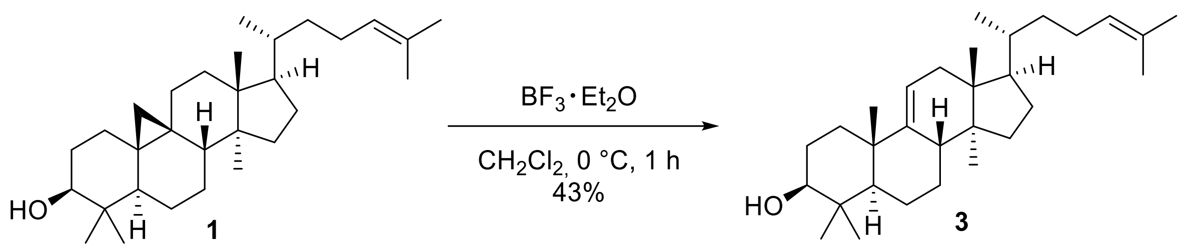

Cycloartenol (**1**) (30.0 mg, 69.7 µmol, 1.0 eq.) was dissolved in CH_2Cl_2 (1.0 mL), $BF_3 \cdot Et_2O$ (92.9 µL, 697 µmol, 10 eq.) was added dropwise at 0 °C for 15 min, and the solution was stirred at 0 °C for 45 min. The reaction was then quenched with sat. aq. Na_2CO_3 (1 mL), the organic phase was separated, and the aqueous phase was extracted with CH_2Cl_2 (3 x 3 mL). The combined organic phases were dried over Na_2SO_4, filtered, and the solvent was removed under reduced pressure. The crude product was purified by column chromatography (SiO_2 impregnated with $AgNO_3$ (20%), CH_2Cl_2/MeOH 600:1, *v*/*v*) to afford parkeol **3** as a white solid (12.9 mg, 32.3 µmol, 43%).

m.p.: 156–157 °C (CH_2Cl_2).

TLC: R_f = 0.46 (CH_2Cl_2/MeOH = 100:2, *v*/*v*).
impregnated with $AgNO_3$ (25%): R_f = 0.31 (CH_2Cl_2/MeOH = 100:2, *v*/*v*).

^{1}H-NMR: (400 MHz, $CDCl_3$) δ [ppm] = 5.22 (dt, *J* = 6.3, 2.0 Hz, 1H), 5.10 (ddq, *J* = 7.1, 5.6, 1.4 Hz, 1H), 3.21 (dd, *J* = 11.5, 4.4 Hz, 1H), 2.20–2.13 (m, 1H), 2.10–1.99 (m, 2H), 1.94–1.82 (m, 3H), 1.82–1.72 (m, 2H), 1.72–1.69 (m, 2H), 1.68 (d, *J* = 1.4 Hz, 3H), 1.67–1.61 (m, 2H), 1.60 (s, 3H), 1.50–1.23 (m, 9H), 1.08–1.00 (m, 1H), 1.05 (s, 3H), 0.99 (s, 3H), 0.89 (d, *J* = 6.5 Hz, 3H), 0.90–0.86 (m, 1H), 0.82 (s, 3H), 0.74 (s, 3H), 0.65 (s, 3H).

^{13}C-NMR: (101 MHz, $CDCl_3$) δ [ppm] = 148.7, 131.1, 125.4, 115.1, 79.1, 52.6, 51.1, 47.2, 44.4, 41.9, 39.5, 39.3, 37.3, 36.6, 36.3, 36.1, 34.1, 28.4, 28.3, 28.2, 28.0, 25.9, 25.1, 22.4, 21.5, 18.6, 18.5, 17.8, 15.8, 14.5.

IR: $\tilde{\nu}$ [cm^{-1}] = 3465 (br), 2964 (m), 2931 (s), 2867 (m), 1458 (m), 1371 (s), 1284 (w), 1248 (w), 1096 (m), 1050 (s), 1025 (w), 999 (m), 982 (w), 862

(w), 814 (w).

HRMS: (ESI-TOF); *m/z* calculated for $C_{30}H_{50}ONa^+$ $[M+Na]^+$: 449.3754, found: 449.3757.

Opt. act.: $[\alpha]_D^{20}$ = + 78.1 (*c* = 0.97, $CHCl_3$).

The spectroscopic data are in agreement with those reported in [9] and [10].

Synthesis of 4,4,14α-trimethyl-9β,19-cyclo-24-nor-5α-cholane-3β,23-diol (**23**)

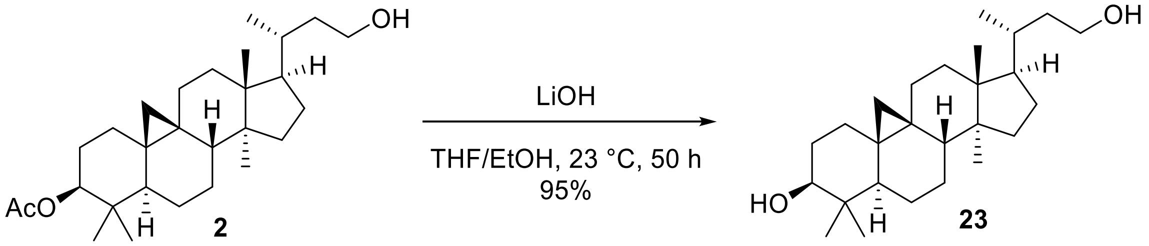

Acetate **2** (80.0 mg, 186 µmol, 1.0 eq.) was dissolved in THF/EtOH (3:2, *v*/*v*, 5.0 mL), LiOH (23.4 mg, 929 µmol, 5.0 eq.) was added at 23 °C, and the resulting solution was stirred for 50 h at 23 °C. The reaction mixture was then diluted with phosphate buffer (pH = 7, 5 mL), the organic phase was separated, and the aqueous phase was extracted with EtOAc (3 x 5 mL). The combined organic phases were dried over Na_2SO_4, filtered, and the solvent was removed under reduced pressure. Diol **23** was obtained as colorless crystals and used in the next reaction without further purification (68.6 mg, 176 µmol, 95%).

m.p.: 147–148 °C (EtOAc).

TLC: R_f = 0.26 (heptane/EtOAc = 7:3, *v*/*v*).

^{1}H-NMR: (400 MHz, $CDCl_3$) δ [ppm] = 3.73 (ddd, *J* = 10.4, 8.1, 4.7 Hz, 1H), 3.65 (dt, *J* = 10.4, 7.5 Hz, 1H), 3.28 (d, *J* = 11.2, 4.3 Hz, 1H), 2.06–1.86 (m, 2H), 1.81–1.70 (m, 2H), 1.65–1.47 (m, 8H), 1.36–1.30 (m, 4H), 1.29–1.21 (m, 5H), 1.18–1.01 (m, 2H), 0.97 (s, 3H), 0.96 (s, 3H), 0.90 (d,

J = 5.6 Hz, 3H), 0.90 (s, 3H), 0.81 (s, 3H), 0.79–0.73 (m, 1H), 0.55 (d, J = 4.2 Hz, 1H), 0.33 (d, J = 4.2 Hz, 1H).

^{13}C-NMR: (101 MHz, $CDCl_3$) δ [ppm] = 79.0, 61.2, 52.7, 49.0, 48.1, 47.2, 45.5, 40.6, 39.4, 35.7, 33.3, 33.0, 32.1, 30.5, 30.0, 28.4, 26.6, 26.2, 26.1, 25.6, 21.3, 20.1, 19.4, 18.6, 18.2, 14.1.

IR: $\tilde{\nu}$ [cm^{-1}] = 3325 (br), 2936 (s), 2866 (s), 1466 (m), 1456 (m), 1447 (m), 1375 (s), 1337 (w), 1098 (m), 1059 (s), 1051 (s), 1024 (s), 1005 (m), 991 (m), 970 (w).

HRMS: (EI-TOF); *m*/*z* calculated for $C_{26}H_{44}O_2^{\cdot+}$ [M]$^{\cdot+}$: 388.3336, found: 388.3340.

Opt. act.: $[\alpha]_D^{20}$ = + 44.0 (c = 0.25, $CHCl_3$).

Synthesis of 4,4,14α-trimethyl-24-nor-5α-chol-9-ene-3β,23-diol (**21**)

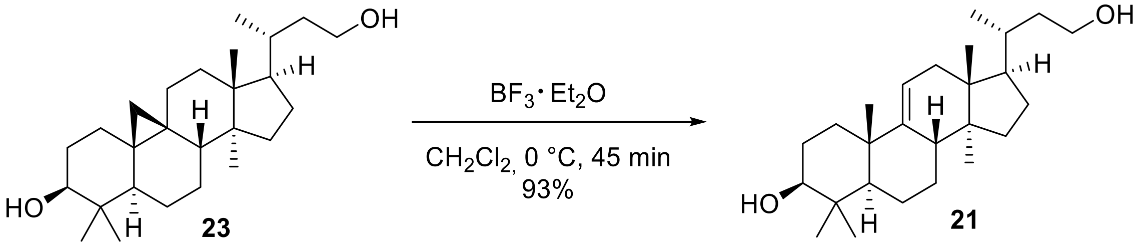

Diol **23** (66.0 mg, 170 µmol, 1.0 eq.) was dissolved in CH_2Cl_2 (2.5 mL), $BF_3\cdot Et_2O$ (254 µL, 1.7 mmol, 10 eq.) was added dropwise at 0 °C for 15 min, and the solution was stirred at 0 °C for 30 min. The reaction was then quenched with sat. aq. Na_2CO_3 (2 mL), the organic phase was separated, and the aqueous phase was extracted with CH_2Cl_2 (3 x 5 mL). The combined organic phases were dried over Na_2SO_4, filtered, and the solvent was removed under reduced pressure. The crude product was purified by column chromatography (SiO_2, heptane/EtOAc 8:2, *v*/*v*) to afford alkene **21** as colorless crystals (61.5 mg, 158 µmol, 93%).

m.p.: 197–198 °C (EtOAc).

TLC: R_f = 0.27 (heptane/EtOAc = 7:3, *v*/*v*).

^{1}H-NMR: (400 MHz, $CDCl_3$) δ [ppm] = 5.24–5.20 (m, 1H), 3.73 (ddd, J = 10.4, 8.1, 4.7 Hz, 1H), 3.65 (dt, J = 10.4, 7.5 Hz, 1H), 3.21 (dd, J = 11.5, 4.3 Hz, 1H), 2.21–2.13 (m, 1H), 2.12–2.04 (m, 1H), 1.98–1.86 (m, 2H), 1.82–1.57 (m, 8H), 1.57–1.41 (m, 4H), 1.40–1.21 (m, 5H), 1.04 (s, 3H), 0.99 (s, 3H), 0.92 (d, J = 6.3 Hz, 3H), 0.90–0.85 (m, 1H), 0.82 (s, 3H), 0.74 (s, 3H), 0.66 (s, 3H).

^{13}C-NMR: (101 MHz, $CDCl_3$) δ [ppm] = 148.7, 115.0, 79.1, 61.1, 52.6, 51.4, 47.2, 44.5, 41.9, 39.5, 39.5, 39.3, 37.3, 36.3, 34.0, 33.3, 28.4, 28.3, 28.2, 27.9, 22.4, 21.5, 18.7, 18.6, 15.8, 14.5.

IR: $\tilde{\nu}$ [cm^{-1}] = 3260 (br), 2959 (m), 2928 (s), 2872 (m), 2855 (m), 2830 (w), 1458 (m), 1441 (m), 1375 (m), 1368 (m), 1111 (w), 1101 (w), 1069 (s), 1061 (s), 1040 (m), 1020 (s), 1011 (m), 966 (s), 694 (m), 677 (m).

HRMS: (EI-TOF); m/z calculated for $C_{26}H_{44}O_2^{\cdot+}$ $[M]^{\cdot+}$: 388.3336, found: 388.3354.

Opt. act.: $[\alpha]_D^{20}$ = + 69.4 (c = 0.34, $CHCl_3$).

Synthesis of 4,4,14α-trimethyl-17,13-friedo-9β,19-cyclo-24-nor-5α-chol-12-ene-3β,12-diol (**24**)

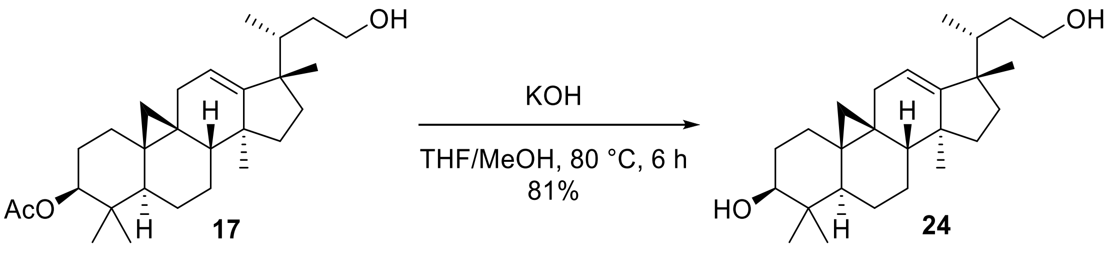

Alcohol **17** (650 mg, 1.52 mmol, 1.0 eq.) was dissolved in THF/MeOH (1:1, *v/v,* 32 mL), KOH (537 mg, 9.10 mmol, 6.0 eq.) was added at 23 °C, and the resulting solution was stirred for 6 h at 80 °C. The reaction mixture was then diluted with phosphate buffer (pH = 7, 20 mL), the organic phase was separated, and the aqueous phase was extracted with EtOAc (3 x 30 mL). The combined organic phases were dried over Na_2SO_4, filtered, and the solvent was removed under reduced pressure. The crude product was purified by column

chromatography (SiO_2, heptane/EtOAc 17:3, *v*/*v*) to afford diol **24** as colorless crystals (476 mg, 1.23 mmol, 81%).

**m.p.:**	146–148 °C (EtOAc).
**TLC:**	R_f = 0.23 (heptane/EtOAc = 7:3, *v*/*v*).
**^{1}H-NMR:**	(400 MHz, $CDCl_3$) δ [ppm] = 5.41 (dd, *J* = 7.7, 2.6 Hz, 1H), 3.79–3.72 (m, 1H), 3.66–3.58 (m, 1H), 3.31 (dd, *J* = 11.1, 4.3 Hz, 1H), 2.41 (dd, *J* = 15.4, 2.5 Hz, 1H), 1.91 (dtd, *J* = 13.3, 8.1, 2.5 Hz, 1H), 1.80–1.71 (m, 2H), 1.69–1.59 (m, 3H), 1.58–1.49 (m, 6H), 1.38 (dd, *J* = 12.3, 5.5 Hz, 1H), 1.34–1.28 (m, 2H), 1.27–1.24 (m, 1H), 1.23–1.14 (m, 3H), 1.13–1.07 (m, 1H), 1.01 (s, 3H), 1.00 (s, 3H), 0.87 (d, *J* = 6.9 Hz, 3H), 0.86 (s, 3H), 0.81 (s, 3H), 0.52 (d, *J* = 4.4 Hz, 1H), 0.17 (d, *J* = 4.4 Hz, 1H).
**^{13}C-NMR:**	(101 MHz, $CDCl_3$) δ [ppm] = 159.8, 115.4, 78.9, 62.4, 50.4, 49.5, 47.3, 46.7, 40.4, 38.0, 35.5, 35.2, 32.8, 31.7, 31.6, 30.2, 28.0, 26.4, 26.2, 25.9, 25.7, 22.1, 20.0, 17.6, 15.2, 14.2.
**IR:**	$\tilde{\nu}$ [cm^{-1}] = 3271 (br), 2965 (m), 2945 (s), 2914 (s), 2841 (m), 1458 (w), 1439 (w), 1371 (s), 1093 (m), 1053 (m), 1037 (s), 1024 (m), 1009 (m), 984 (m), 974 (s), 943 (w), 935 (w), 704 (w), 692 (w).
**HRMS:**	(ESI-TOF); *m*/*z* calculated for $C_{26}H_{43}O_2^+$ $[M+H]^+$: 387.3258, found: 387.3272.
**Opt. act.:**	$[\alpha]_D^{20}$ = + 65.0 (*c* = 0.12, $CHCl_3$).

Synthesis of 4,4,14α-trimethyl-3β-hydroxy-17,13-friedo-24-nor-5α,13β-chol-11-en-23-al (**22**)

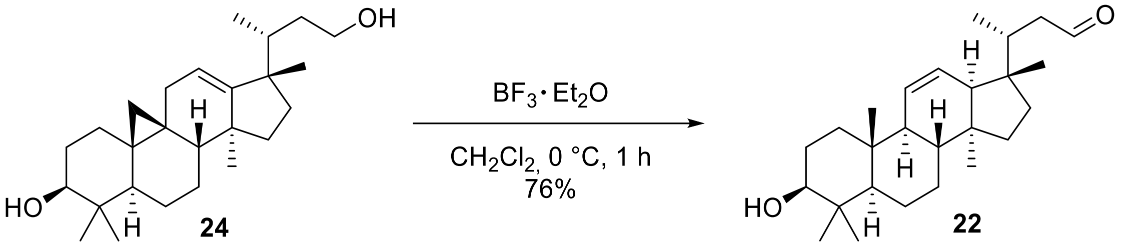

Diol **24** (30.0 mg, 77.6 µmol, 1.0 eq.) was dissolved in CH_2Cl_2 (1.2 mL), BF_3·Et_2O (104 µL, 776 µmol, 10 eq.) was added dropwise at 0 °C for 15 min, and the solution was stirred at 0 °C for 45 min. The reaction was then quenched with sat. aq. Na_2CO_3 (2 mL), the organic phase was separated and the aqueous phase was extracted with CH_2Cl_2 (3 x 5 mL). The combined organic phases were dried over Na_2SO_4, filtered, and the solvent was removed under reduced pressure to afford aldehyde **22** as a yellowish oil (28.8 mg, 59.2 µmol, 76%; yield corrected based on app. 20wt% impurity). Due to the high instability of the compound, the material was not further purified.

TLC: R_f = 0.36 (heptane/EtOAc = 4:1, *v*/*v*).

^{1}H-NMR: (400 MHz, $CDCl_3$) δ [ppm] = 9.76 (dd, *J* = 3.5, 1.1 Hz, 1H), 5.69 (dt, *J* = 10.3, 1.9 Hz, 1H), 5.52 (ddd, *J* = 10.4, 4.7, 2.4 Hz, 1H), 3.23 (dd, *J* = 11.6, 4.6 Hz, 1H), 2.56 (ddt, *J* = 15.9, 2.9, 1.0 Hz, 1H), 2.19 (ddd, *J* = 15.9, 10.7, 3.5 Hz, 1H), 2.05–1.95 (m, 1H), 1.90 (dt, *J* = 13.2, 3.6 Hz, 1H), 1.78–1.70 (m, 5H), 1.68–1.55 (m, 4H), 1.39–1.26 (m, 4H), 1.15–1.00 (m, 3H), 0.97 (s, 3H), 0.92 (dd, *J* = 6.7, 0.8 Hz, 3H), 0.85 (s, 3H), 0.80 (s, 3H), 0.80 (s, 3H), 0.76 (s, 3H).

^{13}C-NMR: (101 MHz, $CDCl_3$) δ [ppm] = 203.5, 128.5, 127.9, 79.1, 55.6, 54.0, 50.3, 47.4, 47.1, 44.1, 39.1, 38.2, 37.3, 37.1, 36.9, 36.3, 36.2, 28.1, 27.6, 27.1, 22.4, 22.4, 21.9, 16.3, 15.3, 15.3.

IR: $\tilde{\nu}$ [cm^{-1}] = 3429 (br), 2930 (s), 1719 (m), 1458 (m), 1375 (m), 1215 (m), 1085 (s), 1051 (s), 945 (m), 802 (m), 690 (w), 665 (m).

HRMS: (ESI-TOF); *m*/*z* calculated for $C_{26}H_{43}O_2^+$ $[M+H]^+$: 387.3258,

found: 387.3264.

Opt. act.: $[\alpha]_D^{20}$ = – 8.8 (c = 0.74, $CHCl_3$).

Synthesis of 4,4,14α-trimethyl-3β-hydroxy-17,13-friedo-9β,19-cyclo-24-nor-5α-chol-12-en-23-yl acetate (**25**) and 4,4,14α-trimethyl-17,13-friedo-9β,19-cyclo-24-nor-5α-chol-12-en-3β,23-yl diacetate (**29**)

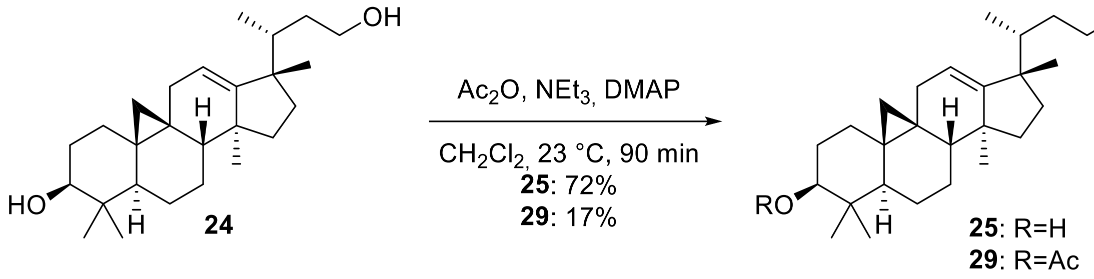

Diol **24** (455 mg, 1.18 mmol, 1.0 eq.) was dissolved in CH_2Cl_2 (50 mL) and triethylamine (243 µL, 2.35 mmol, 2.0 eq.) and 4-(dimethylamino)pyridine (7.2 mg, 59.0 µmol, 0.05 eq.) were added sequentially at 23 °C. Acetic anhydride (165 µL, 1.53 mmol, 1.3 eq.) was then added dropwise and the resulting solution was stirred for 90 min at 23 °C. The reaction was quenched with sat. aq. $NaHCO_3$ (20 mL), the organic phase was separated, and the aqueous phase was extracted with CH_2Cl_2 (3 x 50 mL). The combined organic phases were dried over Na_2SO_4, filtered, and the solvent was removed under reduced pressure. The crude product was purified by column chromatography (SiO_2, heptane/EtOAc 8:2, *v*/*v*) to afford desired acetate **25** as a colorless oil (364 mg, 84.9 µmol, 72%) and minor product **29** as colorless crystals (92.4 mg, 19.6 µmol, 17%).

acetate **25**

TLC: R_f = 0.37 (heptane/EtOAc = 7:3, *v*/*v*).

^{1}H-NMR: (400 MHz, $CDCl_3$) δ [ppm] = 5.41 (dd, J = 7.7, 2.6 Hz, 1H), 4.18 (ddd, J = 10.9, 8.0, 4.8 Hz, 1H), 4.02 (ddd, J = 10.8, 8.2, 7.2 Hz, 1H), 3.31 (d, J = 10.3 Hz, 1H), 2.41 (dd, J = 15.6, 2.6 Hz, 1H), 2.04 (s, 3H), 1.97 (dtd, J = 13.6, 8.1, 2.5 Hz, 1H), 1.80–1.71 (m, 2H), 1.66–1.47 (m, 8H), 1.40–

1.31 (m, 3H), 1.30–1.14 (m, 4H), 1.08 (td, *J* = 12.4, 2.8 Hz, 1H), 1.00 (s, 3H), 0.99 (s, 3H), 0.88 (d, *J* = 6.8 Hz, 3H), 0.86 (s, 3H), 0.81 (s, 3H), 0.51 (d, *J* = 4.4 Hz, 1H), 0.16 (d, *J* = 4.4 Hz, 1H).

^{13}C-NMR: (101 MHz, $CDCl_3$) δ [ppm] = 171.4, 159.6, 115.6, 78.9, 64.2, 50.2, 49.4, 47.3, 46.6, 40.4, 37.8, 35.6, 32.9, 31.7, 31.5, 31.0, 30.2, 28.0, 26.2, 26.0, 25.9, 25.7, 22.0, 21.2, 20.0, 17.7, 15.0, 14.2.

IR: $\tilde{\nu}$ [cm^{-1}] = 3478 (br), 2957 (m), 2940 (m), 2864 (w), 1728 (s), 1456 (w), 1373 (m), 1366 (m), 1248 (s), 1107 (w), 1043 (s), 1022 (s), 1010 (m), 991 (m), 608 (w), 488 (w).

HRMS: (ESI-TOF); *m/z* calculated for $C_{28}H_{44}O_3Na^+$ [M+Na]$^+$: 451.3183, found: 451.3191.

Opt. act.: $[\alpha]_D^{20}$ = + 30.8 (*c* = 0.23, $CHCl_3$).

diacetate **29**

m.p.: 119–121 °C (EtOAc).

TLC: R_f = 0.73 (heptane/EtOAc = 7:3, *v*/*v*),
R_f = 0.49 (CH_2Cl_2/MeOH = 500:1, *v*/*v*).

^{1}H-NMR: (400 MHz, $CDCl_3$) δ [ppm] = 5.40 (dd, *J* = 7.7, 2.6 Hz, 1H), 4.59 (dd, *J* = 11.2, 4.2 Hz, 1H), 4.18 (ddd, *J* = 10.7, 8.0, 4.8 Hz, 1H), 4.02 (dt, *J* = 10.7, 7.9 Hz, 1H), 2.42 (dd, *J* = 15.6, 2.6 Hz, 1H), 2.05 (s, 3H), 2.04 (s, 3H), 1.96 (dtd, *J* = 13.5, 8.0, 2.2 Hz, 1H), 1.80–1.73 (m, 2H), 1.69–1.58 (m, 4H), 1.53–1.47 (m, 3H), 1.40–1.35 (m, 2H), 1.34–1.26 (m, 3H), 1.22–1.14 (m, 2H), 1.13–1.08 (m, 1H), 1.00 (s, 3H), 0.89 (s, 3H), 0.87 (d, *J* = 7.0 Hz, 3H), 0.87 (s, 3H), 0.86 (s, 3H), 0.53 (d, *J* = 4.4 Hz, 1H), 0.18 (d, *J* = 4.4 Hz, 1H).

^{13}C-NMR: (101 MHz, $CDCl_3$) δ [ppm] = 171.4, 171.1, 159.6, 115.5, 80.7, 64.1, 50.2, 49.4, 47.4, 46.6, 39.4, 37.8, 35.6, 32.9, 31.7, 31.2, 31.0, 29.8, 28.0, 26.7, 26.0, 25.8, 25.7, 22.1, 21.5, 21.2, 19.8, 17.7, 15.4, 15.0.

IR: $\tilde{\nu}$ [cm^{-1}] = 2933 (m), 2867 (w), 1728 (s), 1460 (w), 1366 (m), 1281 (w), 1159 (s), 1173 (w), 1144 (w), 1100 (w), 1057 (w), 1030 (m), 1021 (m),

999 (w), 948 (w).

HRMS: (ESI-TOF); *m/z* calculated for $C_{30}H_{46}O_4Na^+$ $[M+Na]^+$: 493.3288, found: 493.3299.

Opt. act.: $[\alpha]_D^{20}$ = + 42.3 (*c* = 0.82, $CHCl_3$).

Synthesis of 4,4,17β-trimethy-9(10→19)abeo-24-nor-5α,13α-chola-8,14-dien-3β,23-yl diacetate (**30**)

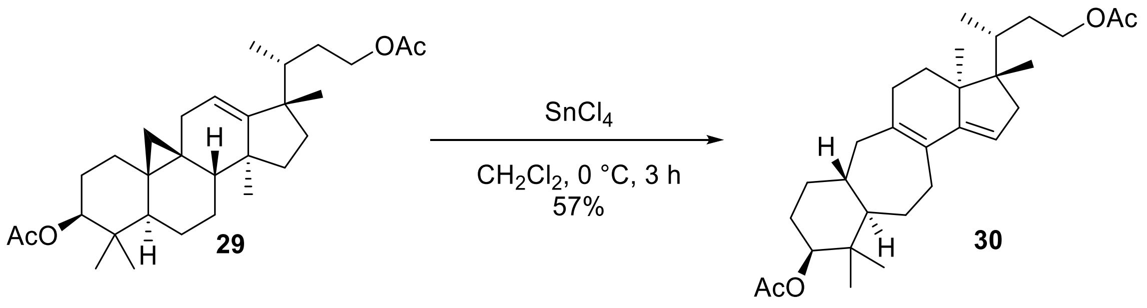

Diacetate **29** (19.7 mg, 41.9 µmol, 1.0 eq.) was dissolved in CH_2Cl_2 (630 µL), $SnCl_4$ (48.9 µL, 419 µmol, 10 eq.) was added dropwise at 0 °C for 15 min, and the resulting solution was stirred at 0 °C for 3 h. The reaction was then quenched with sat. aq. Na_2CO_3 (1 mL), the organic phase was separated, and the aqueous phase was extracted with CH_2Cl_2 (3 x 3 mL). The combined organic phases were dried over Na_2SO_4, filtered, and the solvent was removed under reduced pressure. The crude product was purified by column chromatography (SiO_2, CH_2Cl_2/MeOH 1000:1, *v/v*) to afford diene **30** as a colorless oil (11.3 mg, 24.0 µmol, 57%).

TLC: R_f = 0.54 (CH_2Cl_2/MeOH = 500:1, *v/v*).

^{1}H-NMR: (400 MHz, $CDCl_3$) δ [ppm] = 5.32–5.30 (m, 1H), 4.50 (dd, *J* = 11.7, 4.4 Hz, 1H), 4.17 (ddd, *J* = 12.0, 7.8, 4.5 Hz, 1H), 4.05 (ddd, *J* = 10.7, 8.5, 6.9 Hz, 1H), 2.44 (dd, *J* = 14.8, 7.4 Hz, 1H), 2.34–2.26 (m, 2H), 2.21 (dd, *J* = 13.9, 9.2 Hz, 1H), 2.08–2.06 (m, 1H), 2.05 (s, 3H), 2.04 (s, 3H), 2.02–1.98 (m, 1H), 1.97–1.85 (m, 4H), 1.79–1.71 (m, 2H), 1.69 (d, *J* = 14.3 Hz, 1H), 1.65–1.58 (m, 2H), 1.56–1.49 (m, 1H), 1.32–1.24 (m, 2H),

1.22–1.18 (m, 1H), 1.08–1.04 (m, 1H), 0.93–0.91 (m, 1H), 0.89 (d, J = 6.9 Hz, 3H), 0.89 (s, 3H), 0.84 (s, 3H), 0.80 (s, 3H), 0.79 (s, 3H).

^{13}C-NMR: (101 MHz, $CDCl_3$) δ [ppm] = 171.4, 171.0, 148.1, 135.7, 132.4, 115.8, 80.1, 63.7, 56.2, 50.6, 48.3, 45.5, 42.3, 38.7, 35.5, 34.7, 34.0, 31.7, 30.9, 29.9, 27.3, 27.3, 27.0, 25.4, 21.5, 21.2, 17.5, 16.0, 15.6, 14.9.

IR: $\tilde{\nu}$ [cm^{-1}] = 3435 (br), 2940 (m), 1731 (s), 1455 (w), 1366 (m), 1242 (s), 1026 (s), 971 (m), 902 (w), 801 (w), 752 (m), 666 (w), 492 (w), 444 (w).

HRMS: (ESI-TOF); *m/z* calculated for $C_{30}H_{46}O_4Na^+$ [M+Na]$^+$: 493.3288, found: 493.3305.

Opt. act.: $[\alpha]_D^{20}$ = + 13.1 (c = 0.63, $CHCl_3$).

Synthesis of 4,4,17β-trimethy-3β-hydroxy-24-nor-5α,13α-chola-8,14-dien-23-yl acetate (**28**)

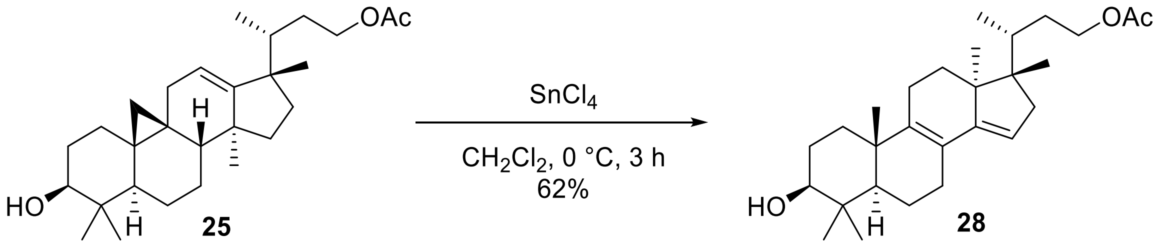

Alcohol **25** (50.0 mg, 117 µmol, 1.0 eq.) was dissolved in CH_2Cl_2 (1.7 mL), $SnCl_4$ (143 µL, 1.17 mmol, 10 eq.) was added dropwise at 0 °C for 15 min, and the resulting solution was stirred at 0 °C for 3 h. The reaction was then quenched with sat. aq. Na_2CO_3 (2 mL), the organic phase was separated, and the aqueous phase was extracted with CH_2Cl_2 (3 x 5 mL). The combined organic phases were dried over Na_2SO_4, filtered, and the solvent was removed under reduced pressure. The crude product was purified by column chromatography (SiO_2, heptane/EtOAc 9:1, *v*/*v*) to afford diene **28** as a colorless oil (31.1 mg, 72.6 µmol, 62%).

TLC: R_f = 0.32 (heptane/EtOAc = 7:3, *v*/*v*).

**^{1}H-NMR:**	(400 MHz, $CDCl_3$) δ [ppm] = 5.28 (t, J = 2.8 Hz, 1H), 4.16 (ddd, J = 10.9, 7.9, 4.6 Hz, 1H), 4.06 (ddd, J = 10.8, 8.4, 6.9 Hz, 1H), 3.24 (dd, J = 11.5, 4.7 Hz, 1H), 2.37 (dd, J = 16.7, 6.3 Hz, 1H), 2.28 (d, J = 16.2, 1H), 2.10–2.02 (m, 2H), 2.04 (s, 3H), 1.98–1.90 (m, 2H), 1.88–1.80 (m, 2H), 1.78–1.67 (m, 3H), 1.65–1.56 (m, 3H), 1.49 (dq, J = 12.5, 5.9 Hz, 1H), 1.31–1.25 (m, 2H), 1.24–1.19 (m, 1H), 1.13 (dd, J = 12.5, 1.9 Hz, 1H), 1.02 (s, 3H), 1.01 (s, 3H), 0.88 (d, J = 6.7 Hz, 3H), 0.83 (s, 3H), 0.83 (s, 3H), 0.77 (s, 3H).
**^{13}C-NMR:**	(101 MHz, $CDCl_3$) δ [ppm] = 171.4, 148.6, 142.2, 123.2, 116.3, 79.0, 63.7, 50.7, 50.3, 48.1, 45.5, 39.0, 38.0, 34.8, 34.6, 31.8, 30.1, 28.2, 27.8, 27.2, 23.0, 21.2, 19.3, 18.4, 17.2, 15.7, 15.7, 15.6.
**IR:**	$\tilde{\nu}$ [cm^{-1}] = 3422 (br), 2963 (m), 2938 (m), 1736 (s), 1721 (m), 1456 (w), 1366 (m), 1240 (s), 1094 (m), 1070 (w), 1038 (s), 1028 (s), 1001 (m), 974 (m), 935 (w), 669 (w), 449 (w).
**HRMS:**	(ESI-TOF); *m/z* calculated for $C_{28}H_{45}O_3^+$ $[M+H]^+$: 429.3363, found: 429.3379.
**Opt. act.:**	$[\alpha]_D^{20}$ = + 70.1 (c = 1.2, $CHCl_3$).

Synthesis of 4,4,17β-23-hydroxy-trimethyl-24-nor-5α,13α-chola-8,14-dien-3β-yl acetate (**32**)

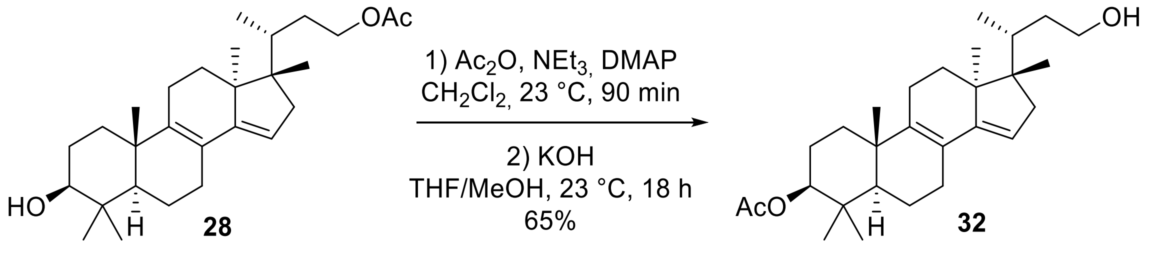

Alcohol **28** (30.0 mg, 70.0 µmol, 1.0 eq.) was dissolved in CH_2Cl_2 (2.1 mL) and triethylamine (21.7 µL, 210 µmol, 3.0 eq.) and 4-(dimethylamino)pyridine (4.0 µg, 3.5 µmol, 0.05 eq.) were added sequentially at 23 °C. Acetic anhydride (20.9 µL, 210 µmol, 3.0 eq.) was then added dropwise and the resulting solution was stirred for 90 min at 23 °C. The

reaction was quenched with sat. aq. $NaHCO_3$ (1 mL), the organic phase was separated, and the aqueous phase was extracted with CH_2Cl_2 (3 x 5 mL). The combined organic phases were dried over Na_2SO_4, filtered, and the solvent was removed under reduced pressure. The crude product (32.4 mg) was dissolved in THF/MeOH (2:1, *v*/*v*, 21 mL), KOH (4.90 mg, 82.6 µmol, 1.2 eq.) was added at 23 °C, and the resulting solution was stirred for 18 h at 23 °C. The reaction mixture was then diluted with phosphate buffer (pH = 7, 10 mL), the organic phase was separated, and the aqueous phase was extracted with EtOAc (3 x 20 mL). The combined organic phases were dried over Na_2SO_4, filtered, and the solvent was removed under reduced pressure. The crude product was purified by column chromatography (SiO_2, heptane/EtOAc 17:3, *v*/*v*) to afford diene **32** as a colorless oil (19.6 mg, 45.7 µmol, 65% over two steps).

TLC: R_f = 0.24 (*n*hexane/EtOAc = 3:1, *v*/*v*).

^{1}H-NMR: (400 MHz, $CDCl_3$) δ [ppm] = 5.28 (t, *J* = 2.8 Hz, 1H), 4.50 (dd, *J* = 11.6, 4.7 Hz, 1H), 3.74 (ddd, *J* = 10.3, 8.4, 4.5 Hz, 1H), 3.64 (dt, *J* = 10.3, 7.4 Hz, 1H), 2.38 (dd, *J* = 16.9, 7.1 Hz, 1H), 2.28 (d, *J* = 16.2 Hz, 1H), 2.08–2.07 (m, 1H), 2.05 (s, 3H), 1.98–1.92 (m, 2H), 1.87–1.70 (m, 4H), 1.70–1.59 (m, 3H), 1.55–1.47 (m, 1H), 1.36–1.24 (m, 4H), 1.22 (dd, *J* = 12.4, 1.7 Hz, 1H), 1.03 (s, 3H), 0.99–0.95 (m, 1H), 0.90 (s, 3H), 0.90 (s, 3H), 0.87 (d, *J* = 6.7 Hz, 3H), 0.85 (s, 3H), 0.77 (s, 3H).

^{13}C-NMR: (101 MHz, $CDCl_3$) δ [ppm] = 171.2, 148.6, 141.9, 123.2, 116.4, 81.0, 62.1, 50.8, 50.3, 48.1, 45.5, 37.9 (2C), 35.9, 34.5, 34.4, 30.1, 28.1, 27.1, 24.1, 22.9, 21.5, 19.4, 18.3, 17.3, 16.8, 15.8, 15.8.

HRMS: (ESI-TOF); *m*/*z* calculated for $C_{28}H_{45}O_3^+$ $[M+H]^+$: 429.3363, found: 429.3366.

The spectroscopic data are in agreement with those reported in [11].

Synthesis of 4,4,14α-trimethyl-3β-hydroxy-17,13-friedo-24-nor-5α-chola-9,12-dien-23-yl acetate (**27**)

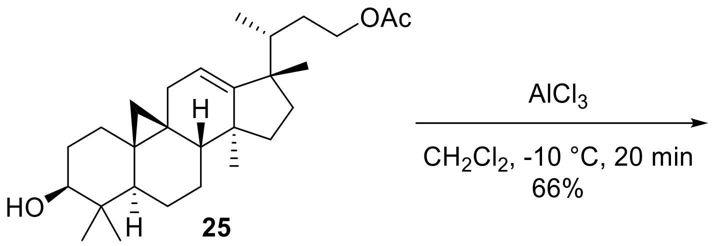

Alcohol **25** (30.0 mg, 70.0 µmol, 1.0 eq.) was dissolved in CH_2Cl_2 (1.0 mL), $AlCl_3$ (98.2 mg, 700 µmol, 10 eq.) was added in one portion at -10 °C, and the resulting solution was stirred at -10 °C for 20 min. The reaction was then quenched with sat. aq. Na_2CO_3 (1 mL), the organic phase was separated, and the aqueous phase was extracted with CH_2Cl_2 (3 x 3 mL). The combined organic phases were dried over Na_2SO_4, filtered, and the solvent was removed under reduced pressure. The crude product was purified by column chromatography (SiO_2 impregnated with $AgNO_3$ (20%), CH_2Cl_2/MeOH 450:1 → 100:1, *v*/*v*) to afford diene **27** as a colorless oil (19.7 mg, 46.0 µmol, 66%).

TLC: R_f = 0.44 (CH_2Cl_2/MeOH = 100:2, *v*/*v*).
impregnated with $AgNO_3$ (25%): R_f = 0.08 (CH_2Cl_2/MeOH = 100:2, *v*/*v*).

^{1}H-NMR: (400 MHz, $CDCl_3$) δ [ppm] = 5.68 (dd, *J* = 5.7, 3.0 Hz, 1H), 5.44 (d, *J* = 5.6 Hz, 1H), 4.18 (ddd, *J* = 10.8, 8.1, 4.9 Hz, 1H), 4.03 (dt, *J* = 10.8, 7.7 Hz, 1H), 3.25 (dd, *J* = 11.6, 4.4 Hz, 1H), 2.27 (ddd, *J* = 12.1, 7.0, 2.9 Hz, 1H), 2.04 (s, 3H), 2.03–1.95 (m, 2H), 1.83 (ddt, *J* = 12.3, 6.0, 2.8 Hz, 1H), 1.77–1.56 (m, 6H), 1.55–1.39 (m, 4H), 1.37–1.24 (m, 3H), 1.05 (s, 3H), 1.03 (s, 3H), 1.02 (s, 3H), 0.92 (dd, *J* = 11.6, 2.0 Hz, 1H), 0.88 (d, *J* = 6.8 Hz, 3H), 0.83 (s, 3H), 0.77 (s, 3H).

^{13}C-NMR: (101 MHz, $CDCl_3$) δ [ppm] = 171.4, 157.4, 149.4, 115.7, 114.4, 78.9, 64.1, 51.5, 49.0, 45.8, 45.1, 39.1, 39.1, 37.3, 37.0, 36.2, 32.8, 31.2, 28.8, 28.6, 27.9, 26.8, 24.2, 21.2, 20.7, 16.0, 15.6, 14.9.

IR: $\tilde{\nu}$ [cm^{-1}] = 3534 (w), 2963 (m), 2928 (m), 2862 (w), 1726 (s), 1458 (w), 1385 (w), 1368 (m), 1250 (s), 1233 (s), 1043 (s), 1032 (m), 1013 (w), 986

(w), 826 (m), 469 (w).

HRMS: (ESI-TOF); *m/z* calculated for $C_{28}H_{44}O_3Na^+$ $[M+Na]^+$: 451.3183, found: 451.3192.

Opt. act.: $[\alpha]_D^{20}$ = + 81.8 (*c* = 1.34, $CHCl_3$).

Synthesis of 4,4,17β-trimethy-l3β-hydroxy-24-nor-5α,13α-chola-8,14-diene-23-yl acetate (**28**) from diene **27**

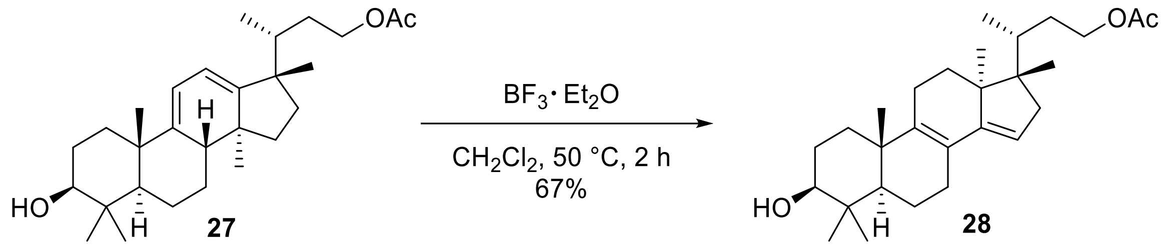

27 **28**

Alcohol **27** (14.0 mg, 32.7 µmol, 1.0 eq.) was dissolved in CH_2Cl_2 (500 µL), $BF_3{\cdot}Et_2O$ (43.6 µL, 327 µmol, 10 eq.) was added dropwise at 23 °C, and the solution was stirred at 50 °C for 2 h. The reaction was then quenched with sat. aq. Na_2CO_3 (1 mL), the organic phase was separated and the aqueous phase was extracted with CH_2Cl_2 (3 x 3 mL). The combined organic phases were dried over Na_2SO_4, filtered, and the solvent was removed under reduced pressure. The crude product was purified by column chromatography (SiO_2, heptane/EtOAc 9:1, *v*/*v*) to afford diene **28** as a colorless oil (9.4 mg, 22.0 µmol, 67%).

All analytical data of diene **28** was identical to the data from the previous procedure.

Synthesis of 4,4,14α-trimethyl-3β-hydroxy-17,13-friedo-24-nor-5α-chola-8,12-dien-23-yl acetate (**26**)

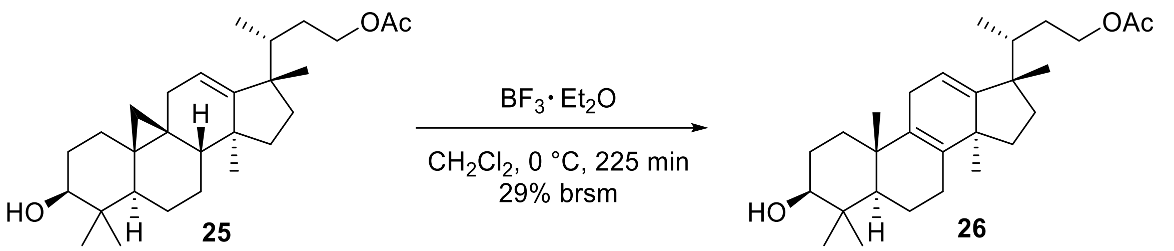

The reaction was carried out in parallel, employing multiple batches which were combined after quenching with sat. aq. Na_2CO_3.

Alcohol **25** (30.0 mg, 70.0 µmol, 1.0 eq.) was dissolved in CH_2Cl_2 (1.0 mL), $BF_3{\cdot}Et_2O$ (93.4 µL, 700 µmol, 10 eq.) was added dropwise at 0 °C, and the solution was stirred at 0 °C for 225 min. The reaction was then quenched with sat. aq. Na_2CO_3 (1 mL), the organic phase was separated, and the aqueous phase was extracted with CH_2Cl_2 (3 x 3 mL). The combined organic phases were dried over Na_2SO_4, filtered, and the solvent was removed under reduced pressure. The crude product was purified by column chromatography (SiO_2 impregnated with $AgNO_3$ (20%), CH_2Cl_2/MeOH 450:1 → 100:1, *v*/*v*) to afford diene **26** as a colorless oil (8.8 mg, 21.0 µmol, 29% brsm).

TLC: R_f = 0.45 (CH_2Cl_2/MeOH = 100:2, *v*/*v*),
impregnated with $AgNO_3$ (25%): R_f = 0.20 (CH_2Cl_2/MeOH = 100:2, *v*/*v*).

^{1}H-NMR: (400 MHz, $CDCl_3$) δ [ppm] = 5.36 (dd, *J* = 6.1, 2.1 Hz, 1H), 4.18 (ddd, *J* = 10.7, 8.2, 5.0 Hz, 1H), 4.02 (dt, *J* = 10.7, 7.7 Hz, 1H), 3.25 (dd, *J* = 11.5, 4.7 Hz, 1H), 2.57 (ddt, *J* = 20.5, 6.2, 1.8 Hz, 1H), 2.46 (dq, *J* = 20.4, 2.8 Hz, 1H), 2.13–2.07 (m, 2H), 2.04 (s, 3H), 2.04–1.98 (m, 1H), 1.80–1.65 (m, 4H), 1.62–1.44 (m, 5H), 1.33–1.17 (m, 4H), 1.06 (dd, *J* = 12.5, 2.0 Hz, 1H), 1.02 (s, 3H), 0.95 (s, 3H), 0.93 (s, 3H), 0.89 (s, 3H), 0.89 (d, *J* = 5.6 Hz, 3H), 0.82 (s, 3H).

^{13}C-NMR: (101 MHz, $CDCl_3$) δ [ppm] = 171.4, 155.9, 136.4, 135.2, 116.1, 79.1, 64.2, 51.1, 47.7, 47.5, 38.9, 38.9, 37.2, 35.1, 34.9, 33.7, 31.1, 28.2, 27.9, 27.1, 26.5, 26.1, 23.7, 21.2, 19.4, 16.8, 15.7, 15.6.

IR: $\tilde{\nu}$ [cm^{-1}] = 3441 (br), 2959 (m), 2941 (m), 2870 (w), 1736 (s), 1719 (m), 1456 (w), 1366 (m), 1242 (s), 1233 (s), 1088 (w), 1028 (s), 976 (w), 934 (w), 814 (w), 507 (w).

HRMS: (ESI-TOF); *m/z* calculated for $C_{28}H_{44}O_3Na^+$ $[M+Na]^+$: 451.3183, found: 451.3201.

Opt. act.: $[\alpha]_D^{20}$ = + 50.2 (c = 1.98, $CHCl_3$).

Synthesis of 4,4,17β-trimethy-l3β-hydroxy-24-nor-5α,13α-chola-8,14-diene-23-yl acetate (**28**) from diene **26**

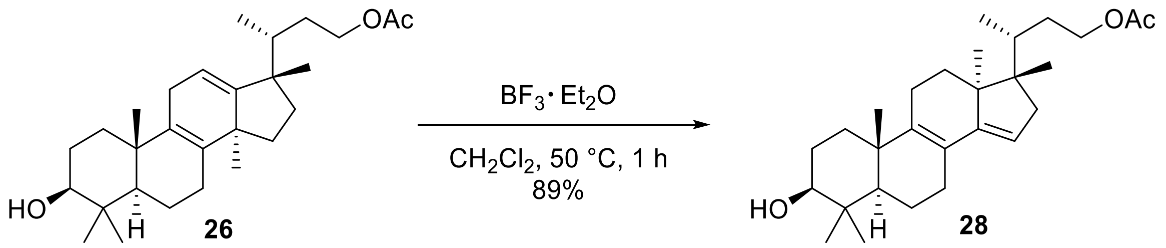

Alcohol **26** (15.6 mg, 36.4 µmol, 1.0 eq.) was dissolved in CH_2Cl_2 (550 µL), $BF_3{\cdot}Et_2O$ (48.5 µL, 364 µmol, 10 eq.) was added dropwise at 23 °C, and the solution was stirred at 50 °C for 1 h. The reaction was then quenched with sat. aq. Na_2CO_3 (1 mL), the organic phase was separated and the aqueous phase was extracted with CH_2Cl_2 (3 x 3 mL). The combined organic phases were dried over Na_2SO_4, filtered, and the solvent was removed under reduced pressure. The crude product was purified by column chromatography (SiO_2 impregnated with $AgNO_3$ (20%), CH_2Cl_2/MeOH 450:1, *v*/*v*) to afford diene **28** as a colorless oil (13.9 mg, 32.4 µmol, 89%).

All analytical data of diene **28** was identical to the data from the previous procedure.

Synthesis of 4,4,14α-trimethyl-17,13-friedo-24-nor-5α-chola-8,12-diene-3β,23-diol (**34**)

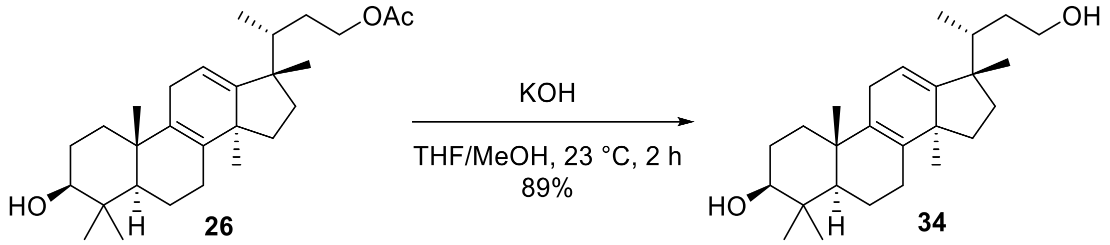

Diene **26** (86.0 mg, 201 µmol, 1.0 eq.) was dissolved in THF/MeOH (1:1, *v*/*v*, 4.4 mL), KOH (71.1 mg, 1.20 mmol, 6.0 eq.) was added at 23 °C, and the resulting solution was stirred for 2 h at 23 °C. The reaction mixture was then diluted with phosphate buffer (pH = 7, 3 mL), the organic phase was separated, and the aqueous phase was extracted with EtOAc (3 x 5 mL). The combined organic phases were dried over Na_2SO_4, filtered, and the solvent was removed under reduced pressure. The crude product was purified by column chromatography (SiO_2, heptane/EtOAc 8:2, *v*/*v*) to afford diol **34** as colorless crystals (69.2 mg, 179 µmol, 89%).

TLC: R_f = 0.22 (heptane/EtOAc = 7:3, *v*/*v*).

^{1}H-NMR: (400 MHz, $CDCl_3$) δ [ppm] = 5.37 (dd, *J* = 6.1, 2.1 Hz, 1H), 3.76 (ddd, *J* = 10.3, 8.3, 4.7 Hz, 1H), 3.62 (dt, *J* = 10.3, 7.6 Hz, 1H), 3.25 (dd, *J* = 11.5, 4.7 Hz, 1H), 2.58 (ddt, *J* = 20.5, 6.2, 1.8 Hz, 1H), 2.47 (dq, *J* = 20.4, 2.8 Hz, 1H), 2.13–2.07 (m, 2H), 1.97 (dtd, *J* = 13.2, 8.0, 2.7 Hz, 1H), 1.81–1.65 (m, 4H), 1.64–1.45 (m, 5H), 1.32–1.15 (m, 5H), 1.06 (dd, *J* = 12.5, 2.0 Hz, 1H), 1.02 (s, 3H), 0.96 (s, 3H), 0.94 (s, 3H), 0.90 (s, 3H), 0.88 (d, *J* = 6.8 Hz, 3H), 0.82 (s, 3H).

^{13}C-NMR: (101 MHz, $CDCl_3$) δ [ppm] = 156.1, 136.4, 135.3, 116.0, 79.1, 62.4, 51.1, 47.7, 47.6, 38.9, 38.6, 37.2, 35.3, 35.1, 35.0, 33.7, 28.2, 28.0, 27.2, 26.5, 26.1, 23.7, 19.4, 18.6, 15.8, 15.6.

IR: $\tilde{\nu}$ [cm^{-1}] = 3306 (br), 2959 (m), 2928 (m), 2868 (w), 1458 (w), 1369 (w), 1261 (w), 1094 (m), 1055 (m), 1039 (m), 1030 (s), 1018 (s), 1005 (s), 974 (w), 935 (w), 800 (m), 706 (w), 669 (w), 635 (w), 515 (w).

HRMS: (ESI-TOF); *m*/*z* calculated for $C_{26}H_{43}O_2^+$ $[M+H]^+$: 387.3258,

found: 387.3246.

Opt. act.: $[\alpha]_D^{20}$ = + 64.6 (c = 0.20, $CHCl_3$).

Synthesis of 4,4,14α-trimethyl-17,13-friedo-5α-chola-8,12,23-trien-3-one (**31**)

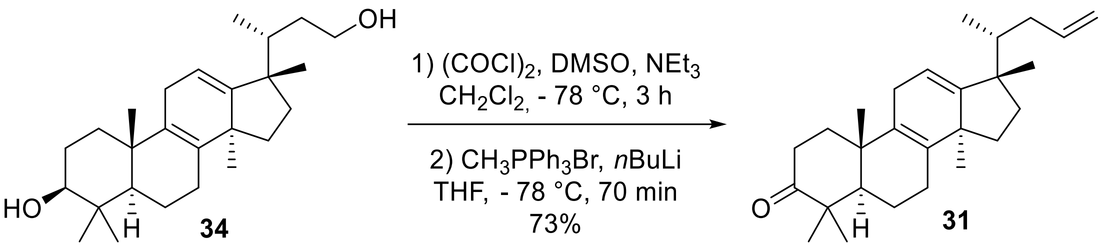

Oxalyl chloride (81.7 mL, 905 μmol, 10 eq.) was dissolved in CH_2Cl_2 (3.1 mL), DMSO (180 μL, 2.54 mmol, 28 eq.) was added at -78 °C, and the resulting solution was stirred for 20 min at -78 °C. Diol **34** (35.0 mg, 90.5 μmol, 1.0 eq.) in CH_2Cl_2 (1.0 mL) was then added dropwise and the resulting solution was stirred for 90 min at -78 °C. After this time, NEt_3 (450 μL) was added and the reaction solution was allowed to warm to 23 °C over 1 h. The reaction mixture was then diluted with heptane/toluene (3:1, *v*/*v*, 3 mL) and filtered through Celite®. The solvent of the filtrate was removed under reduced pressure and the so-obtained crude was dispersed in EtOAc (5 mL). The mixture was filtered again through Celite®, the solvent of the filtrate was removed under reduced pressure, and the crude was redissolved in heptane/toluene (3:1, 5 mL). The slightly cloudy solution was filtered a third time through Celite® and solvent of the filtrate was removed under reduced pressure to obtain crude aldehyde which was used in the next reaction without further purification.

Methyltriphenylphosphonium bromide (172.0 mg, 457 μmol, 5.0 eq.) was dissolved in THF (1.6 mL), *n*butyllithium (2.5 M in hexanes, 165 μL, 412 μmol, 4.5 eq.) was added at -78 °C, and the resulting solution was stirred for 40 min at 23 °C. Crude aldehyde (36.9 mg) in THF (1.0 mL) was then added dropwise and the resulting yellowish solution was stirred for 20 min at -78 °C. After this time, the reaction mixture was allowed to warm to 23 °C over 10 min and quenched with water (1 mL). The organic phase was separated and the aqueous phase was extracted with CH_2Cl_2 (3 x 3 mL). The combined organic phases were dried over Na_2SO_4, filtered, and the solvent was removed under reduced pressure. The crude

product was purified by column chromatography (SiO_2, heptane/EtOAc 19:1, *v*/*v*) to afford alkene **31** as a colorless oil (25.4 mg, 66.4 µmol, 73% over two steps).

TLC: R_f = 0.72 (heptane/EtOAc = 7:3, *v*/*v*).

^{1}H-NMR: (500 MHz, $CDCl_3$) δ [ppm] = 5.79 (dddd, *J* = 17.0, 10.1, 8.4, 5.7 Hz, 1H), 5.38 (dd, *J* = 6.2, 2.0 Hz, 1H), 5.03–4.94 (m, 2H), 2.65–2.42 (m, 5H), 2.19–2.12 (m, 2H), 1.99 (ddd, *J* = 13.1, 7.5, 3.7 Hz, 1H), 1.79 (td, *J* = 12.5, 6.9 Hz, 1H), 1.73–1.63 (m, 4H), 1.62–1.50 (m, 4H), 1.30 (dd, *J* = 12.3, 6.2 Hz, 1H), 1.12 (s, 3H), 1.08 (s, 3H), 1.06 (s, 3H), 0.98 (s, 3H), 0.92 (s, 3H), 0.85 (d, *J* = 6.8 Hz, 3H).

^{13}C-NMR: (126 MHz, $CDCl_3$) δ [ppm] = 218.1, 156.4, 139.2, 136.4, 134.9, 115.6, 115.2, 51.5, 47.8, 47.6, 47.4, 42.0, 37.0, 37.0, 35.4, 35.1, 34.6, 33.7, 27.4, 26.8, 26.5, 25.9, 23.5, 21.3, 19.9, 18.9, 15.4.

IR: $\tilde{\nu}$ [cm^{-1}] = 3374 (br), 2959 (m), 2934 (m), 1703 (s), 1458 (w), 1439 (w), 1261 (w), 1113 (m), 1040 (m), 1015 (m), 993 (m), 907 (m), 802 (m), 542 (m), 480 (w), 467 (w).

HRMS: (ESI-TOF); *m/z* calculated for $C_{27}H_{40}ONa^+$ $[M+Na]^+$: 403.2971, found: 403.2976.

Opt. act.: $[\alpha]_D^{20}$ = + 54.1 (*c* = 0.46, $CHCl_3$).

Synthesis of 4,4,14α-trimethyl-17,13-friedo-5α-chola-8,12,23-trien-3α-ol (**44**)

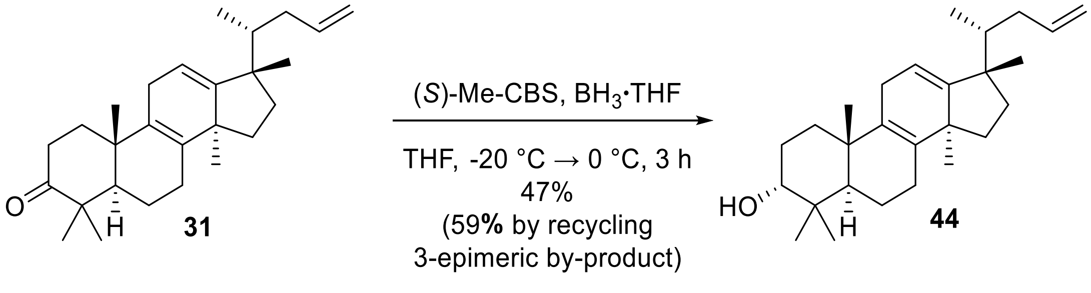

(*S*)-2-methyl-CBS-oxazaborolidine (17.9 mg, 61.5 µmol, 2.0 eq.) was dissolved in THF (600 µL), BH_3·THF (1 M, 55.3 µL, 55.3 µmol, 1.8 eq.) was slowly added dropwise at 0 °C

and the mixture was stirred at 0 °C for 15 min. Ketone **31** (11.7 mg, 30.7 µmol, 1.0 eq.) in THF (600 µL) was then added dropwise at -20 °C and the resulting solution was stirred for 1 h at this temperature and then warmed up to 0 °C over 2 h. The reaction was quenched by adding MeOH (1.0 mL) and all volatiles were removed under reduced pressure. The crude product was purified by column chromatography (SiO_2, heptane/EtOAc 19:1, *v*/*v*) to afford alcohol **44** (5.5 mg, 14.4 µmol, 47%) and the undesired alcohol **45** (3.4 mg, 8.9 µmol, 29%) as colourless oil. The undesired material was oxidized and reduced again to give another 12% of **44** leading to an overall yield of 59%.

α-epimer **44**

TLC: R_f = 0.61 (heptane/EtOAc = 7:3, *v*/*v*).

^{1}H-NMR: (600 MHz, $CDCl_3$) δ [ppm] = 5.79 (dddd, *J* = 17.1, 10.1, 8.4, 5.7 Hz, 1H), 5.36 (dd, *J* = 6.1, 2.0 Hz, 1H), 5.03–4.94 (m, 2H), 3.45 (t, *J* = 2.9 Hz, 1H), 2.58 (ddt, *J* = 20.5, 6.2, 1.8 Hz, 1H), 2.54–2.45 (m, 2H), 2.15–2.08 (m, 2H), 1.97 (ddd, *J* = 13.1, 7.5, 3.7 Hz, 1H), 1.81–1.60 (m, 6H), 1.57–1.44 (m, 6H), 1.30–1.24 (m, 1H), 0.99 (s, 3H), 0.96 (s, 3H), 0.94 (s, 3H), 0.90 (s, 3H), 0.88 (s, 3H), 0.85 (d, *J* = 6.8 Hz, 3H).

^{13}C-NMR: (151 MHz, $CDCl_3$) δ [ppm] = 156.3, 139.4, 136.6, 135.0, 115.9, 115.1, 76.1, 47.8, 47.5, 44.9, 42.0, 37.7, 37.1, 37.0, 35.0, 33.8, 29.7, 28.2, 27.4, 26.5, 25.9, 25.8, 23.7, 22.3, 19.2, 18.5, 15.4.

IR: $\tilde{\nu}$ [cm^{-1}] = 3404 (br), 2957 (s), 2928 (s), 2866 (m), 1707 (w), 1639 (w), 1456 (m), 1373 (m), 1088 (w), 1024 (s), 991 (m), 934 (w), 908 (s), 812 (w), 633 (w), 503 (w).

HRMS: (EI-TOF); *m*/*z* calculated for $C_{27}H_{42}O^{\cdot+}$ $[M]^{\cdot+}$: 382.3230, found: 382.3237.

Opt. act.: $[\alpha]_D^{20}$ = + 31.2 (*c* = 0.37, $CHCl_3$).

β-epimer **45**

TLC: R_f = 0.51 (heptane/EtOAc = 7:3, *v*/*v*).

^{1}H-NMR: (600 MHz, $CDCl_3$) δ [ppm] = 5.79 (dddd, *J* = 17.2, 10.2, 8.4, 5.6 Hz, 1H), 5.36 (dd, *J* = 6.2, 1.9 Hz, 1H), 5.04–4.94 (m, 2H), 3.26 (dd, *J* = 11.6,

4.5 Hz, 1H), 2.57 (ddt, J = 20.4, 6.2, 1.4 Hz, 1H), 2.51–2.44 (m, 2H), 2.14–2.08 (m, 2H), 1.81–1.67 (m, 6H), 1.66–1.55 (m, 3H), 1.53–1.45 (m, 3H), 1.29 (dd, J = 12.4, 6.7 Hz, 1H), 1.22 (dt, J = 12.5, 3.0 Hz, 1H), 1.02 (s, 3H), 0.96 (s, 3H), 0.94 (s, 3H), 0.90 (s, 3H), 0.85 (d, J = 6.8 Hz, 3H), 0.82 (s, 3H).

^{13}C-NMR: (151 MHz, $CDCl_3$) δ [ppm] = 156.3, 139.3, 136.4, 135.3, 115.9, 115.1, 79.2, 51.1, 47.7, 47.5, 42.0, 38.9, 37.2, 37.0, 35.1, 35.0, 33.8, 28.2, 28.0, 27.3, 26.5, 26.2, 23.7, 19.4, 18.6, 15.6, 15.4.

IR: $\tilde{\nu}$ [cm^{-1}] = 3418 (br), 2957 (s), 2924 (s), 2868 (m), 1709 (w), 1458 (m), 1373 (m), 1263 (w), 1088 (w), 1024 (s), 991 (m), 935 (w), 908 (s), 812 (w), 731 (w), 633 (w), 550 (w), 444 (w).

HRMS: (ESI-TOF); *m/z* calculated for $C_{27}H_{43}O^+$ $[M+H]^+$: 383.3308, found: 383.3304.

Opt. act.: $[\alpha]_D^{20}$ = + 51.5 (c = 0.11, $CHCl_3$).

Synthesis of 4,4,14α-trimethyl-17,13-friedo-5α-chola-8,12,23-trien-3-one (**31**) from undesired epimer **45**

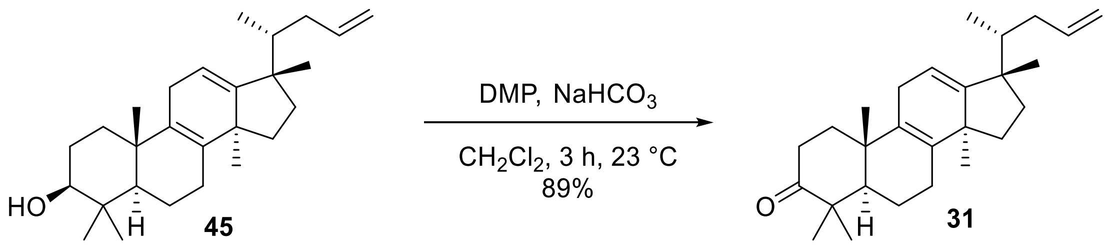

Alcohol **45** (3.4 mg, 8.9 µmol, 1.0 eq.) was dissolved in CH_2Cl_2 (200 µL), $NaHCO_3$ (2.6 mg, 31.1 µmol, 3.5 eq.) and Dess–Martin periodinane (95%, 5.2 mg, 11.6 µmol, 1.3 eq.) were added successively at 23 °C and the reaction mixture was stirred at this temperature for 2 h. The reaction was quenched with sat. aq. $Na_2S_2O_3$ (200 µL) and stirred for a further 30 min. The mixture was then extracted with CH_2Cl_2 (4 x 2 mL), the combined organic phases were washed with sat. aq. $NaHCO_3$ (10 mL), dried over Na_2SO_4, filtered, and the solvent was removed under reduced pressure. The crude product was purified by column

chromatography (SiO_2, heptane/EtOAc 19:1, *v*/*v*) to afford ketone **31** as a colorless oil (3.0 mg, 7.9 µmol, 89%).

All analytical data of ketone **31** was identical to the data from the previous procedure.

Synthesis of 25,26,27-Trinor-3α-hydroxy-17,13-friedolanostan-8,12-dien-23-one, "Sibiricanone" (**5**)

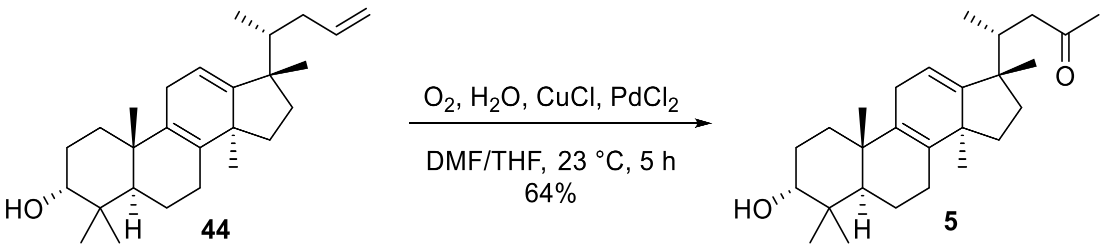

Triene **44** (3.9 mg, 10.2 µmol, 1.0 eq.) was dissolved in DMF/THF/H_2O (5:1:1, *v*/*v*/*v*, 350 µL), and $PdCl_2$ (0.4 mg, 2.0 µmol, 0.2 eq.) and CuCl (1.4 mg, 14.3 µmol, 1.4 eq.) were added successively. Oxygen was sparged through the stirred mixture at 23 °C for 5 h using a 22-gauge needle. After this time, the reaction mixture was diluted with CH_2Cl_2 (3 mL) and water (3 mL), the organic phase was separated, and the aqueous phase was extracted with CH_2Cl_2 (4 x 5 mL). The combined organic phases were dried over Na_2SO_4, filtered, and the solvent was removed under reduced pressure. The crude product was purified by column chromatography (SiO_2, heptane/EtOAc 8:2, *v*/*v*) to afford natural ketone **5** as a colourless oil (2.6 mg, 6.5 µmol, 64%).

TLC: R_f = 0.45 (heptane/EtOAc = 7:3, *v*/*v*).

^{1}H-NMR: (600 MHz, $CDCl_3$) δ [ppm] = 5.36 (dd, *J* = 6.1, 2.0 Hz, 1H), 3.42 (t, *J* = 2.5 Hz, 1H), 2.69 (dd, *J* = 15.2, 2.7 Hz, 1H), 2.56 (ddt, *J* = 20.4, 6.2, 1.7 Hz, 1H), 2.47 (ddt, *J* = 20.3, 4.0, 2.2 Hz, 1H), 2.17–2.14 (m, 1H), 2.11 (s, 3H), 2.11–2.05 (m, 3H), 1.97–1.91 (m, 1H), 1.75 (td, *J* = 12.4, 6.9 Hz, 1H), 1.64–1.58 (m, 2H), 1.56–1.53 (m, 2H), 1.52–1.41 (m, 4H), 1.26 (ddd, *J* = 12.4, 6.6, 1.2 Hz, 1H), 0.96 (s, 3H), 0.91 (s, 3H), 0.91 (s, 3H), 0.89 (s, 3H), 0.85 (s, 3H), 0.84 (d, *J* = 6.1 Hz, 3H).

**^{13}C-NMR:**	(151 MHz, $CDCl_3$) δ [ppm] = 209.7, 155.2, 136.3, 134.5, 116.3, 75.8, 47.6, 47.2, 46.8, 44.6, 38.0, 37.4, 36.8, 34.7, 33.8, 30.3, 29.4, 27.9, 26.6, 26.2, 25.6, 25.5, 23.4, 22.0, 18.9, 18.2, 15.9.
**IR:**	$\tilde{\nu}$ [cm^{-1}] = 3460 (br), 2953 (s), 2918 (s), 2868 (w), 2851 (w), 1709 (s), 1632 (w), 1456 (w), 1373 (w), 1364 (w), 1167 (w), 1065 (m), 1018 (m), 988 (m), 959 (m), 926 (m), 812 (w), 802 (w), 719 (w).
**HRMS:**	(ESI-TOF); *m/z* calculated for $C_{27}H_{42}O_2Na^+$ $[M+Na]^+$: 421.3077, found: 421.3075.
**Opt. act.:**	$[\alpha]_D^{20}$ = + 35.0 (*c* = 0.14, $CHCl_3$).

The spectroscopic data are in agreement with those reported in [12].

S6 X-ray crystallographic data.

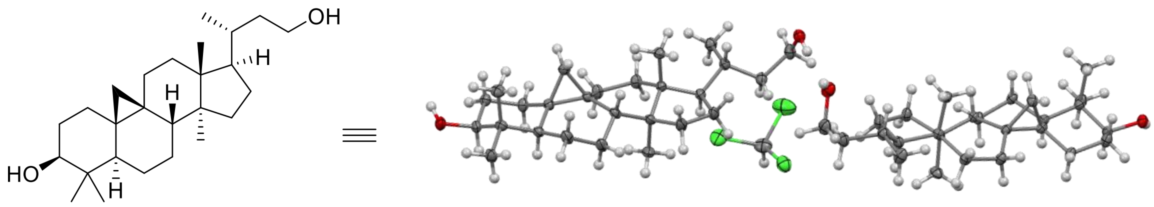

Table 1: Crystal data and structure refinement for **23**.

Identification code	2529341
Empirical formular	$C_{53}H_{88}Cl_3DO_4$
Formula weight	897.59
Temperature/K	100.0(2)
Crystal system	monoclinic
Space group	$P2_1$
a/Å	7.70460(10)
b/Å	11.15200(10)
c/Å	29.1031(3)
α/°	90
β/°	91.3380(10)
γ/°	90
Volume/Å^3	2499.91(5)
Z	2
D_{calc} g/cm^3	1.192
μ/mm^{-1}	1.981
F(000)	980.0
Crystal size/mm	0.70 x 0.57 x 0.27
Radiation	Cu K_α (λ = 1.54184 Å)
Θ_{max}/°	72.886
Index ranges	$-9 \le h \le 9$, $-13 \le k \le 13$, $-36 \le l \le 36$
Reflections collected	46112
Independent reflections	9631
Goodness-of-fit on F^2	1.069
Final R indexes [all data]	R_1 = 0.0376, wR_2 = 0.1007
Largest diff. peak/hole / e Å^{-3}	0.217/-0.394
Flack parameter	-0.001(5)

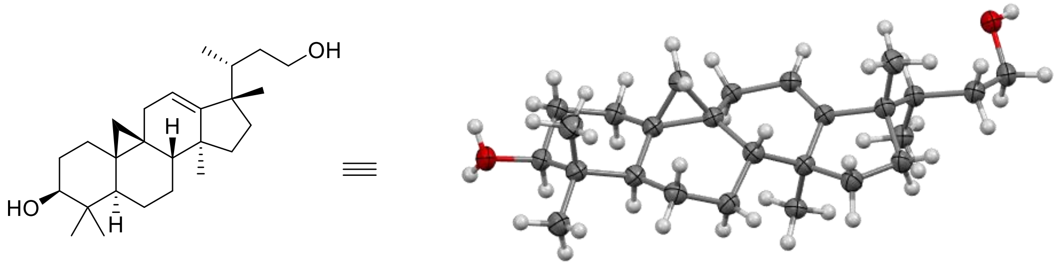

Table 2: Crystal data and structure refinement for **24**.

Identification code	2529339
Empirical Formular	$C_{26}H_{42}O_2$
Formula weight	386.59
Temperature/K	100.00(10)
Crystal system	orthorhombic
Space group	*I*222
a/Å	12.5209(3)
b/Å	24.8284(8)
c/Å	14.7661(5)
α/°	90
β/°	90
γ/°	90
Volume/Å^3	4590.4(2)
Z	8
D_{calc} g/cm^3	1.119
μ/mm^{-1}	0.518
F(000)	1712.0
Crystal size/mm	0.95 x 0.48 x 0.17
Radiation	Cu K_α (λ = 1.54184 Å)
Θ_{max}/°	72.271
Index ranges	$-15 \le h \le 15$, $-30 \le k \le 30$, $-18 \le l \le 18$
Reflections collected	20957
Independent reflections	4436
Goodness-of-fit on F^2	1.053
Final R indexes [all data]	R_1 = 0.0501, wR_2 = 0.1300
Largest diff. peak/hole / e Å^{-3}	0.247/-0.140
Flack parameter	0.16(15)

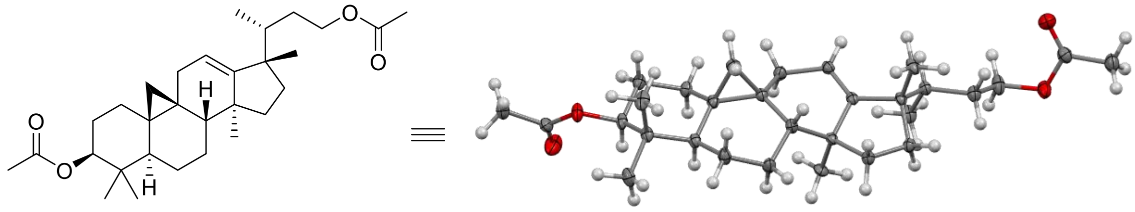

Table 3: Crystal data and structure refinement for **29**.

Identification code	2529340
Empirical Formular	$C_{30}H_{46}O_4$
Formula weight	470.67
Temperature/K	100.00(10)
Crystal system	orthorhombic
Space group	$P2_12_12_1$
a/Å	11.40720(10)
b/Å	11.97880(10)
c/Å	19.6655(3)
α/°	90
β/°	90
γ/°	90
Volume/Å^3	2687.18(5)
Z	4
D_{calc} g/cm^3	1.163
μ/mm^{-1}	0.587
F(000)	1032.0
Crystal size/mm	0.75 x 0.54 x 0.10
Radiation	Cu K_α (λ = 1.54184 Å)
Θ_{max}/°	72.660
Index ranges	$-14 \leq h \leq 14$, $-14 \leq k \leq 14$, $-24 \leq l \leq 24$
Reflections collected	25670
Independent reflections	5207
Goodness-of-fit on F^2	1.048
Final R indexes [all data]	R_1 = 0.0371, wR_2 = 0.0965
Largest diff. peak/hole / e Å^{-3}	0.299/-0.208
Flack parameter	0.03(7)

S7 Optimization of the furan formation to 19.

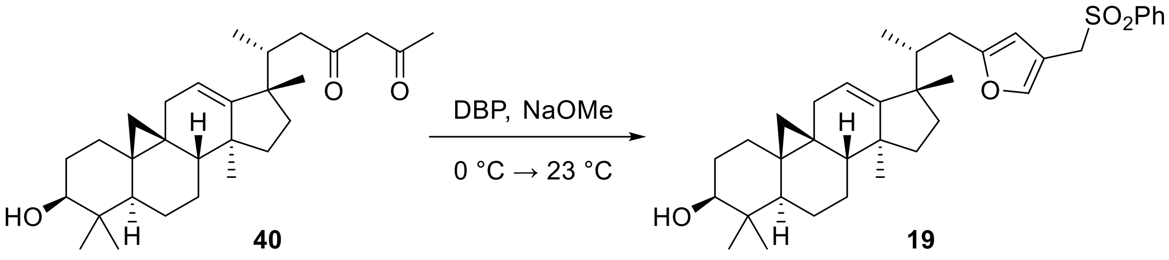

Scale	solvent	1st portion NaOMe	2nd protion NaOMe[a]	DBP	yield
15 mg	MeOH	1.2 eq.	1.4 eq. (after 90 min)	(*E*)-DBP (1.0 eq.)	29%
15 mg	MeOH/THF 4:1 (*v*/*v*)	1.2 eq.	1.4 eq. (after 90 min)	(*E*)-DBP (1.0 eq.)	42%
15 mg	MeOH/CH_2Cl_2 4:1 (*v*/*v*)	1.2 eq.	1.4 eq. (after 90 min)	(*E*)-DBP (1.0 eq.)	34%
15 mg	MeOH/DMF 4:1 (*v*/*v*)	1.2 eq.	1.4 eq. (after 90 min)	(*E*)-DBP (1.0 eq.)	27%
15 mg	MeOH/THF 4:1 (*v*/*v*)	2.2 eq.	1.4 eq. (after 60 min)	(*E*)-DBP (2.0 eq.)	36%
15 mg	MeOH/THF 4:1 (*v*/*v*)	1.2 eq.	1.4 eq. (after 90 min)	(*Z*)-DBP (1.0 eq.)	41%
40 mg	MeOH/THF 4:1 (*v*/*v*)	1.2 eq.	1.4 eq. (after 120 min)	(*E*)-DBP (1.0 eq.)	40%
80 mg	MeOH/THF 4:1 (*v*/*v*)	1.2 eq.	1.4 eq. (after 180 min)	(*E*)-DBP (1.0 eq.)	35%

[a] NaOMe was added when the starting material disappeared on the TLC.

S8 Optimization of the reduction to α-alcohol in position 3.

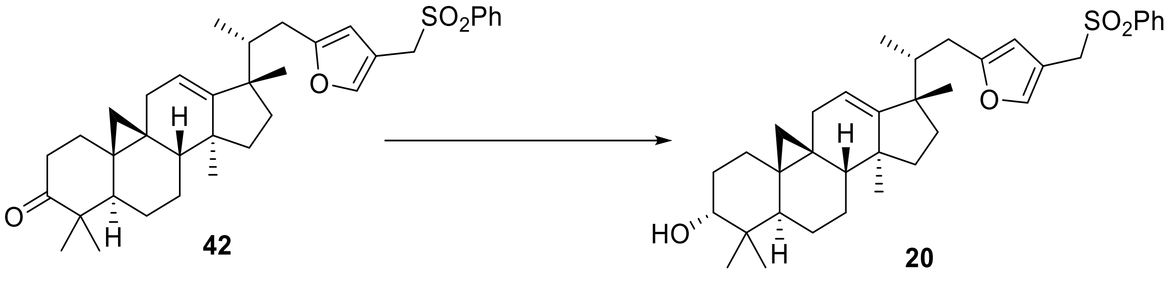

Reducing agent	temperature	Reaction time	yield α-epimer	yield β-epimer
L-selectride	-78°C	1 h	28%	47%
(*S*)-Me-CBS, BH_3·THF	0 °C	20 min	63%	12%

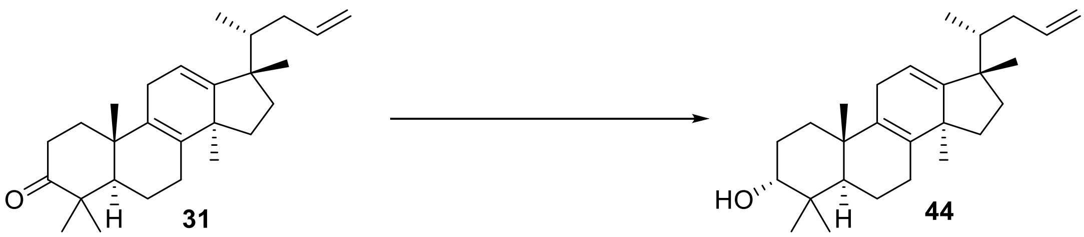

Reducing agent	temperature	Reaction time	yield α-epimer	yield β-epimer
L-selectride	-78°C	1 h	17%	44%
(*S*)-Me-CBS, BH_3·THF	0 °C	20 min	34%	9%
(*S*)-Me-CBS, BH_3·THF	-20 °C → 0 °C	3 h	47%	29%

S9 Cyclopropane Opening Experiments on Acetate 25.

Table 4 summarizes the experiments on opening the cyclopropane ring with various Lewis acids. Initially, $BF_3 \cdot Et_2O$ was used as the Lewis acid, which led to the parkeol system. However, with acetate **25**, a mixture of different prodcuts was observed. Therefore, attempts were made to selectively obtain these products using different Lewis acids. It was observed that the 17,13-friedoparkeol system could be selectively obtained with $AlCl_3$ and the 17,14-friedolanostane system could be obtained with $SnCl_4$. Transition metals showed no reactivity. The largest amount of 17,13-friedolanostane was obtained with $BF_3 \cdot Et_2O$.

Table 4: Cyclopropane Ring Opening Experiments with different Lewis Acids using 20 mg of Acetate **25** and CH_2Cl_2 as solvent.

conditions	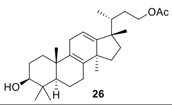 26	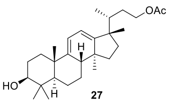 27	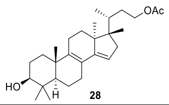 28	unknown by-products	starting material **25**
$BF_3 \cdot Et_2O$, 4 h, 0 °C	0.66	1.00	0.21	0.51	0.11
BCl_3, 30 min, -78 °C			decomposition		
BBr_3, 30 min, -78 °C			decomposition		
$AlCl_3$, 20 min, -10°C	0.21	1.00	–	0.11	–
$SnCl_4$, 3 h, 0 °C	–	–	1.00	0.38	–
$TiCl_4$, 1 h, -78 °C	–	1.00	–	1.93	–
$SiCl_4$, 1 h, -78 °C	–	0.11	–	0.14	1.00
Tris(pentafluoro-phenyl)boran, 2 h, 0 °C	–	0.07	–	0.06	1.00
$Sc(OTf)_3$, 4 h, 30 °C	–	0.10	–	0.17	1.00
Et_2AlCl, 2 h, 0 °C	–	–	–	–	1.00
$CuCl_2$	–	–	–	–	1.00
$Cu(OTf)_2$	–	–	–	–	1.00
$Zn(OTf)_2$	–	–	–	–	1.00

Table 5 summarizes the product distribution at different reaction times. A reaction time of 225 minutes was identified as ideal, as it results in a favorable ratio between conversion of the starting material and the formation of by-products.

Table 5: Cyclopropane Ring Opening Experiments with different reaction times using 30 mg of Acetate **25**, CH_2Cl_2 as solvent, and $BF_3{\cdot}Et_2O$ as Lewis acid.

Reaction time	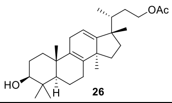 26	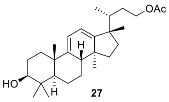 27	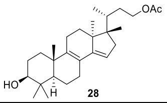 28	unknown by-products	starting material **25**
45 min	0.92	1.00	–	0.41	1.79
75 min	0.82	1.00	–	0.42	1.64
105 min	0.93	1.00	–	0.46	1.17
135 min	0.87	1.00	–	0.43	0.80
165 min	0.79	1.00	–	0.42	0.50
195 min	0.86	1.00	0.12	0.46	0.40
225 min	0.80	1.00	0.11	0.45	0.32
240 min	0.66	1.00	0.21	0.51	0.11

The investigation of the reaction temperature, summarized in Table 6, shows that the formation of the 17,13-friedolanostane system is most favored at a temperature of 0 °C. At lower temperatures, the 17,13-friedoparkeol system is formed more selectively, and at higher temperatures, the 17,14-friedolanostane system is formed.

Table 6: Cyclopropane Ring Opening Experiments with different temperatures using 20 mg of Acetate **25**, CH_2Cl_2 as solvent, and $BF_3{\cdot}Et_2O$ as Lewis acid.

conditions	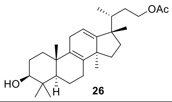 26	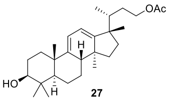 27	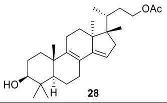 28	unknown by-products	starting material **25**
4 h, -30 °C	–	0.09	–	–	1.00
5 h, -10 °C	0.38	1.00	–	0.26	2.22
225 min, 0 °C	0.80	1.00	0.11	0.45	0.32
105 min, 10 °C	0.67	1.00	0.18	0.34	–
2 h, 26 °C	0.92	1.00	1.88	0.54	–

Table 7 shows that increasing the batch size leads to a lower conversion of starting material and greater formation of by-products. Therefore, several 30 mg batches were prepared for accumulation of products.

Table 7: Cyclopropane Ring Opening Experiments with different batch sizes using CH_2Cl_2 as solvent, and $BF_3 \cdot Et_2O$ as Lewis acid.

conditions	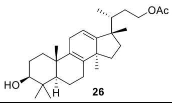 26	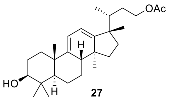 27	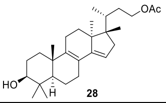 28	unknown by-prod-ucts	starting material **25**
30 mg, 4 h	0.66	1.00	0.21	0.51	0.11
50 mg, 4 h	0.71	1.00	–	0.40	0.55
200 mg, 4 h	0.78	1.00	0.14	1.12	0.33

The substance ratios in the tables were determined using ^{1}H-NMR by comparing the integrals of the characteristic vinylic protons of the substances. An example spectrum is shown in Figure 1.

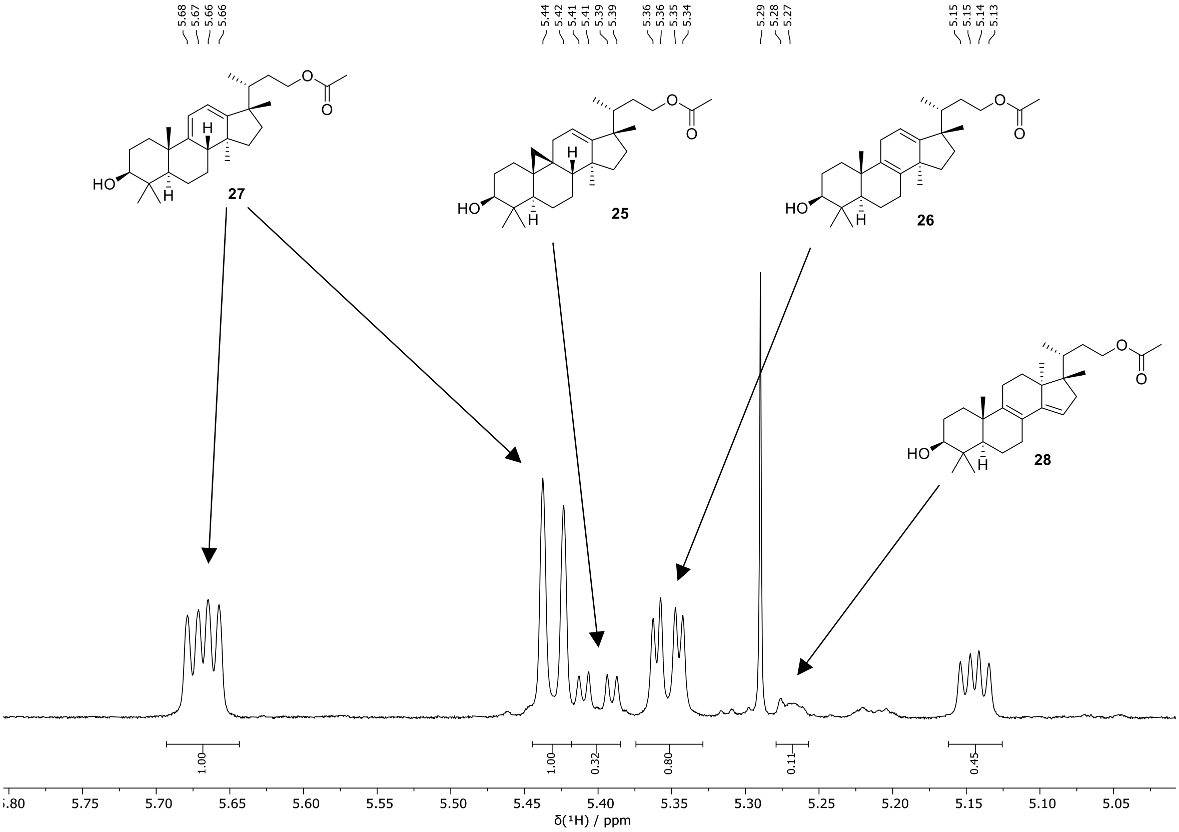

Figure 1: ^{1}H-NMR (400 MHz) spectrum in the range between 5.0–5.8 ppm of the crude product from cyclopropane ring opening experiment with $BF_3 \cdot Et_2O$ after 225 min.

S10 DFT calculations.

To investigate the reaction of **25** forming **26**, **27** and **28**, respectively, the pristine molecules as well as Lewis acid adducts were investigated. In a first step, the global minima were determined using the GOAT program (global geometry optimization and ensemble generator) implemented in ORCA in combination with GFN2-xTB.[13,14] Subsequently, DFT calculations were performed to further optimize the global minima using the ORCA program (version 6.0.1) with the libXC and libint2 libraries at B3LYP/def2-TZVP level. To take solvent effects into account, the conductor-like polarizable continuum model (C-PCM) was used as implicit solvation model. The calculated reaction Gibbs energies are shown in Figure 2 and summarized in Table 8.

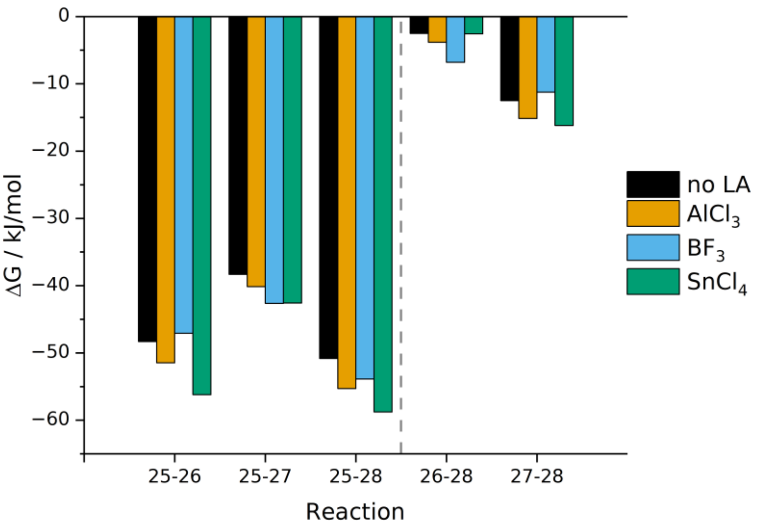

Figure 2: Calculated reaction Gibbs energies (DFT) with pristine molecules as well as Lewis acid adducts.

Table 8: Calculated reaction Gibbs energies with pristine molecules as well as Lewis acid adducts.

Reaction	Reaction Gibbs energy / kJ/mol			
	No Lewis acid	$AlCl_3$	BF_3	$SnCl_4$
**25-26**	-48.32	-51.47	-47.10	-56.21
**25-27**	-38.33	-40.16	-42.65	-42.60
**25-28**	-50.85	-55.31	-53.89	-58.79
**26-28**	-2.53	-3.84	-6.80	-2.57
**27-28**	-12.51	-15.15	-11.25	-16.19

In addition, DLPNO-CCSD(T)/def2-TZVP calculations with C-PCM were performed to obtain more accurate electronic energies. By adding the Gibbs energies minus the DFT electronic energies G-E(el,DFT), the final Gibbs energies were computed (see Figure 3 and Table 9).

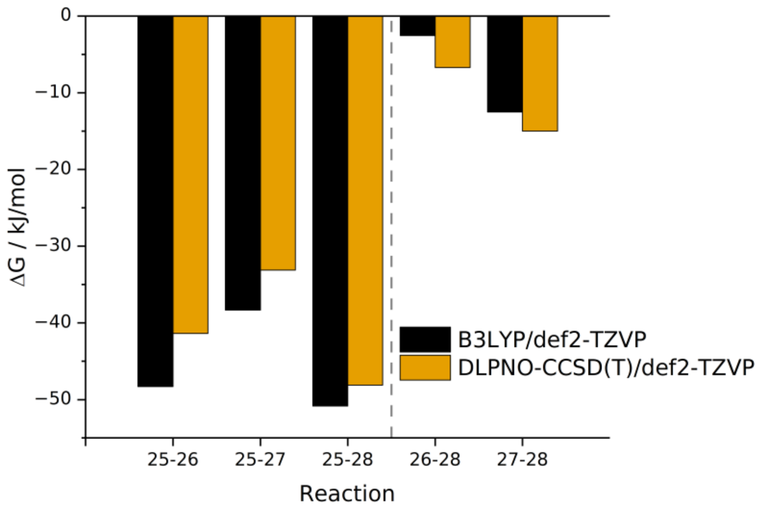

Figure 3: Comparison of reaction Gibbs energies.

Table 9: Comparison of reaction Gibbs energies.

Reaction	Reaction Gibbs energy / kJ/mol	
	B3LYP/def2-TZVP	DLPNO-CCSD(T)/def2-TZVP
**25-26**	-48.32	-41.38
**25-27**	-38.33	-33.11
**25-28**	-50.85	-48.11
**26-28**	-2.53	-6.72
**27-28**	-12.51	-15.00

To evaluate the formation of the boat conformation of the A ring, the molecules chair-**I** and boat-**I** were investigated. BF_3 was chosen as Lewis acid. In this case, chair-**I** is identical with the global minimum of the Lewis acid adduct of **25**. A comparison with the boat conformer of this adduct revealed difference of 15.33 kJ/mol (DFT: 18.30 kJ/mol). Furthermore, this conformational change was investigated using pristine molecule **25**. In the absence of a Lewis acid, the energy difference was calculated to be 20.48 kJ/mol (DFT: 23.70 kJ/mol).

S11 NMR Comparisons.

Table 10: NMR comparison of natural Fortunefuroic Acid I (**4**) and synthetic material.[6]

No.	^{1}H-NMR		^{13}C-NMR	
	natural	synthetic	natural	synthetic
1	1.98 br dd (11.9, 8.5)<br>1.67 ddd (11.9, 3.3, 3.3)	1.99 br dd (12.3, 9.1)<br>1.67 ddd (10.2, 3.1, 3.1)	28.3	28.3
2	1.96 br ddd (9.7, 3.3, 3.2)<br>1.07 br dd (9.7, 8.5)	1.96 br ddd (10.1, 2.8, 2.8)<br>1.08 br dd (9.2, 7.8)	26.3	26.3
3	3.50 dd (3.2, 3.2)	3.50 dd (2.7, 2.7)	77.1	77.1
4	–	–	39.2	39.3
5	1.80 dd (12.1, 3.5)	1.81 dd (11.5, 3.5)	41.1	41.1
6	1.47 dt (12.9, 3.5)<br>0.84 m	1.47 dt (12.9, 3.6)<br>0.84 m	19.7	19.8
7	1.16 qd (12.6, 2.8)<br>1.55 br d (12.6, 6.4)	1.16 qd (12.9, 2.6)<br>1.55 br dd (11.3, 5.5)	25.5	25.5
8	1.41 dd (12.6, 6.4)	1.41 dd (12.3, 6.0)	50.2	50.2
9	–	–	28.3*	21.7
10	–	–	26.8	26.8
11	1.54 dd (15.0, 8.0)<br>2.45 dd (15.0, 2.3)	1.54 dd (13.4, 8.2)<br>2.45 dd (15.7, 2.6)	31.4	31.4
12	5.46 dd (8.0, 2.3)	5.47 dd (7.7, 2.6)	115.8	115.8
13	–	–	159.3	159.3
14	–	–	46.5	46.5
15	1.52 m<br>1.55 m	1.52 m<br>1.55 m	37.6	37.6
16	1.81 br dd (13.0, 3.6)<br>1.23 ddd (13.0, 6.7, 5.8)	1.81 br dd (12.5, 3.4)<br>1.23 ddd (11.4, 6.2, 5.4)	32.9	32.9
17	–	–	49.1	49.1

18	1.08 s	1.08 s	25.6	25.6
19	0.49 d (4.2)<br>0.20 d (4.2)	0.50 d (4.3)<br>0.20 d (4.3)	27.8	27.8
20	1.97 m	1.97 m	38.1	38.2
21	0.83 d (6.8)	0.84 d (6.7)	14.9	14.9
22	2.97 dd (14.2, 1.6)<br>2.24 dd (14.2, 10.8)	2.97 dd (14.9, 2.9)<br>2.25 dd (14.8, 11.3)	30.8	30.8
23	–	–	158.4	158.5
24	6.36 s	6.36 s	105.6	105.7
25	–	–	118.8	118.3
26	–	–	167.1	164.5
27	7.97 s	7.96 s	147.4	147.2
28	0.99 s	1.00 s	26.0	26.1
29	0.89 s	0.90 s	21.4	21.4
30	0.91 s	0.91 s	17.7	17.8

All chemical shifts are given in ppm. The coupling constants are in parentheses and are given in Hz. The spectra were measured in $CDCl_3$ and referenced to the residual solvent signal at δ_H = 7.27 ppm and δ_C = 77.00 ppm. The synthetic material was measured at a concentration of 1.8 mg/mL at 298 K. ^{1}H-NMR spectra were recorded at 600 MHz. ^{13}C-NMR spectra were recorded at 151 MHz.

*Suspected error since the peak was assigned twice and another unassigned peak can be seen at 21.67 in the copy of the ^{13}C-NMR spectrum.[6]

Table 11: NMR comparison of natural alcohol **2** and synthetic material.[5]

No.	^{1}H-NMR		^{13}C-NMR	
	natural	synthetic	natural	synthetic
1	1.61 m<br>1.52 m	1.61 m<br>1.51 m	31.6	31.6
2	1.77 m<br>1.62 m	1.78 m<br>1.62 m	26.8	26.8
3	4.57 brdd (10.1, 4.7)	4.56 brdd (10.8, 5.0)	80.7	80.7
4	–	–	39.4	39.4
5	1,39 dd (12.5, 4.6)	1.39 dd (12.4, 4.4)	47.2	47.1
6	1.58 m<br>0.80 qd (12.5, 2.6)	1.57 m<br>0.79 qd (12.6, 2.6)	20.9	20.9
7	1.33 m<br>1.09 m	1.33 m<br>1.07 m	25.8	25.8
8	1.53 m	1.52 m	47.8	47.8
9	–	–	20.1	20.1
10	–	–	26.0	25.9
11	1.99 m<br>1.13 m	1.99 m<br>1.14 m	26.5	26.4
12	1.63 m	1.63 m	32.9	32.8
13	–	–	45.4	45.3
14	–	–	48.9	48.8
15	1.31 m	1.30 m	35.5	35.5
16	1.93 m<br>1.31 m	1.93 m<br>1.30 m	28.3	28.2
17	1.60 m	1.59 m	52.6	52.6
18	0.98 s	0.97 s	17.9	17.9
19	0.58 d (4.3)<br>0.34 d (4.3)	0.58 d (4.3)<br>0.34 d (4.3)	29.7	29.7

20	1.53 m	1.54 m	33.1	33.1
21	0.91 d (6.4)	0.90 d (6.3)	18.5	18.5
22	1.76 m<br>1.26 m	1.75 m<br>1.26 m	39.3	39.3
23	3.73 ddd (10.4, 8.1, 4.7)<br>3.66 dt (10.4, 7.5)	3.73 ddd (10.4, 8.1, 4.7)<br>3.65 dt (10.4, 7.5)	61.0	61.0
24	–	–	–	–
25	–	–	–	–
26	–	–	–	–
27	–	–	–	–
28	0.90 s	0.90 s	19.3	19.3
29	0.85 s	0.84 s	25.4	25.4
30	0.89 s	0.89 s	15.1	15.1
Ac	–	–	170.9	171.0
	2.05 s	2.05 s	21.3	21.3

All chemical shifts are given in ppm. The coupling constants are in parentheses and are given in Hz. The spectra of the synthetic material were measured in $CDCl_3$ at 298 K. ^{1}H-NMR spectrum was recorded at 400 MHz. ^{13}C-NMR spectrum was recorded at 101 MHz.
The spectra of the natural product were measured in $CDCl_3$ and referenced to TMS as an internal standard. ^{1}H-NMR spectrum was recorded at 700 MHz. ^{13}C-NMR spectrum was recorded at 176 MHz.
For better comparison of the NMR data, the spectra of the synthetic material were referenced to the solvent signals in the spectra of the natural product, which are marked at δ_H = 7.261 ppm and δ_C = 77.001 ppm.

Table 12: NMR comparison of parkeol (**3**) and synthetic material.[9]

No.	^{1}H-NMR		^{13}C-NMR	
	Literature[a]	synthetic	Literature[a]	synthetic
1	–*<br>–*	1.79 m<br>1.49 m	36.1	36.3
2	–*<br>–*	1.72 m<br>1.64 m	27.8	28.0
3	3.22 dd (12.0, 4.6)	3.21 dd (11.5, 4.4)	78.9	79.1
4	–	–	39.1	39.3
5	–*	0.88 m	52.5	52.6
6	–*<br>–*	1.68 m<br>1.47 m	21.4	21.5
7	–*<br>–*	1.63 m<br>1.30 m	28.1	28.3
8	–*	2.17 m	41.8	41.9
9	–	–	148.5	148.7
10	–	–	39.4	39.5
11	5.22 d (6.3)	5.22 dt (6.3, 2.0)	115.0	115.1
12	–*<br>–*	2.07 m<br>1.90 m	37.2	37.3
13	–	–	44.3	44.4
14	–	–	47.0	47.2
15	–*<br>–*	1.32 m<br>1.36 m	33.9	34.1
16	–*<br>–*	1.90 m<br>1.30 m	28.0	28.2
17	–*	1.60 m	50.9	51.1
18	0.65 s	0.65 s	14.4	14.5
19	1.04 s	1.04 s	22.3	22.4

20	–*	1.38 m	35.9	36.1
21	0.90 d (6.3)	0.89 d (6.5)	18.3	18.5
22	–*<br>–*	1.45 m<br>1.03 m	36.4	36.6
23	–*<br>–*	2.03 m<br>1.86 m	24.9	25.1
24	5.09 t (6.9)	5.10 ddq (7.1, 5.6, 1.4)	125.2	125.4
25	–	–	130.9	131.1
26	1.68 s	1.69 d (1.4)	25.7	25.9
27	1.60 s	1.60 s	17.6	17.8
28	0.99 s	0.99 s	28.2	28.4
29	0.82 s	0.82 s	15.7	15.8
30	0.74 s	0.74 s	18.5	18.6

All chemical shifts are given in ppm. The coupling constants are in parentheses and are given in Hz. The spectra were measured in $CDCl_3$. The spectra of the synthetic material referenced to the residual solvent signal at δ_H = 7.26 ppm and δ_C = 77.16 ppm. The comparison spectrum was referenced with tetramethylsilane as an internal standard.

–*: Signals were not specified in the reference spectrum.
[a]: The signals were not assigned.

Table 13: NMR comparison of natural 25,26,27-Trinor-3α-hydroxy-17,13-friedolanostan-8,12-dien-23-one ("Sibiricanone", **5**) and synthetic material.[12]

No.	^{1}H-NMR		^{13}C-NMR	
	natural	synthetic	natural	synthetic
1	1.49 m<br>1.53 m	1.49 m<br>1.53 m	29.5	29.4
2	1.60 m<br>1.94 m	1.60 m<br>1.94 m	25.6	25.6
3	3.42 t (2.5)	3.42 t (2.5)	75.8	75.8
4	–	–	37.4	37.4
5	1.51 m	1.51 m	44.7	44.6
6	1.45 m<br>1.61 m	1.45 m<br>1.62 m	18.2	18.2
7	2.06 m<br>2.09 m	2.06 m<br>2.09 m	25.5	25.5
8	–	–	134.6	134.5
9	–	–	136.3	136.3
10	–	–	36.9	36.8
11	2.49 ddt (20.0, 3.0, 1.6)<br>2.54 ddt (20.0, 6.0, 1.6)	2.47 ddt (20.3, 4.0, 2.2)<br>2.56 ddt (20.4, 6.2, 1.7)	26.2	26.2
12	5.37 dd (6.0, 2.2)	5.36 dd (6.1, 2.0)	116.3	116.3
13	–	–	155.3	155.2
14	–	–	47.6	47.6
15	1.50 m<br>1.52 m	1.50 m<br>1.53 m	34.8	34.7
16	1.26 ddd (12.0, 6.0, 1.6)<br>1.74 dt (12.0, 7.5)	1.26 ddd (12.4, 6.6, 1.2)<br>1.75 td (12.4, 6.9)	33.9	33.8
17	–	–	46.8	46.8
18	0.91 d (0.7)	0.91 s	26.6	26.6
19	0.91 d (0.5)	0.91 s	19.0	18.9

20	2.08 m	2.08 m	38.0	38.0
21	0.84 dd (6.5, 1.2)	0.84 d (6.1)	15.9	15.9
22	2.14 m<br>2.70 m	2.14 m<br>2.69 dd (15.2, 2.7)	47.2	47.2
23	–	–	209.6	209.7
24	2.10 s	2.10 s	30.2	30.3
25	–	–	–	–
26	–	–	–	–
27	–	–	–	–
28	0.96 s	0.96 s	27.9	27.9
29	0.85 s	0.85 s	22.0	22.0
30	0.89 d (0.4)	0.89 s	23.5	23.4

All chemical shifts are given in ppm. The coupling constants are in parentheses and are given in Hz. The spectra of the synthetic material were measured in $CDCl_3$ at 298 K. ^{1}H-NMR spectrum was recorded at 600 MHz. ^{13}C-NMR spectrum was recorded at 151 MHz.

The spectra of the natural product were measured in $CDCl_3$ and referenced to TMS as an internal standard. ^{1}H-NMR spectrum was recorded at 400 MHz. ^{13}C-NMR spectrum was recorded at 101 MHz.

For better comparison of the NMR data, the spectra of the synthetic material were referenced to the signals on C3 in the spectra of the natural product, which are marked at δ_H = 3.416 ppm and δ_C = 75.81 ppm.

S12 Recrystallizations of γ-Oryzanol.

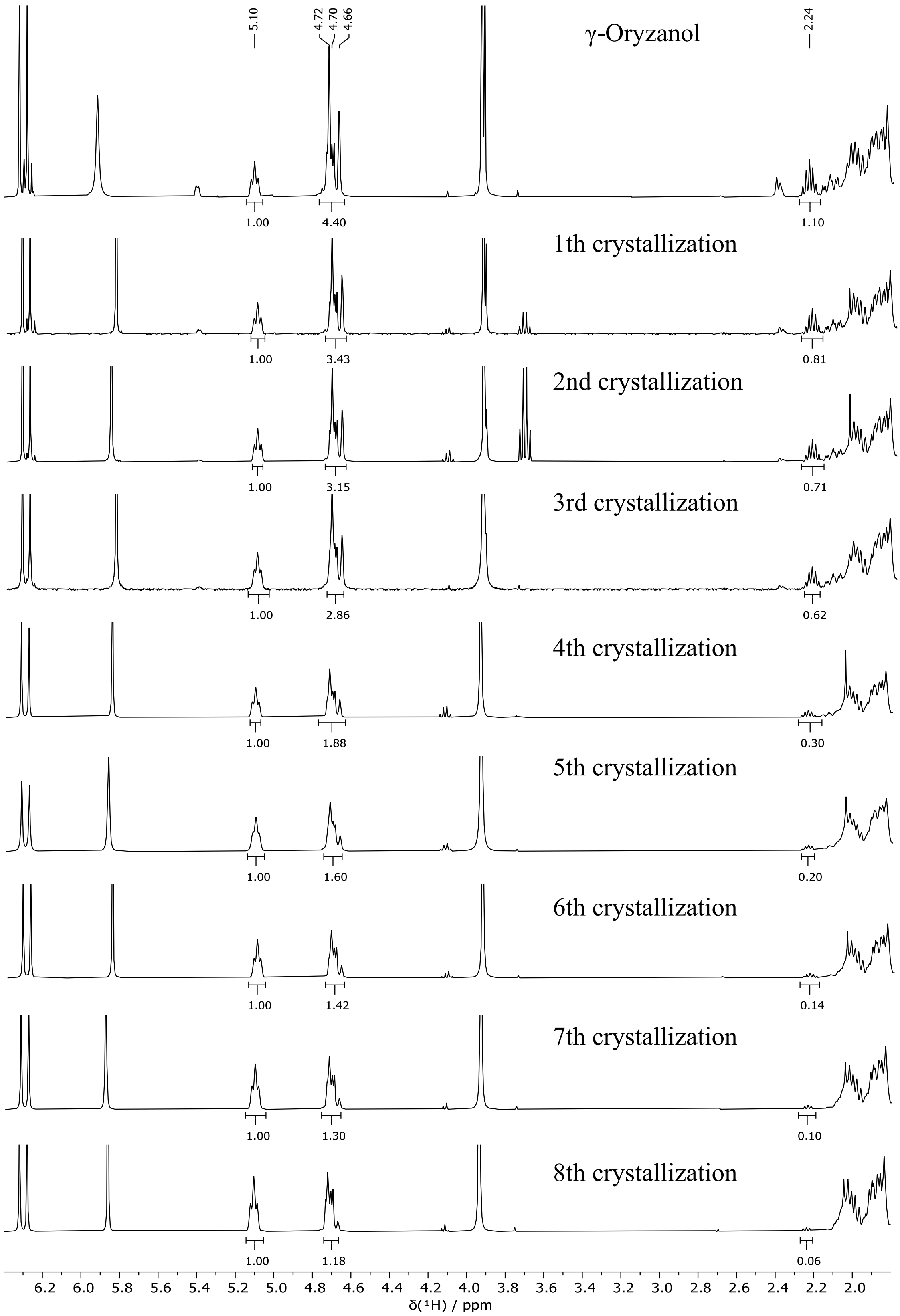

Figure 4: NMR-spectra (400 MHz in $CDCl_3$) after crystallizations of γ-Oryzanol.

Table 14: Appearance after recrystallizations of γ-Oryzanol.

#	hot solution	after crystallization	appearance	CAF (**12**): mCAF (**14**)
/			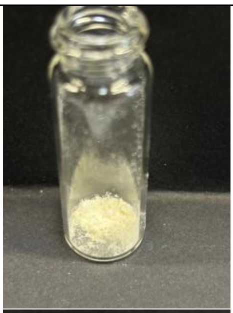	100:110
1.	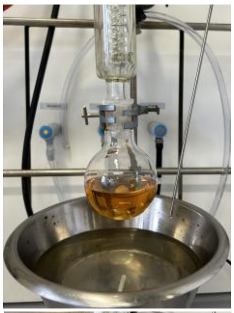	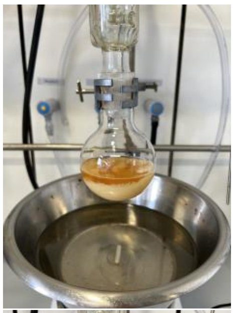	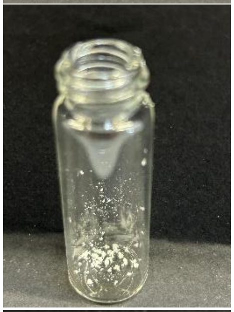	100:81
2.	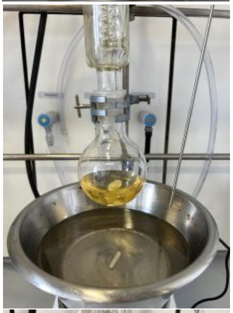	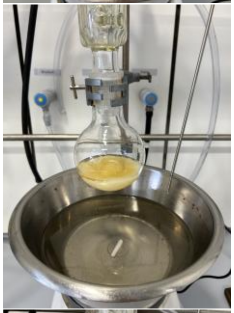	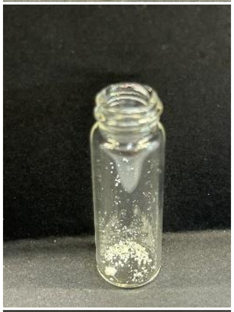	100:71
3.	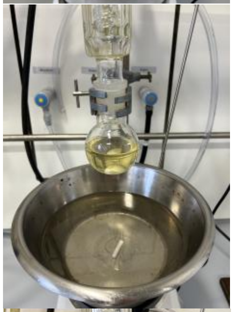	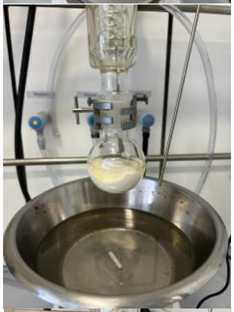	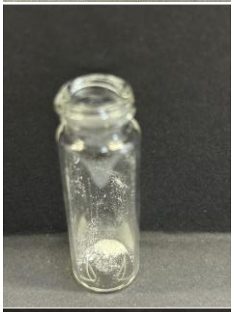	100:62
4.	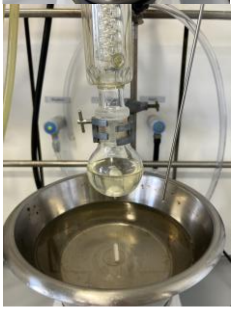	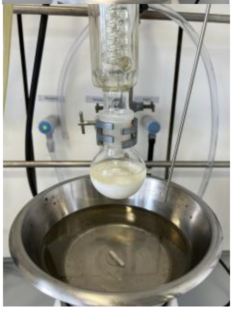	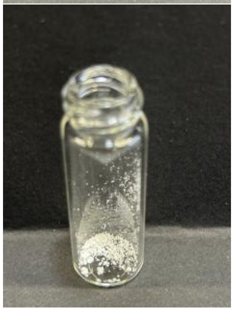	100:30

5.	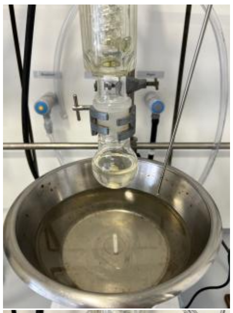	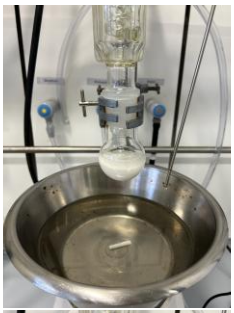	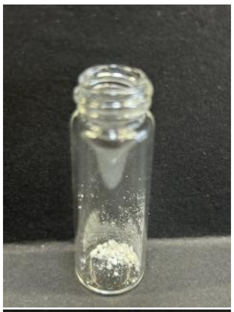	100:20
6.	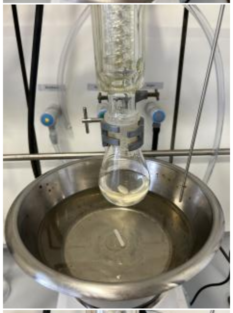	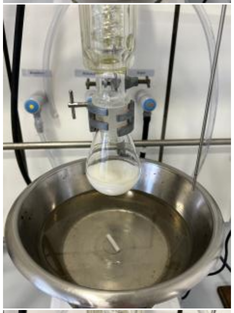	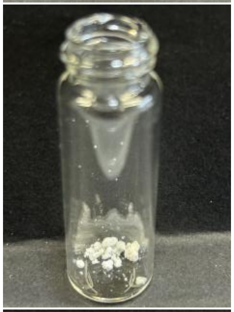	100:14
7.	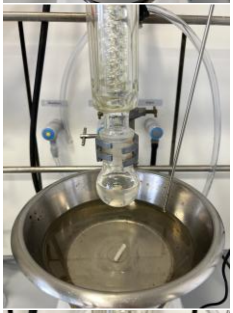	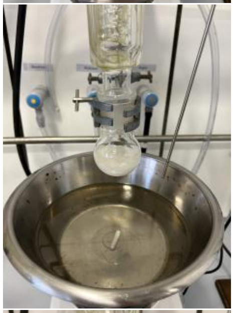	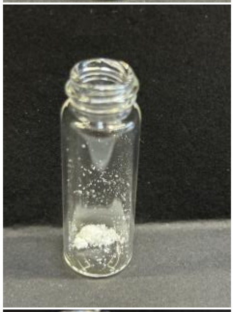	100:10
8.	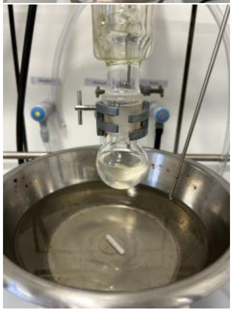	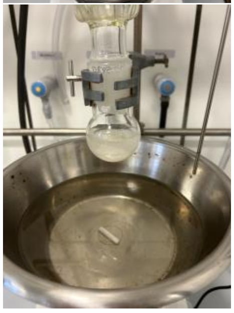	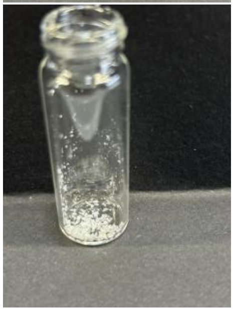	100:6

S13 References.

[1] Li, T.-S.; Li, J.-T.; Li, H.-Z. Modified and convenient preparation of silica impregnated with silver nitrate and its application to the separation of steroids and triterpenes. *J. Chromatogr. A.*, **1995**, 715, 372–375. DOI: 10.1016/0021-9673(95)00619-X.

[2] Yoshida, K.; Hirose, Y.; Imai, Y.; Kondo, T. Conformational Analysis of Cycloartenol, 24-Methylenecycloartanol and Their Derivatives. *Agric. Biol. Chem*. **1989**, *53*, 1901. DOI: 10.1080/00021369.1989.10869552.

[3] Greca, M. D.; Fiorentino, A.; Monaco, P.; Previtera, L. Cycloartane triterpenes from *Juncus Effusus*. *Phytochem*., **1994**, *35*, 1017–1022. DOI: 10.1016/S0031-9422(00)90659-9.

[4] Böhme, H. Monoperphthalic acid. *Org. Synth.*, **1940**, *20*, 70. DOI: 10.15227/orgsyn.042.0077.

[5] Ponomarenko, L. P.; Kalinovsky, A. I.; Martyyas, E. A.; Doudkin, R. V.; Gorovoy, Petr G.; Stonik, V. A. Terpenoid metabolites from the aerial part of *Artemisia lagocephala. Phytochem. Lett.* **2012**, *5*, 118. DOI: 10.1016/j.phytol.2011.11.006.

[6] Zhao, Z.-Y.; Tong, Y.-P.; Jiang, W.; Zang, Y.; Xiong, J.; Li, J.; Hu, J.-F. Structurally Diverse Triterpene-26-oic Acids as Potential Dual ACL and ACC1 Inhibitors from the Vulnerable Conifer *Keteleeria fortunei*. *J. Nat. Prod.* **2023**, *86*, 1487. DOI: 10.1021/acs.jnatprod.3c00181.

[7] Feilner, J. M.; Plangger, I.; Wurst, K.; Magauer, T. Bifunctional Polyene Cyclizations: Synthetic Studies on Pimarane Natural Products. *Chem. Eur. J.*, **2021**, *27*, 12410–12421. DOI: 10.1002/chem.202101926.

[8] Watterson, S. H.; Ni, Z.; Murphree, S. S.; Padwa, A. 2,3-Dibromo-(phenylsulfonyl)-1-propene as a versatile reagent for the synthesis of furans and cyclopentenones: 2-methyl-4-[(phenyl-sulfonyl)methyl]furan and 2-methyl-3-[(phenylsulfonyl)methyl]-2-cyclopenten-1-one. *Org. Synth.*, **1997**, *74*, 115. DOI: 10.15227/orgsyn.074.0115.

[9] Sawai, S.; Akashi, T.; Sakurai, N.; Suzuki, H.; Shibata, D.; Ayabe, S.-i.; Aoki, T. Plant Lanosterol Synthase: Divergence of the Sterol and Triterpene Biosynthetic Pathways in Eukaryotes. *Plant. Cell. Physiol.*, **2006**, *47(5)*, 673–677. DOI: 10.1093/pcp/pcj032.

[10] Barton, D. H. R.; Kempe, U. M.; Widdowson, D. A. Investigations on the Biosynthesis of Steroids and Terpenoids. Part VI. The sterols of yeast. *J. Chem. Soc., Perkin Trans. 1*, **1972**, 513–522. DOI: 10.1039/P19720000513.

[11] Alekseychuk, M.; Adrian, S., Heinze, R. C.; Heretsch, P. Biogenesis-Inspired, Divergent Synthesis of Spirochensilide A, Spirochesilide B, and Abifarine B Employing a Radical-Polar Crossover Rearrangement Strategy. *J. Am. Chem. Soc.* **2022**, *144*, 11574. DOI: 10.1021/jacs.2c05358.

[12] Grishko, V. V.; Shakirov, M. M.; Raldugin, V. A. Grishko, V. V.; Shakirov, M. M.; Raldugin, V. A. Triterpenoids from *Abies* species 20.* Identification of new trinortriterpene methyl ketones in the products of alkaline degradation of total acids of *Abies sibirica* needles. *Russ. Chem. Bull.* **1996**, *45*, 2847. DOI: 10.1007/bf02495313.

[13] Neese, F. Software update: The ORCA program system—Version 5.0. *WIREs Comput. Mol. Sci.* **2022**, *12*, e1606. DOI: 10.1002/wcms.1606.

[14] Bannwarth, C.; Ehlert, S.; Grimme, S. GFN2-xTB-An Accurate and Broadly Parametrized Self-Consistent Tight-Binding Quantum Chemical Method with Multipole Electrostatics and Density-Dependent Dispersion Contributions. *J. Chem. Theory Comput*. **2019**, *15*, 1652. DOI: 10.1021/acs.jctc.8b01176.

S14 NMR-spectra.

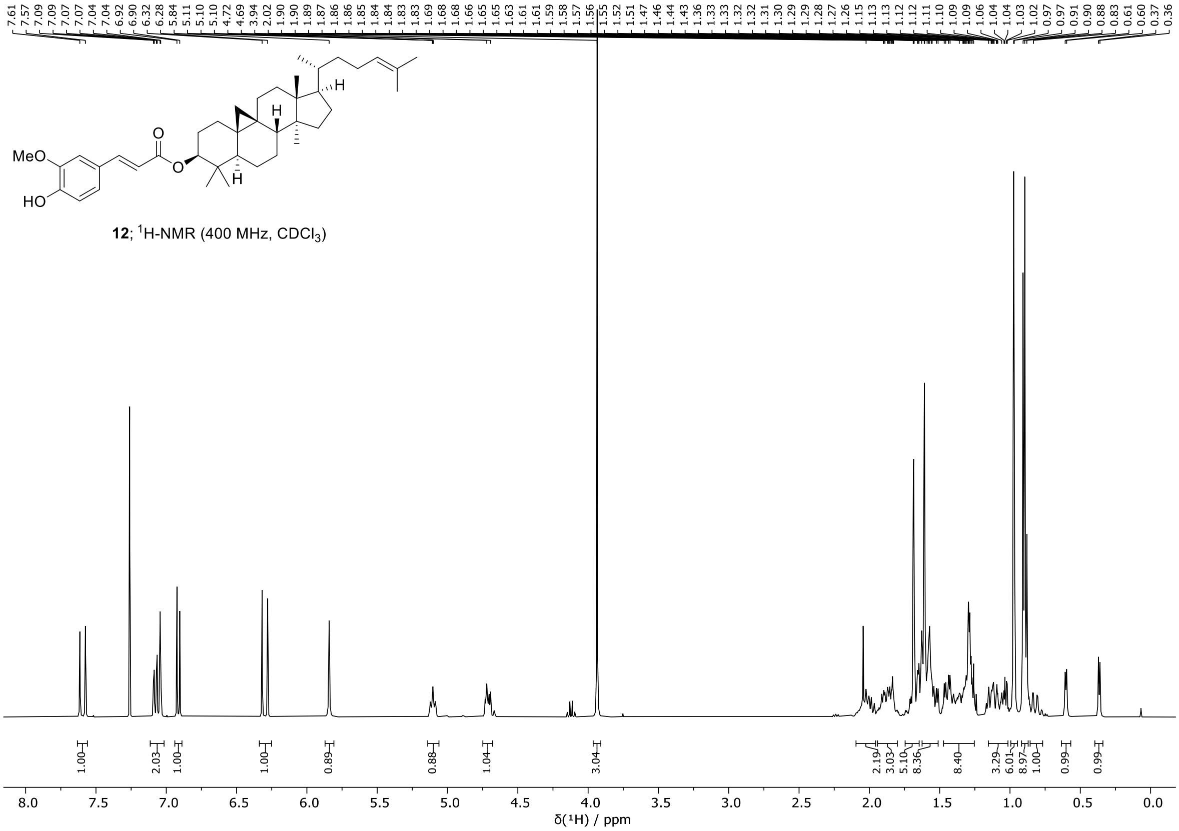

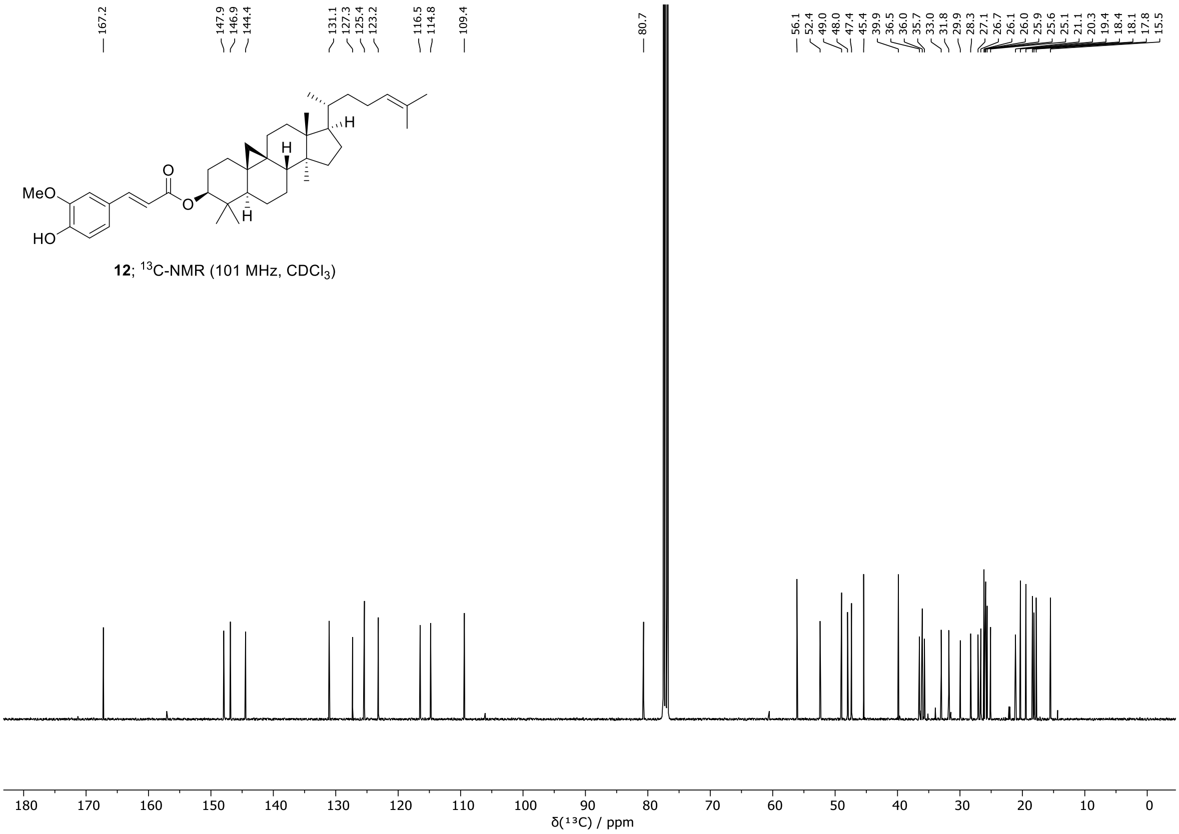

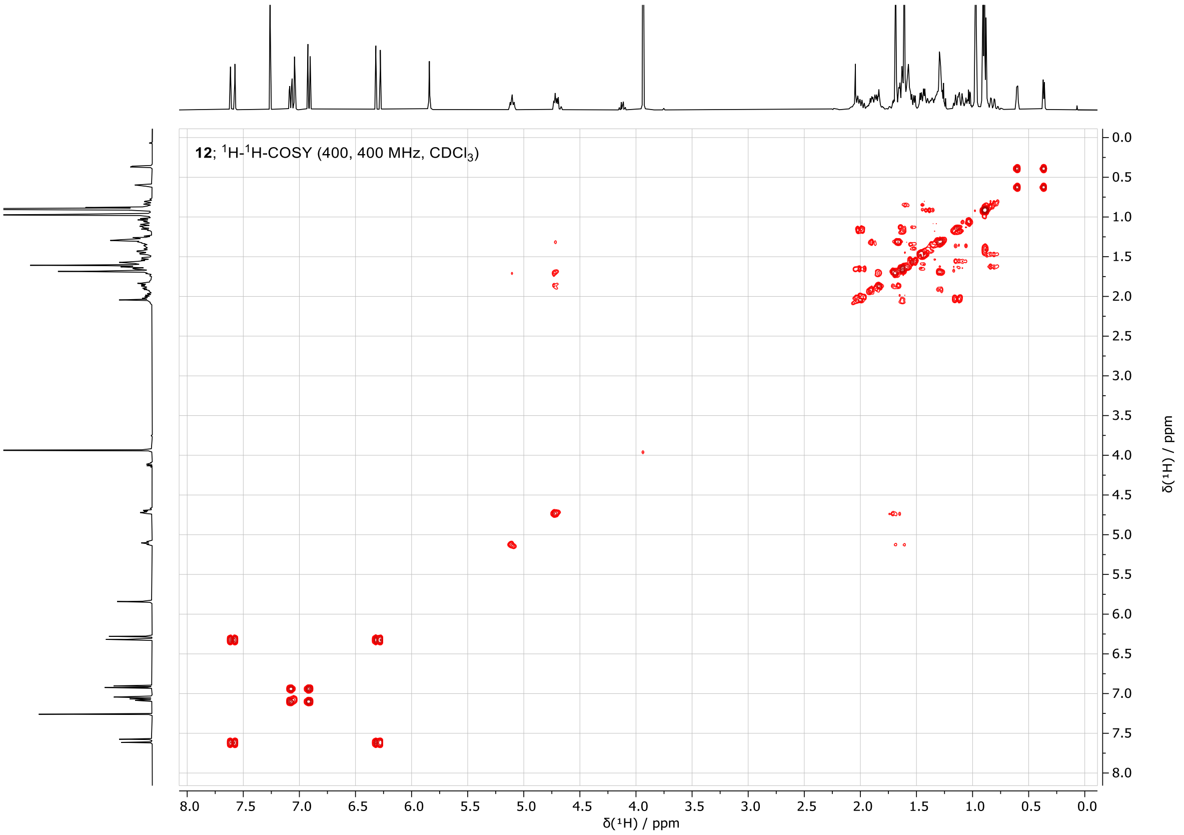
12; ^{1}H-^{1}H-COSY (400, 400 MHz, $CDCl_3$)
δ(¹H) / ppm
δ(¹H) / ppm

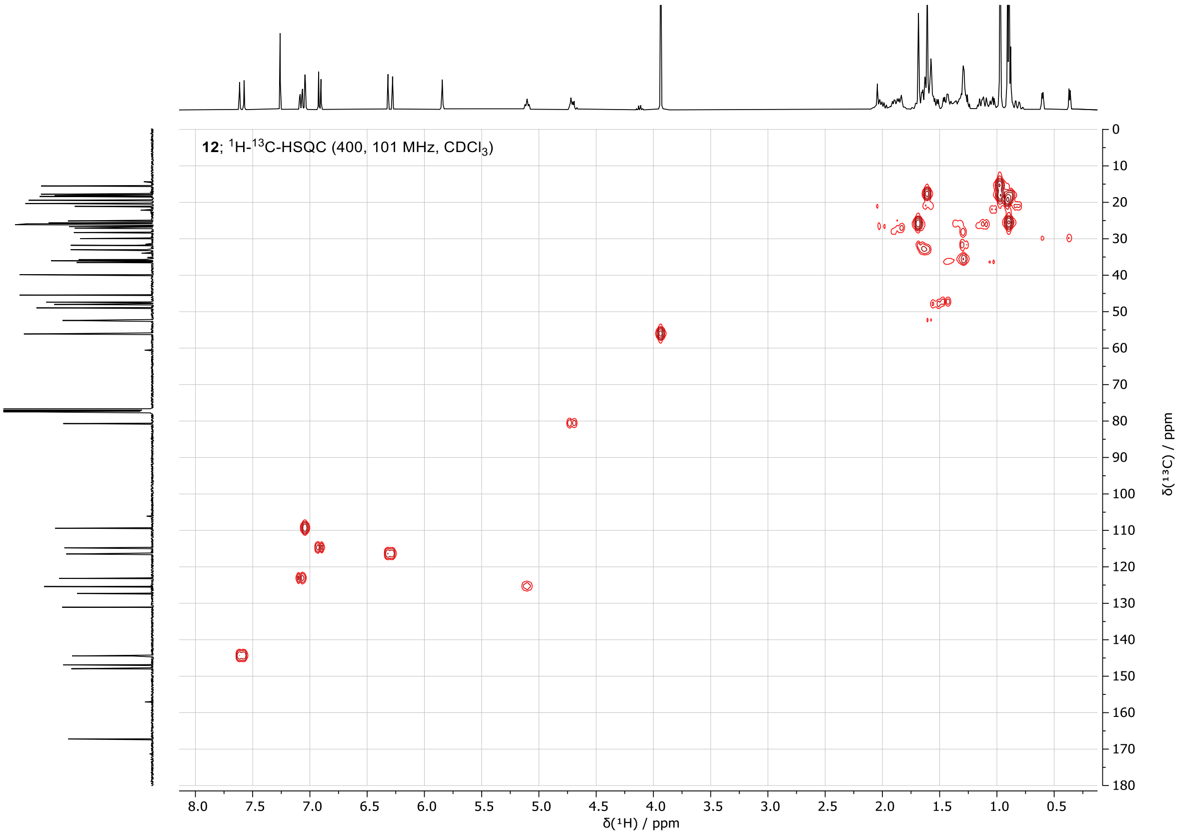
12; ^{1}H-^{13}C-HSQC (400, 101 MHz, $CDCl_3$)
δ(¹H) / ppm
δ(¹³C) / ppm

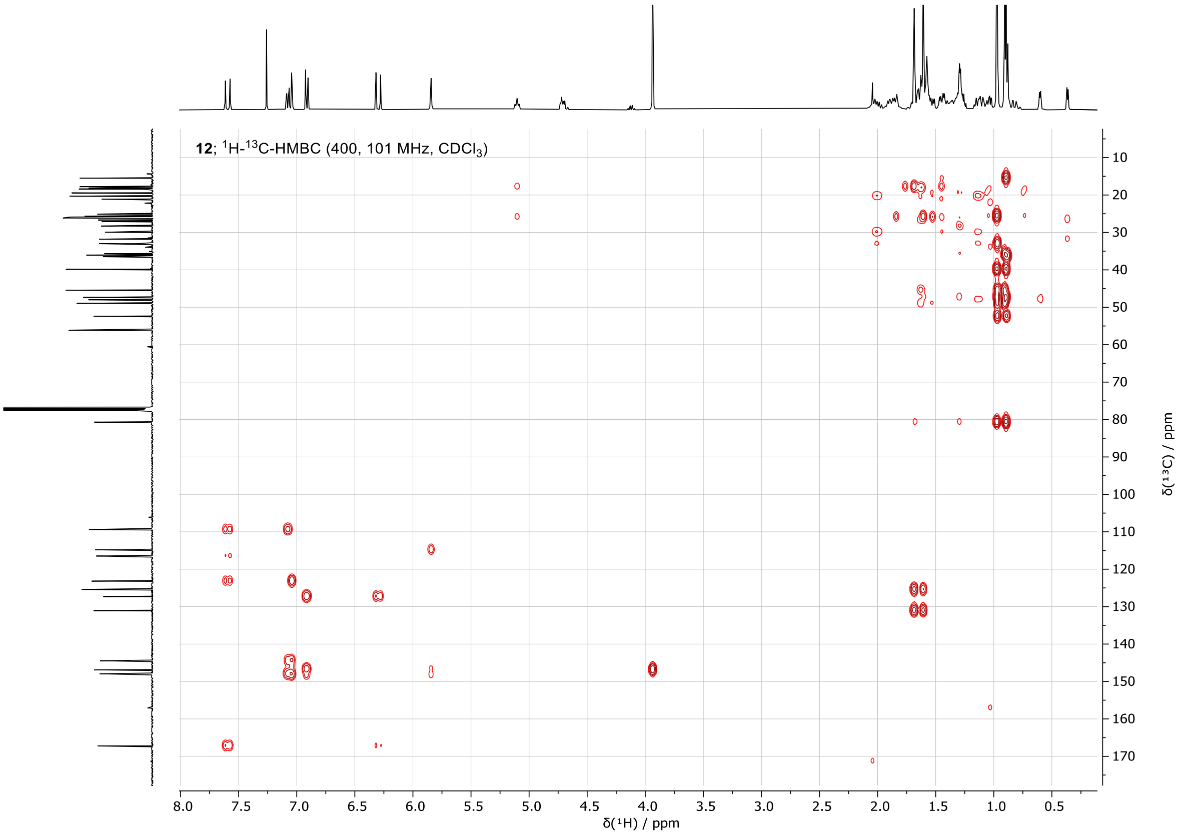
12; 1H-13C-HMBC (400, 101 MHz, CDCl3)
δ(¹³C) / ppm
δ(¹H) / ppm

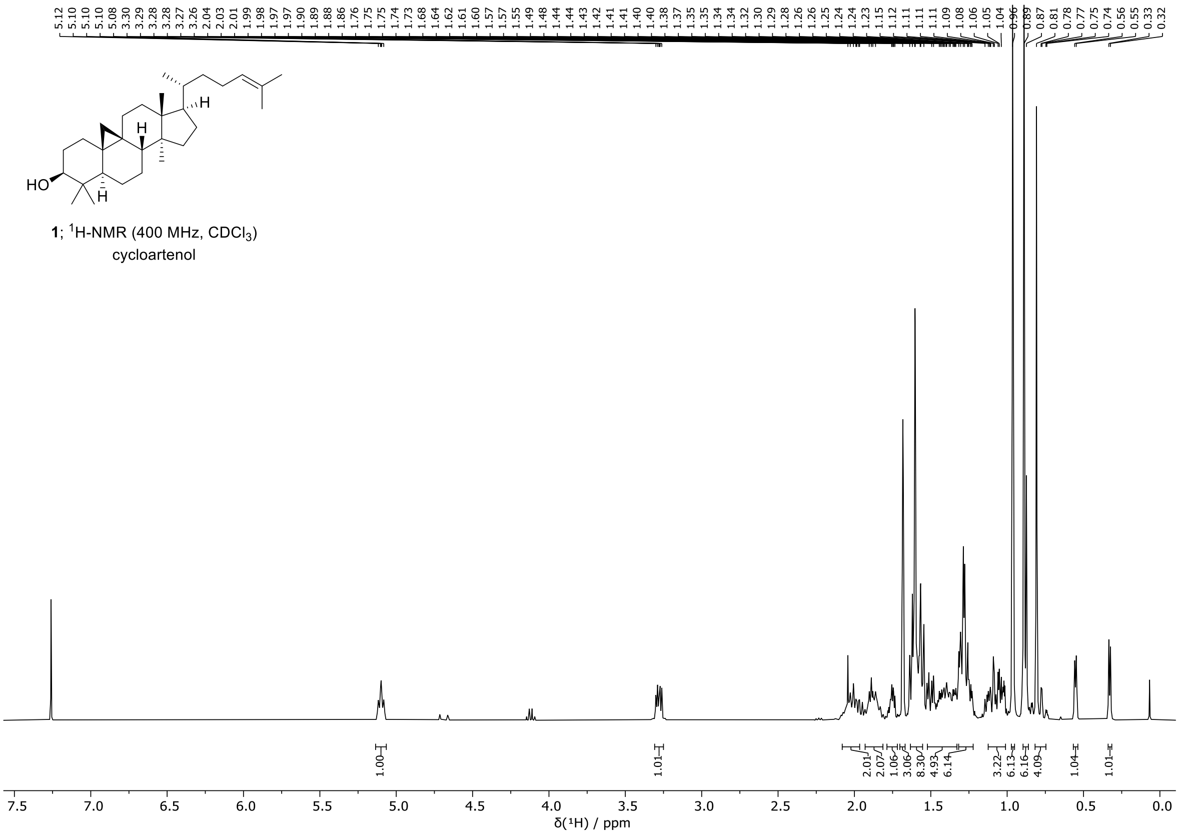
5.12 5.10 5.10 5.10 5.08 3.30 3.29 3.28 3.28 3.27 3.26 2.04 2.03 2.01 1.99 1.98 1.97 1.97 1.90 1.89 1.88 1.86 1.76 1.75 1.75 1.74 1.73 1.68 1.64 1.62 1.61 1.60 1.57 1.55 1.49 1.48 1.44 1.43 1.42 1.41 1.40 1.38 1.37 1.35 1.35 1.34 1.32 1.30 1.29 1.28 1.26 1.25 1.24 1.24 1.23 1.15 1.12 1.11 1.11 1.11 1.09 1.08 1.06 1.05 1.04 0.96 0.89 0.87 0.81 0.78 0.77 0.75 0.74 0.56 0.55 0.33 0.32
HO
H
1; 1H-NMR (400 MHz, CDCl3)
cycloartenol
1.00
1.01
2.01
2.07
1.06
3.06
8.30
4.93
6.14
3.22
6.13
6.16
4.09
1.04
1.01
δ(¹H) / ppm

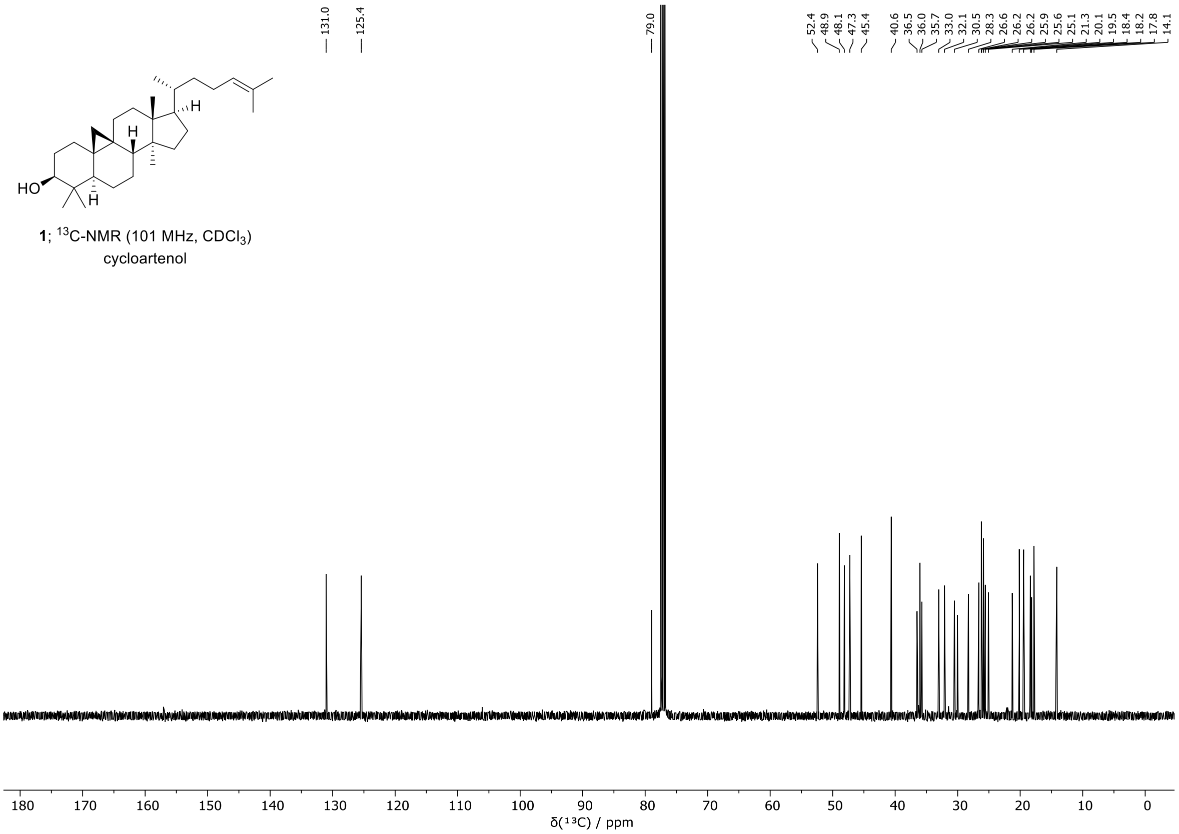
1; ^{13}C-NMR (101 MHz, $CDCl_3$)
cycloartenol
HO
H
131.0
125.4
79.0
52.4
48.9
48.1
47.3
45.4
40.6
36.5
36.0
35.7
33.0
32.1
30.5
28.3
26.6
26.2
26.2
25.9
25.6
25.1
21.3
20.1
19.5
18.4
18.2
17.8
14.1
δ(^{13}C) / ppm

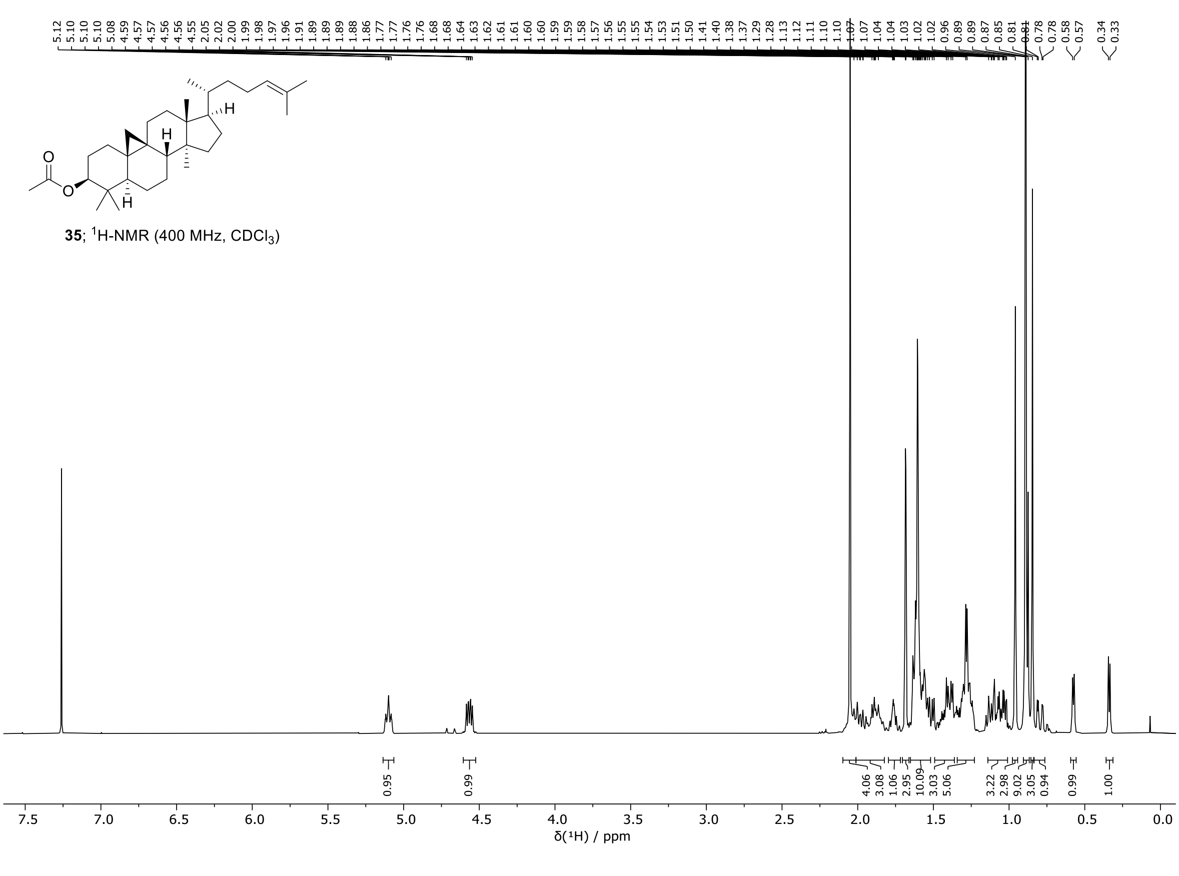
35; ^{1}H-NMR (400 MHz, $CDCl_3$)
O
H
0.95
0.99
4.06
3.08
1.06
2.95
10.09
3.03
5.06
3.22
2.98
9.02
3.05
0.94
0.99
1.00
δ(^{1}H) / ppm

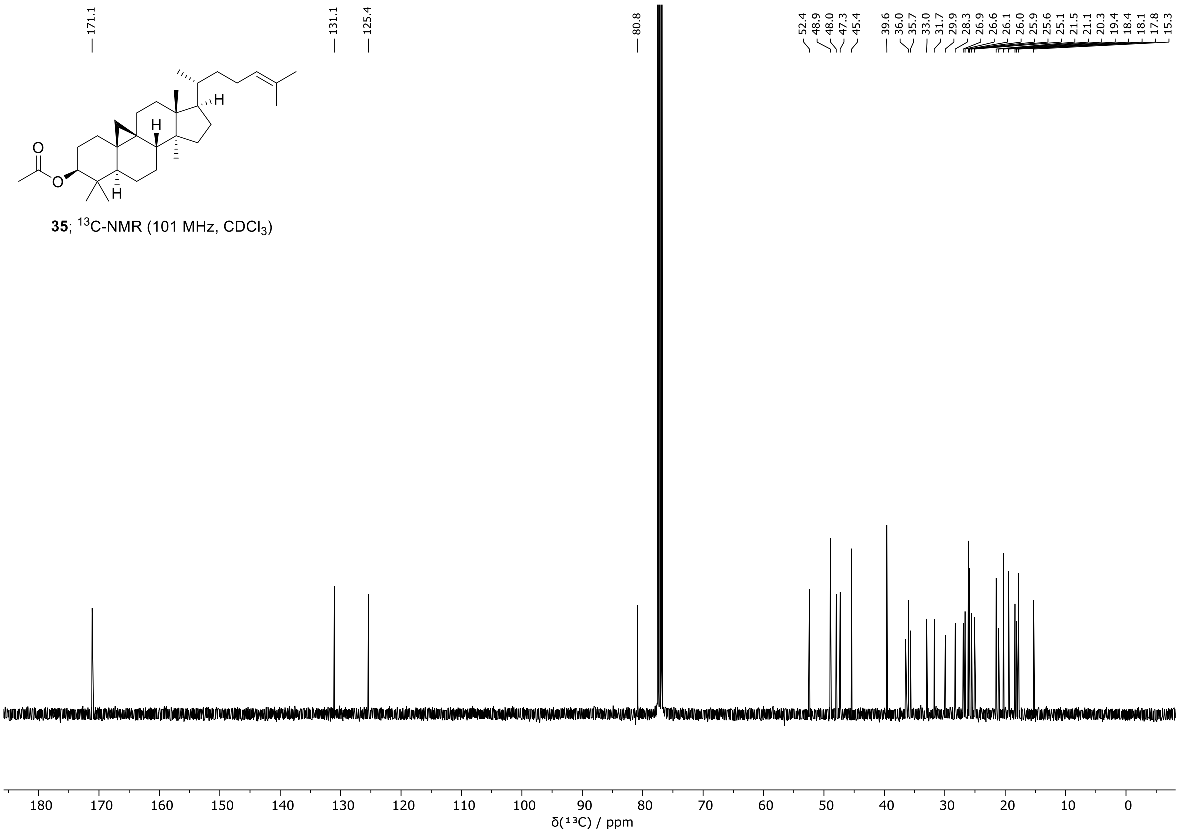
171.1
131.1
125.4
80.8
52.4 48.9 48.0 47.3 45.4 39.6 36.0 35.7 33.0 31.7 29.9 28.3 26.9 26.6 26.1 26.0 25.9 25.6 25.1 21.5 21.1 20.3 19.4 18.4 18.1 17.8 15.3
H
H
O
O
H
35; ^{13}C-NMR (101 MHz, $CDCl_3$)
180 170 160 150 140 130 120 110 100 90 80 70 60 50 40 30 20 10 0
δ(^{13}C) / ppm

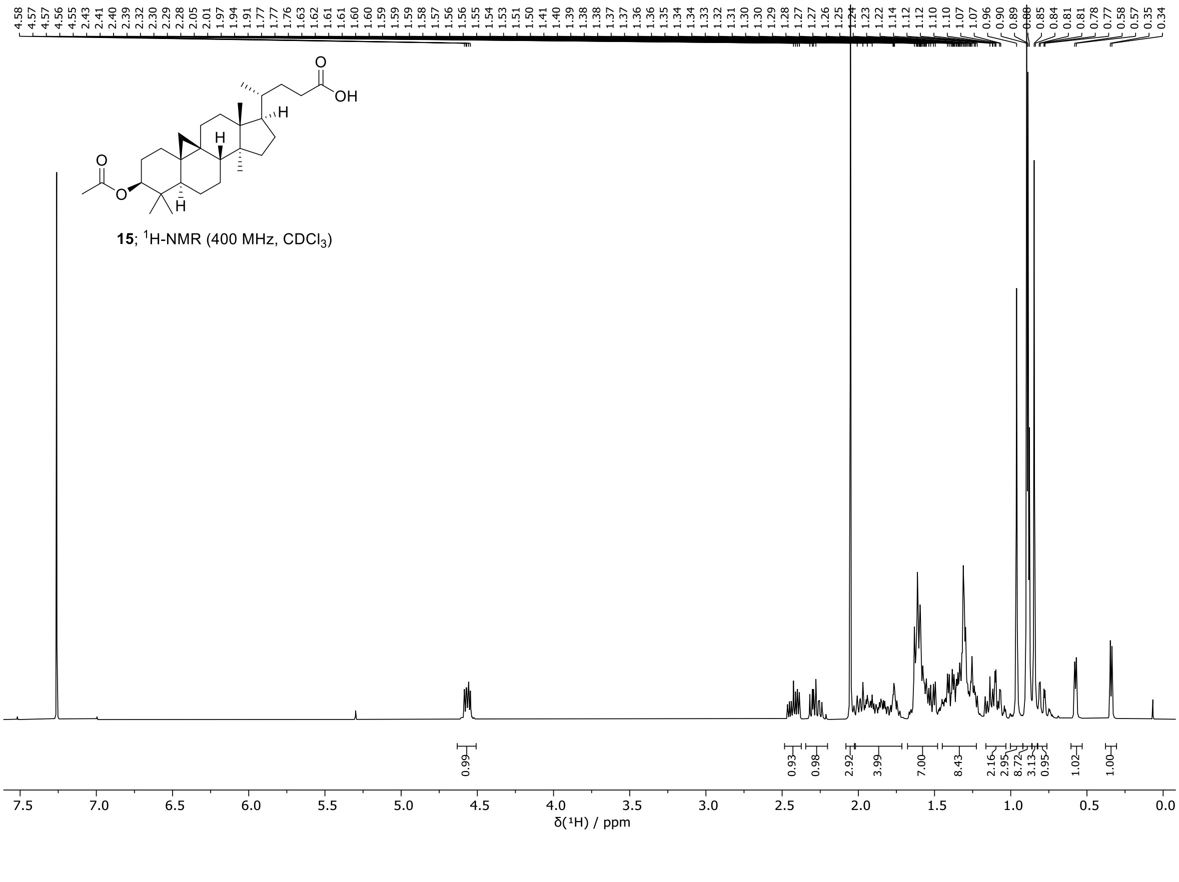
4.58 4.57 4.57 4.56 4.55 2.43 2.41 2.40 2.39 2.32 2.30 2.29 2.28 2.05 2.01 1.97 1.94 1.91 1.77 1.77 1.76 1.63 1.62 1.61 1.61 1.60 1.59 1.59 1.59 1.58 1.57 1.56 1.55 1.54 1.53 1.51 1.50 1.41 1.40 1.39 1.38 1.38 1.37 1.37 1.36 1.36 1.35 1.34 1.34 1.33 1.32 1.31 1.30 1.30 1.29 1.28 1.27 1.27 1.26 1.25 1.24 1.23 1.22 1.14 1.12 1.12 1.10 1.07 1.07 0.96 0.90 0.89 0.88 0.85 0.84 0.81 0.81 0.78 0.77 0.58 0.57 0.35 0.34
O
OH
H
H
O
O
H
15; ^{1}H-NMR (400 MHz, $CDCl_3$)
0.99
0.93 0.98 2.92 3.99 7.00 8.43 2.16 2.95 8.72 3.13 0.95 1.02 1.00
7.5 7.0 6.5 6.0 5.5 5.0 4.5 4.0 3.5 3.0 2.5 2.0 1.5 1.0 0.5 0.0
δ(^{1}H) / ppm

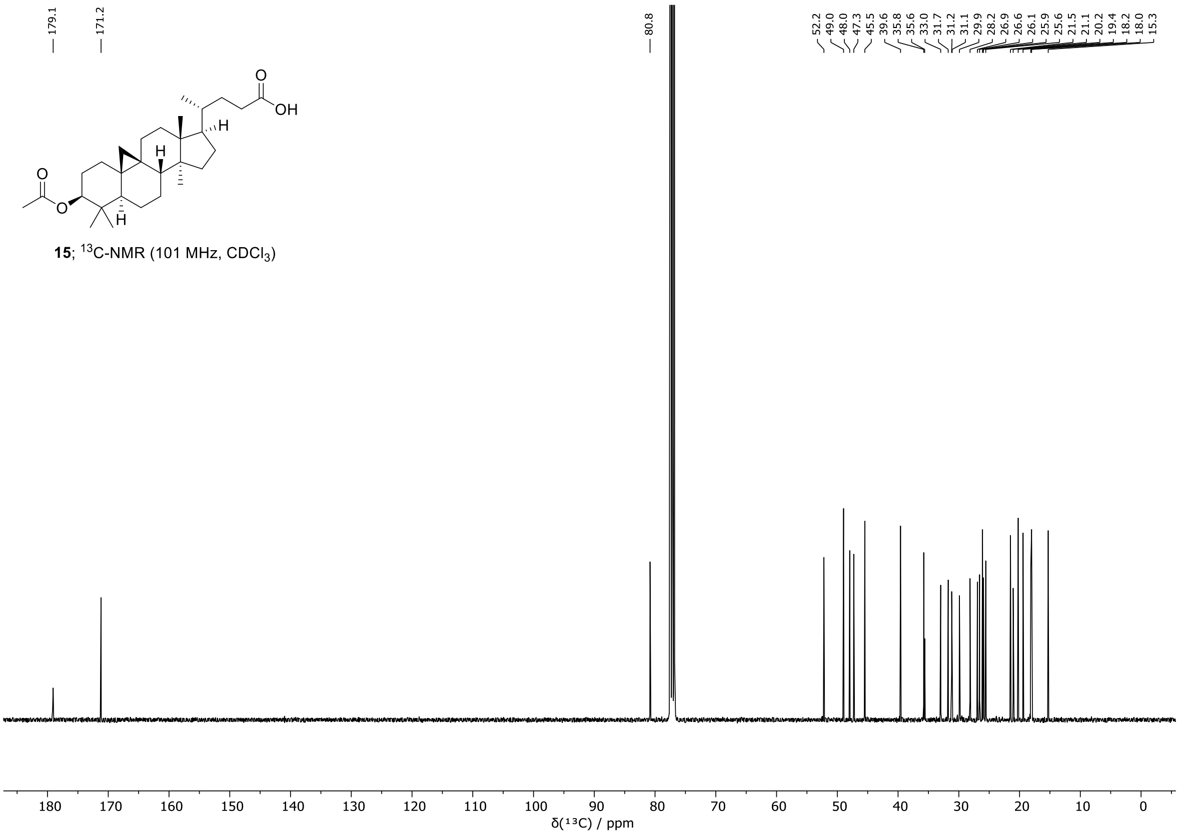
179.1
171.2
80.8
52.2
49.0
48.0
47.3
45.5
39.6
35.8
35.6
33.0
31.7
31.2
31.1
29.9
28.2
26.9
26.6
26.1
25.9
25.6
21.5
21.1
20.2
19.4
18.2
18.0
15.3
O
OH
H
H
O
O
H
15; ^{13}C-NMR (101 MHz, $CDCl_3$)
180 170 160 150 140 130 120 110 100 90 80 70 60 50 40 30 20 10 0
δ(¹³C) / ppm

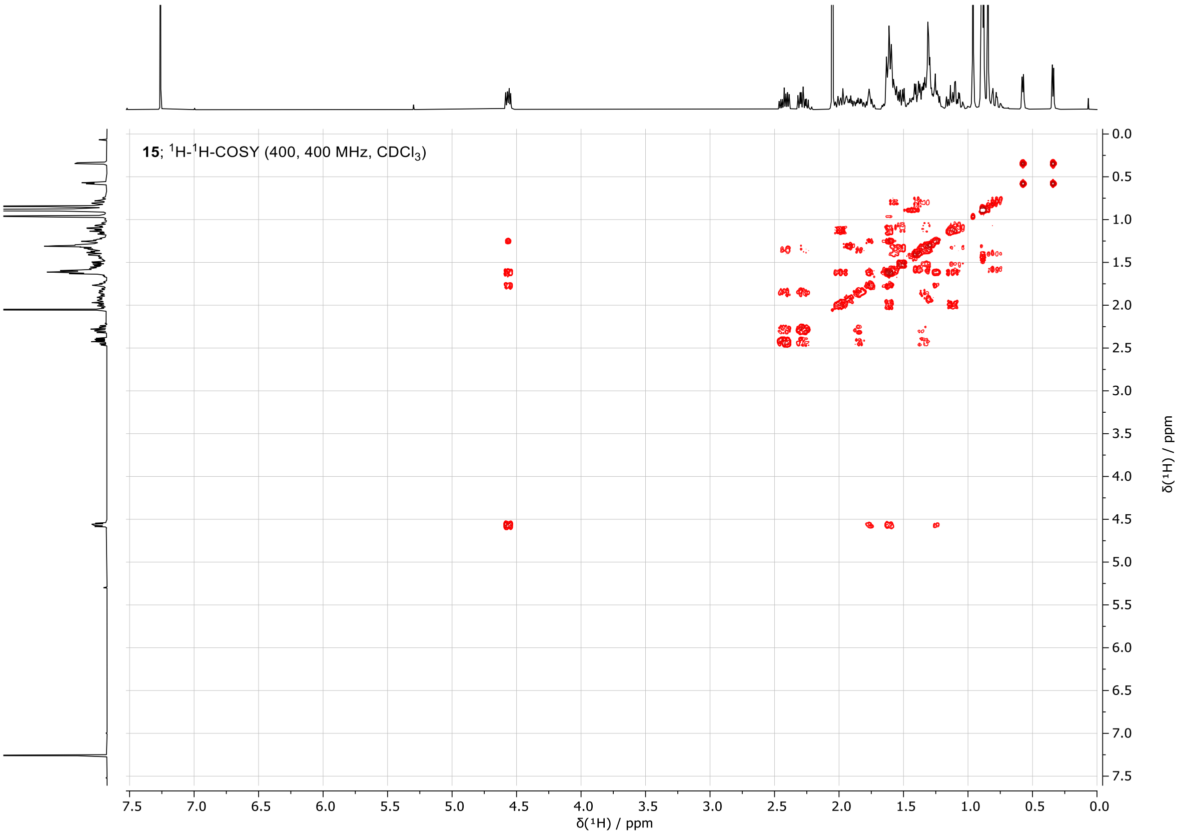
15; ^{1}H-^{1}H-COSY (400, 400 MHz, $CDCl_3$)
7.5 7.0 6.5 6.0 5.5 5.0 4.5 4.0 3.5 3.0 2.5 2.0 1.5 1.0 0.5 0.0
δ(¹H) / ppm
0.0 0.5 1.0 1.5 2.0 2.5 3.0 3.5 4.0 4.5 5.0 5.5 6.0 6.5 7.0 7.5
δ(¹H) / ppm

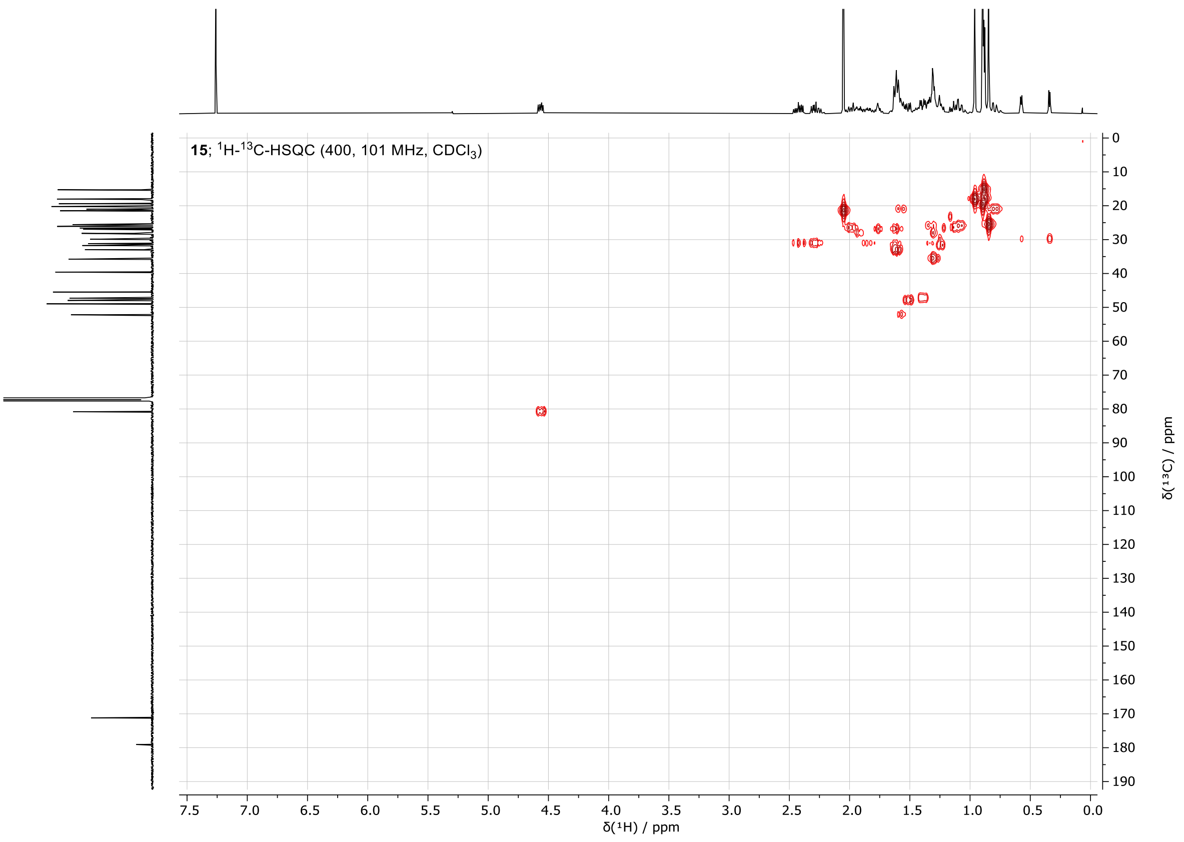
15; ¹H-¹³C-HSQC (400, 101 MHz, CDCl₃)
δ(¹H) / ppm
δ(¹³C) / ppm

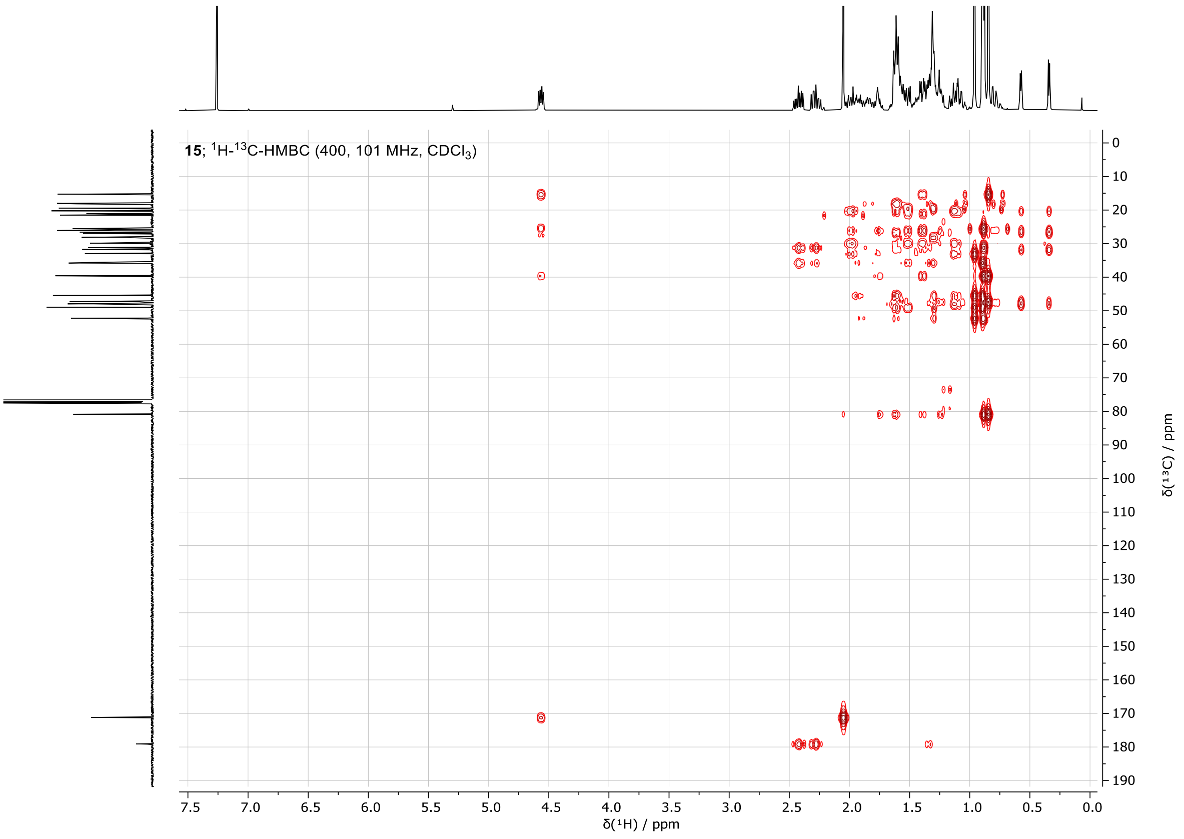
15; ¹H-¹³C-HMBC (400, 101 MHz, CDCl₃)
δ(¹H) / ppm
δ(¹³C) / ppm

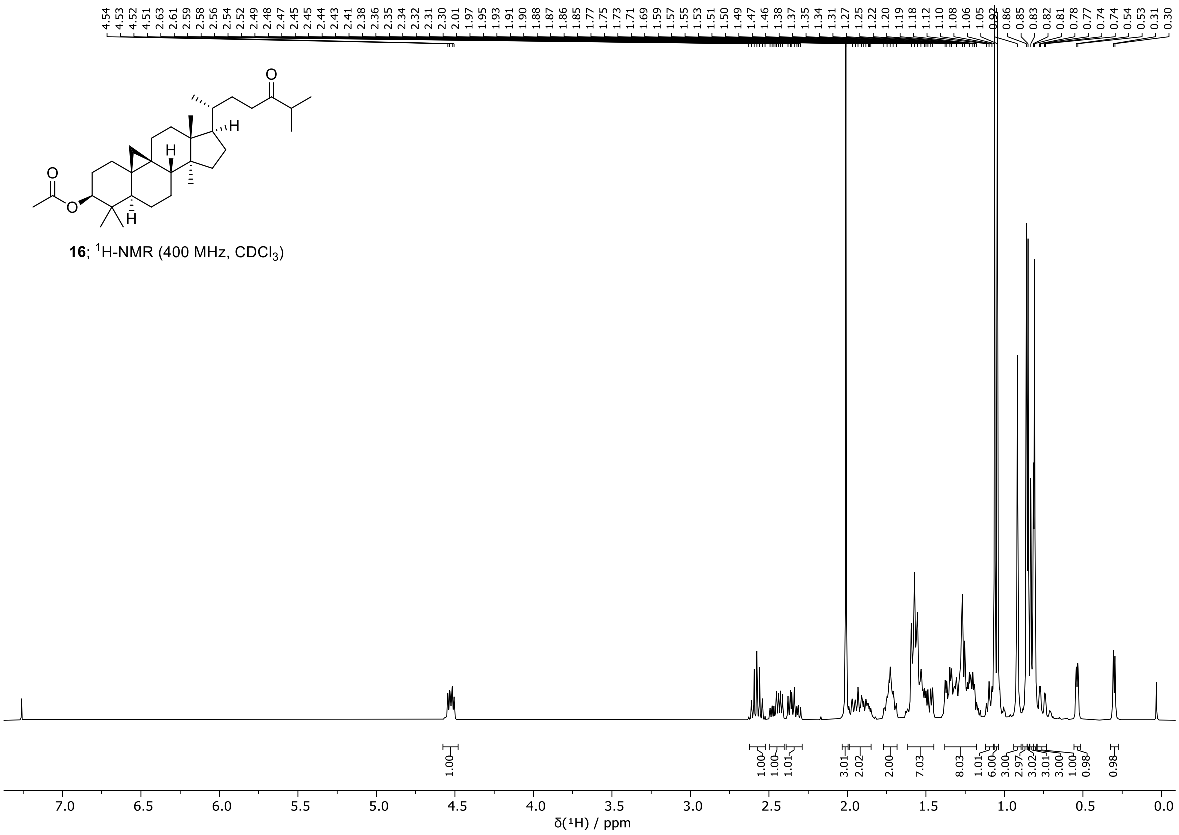

16; ^{1}H-NMR (400 MHz, $CDCl_3$)

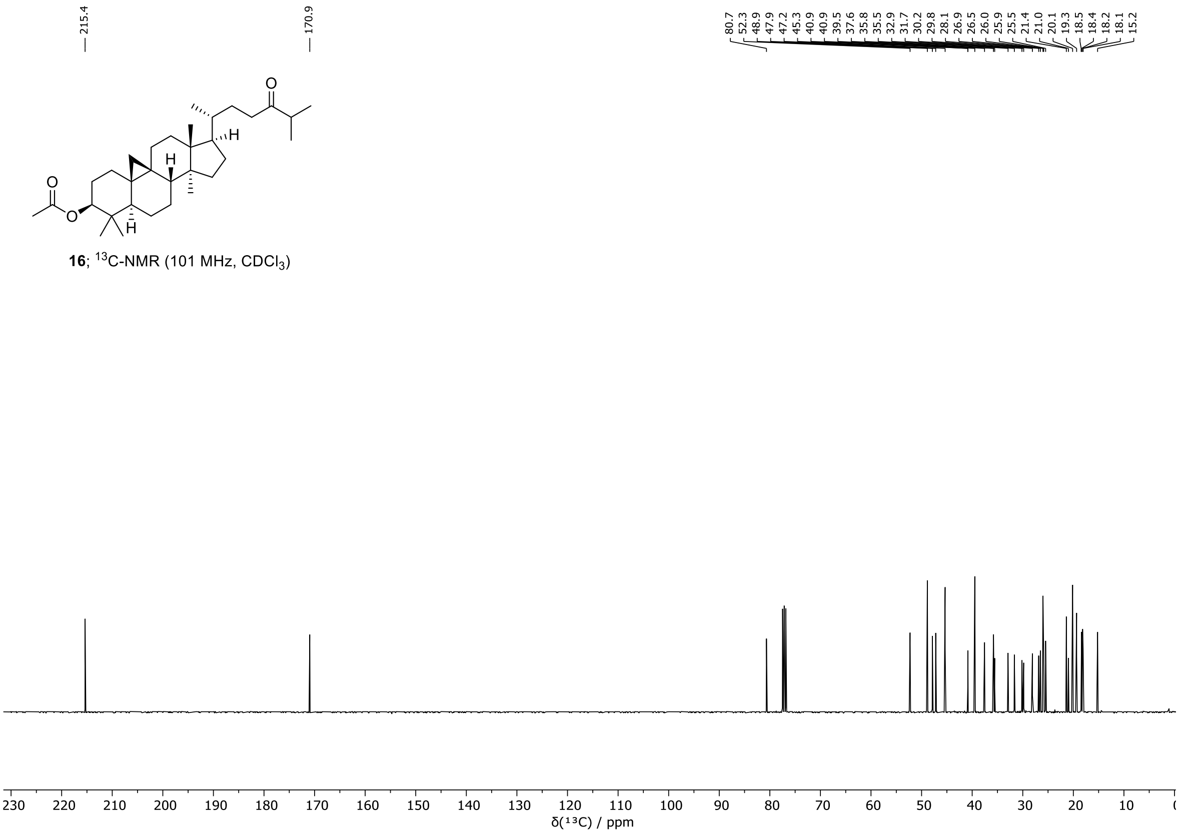

16; ^{13}C-NMR (101 MHz, $CDCl_3$)

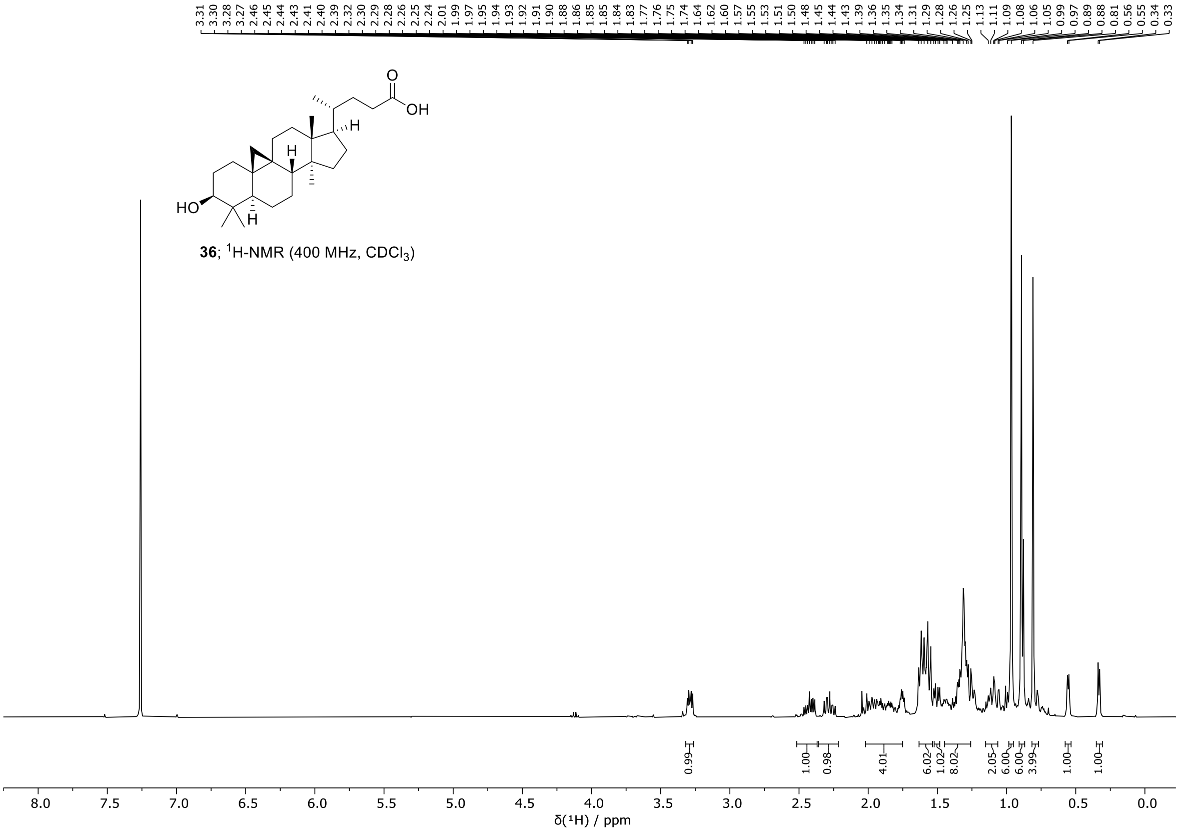

36; ^{1}H-NMR (400 MHz, $CDCl_3$)

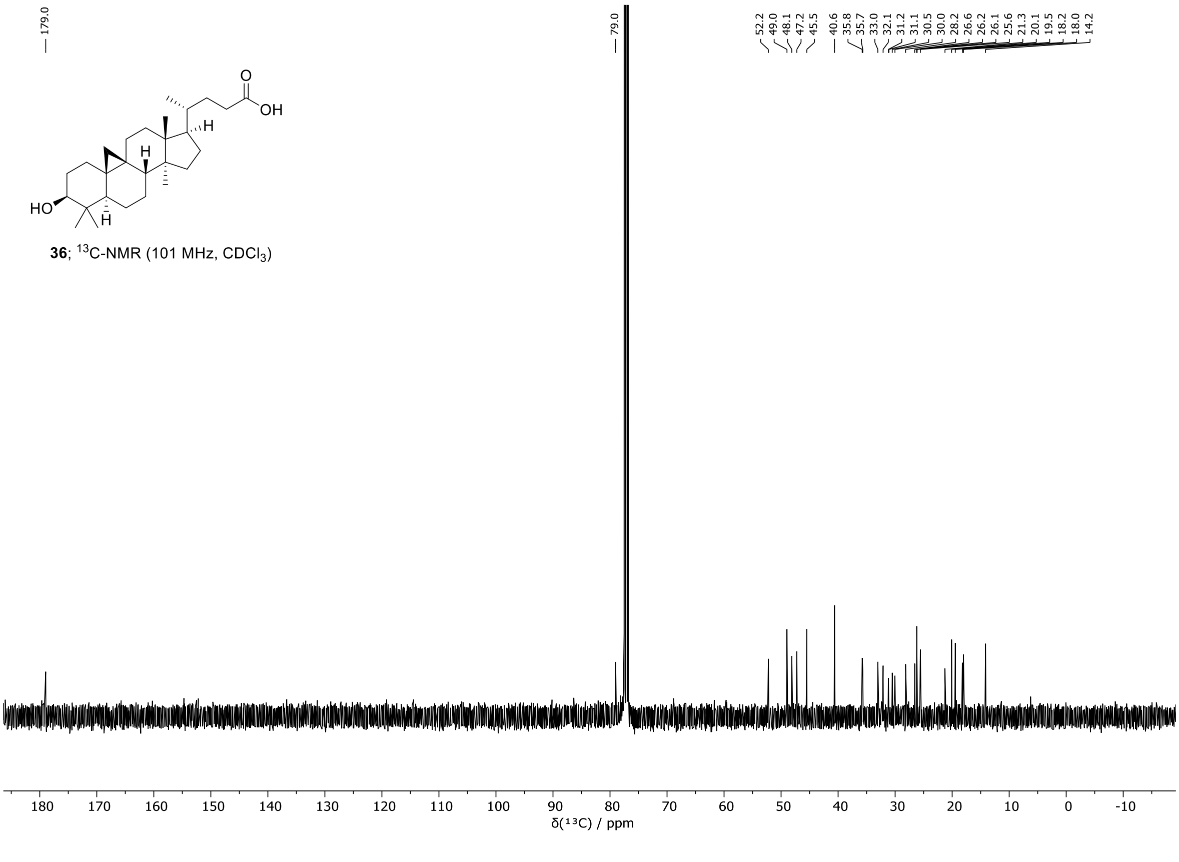

36; ^{13}C-NMR (101 MHz, $CDCl_3$)

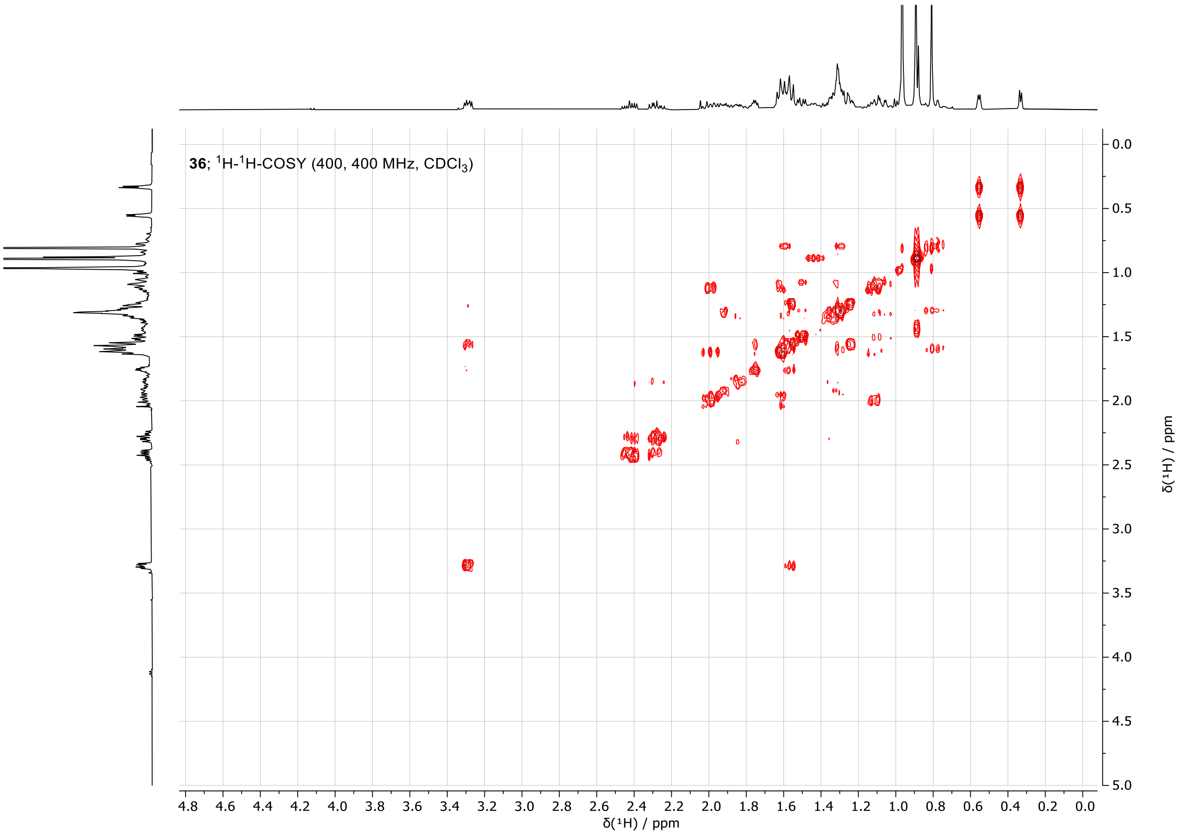
36; ¹H-¹H-COSY (400, 400 MHz, CDCl3)
δ(¹H) / ppm
δ(¹H) / ppm

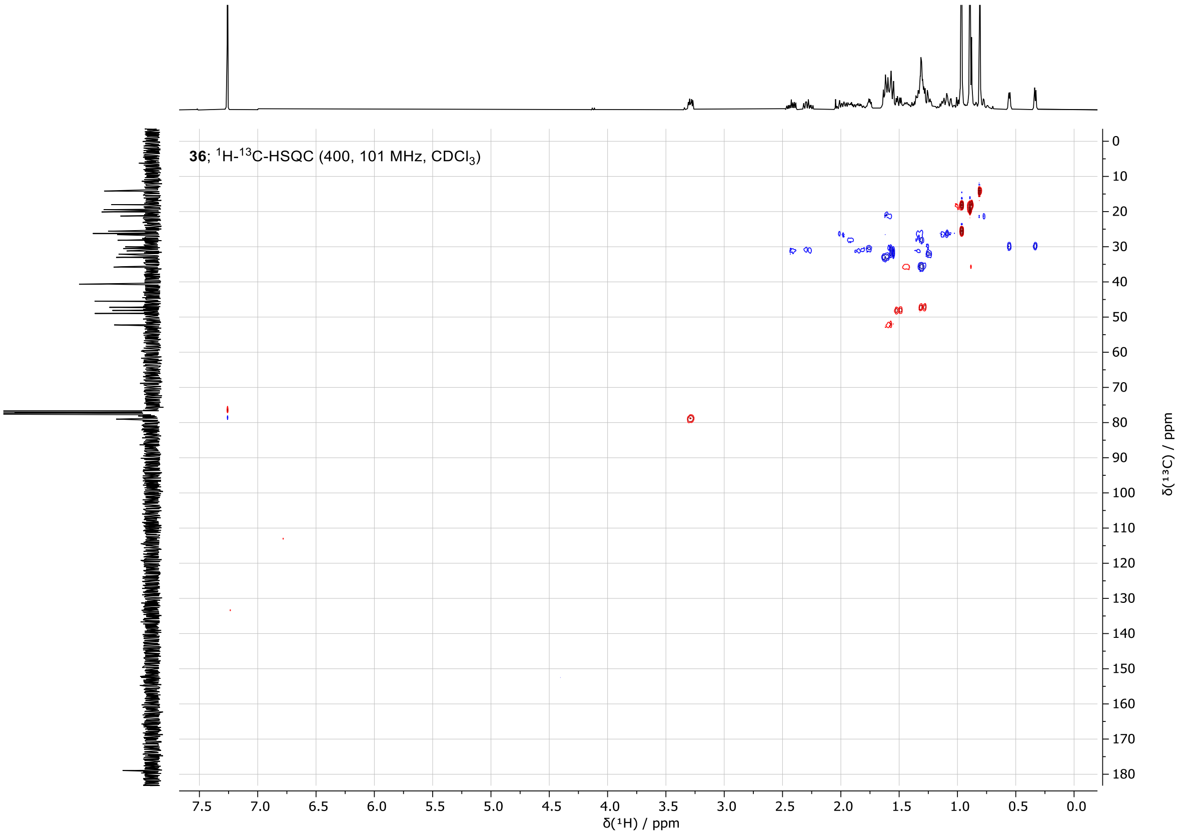
36; ¹H-¹³C-HSQC (400, 101 MHz, CDCl3)
δ(¹H) / ppm
δ(¹³C) / ppm

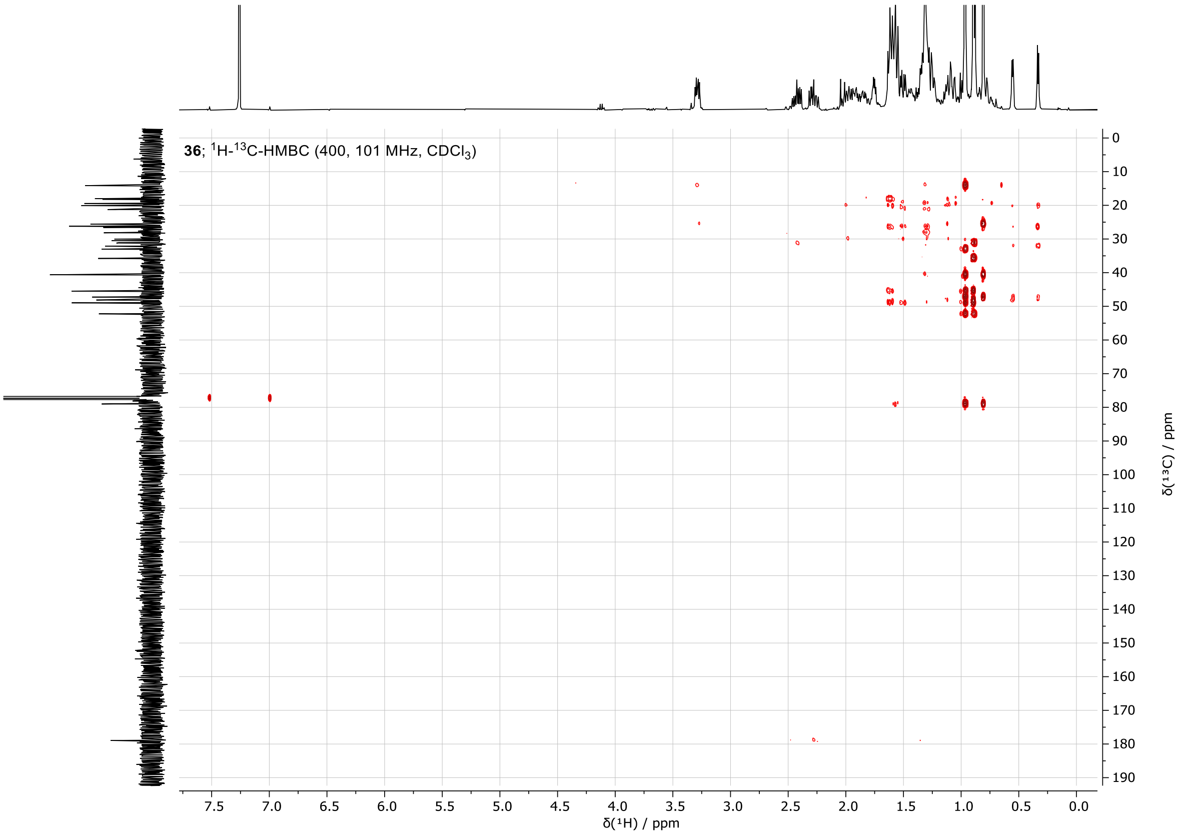

36; ^{1}H-^{13}C-HMBC (400, 101 MHz, $CDCl_3$)

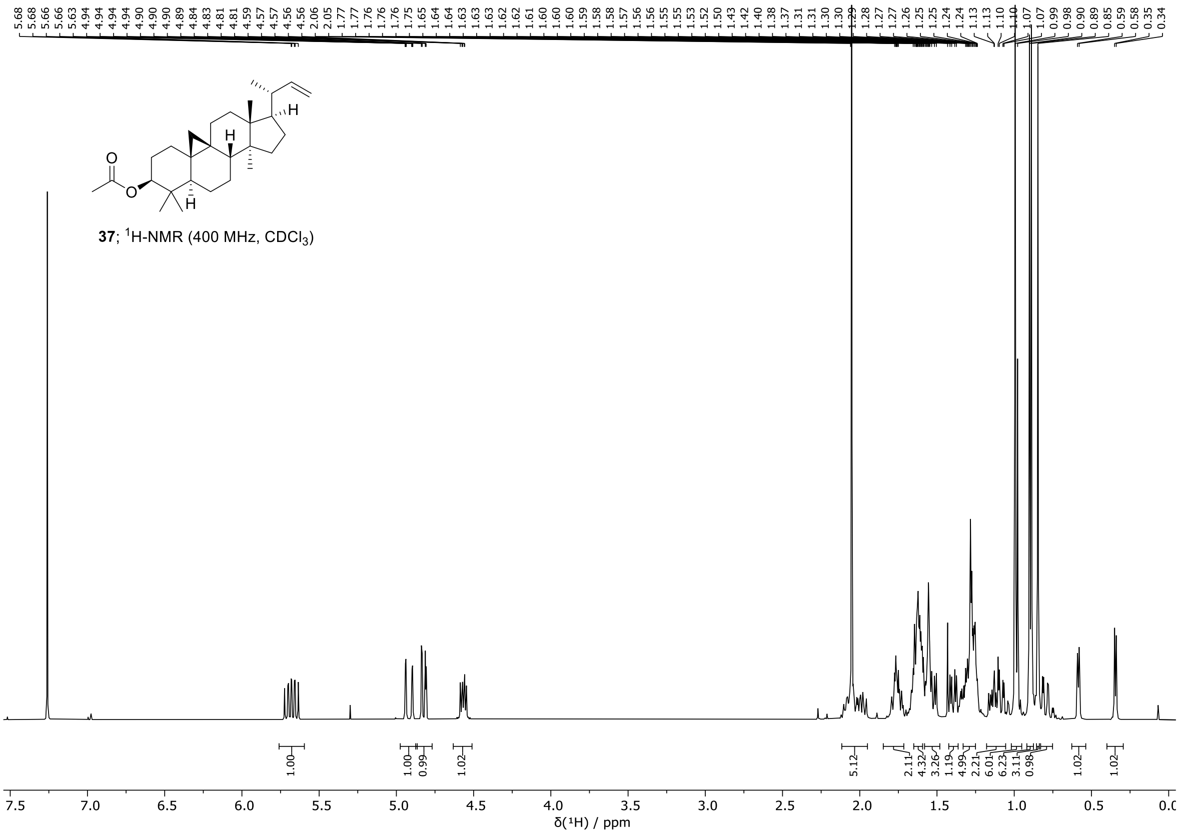

37; ^{1}H-NMR (400 MHz, $CDCl_3$)

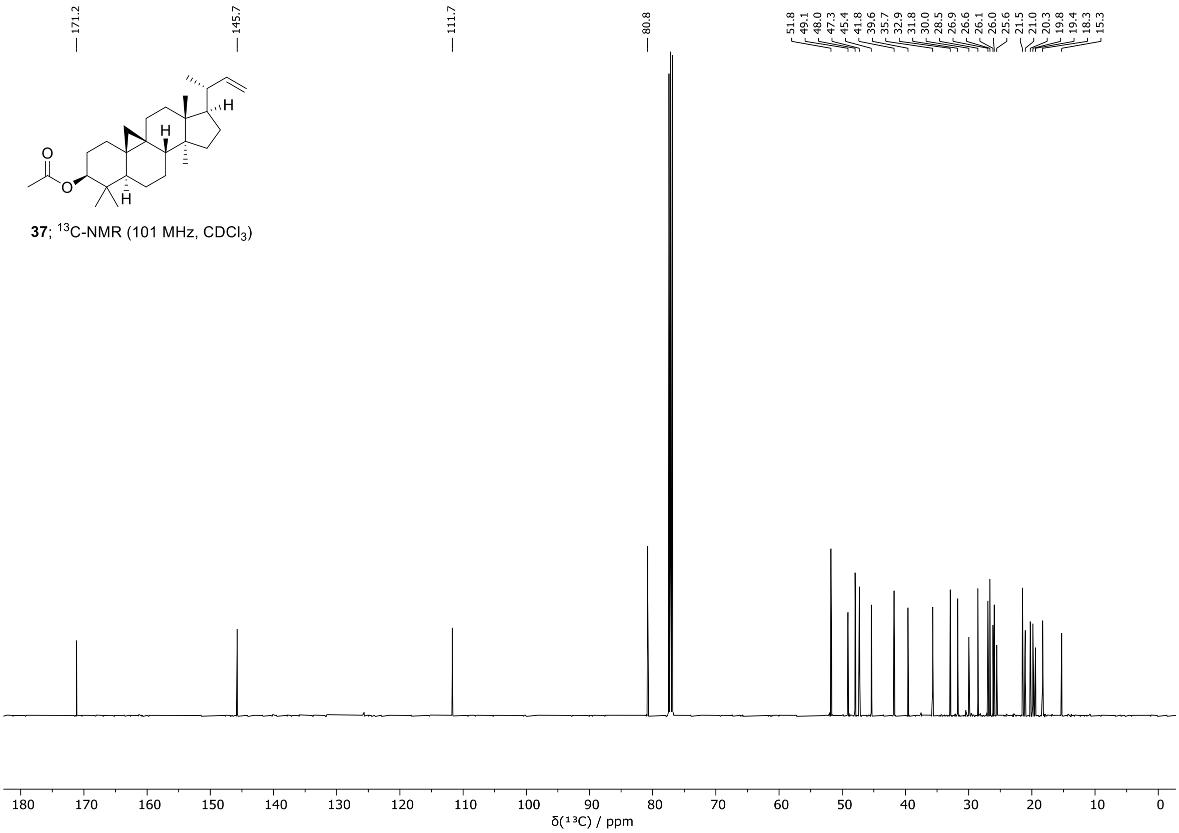
37; ¹³C-NMR (101 MHz, CDCl₃)
171.2
145.7
111.7
80.8
51.8 49.1 48.0 47.3 45.4 41.8 39.6 35.7 32.9 31.8 30.0 28.5 26.9 26.6 26.1 26.0 25.6 21.5 21.0 20.3 19.8 19.4 18.3 15.3
δ(¹³C) / ppm

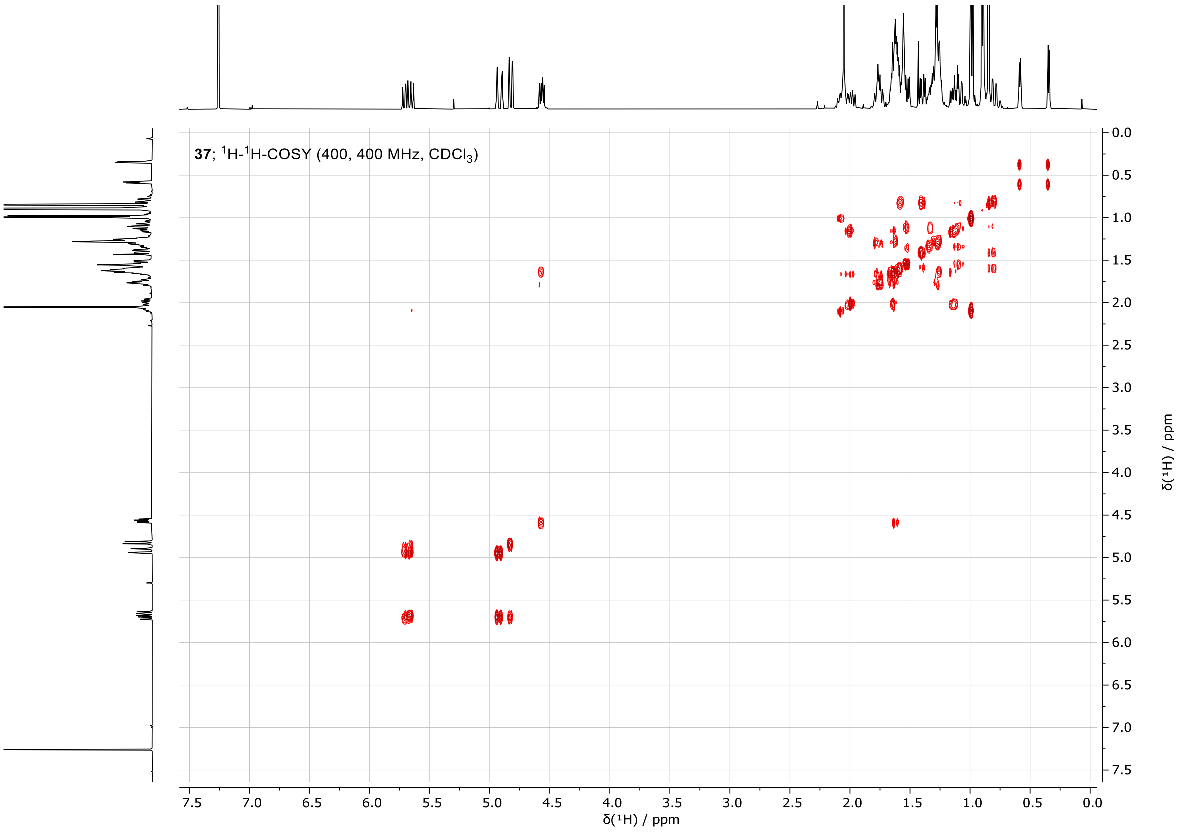
37; ¹H-¹H-COSY (400, 400 MHz, CDCl₃)
δ(¹H) / ppm
δ(¹H) / ppm

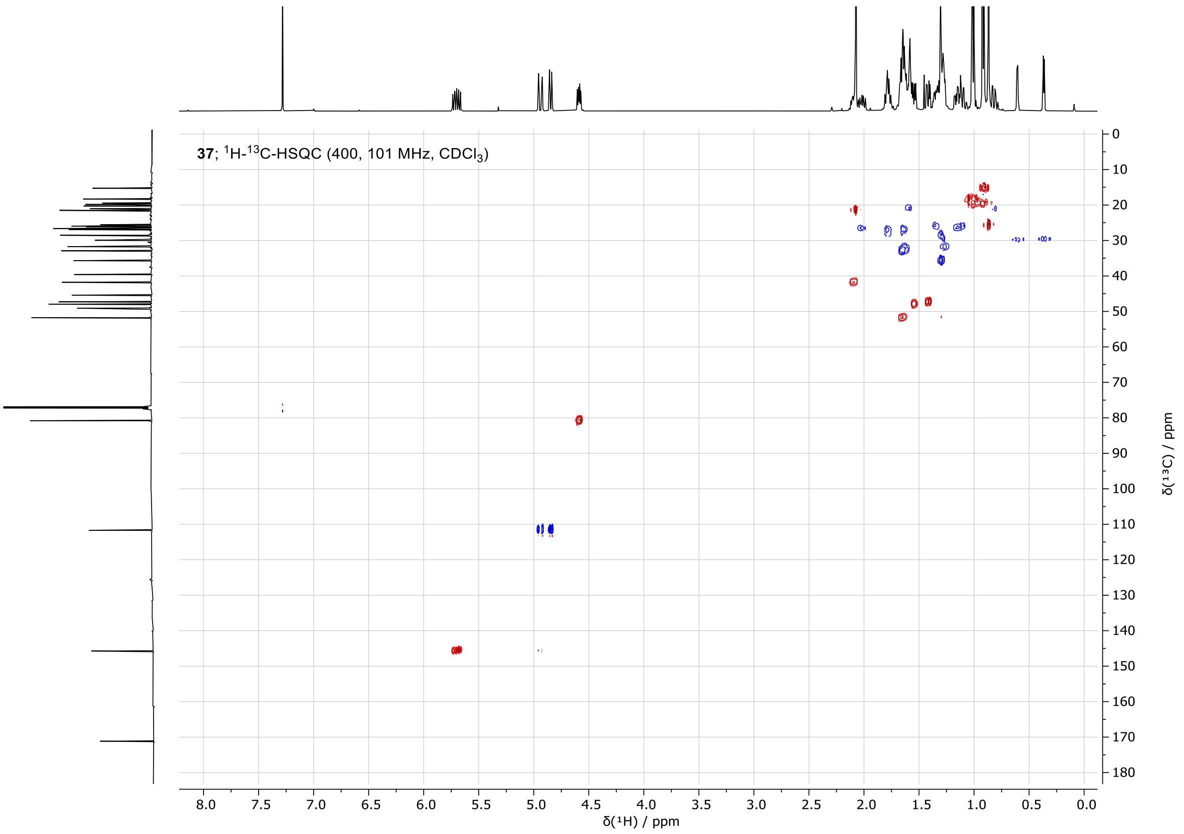
37; ¹H-¹³C-HSQC (400, 101 MHz, CDCl₃)
δ(¹³C) / ppm
δ(¹H) / ppm

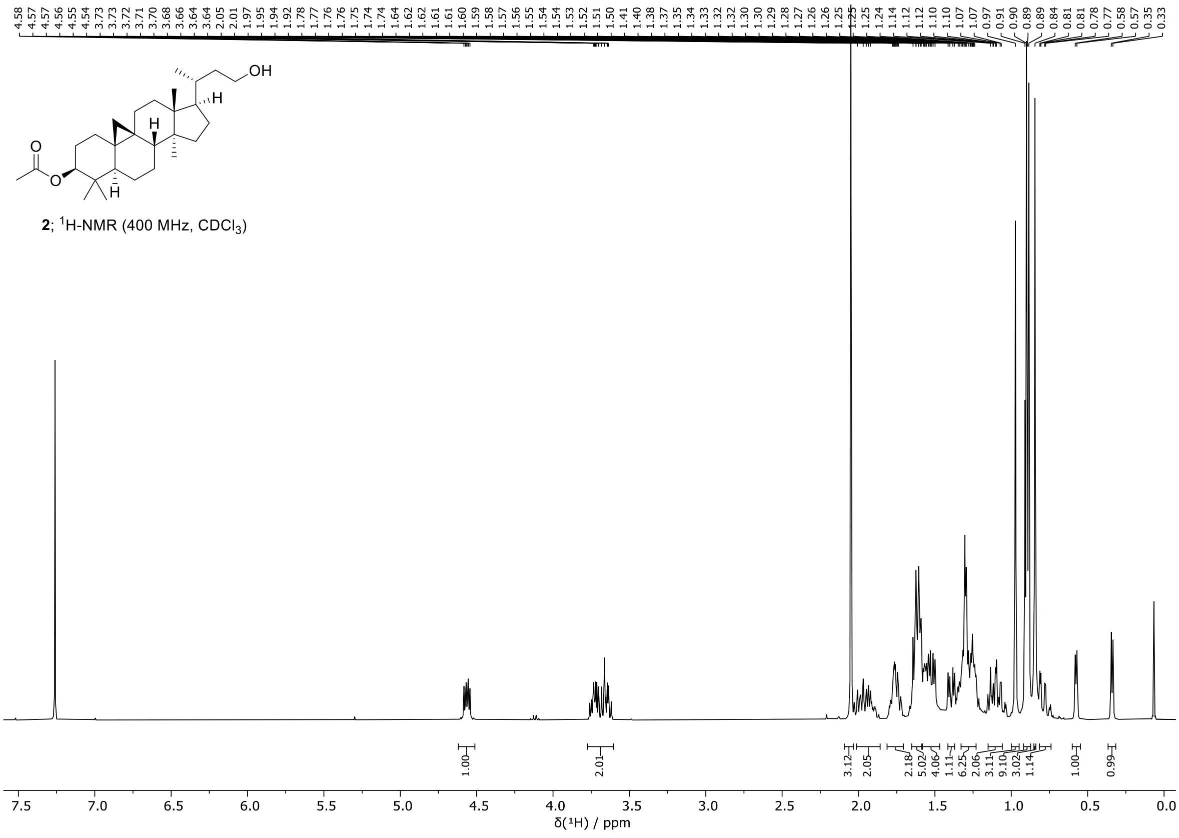
4.58 4.57 4.57 4.56 4.55 4.54 3.73 3.73 3.72 3.71 3.70 3.68 3.66 3.64 3.64 2.05 2.01 1.97 1.95 1.94 1.92 1.78 1.77 1.76 1.76 1.75 1.74 1.74 1.64 1.62 1.62 1.61 1.61 1.60 1.59 1.58 1.56 1.55 1.54 1.54 1.53 1.52 1.51 1.50 1.41 1.40 1.38 1.37 1.35 1.34 1.33 1.32 1.32 1.30 1.30 1.29 1.28 1.27 1.26 1.26 1.25 1.25 1.25 1.24 1.14 1.12 1.12 1.10 1.10 1.07 1.07 0.97 0.91 0.90 0.89 0.89 0.84 0.81 0.81 0.78 0.77 0.58 0.57 0.35 0.33
OH
H
H
O
O
H
2; ¹H-NMR (400 MHz, CDCl₃)
1.00 2.01 3.12 2.05 2.18 5.02 4.06 1.11 6.25 2.06 3.11 9.10 3.02 1.14 1.00 0.99
δ(¹H) / ppm

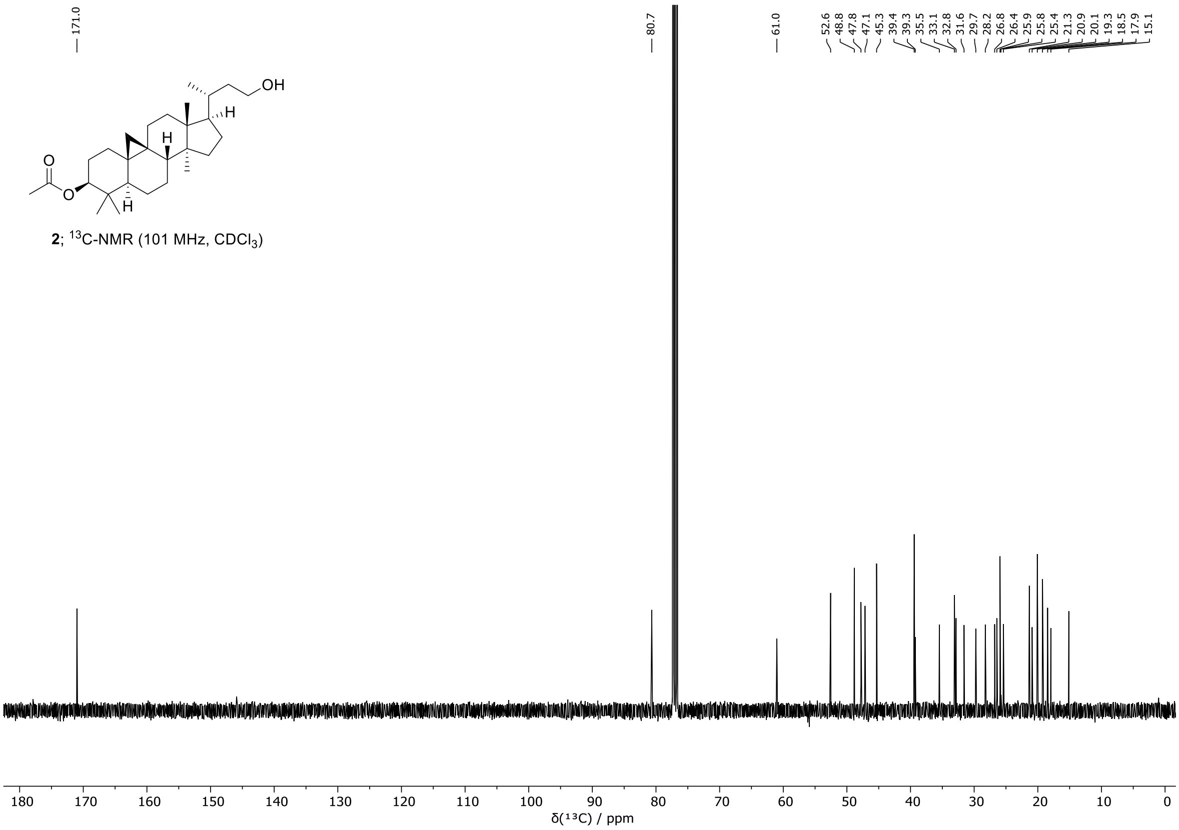
171.0
80.7
61.0
52.6
48.8
47.8
47.1
45.3
39.4
39.3
35.5
33.1
32.8
31.6
29.7
28.2
26.8
26.4
25.9
25.8
25.4
21.3
20.9
20.1
19.3
18.5
17.9
15.1
OH
H
H
O
O
H
2; 13C-NMR (101 MHz, CDCl3)
δ(13C) / ppm

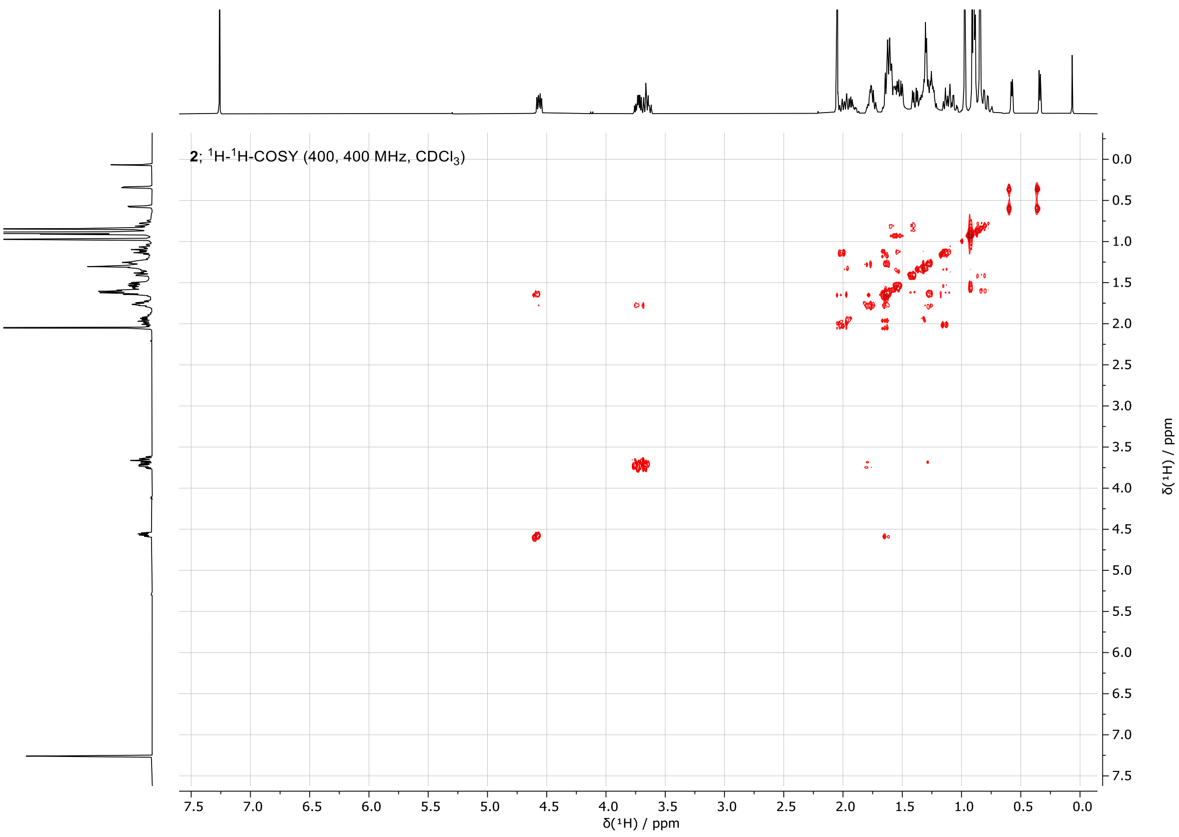
2; 1H-1H-COSY (400, 400 MHz, CDCl3)
δ(1H) / ppm
δ(1H) / ppm

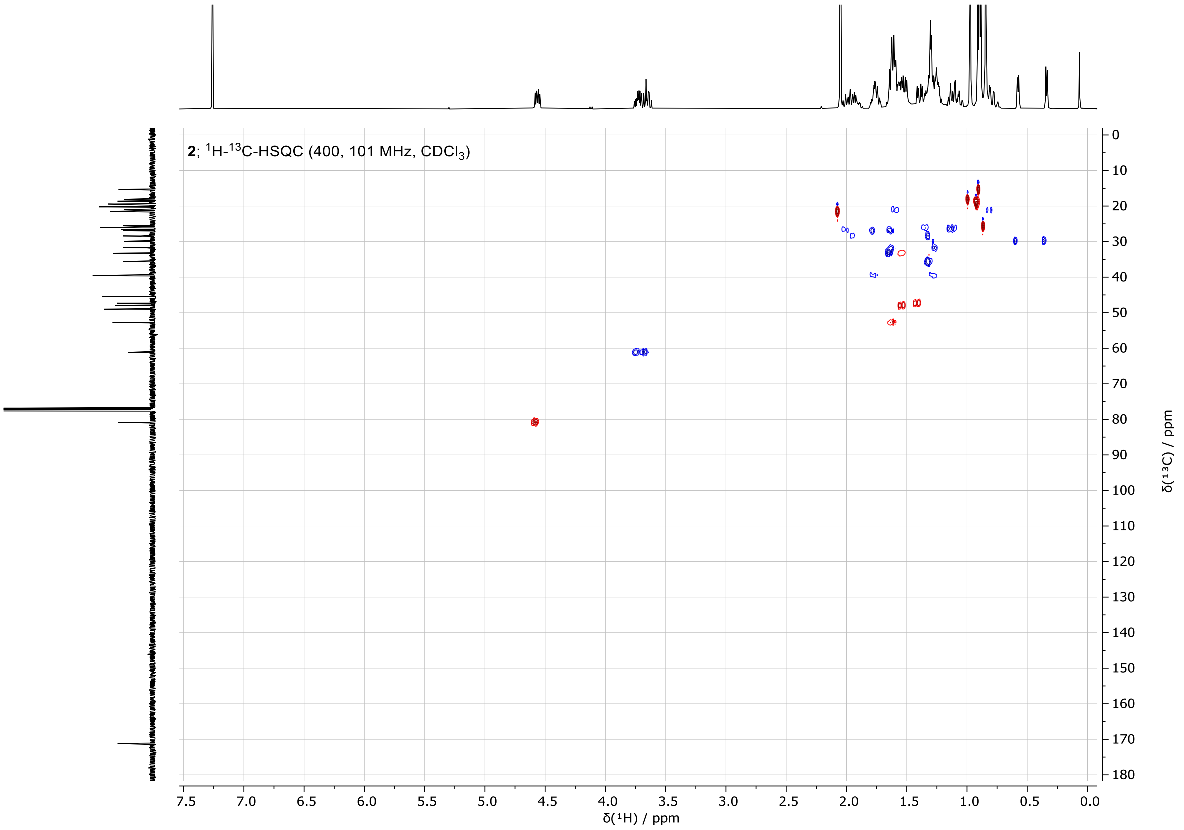
2; ¹H-¹³C-HSQC (400, 101 MHz, CDCl₃)
δ(¹H) / ppm
δ(¹³C) / ppm

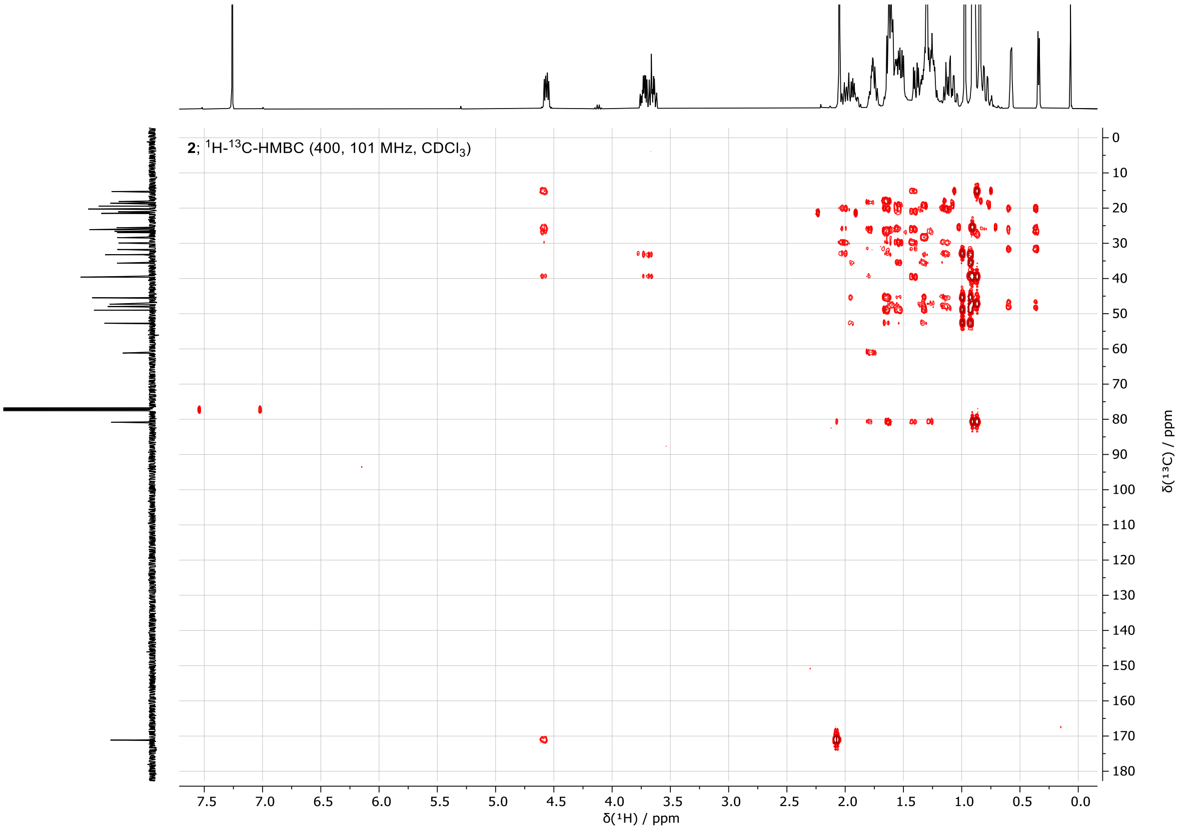
2; ¹H-¹³C-HMBC (400, 101 MHz, CDCl₃)
δ(¹H) / ppm
δ(¹³C) / ppm

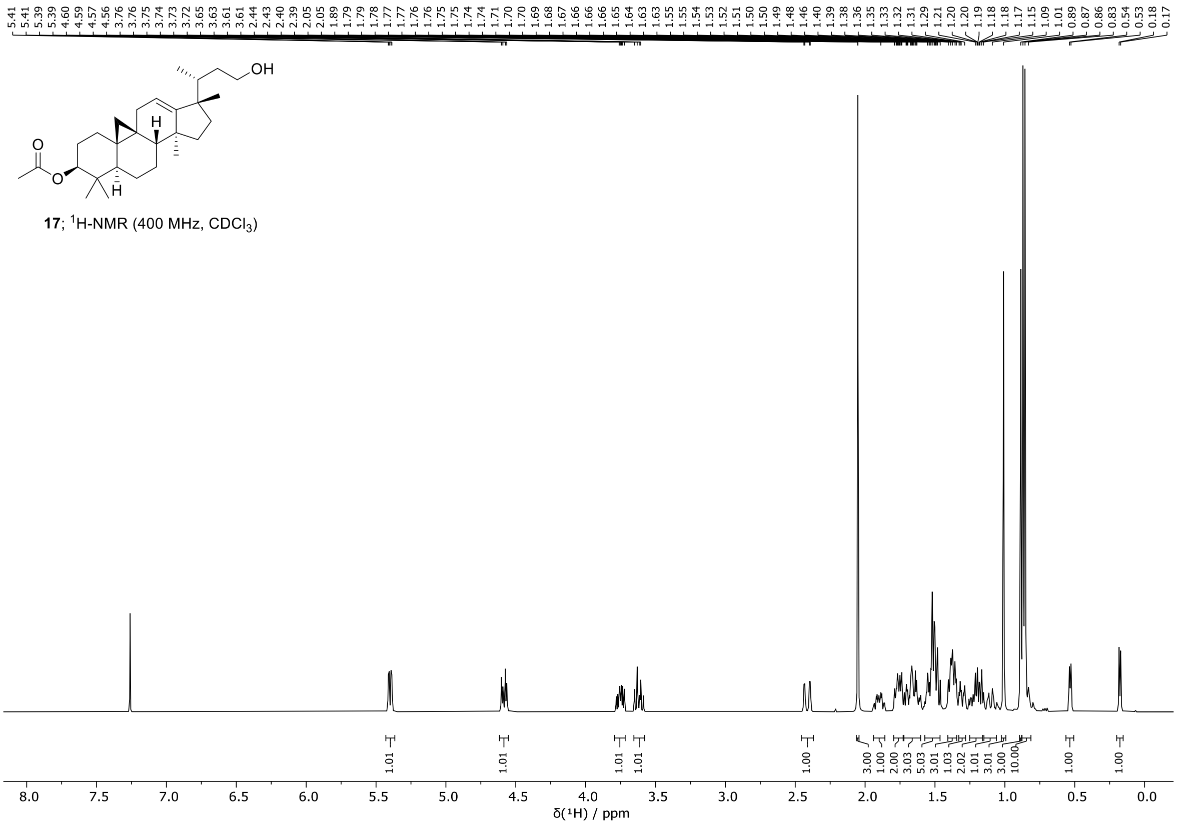

17; ^{1}H-NMR (400 MHz, $CDCl_3$)

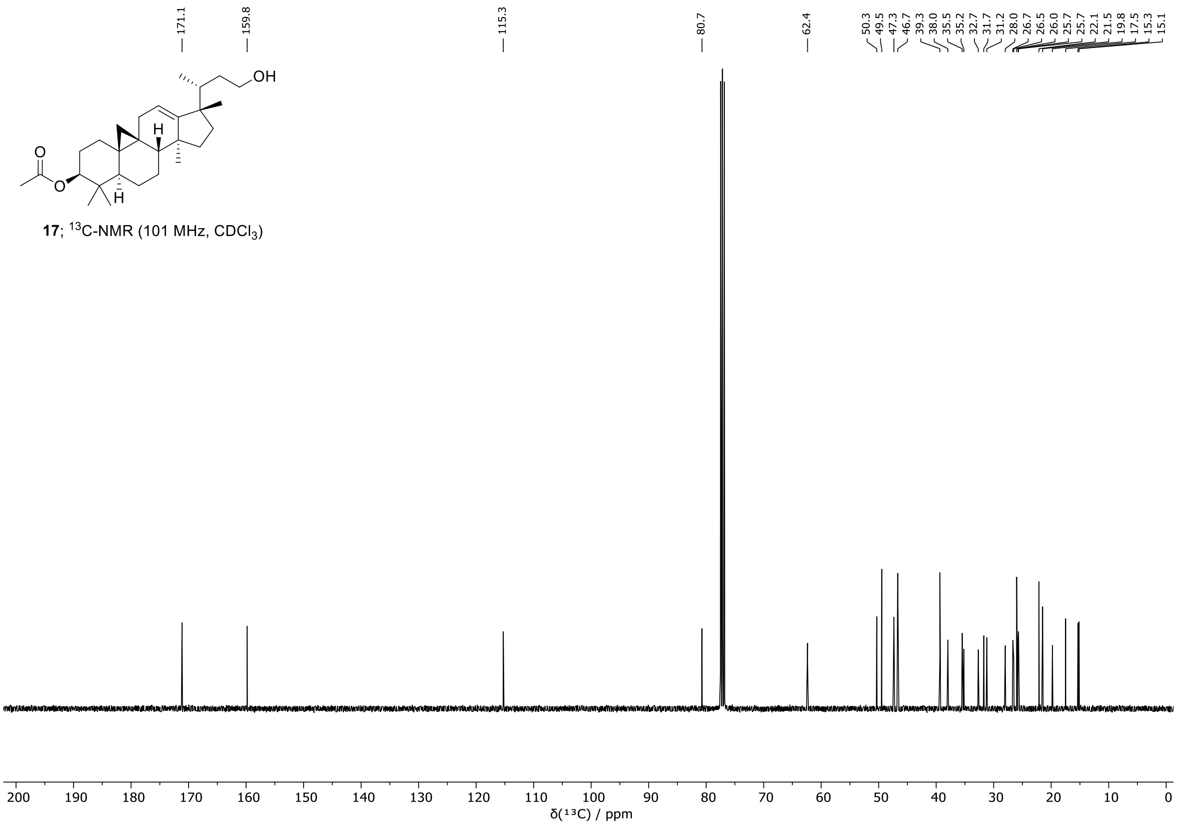

17; ^{13}C-NMR (101 MHz, $CDCl_3$)

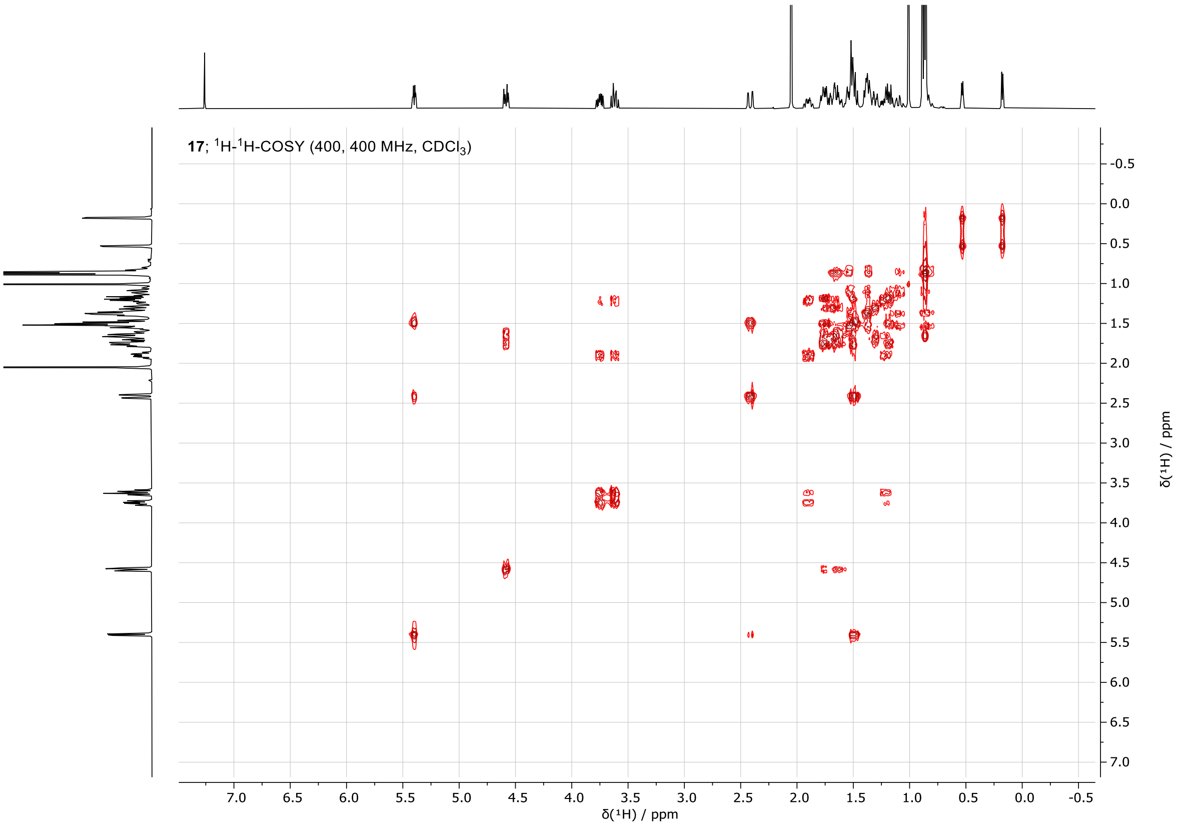
17; 1H-1H-COSY (400, 400 MHz, CDCl3)
δ(¹H) / ppm
δ(¹H) / ppm

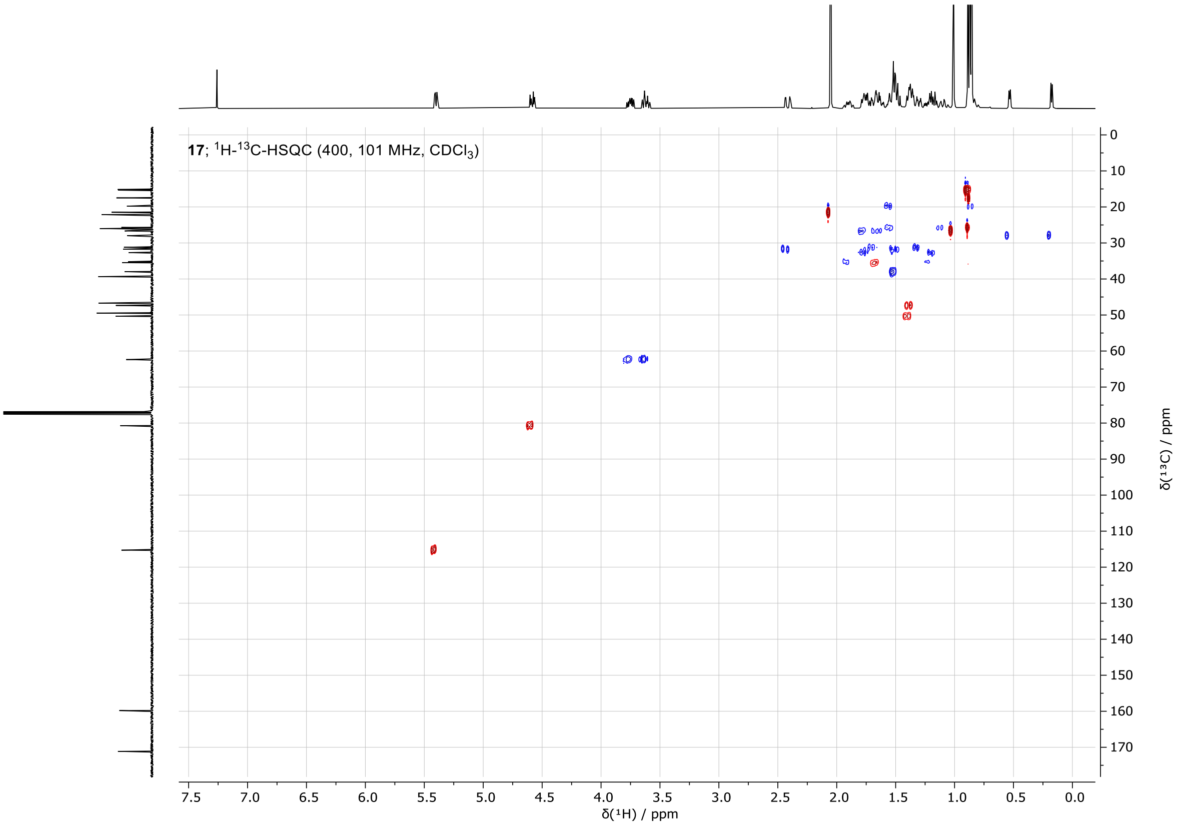
17; 1H-13C-HSQC (400, 101 MHz, CDCl3)
δ(¹H) / ppm
δ(¹³C) / ppm

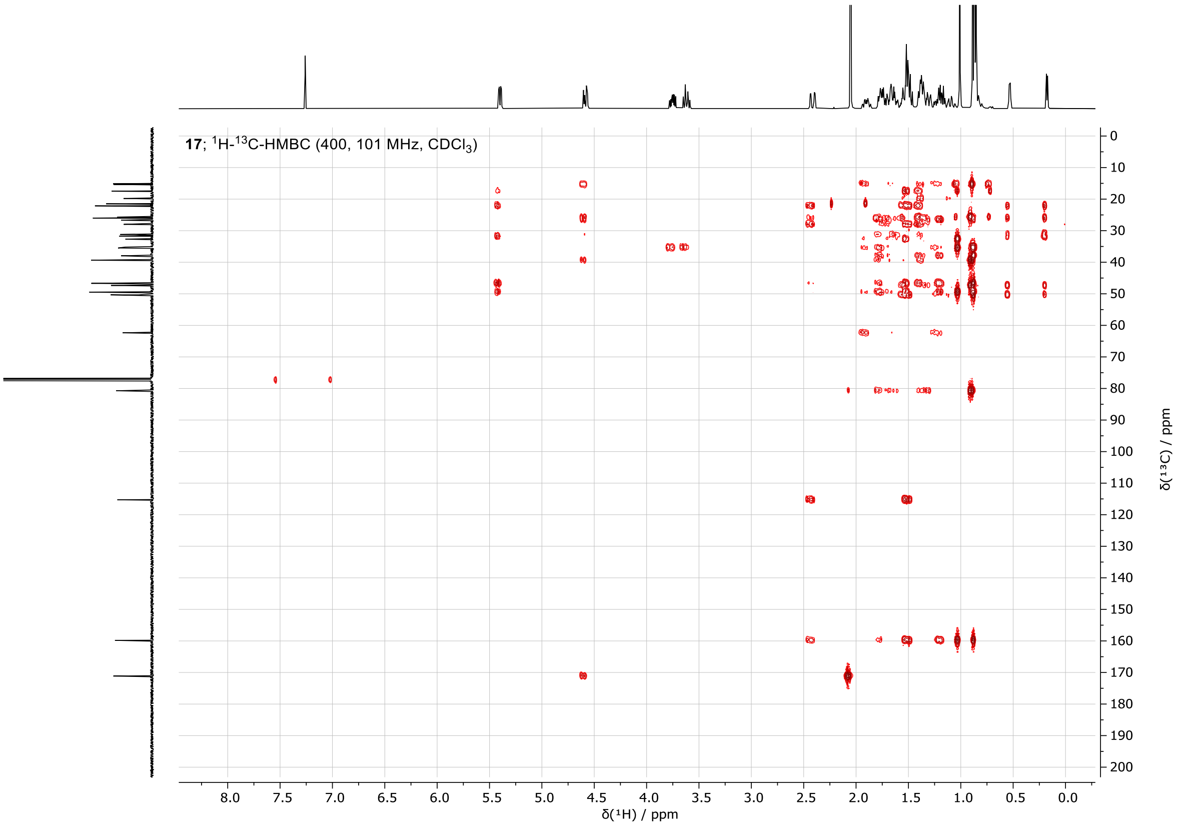

17; ^{1}H-^{13}C-HMBC (400, 101 MHz, $CDCl_3$)

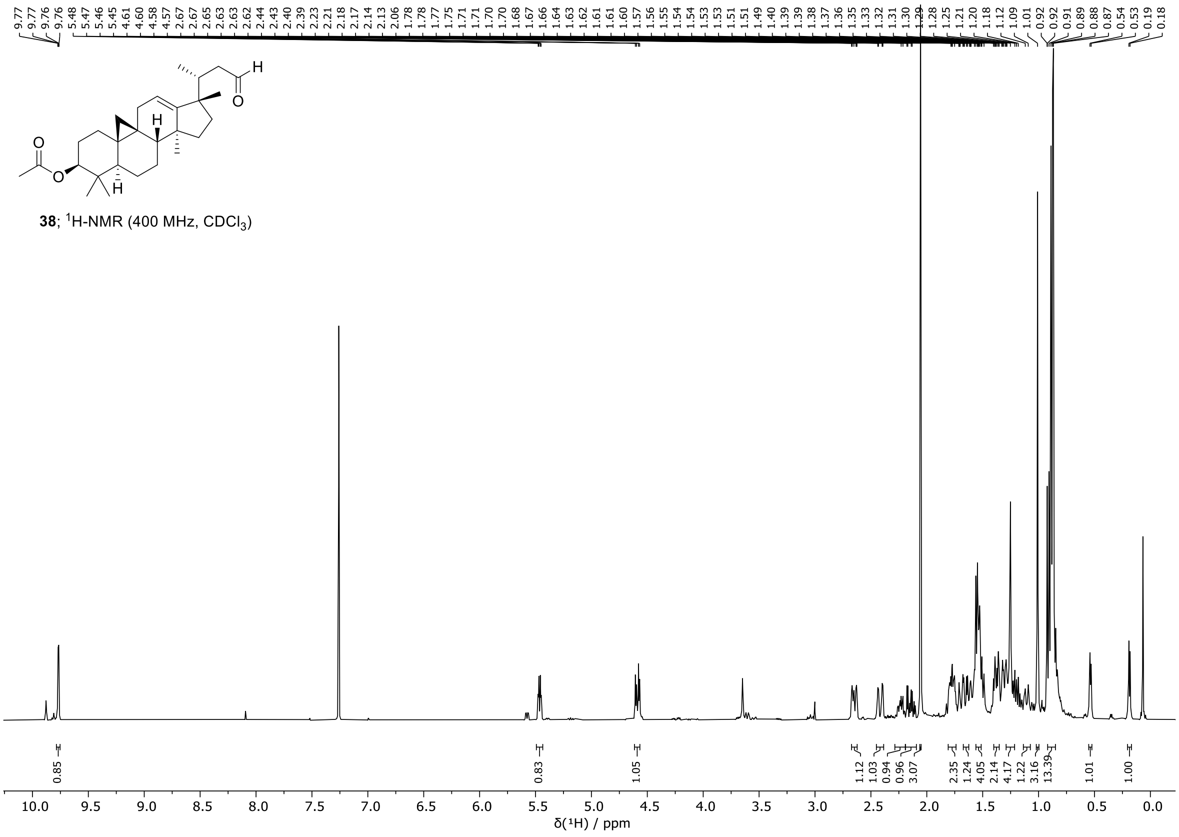

38; ^{1}H-NMR (400 MHz, $CDCl_3$)

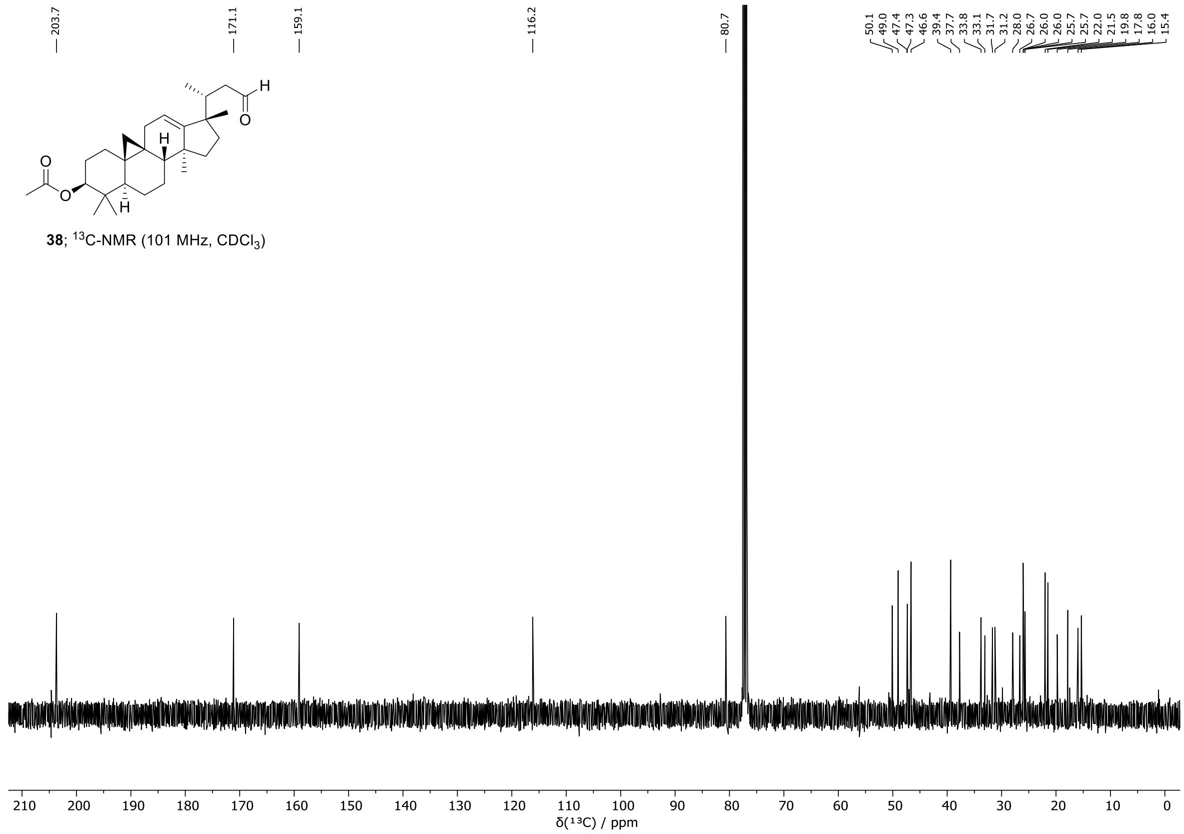

38; ^{13}C-NMR (101 MHz, CDCl$_3$)

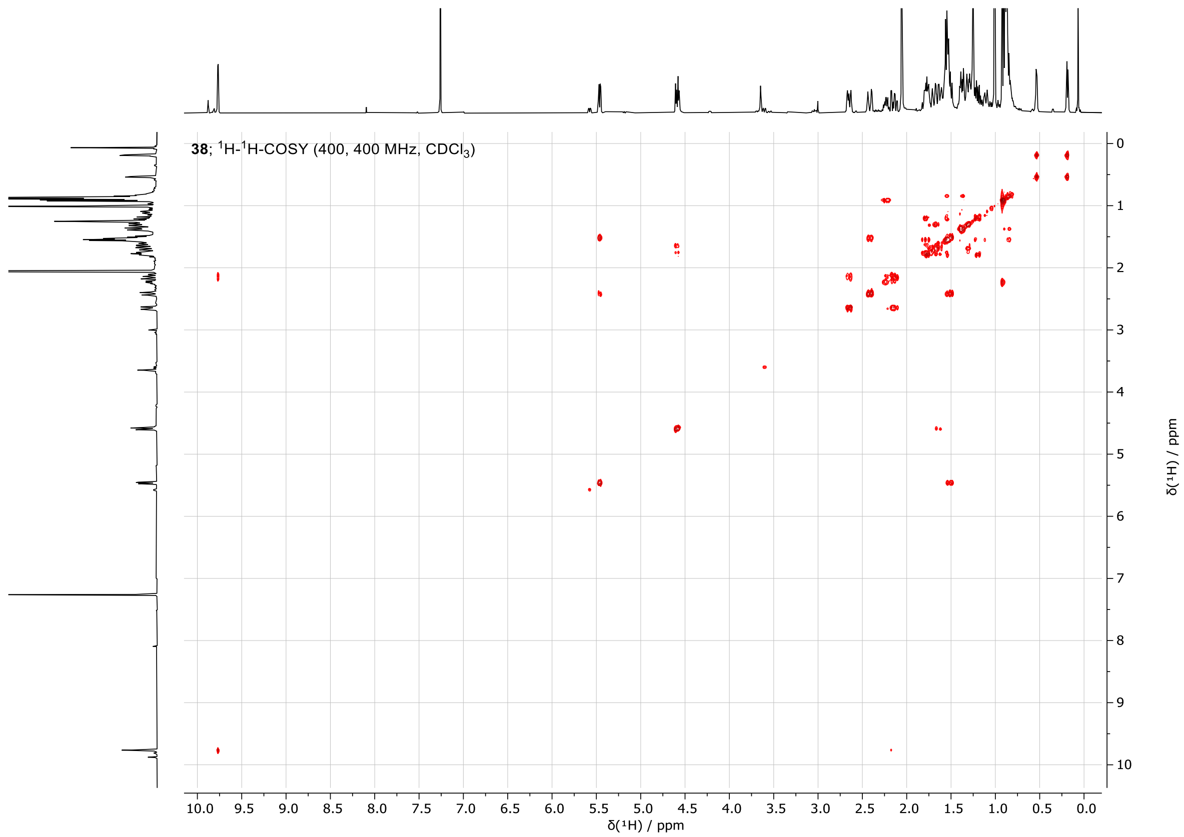

38; ^{1}H-^{1}H-COSY (400, 400 MHz, CDCl$_3$)

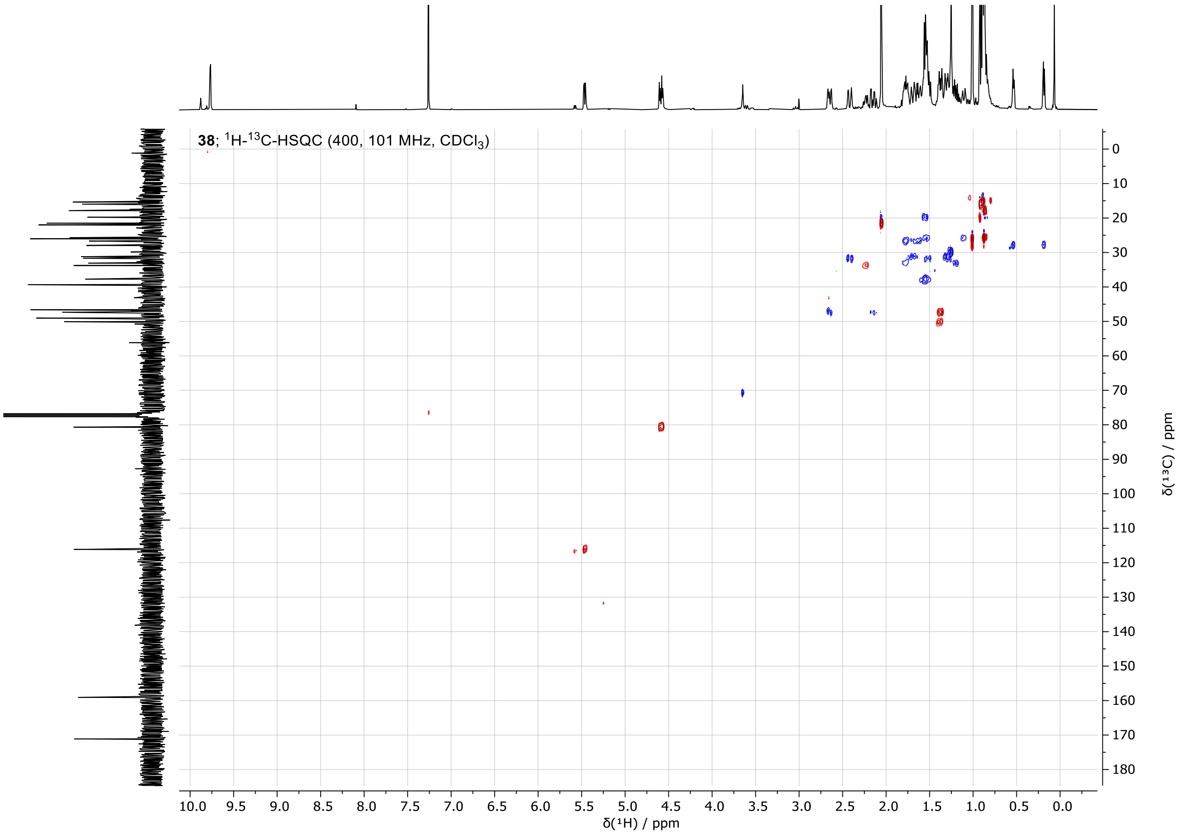

38; ¹H-¹³C-HSQC (400, 101 MHz, CDCl₃)
δ(¹H) / ppm
δ(¹³C) / ppm

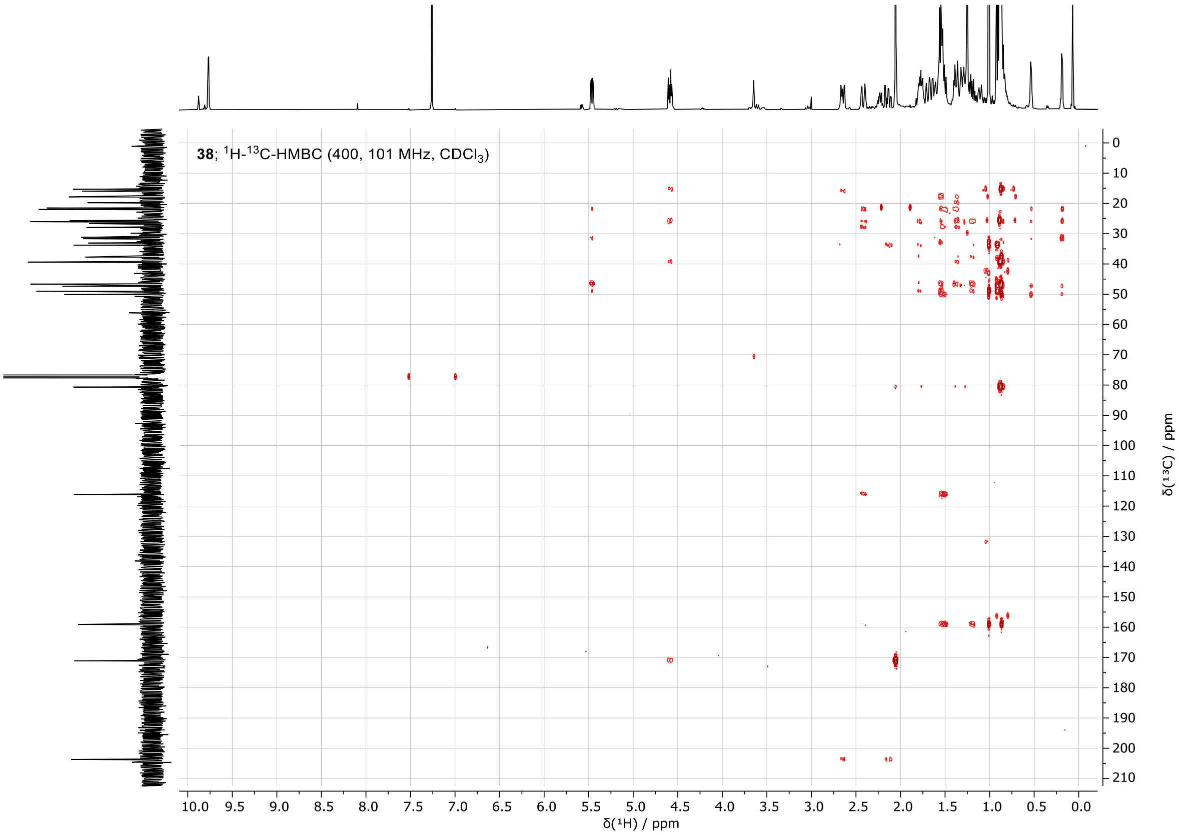

38; ¹H-¹³C-HMBC (400, 101 MHz, CDCl₃)
δ(¹H) / ppm
δ(¹³C) / ppm

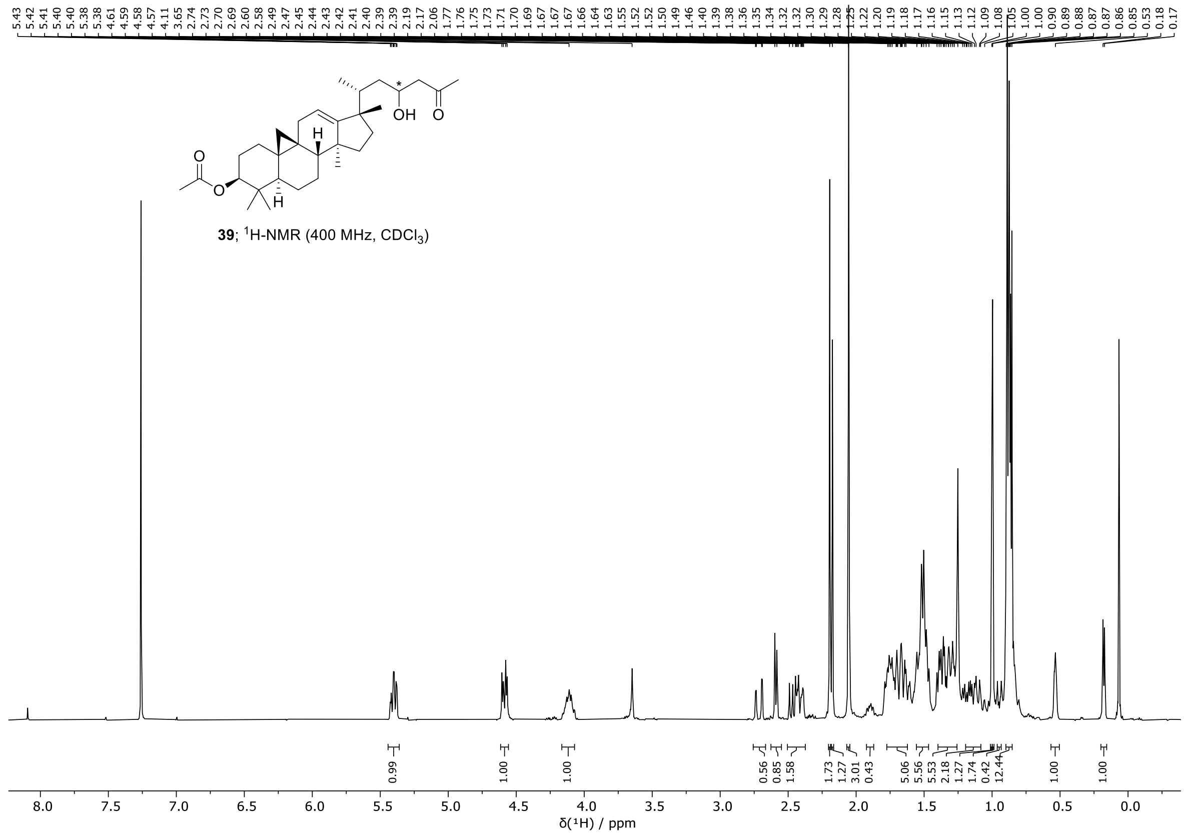
39; ^{1}H-NMR (400 MHz, $CDCl_3$)
δ(¹H) / ppm

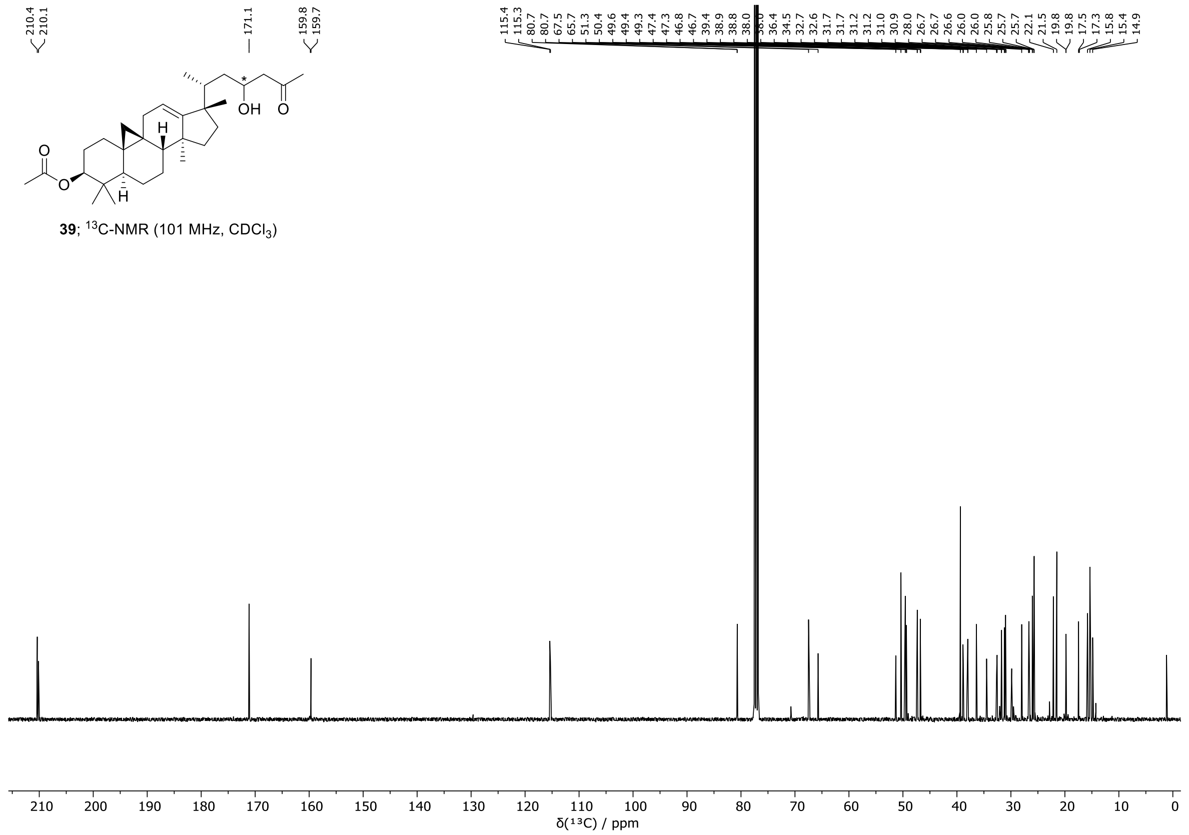
39; ^{13}C-NMR (101 MHz, $CDCl_3$)
δ(¹³C) / ppm

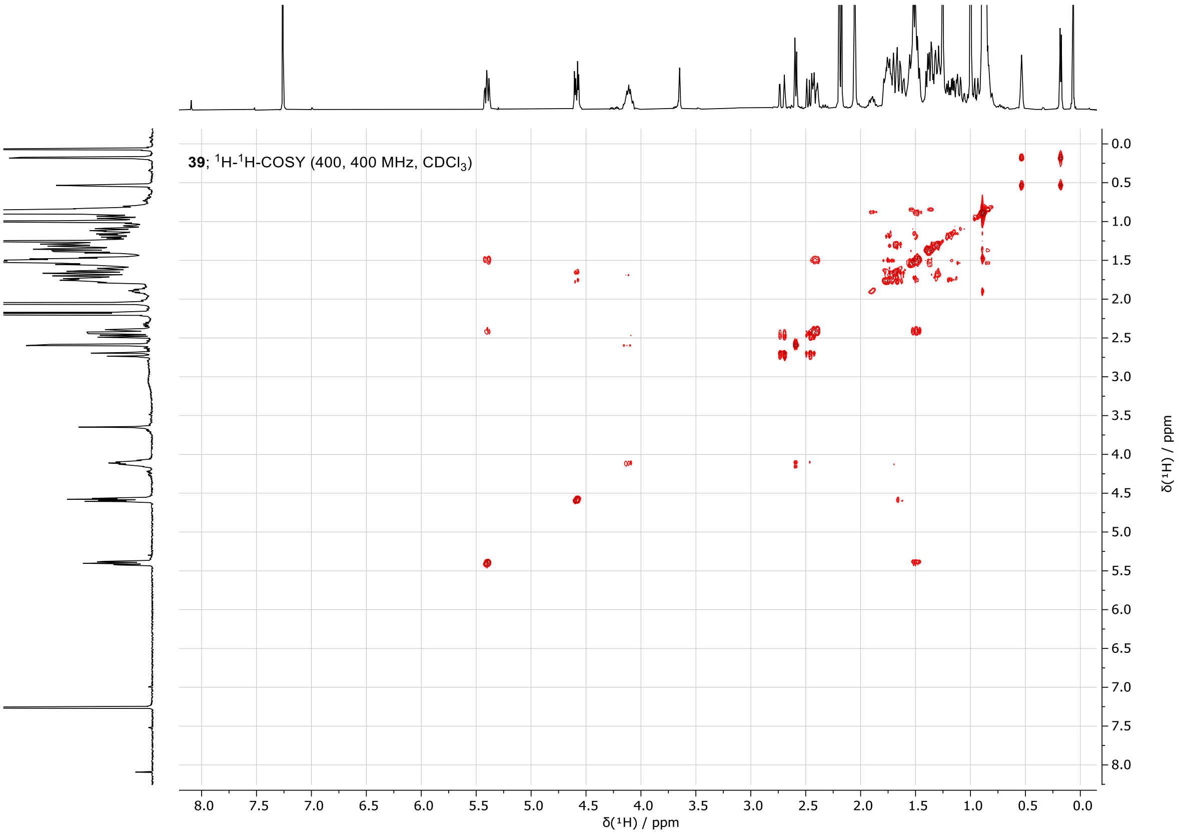

39; ¹H-¹H-COSY (400, 400 MHz, CDCl₃)
δ(¹H) / ppm
δ(¹H) / ppm

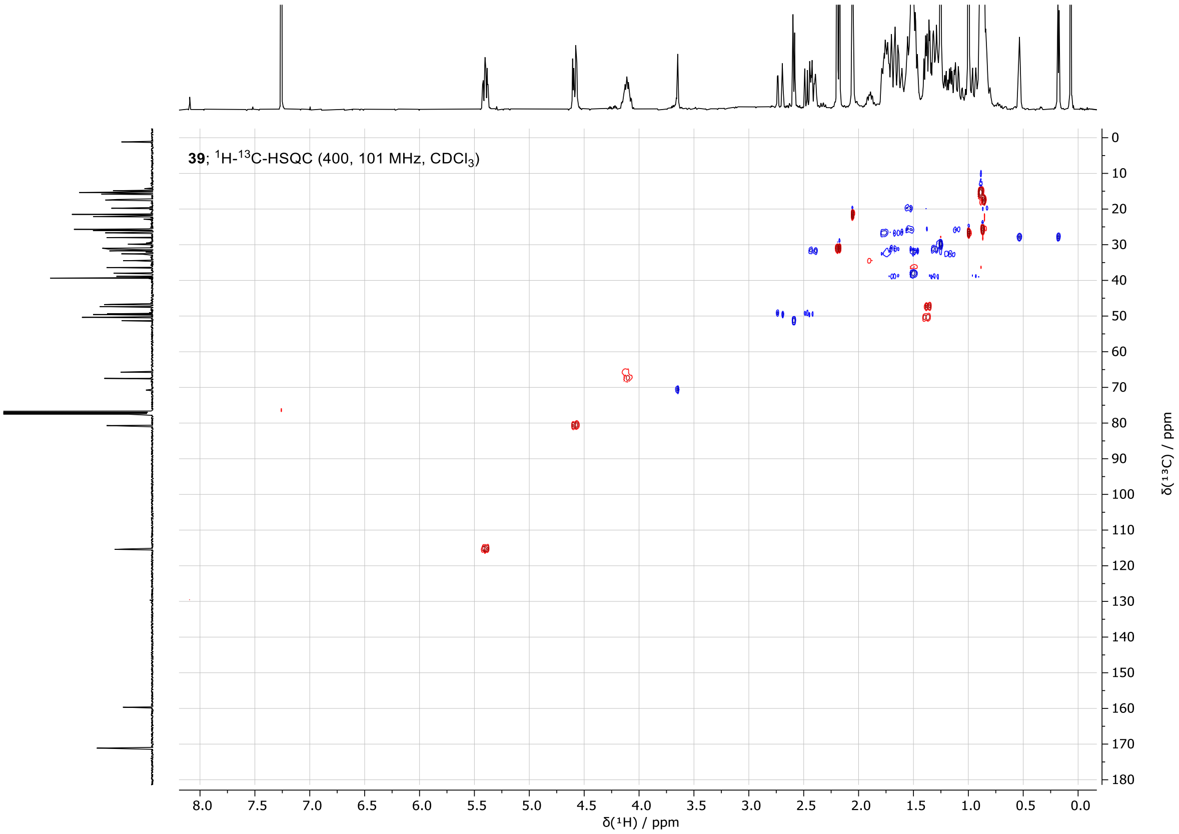

39; ¹H-¹³C-HSQC (400, 101 MHz, CDCl₃)
δ(¹H) / ppm
δ(¹³C) / ppm

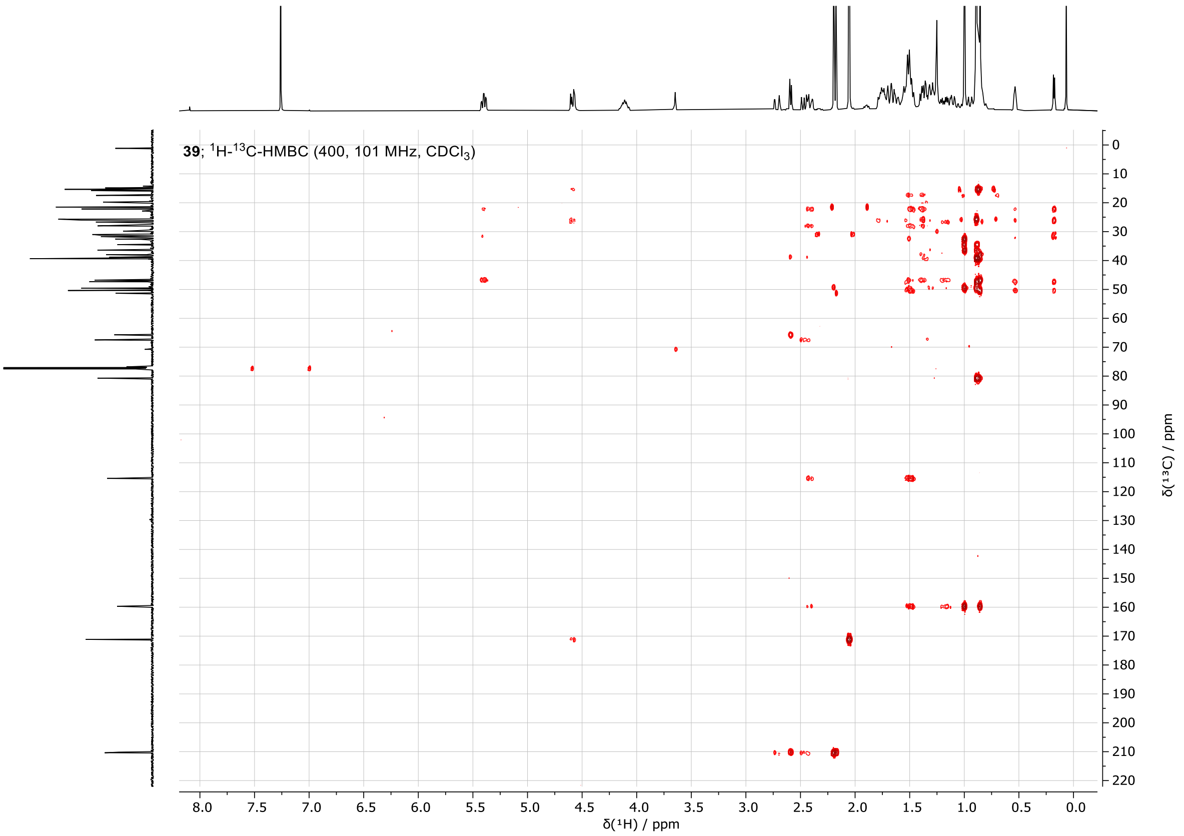

39; ¹H-¹³C-HMBC (400, 101 MHz, CDCl₃)
δ(¹H) / ppm
δ(¹³C) / ppm
8.0 7.5 7.0 6.5 6.0 5.5 5.0 4.5 4.0 3.5 3.0 2.5 2.0 1.5 1.0 0.5 0.0
0 10 20 30 40 50 60 70 80 90 100 110 120 130 140 150 160 170 180 190 200 210 220

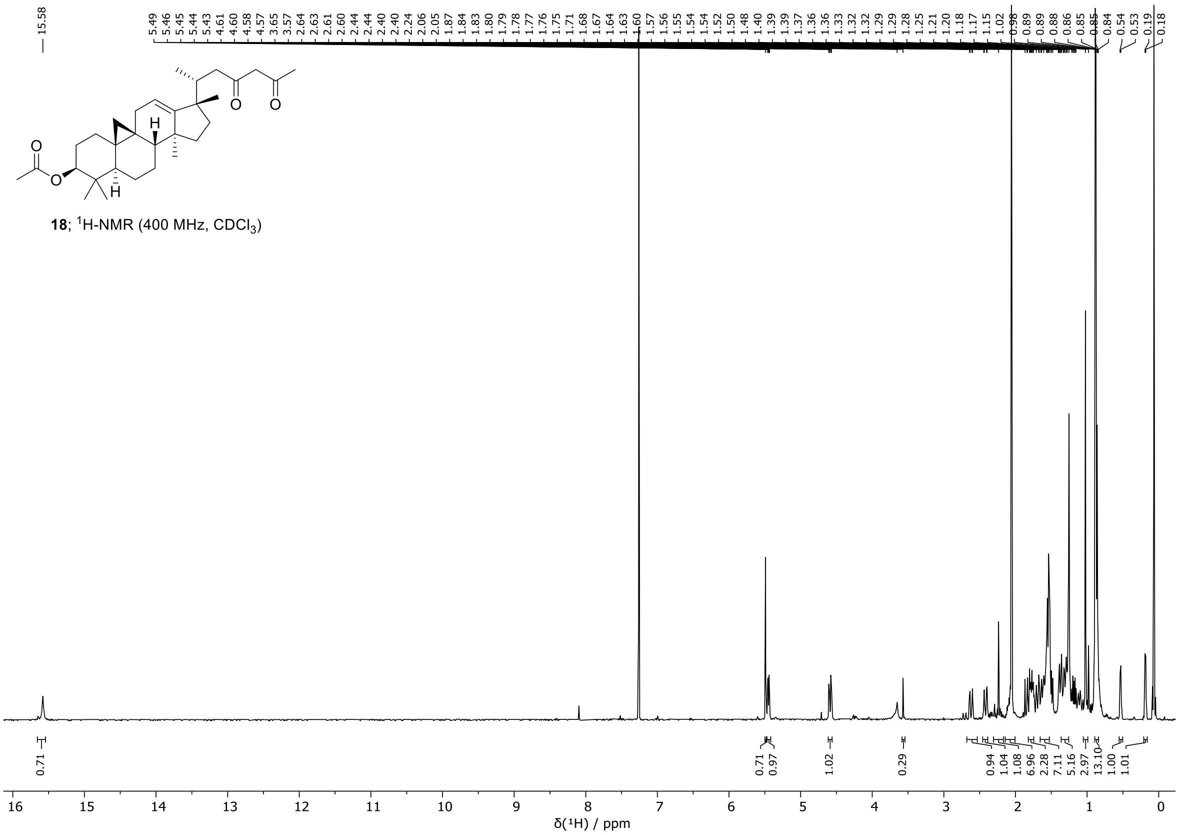

15.58
5.49 5.46 5.45 5.44 5.43 4.61 4.60 4.58 4.57 3.65 3.57 2.64 2.63 2.61 2.60 2.44 2.44 2.40 2.40 2.24 2.06 2.05 1.87 1.84 1.83 1.80 1.79 1.78 1.77 1.76 1.75 1.71 1.68 1.67 1.64 1.63 1.60 1.57 1.56 1.55 1.54 1.54 1.52 1.50 1.48 1.40 1.39 1.39 1.37 1.36 1.33 1.32 1.32 1.29 1.28 1.25 1.21 1.20 1.18 1.17 1.15 1.02 [illegible] 0.89 0.89 0.88 0.86 0.85 0.85 0.84 0.54 0.53 0.19 0.18
H
H
O
O
O
O
18; ¹H-NMR (400 MHz, CDCl₃)
0.71 0.71 0.97 1.02 0.29 0.94 1.04 1.08 6.96 2.28 7.11 5.16 2.97 13.10 1.00 1.01
16 15 14 13 12 11 10 9 8 7 6 5 4 3 2 1 0
δ(¹H) / ppm

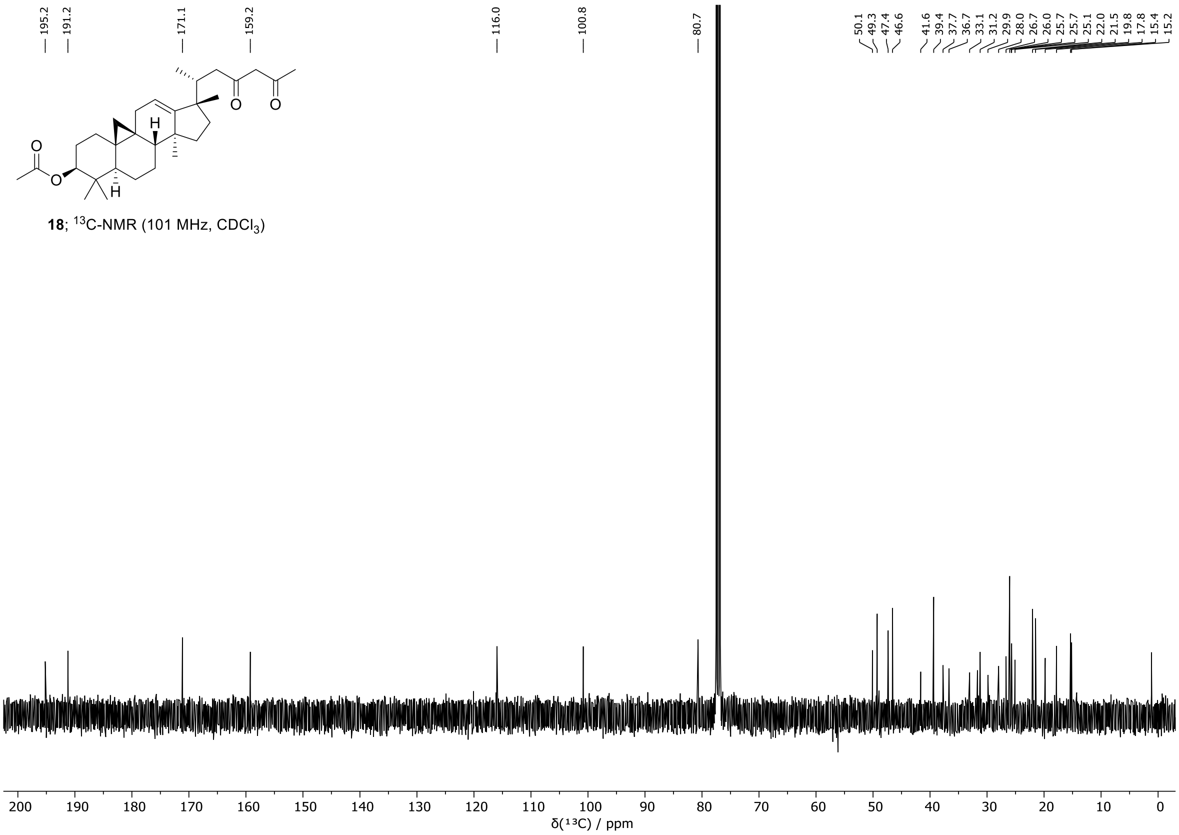

18; ^{13}C-NMR (101 MHz, $CDCl_3$)

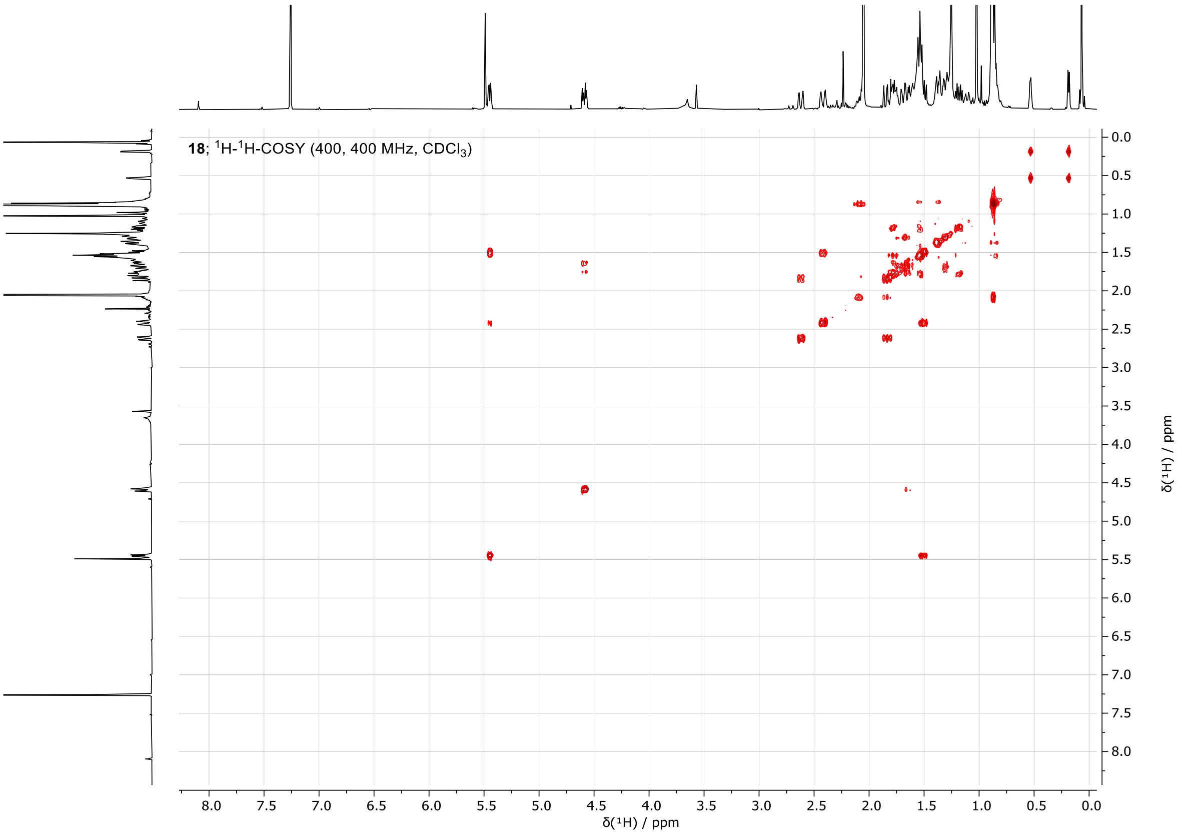

18; ^{1}H-^{1}H-COSY (400, 400 MHz, $CDCl_3$)

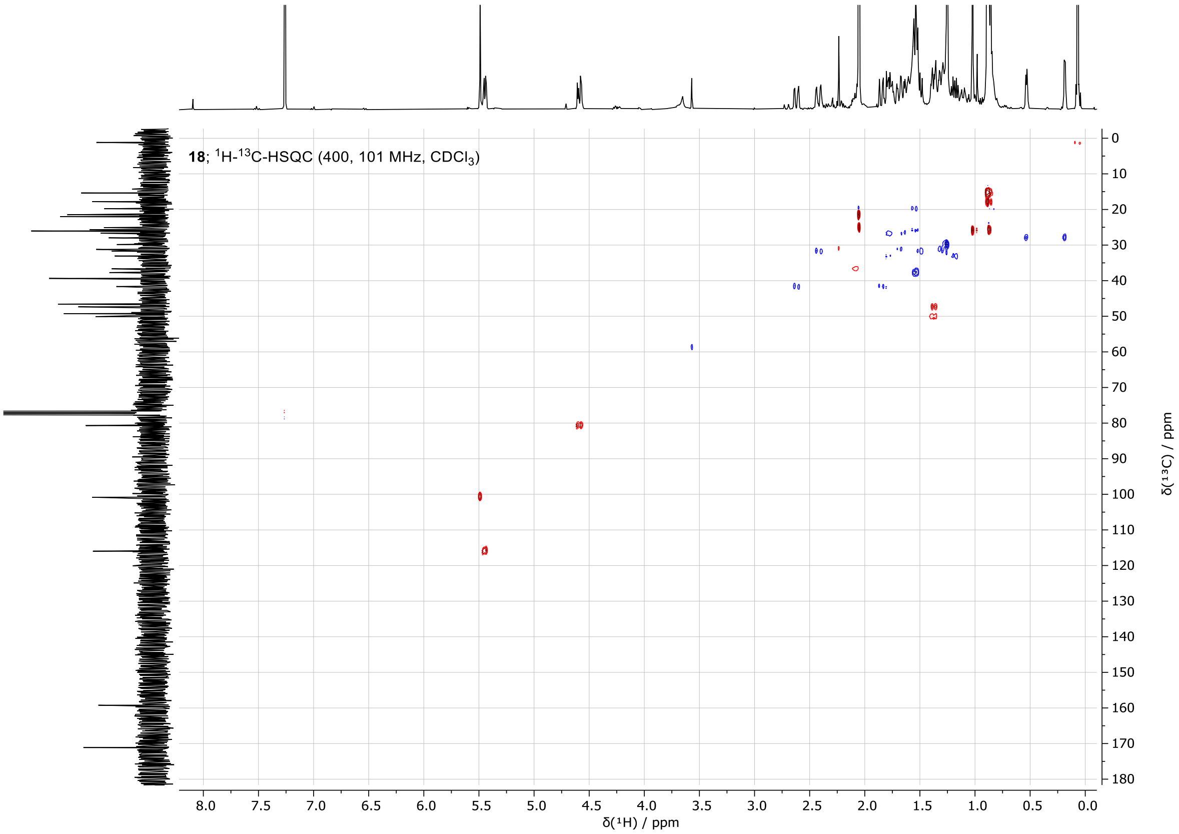
18; ^{1}H-^{13}C-HSQC (400, 101 MHz, $CDCl_3$)
δ(¹H) / ppm
δ(¹³C) / ppm

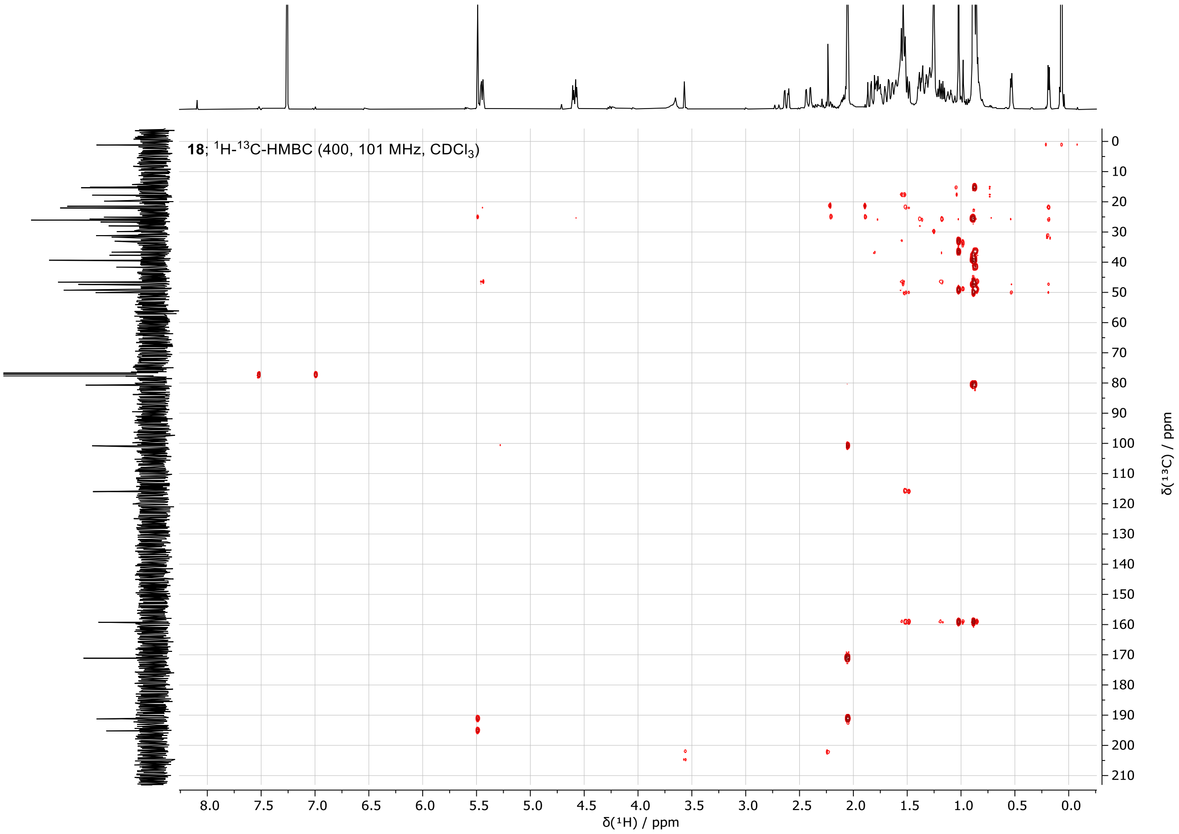
18; ^{1}H-^{13}C-HMBC (400, 101 MHz, $CDCl_3$)
δ(¹H) / ppm
δ(¹³C) / ppm

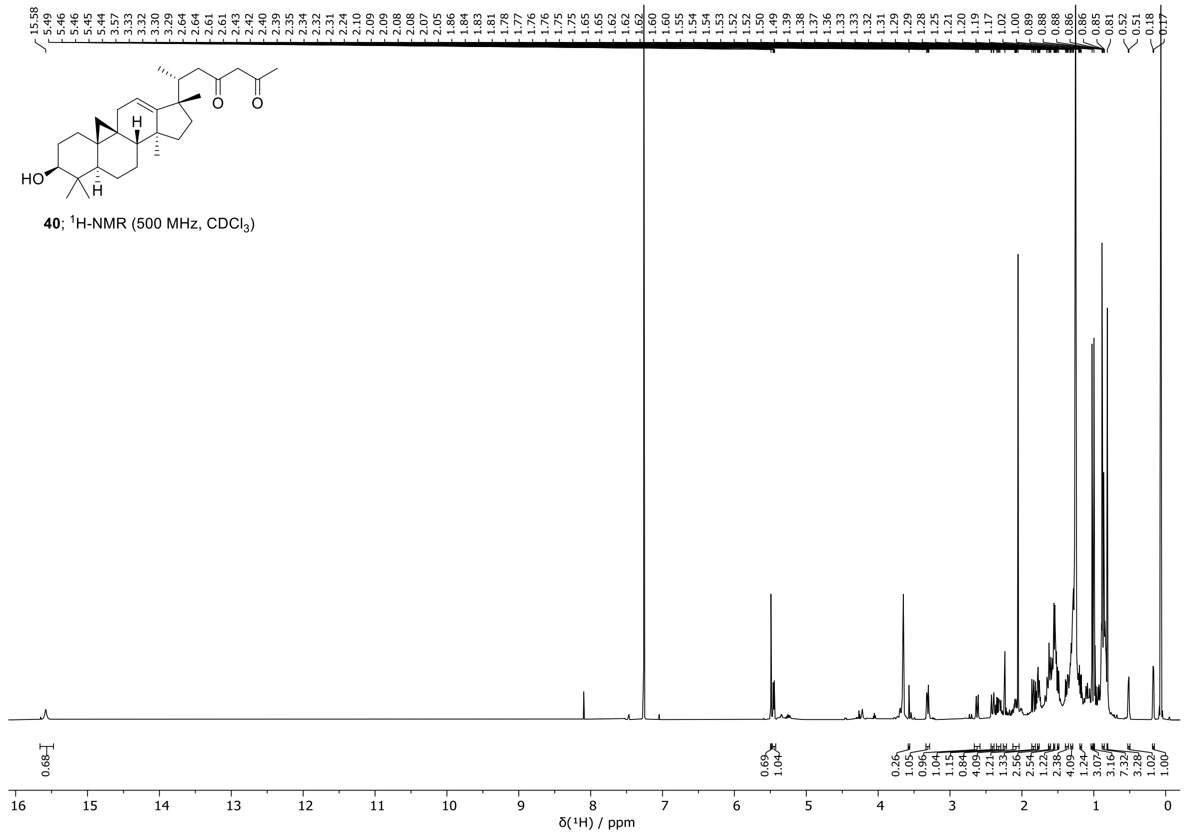

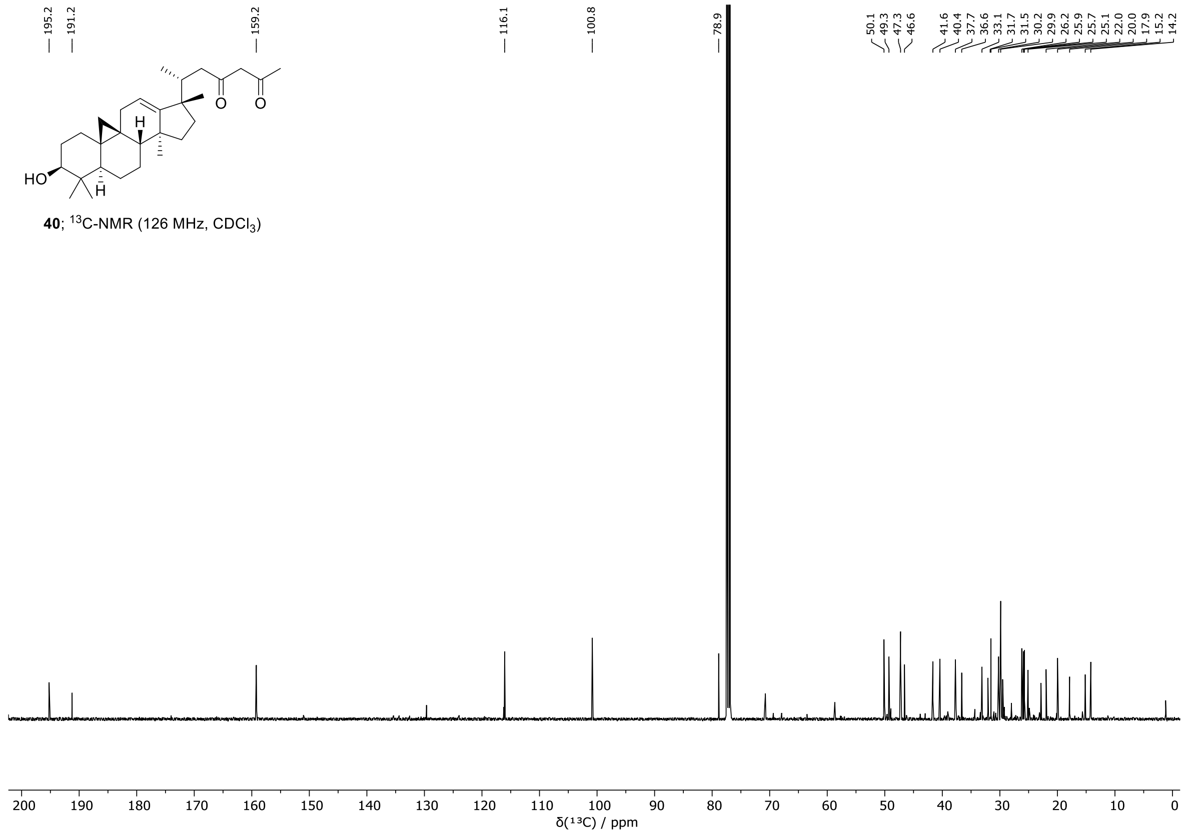

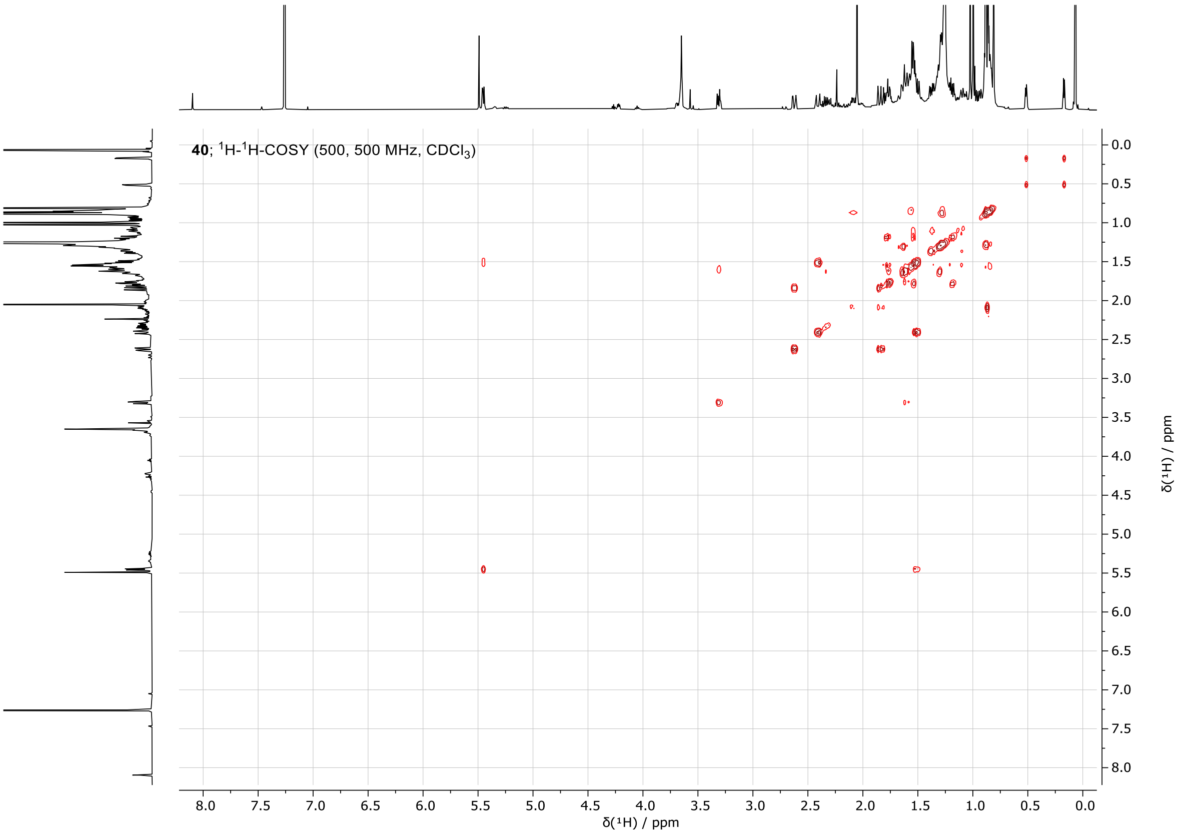
40; ¹H-¹H-COSY (500, 500 MHz, CDCl3)
δ(¹H) / ppm
δ(¹H) / ppm

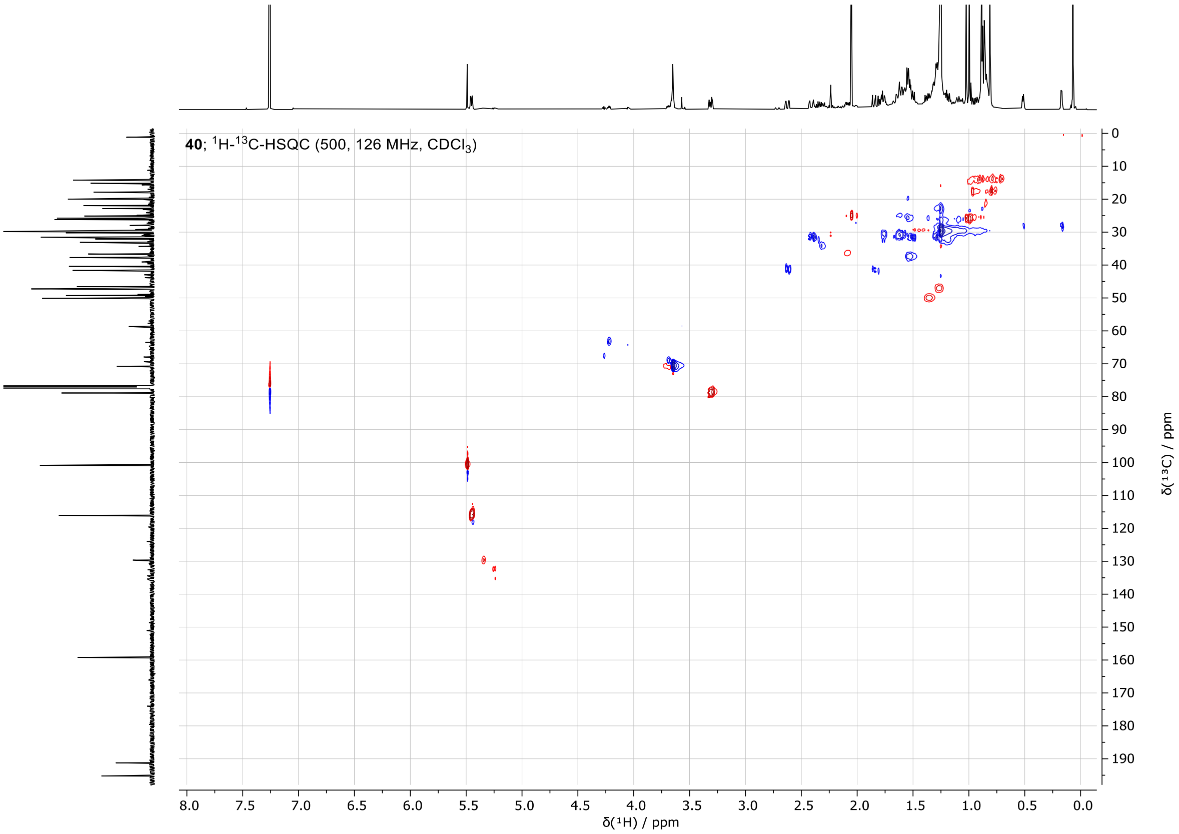
40; ¹H-¹³C-HSQC (500, 126 MHz, CDCl3)
δ(¹H) / ppm
δ(¹³C) / ppm

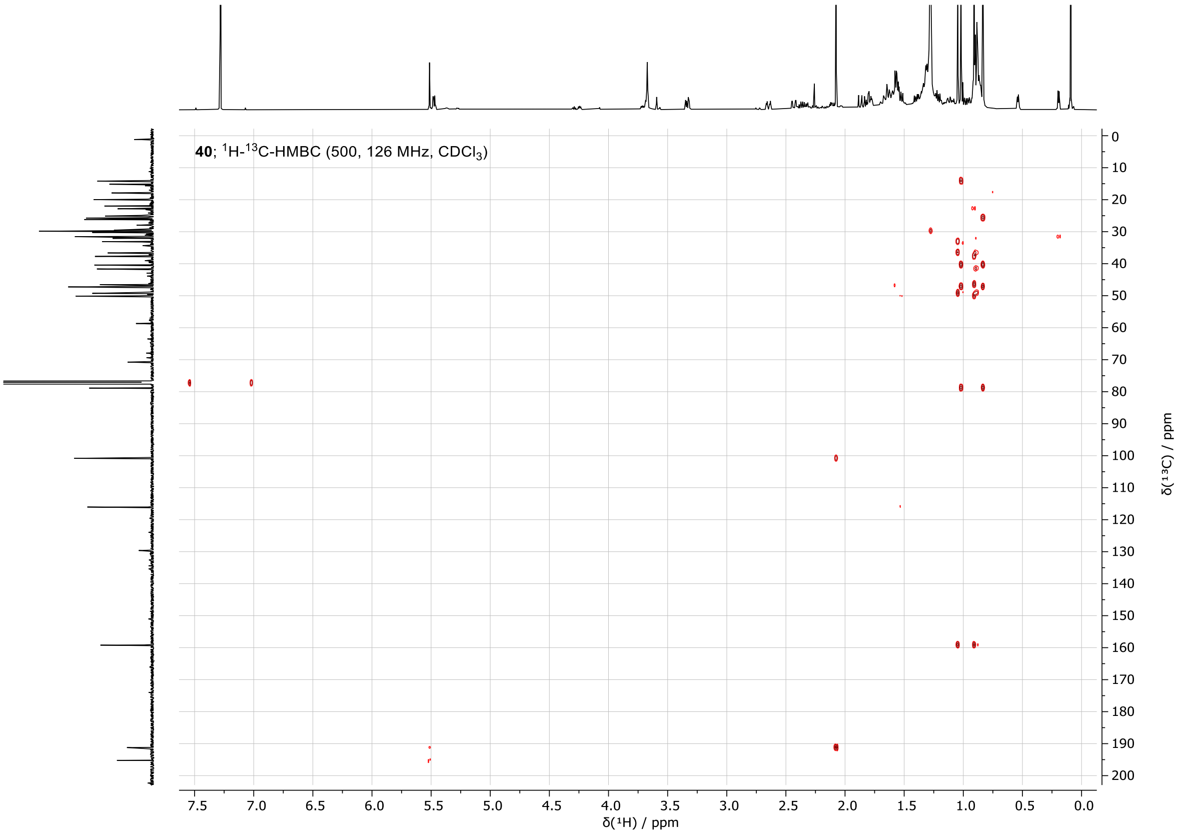
40; ^{1}H-^{13}C-HMBC (500, 126 MHz, CDCl$_3$)
δ(¹H) / ppm
δ(¹³C) / ppm

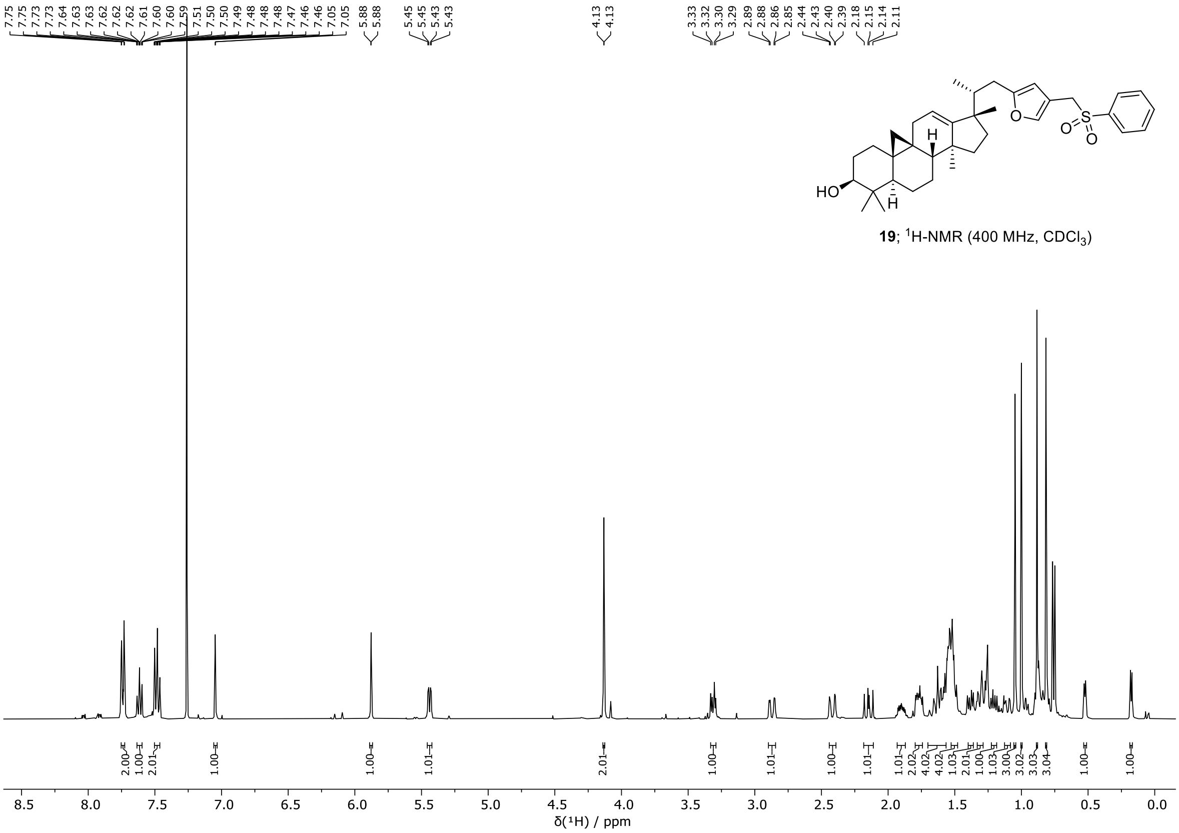
HO
H
H
19; ^{1}H-NMR (400 MHz, CDCl$_3$)
δ(¹H) / ppm

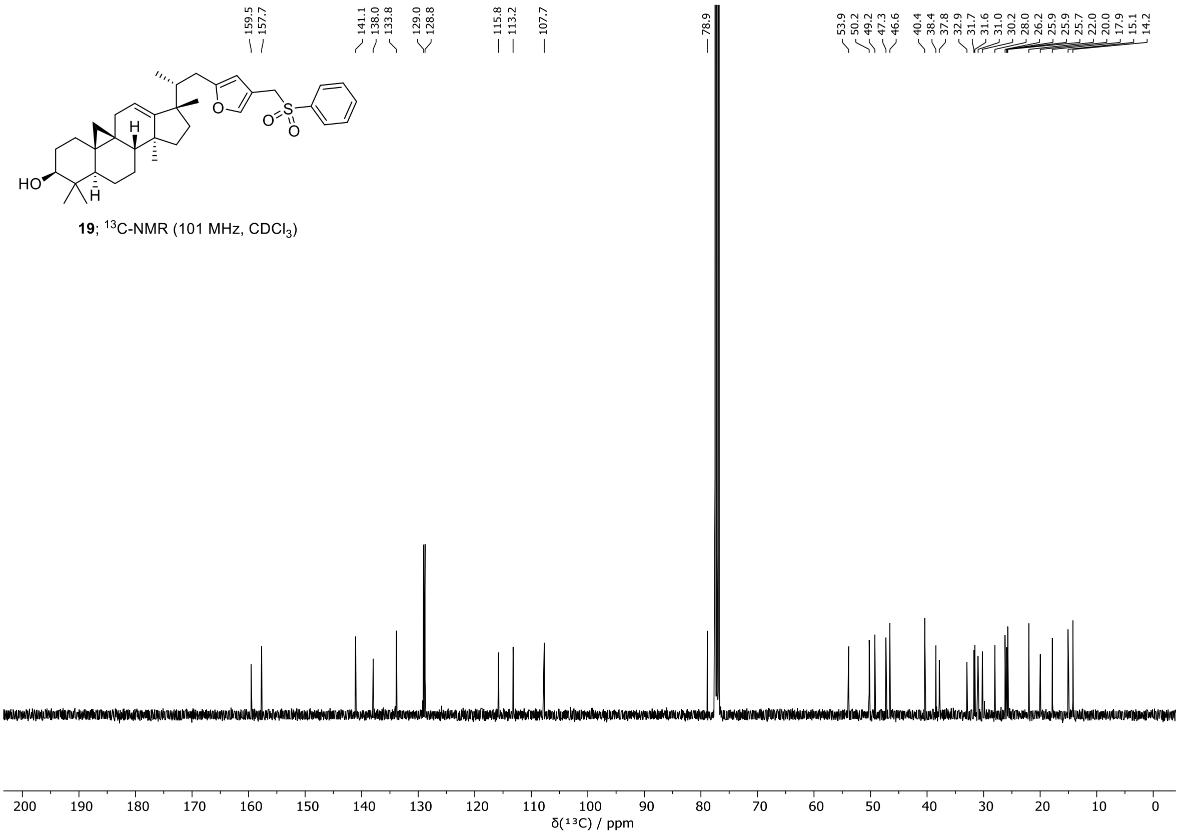

19; ^{13}C-NMR (101 MHz, $CDCl_3$)

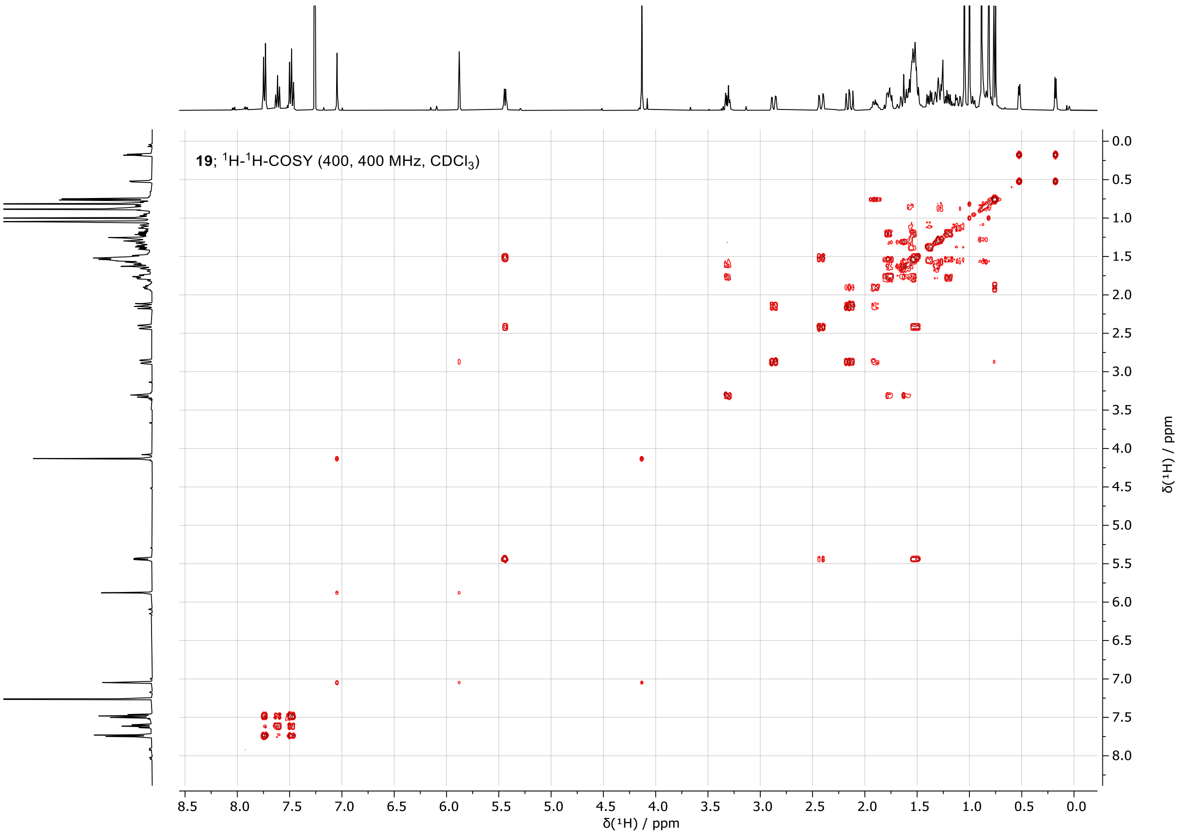

19; ^{1}H-^{1}H-COSY (400, 400 MHz, $CDCl_3$)

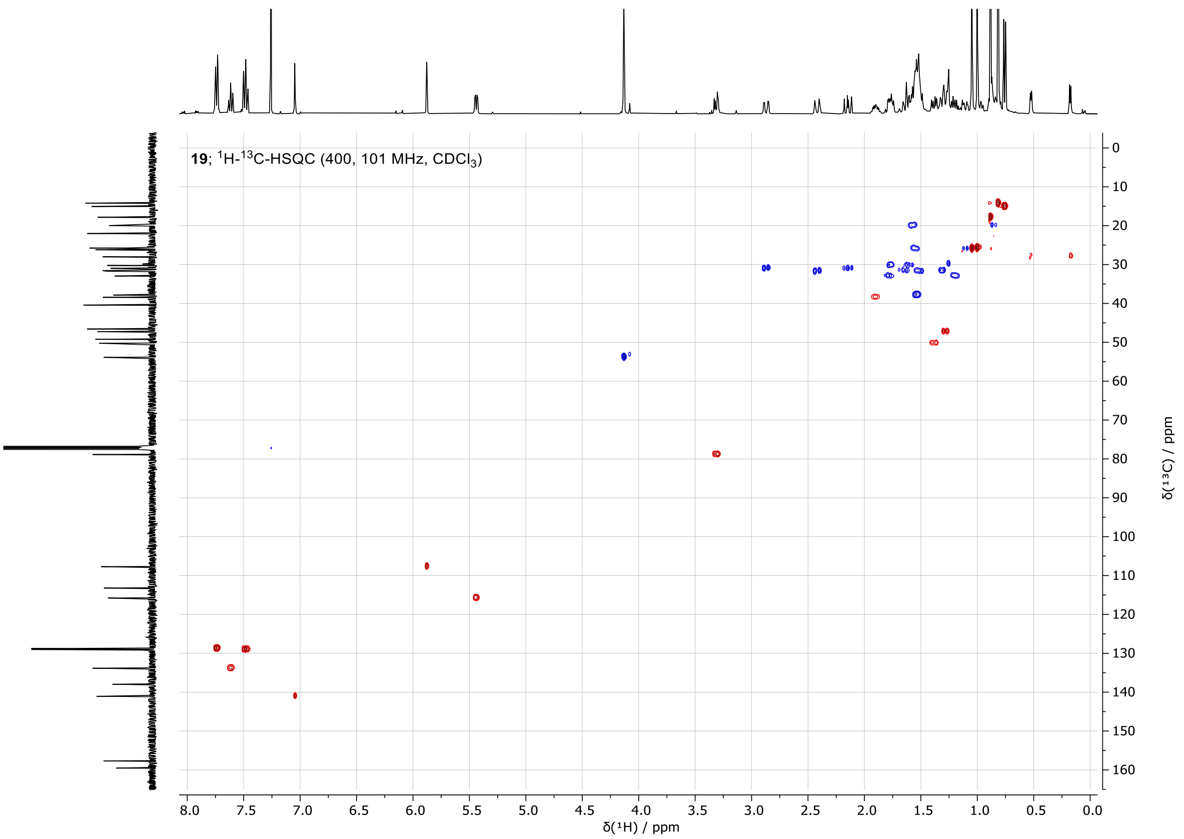
19; ^{1}H-^{13}C-HSQC (400, 101 MHz, CDCl$_3$)
δ(¹H) / ppm
δ(¹³C) / ppm

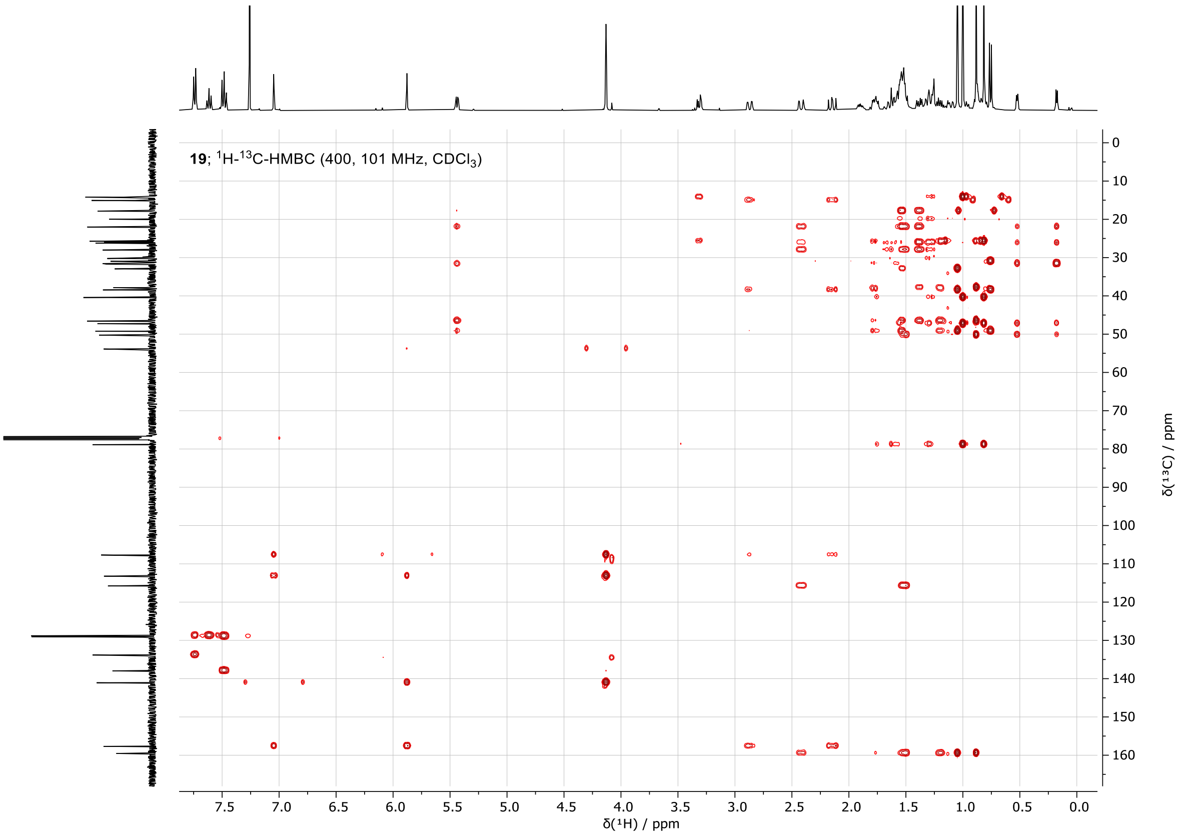
19; ^{1}H-^{13}C-HMBC (400, 101 MHz, CDCl$_3$)
δ(¹H) / ppm
δ(¹³C) / ppm

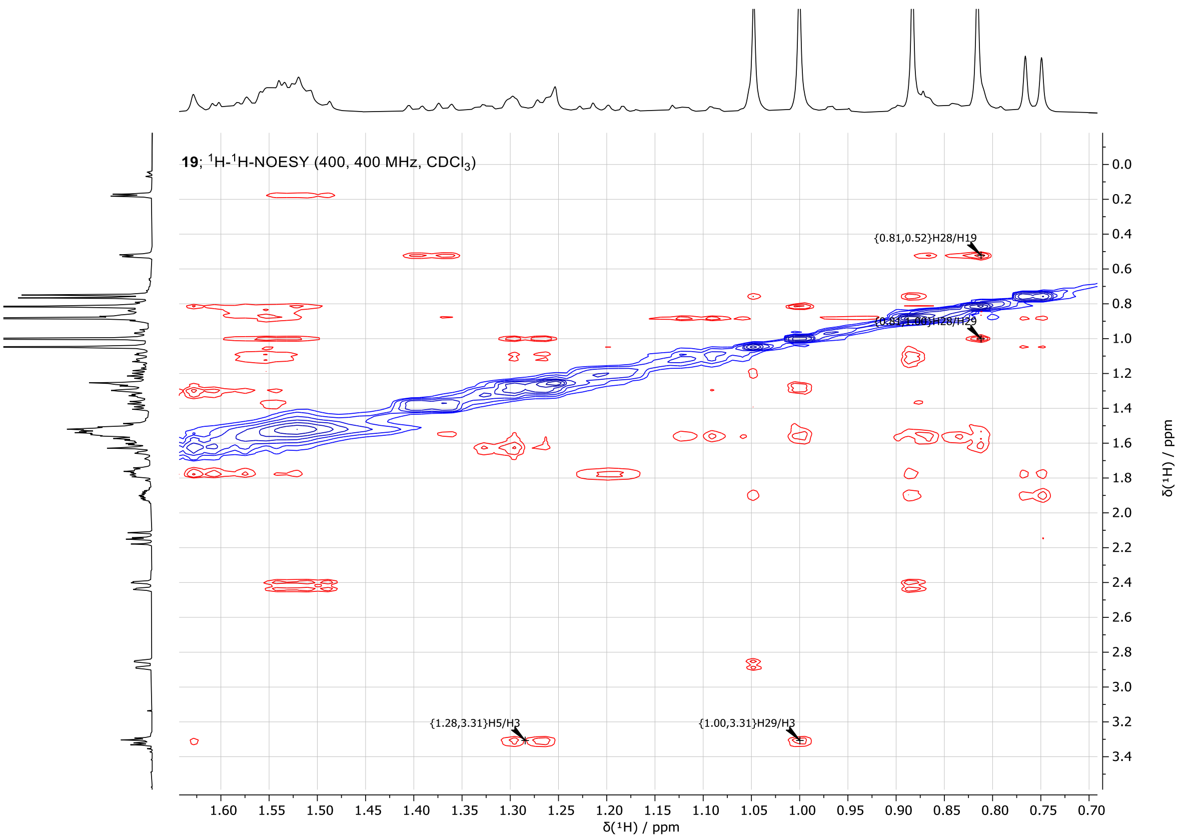
19; ¹H-¹H-NOESY (400, 400 MHz, CDCl3)
{0.81,0.52}H28/H19
{1.28,3.31}H5/H3
{1.00,3.31}H29/H3
δ(¹H) / ppm

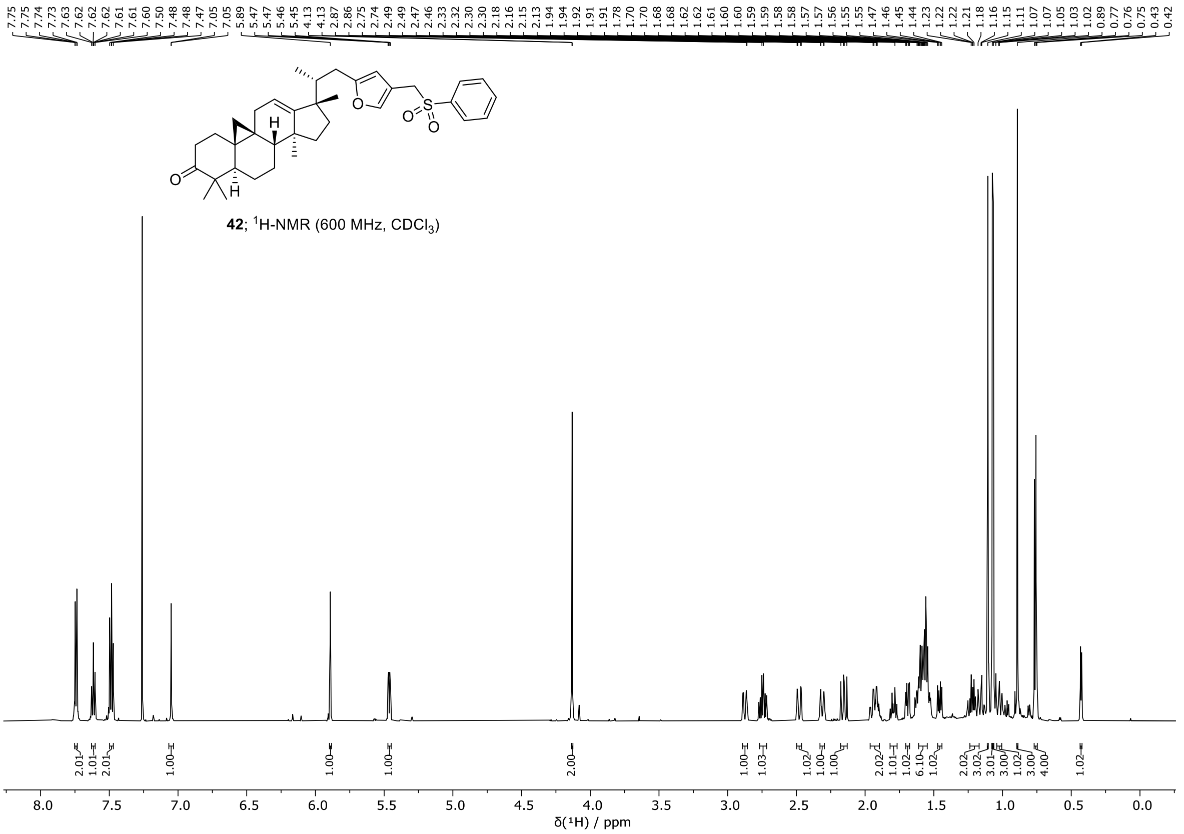
42; ¹H-NMR (600 MHz, CDCl3)
δ(¹H) / ppm

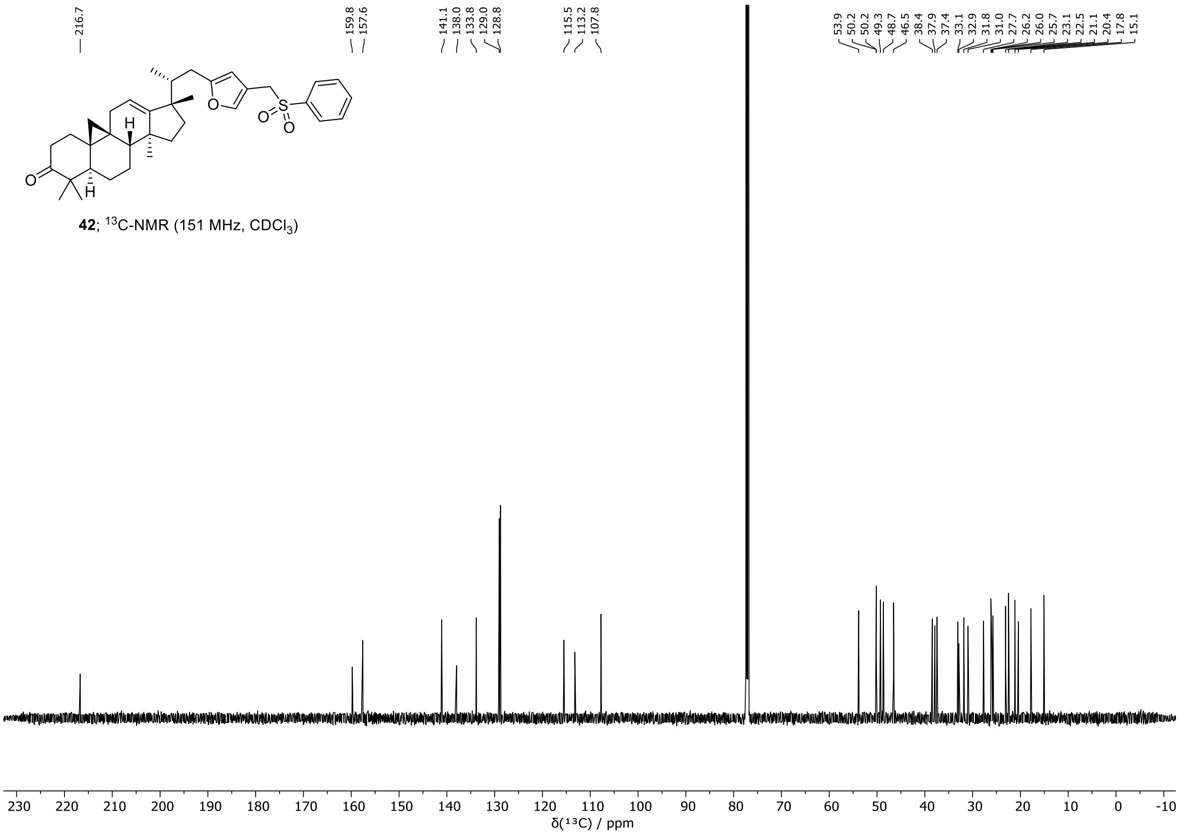
216.7
159.8
157.6
141.1
138.0
133.8
129.0
128.8
115.5
113.2
107.8
53.9
50.2
50.2
49.3
48.7
46.5
38.4
37.9
37.4
33.1
32.9
31.8
31.0
27.7
26.2
26.0
25.7
23.1
22.5
21.1
20.4
17.8
15.1
H
O
O
S
O
O
H
42; 13C-NMR (151 MHz, CDCl3)
δ(13C) / ppm

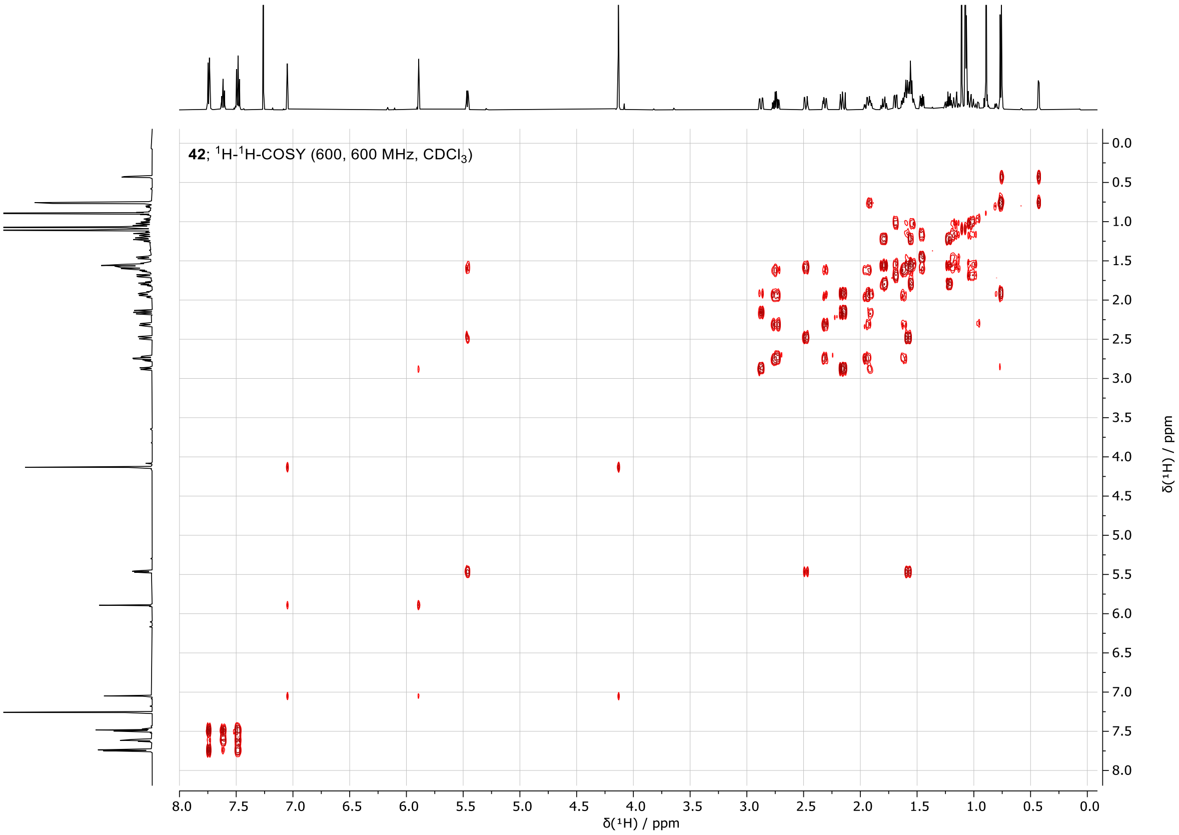
42; 1H-1H-COSY (600, 600 MHz, CDCl3)
δ(1H) / ppm
δ(1H) / ppm

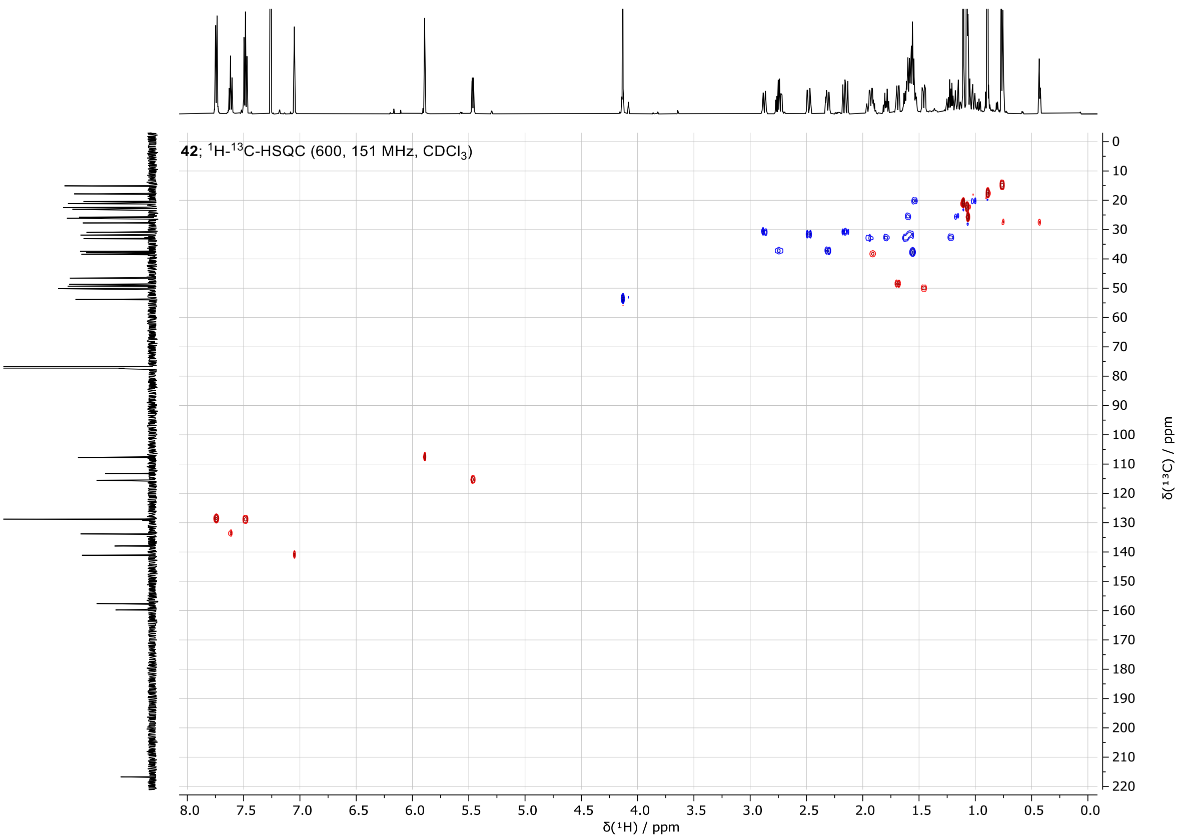

42; ¹H-¹³C-HSQC (600, 151 MHz, CDCl₃)
δ(¹H) / ppm
δ(¹³C) / ppm

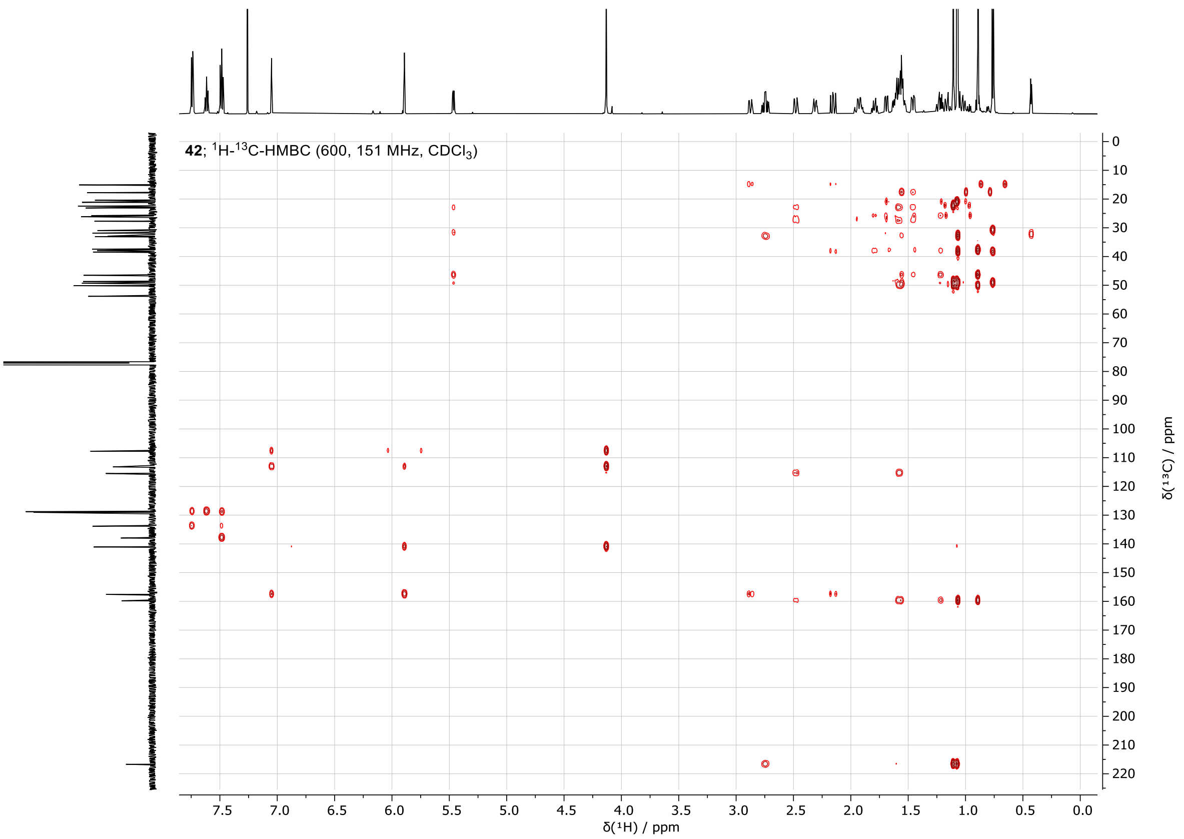

42; ¹H-¹³C-HMBC (600, 151 MHz, CDCl₃)
δ(¹H) / ppm
δ(¹³C) / ppm

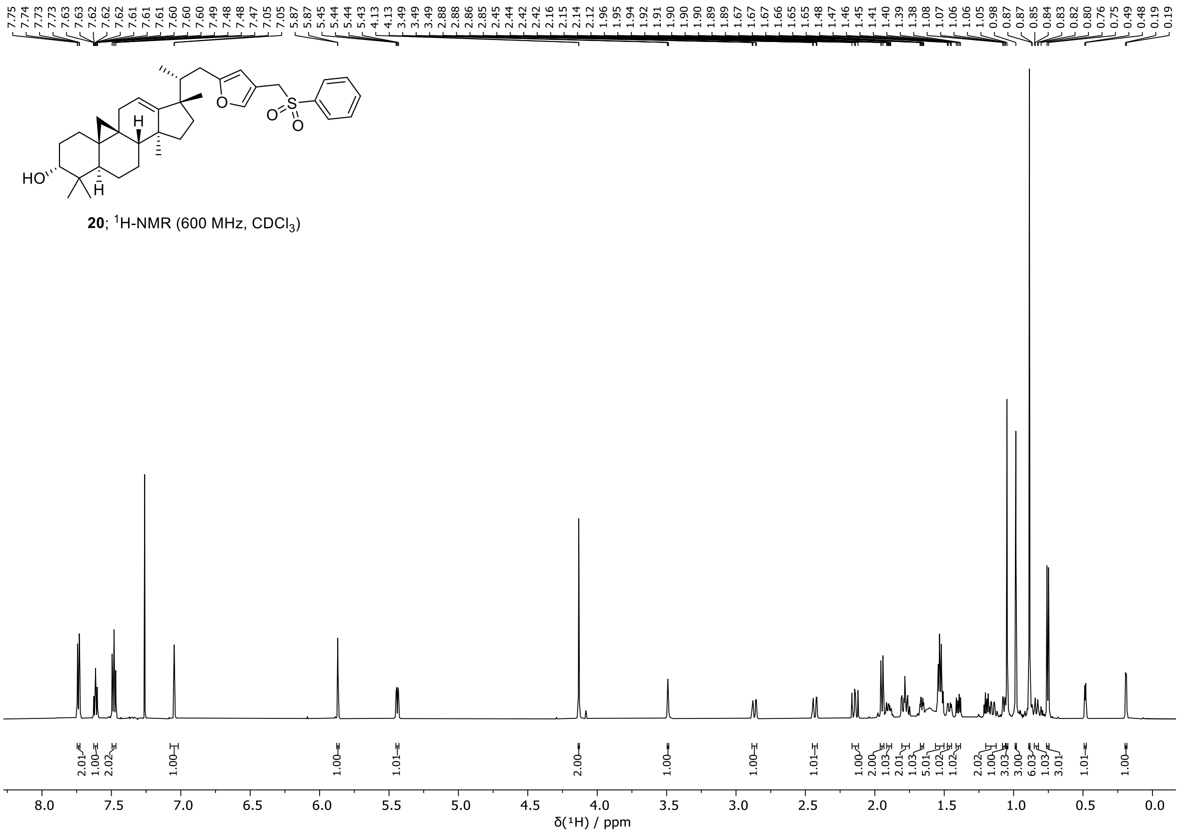

20; ^{1}H-NMR (600 MHz, CDCl$_3$)
δ(¹H) / ppm

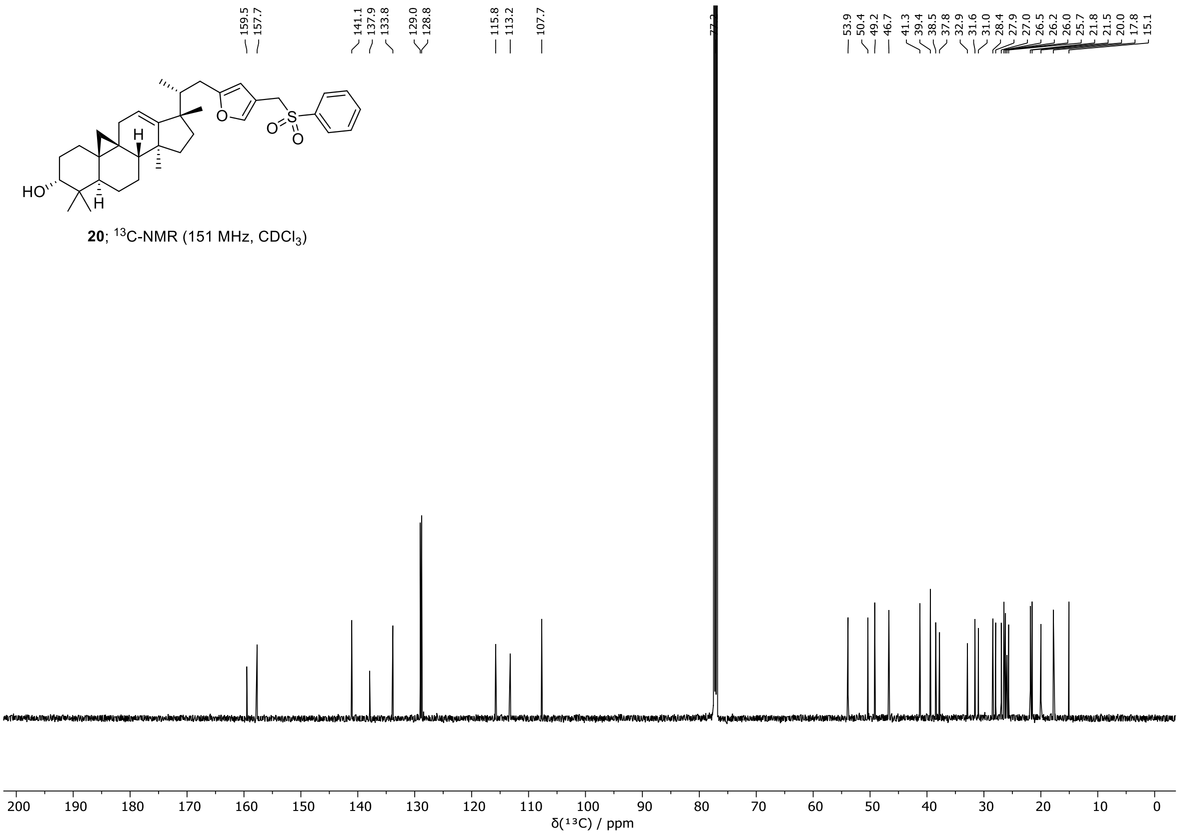

159.5
157.7
141.1
137.9
133.8
129.0
128.8
115.8
113.2
107.7
53.9
50.4
49.2
46.7
41.3
39.4
38.5
37.8
32.9
31.6
31.0
28.4
27.9
27.0
26.5
26.2
26.0
25.7
21.8
21.5
20.0
17.8
15.1
20; ^{13}C-NMR (151 MHz, CDCl$_3$)
δ(¹³C) / ppm

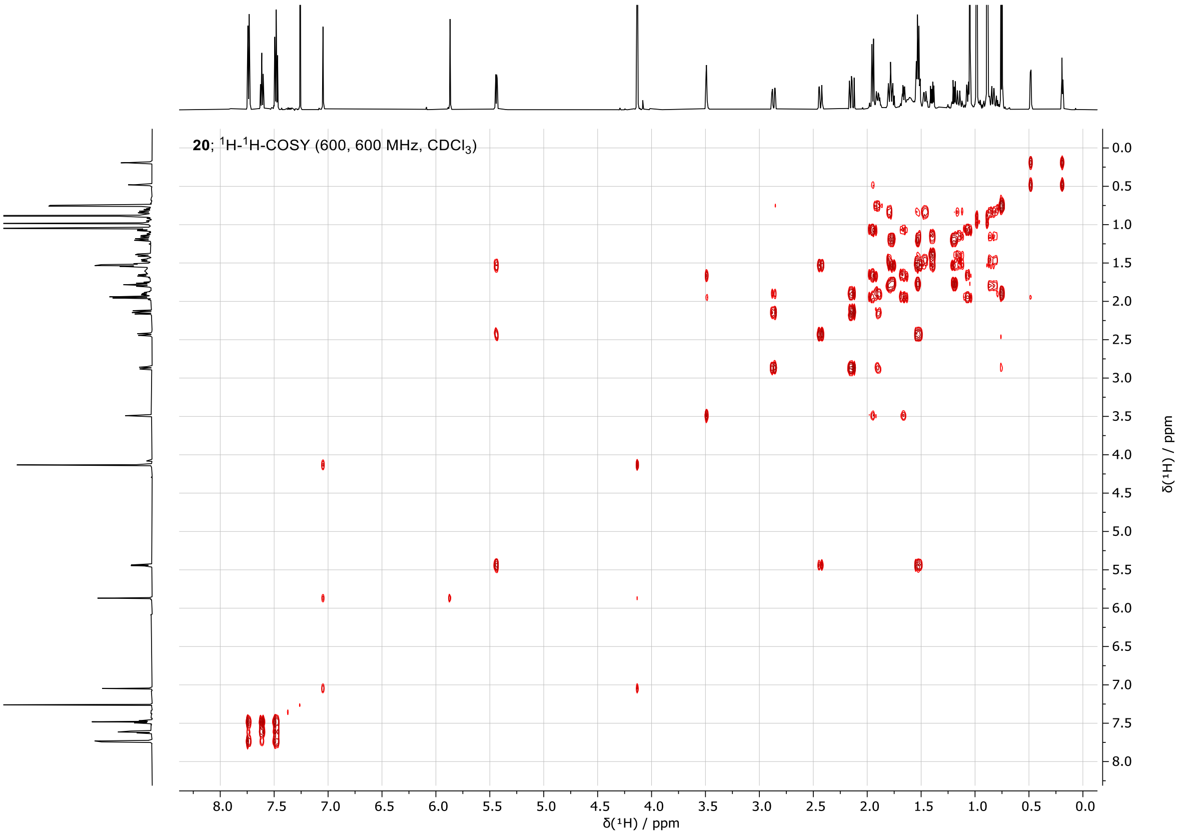

20; ^{1}H-^{1}H-COSY (600, 600 MHz, $CDCl_3$)
δ(¹H) / ppm
δ(¹H) / ppm

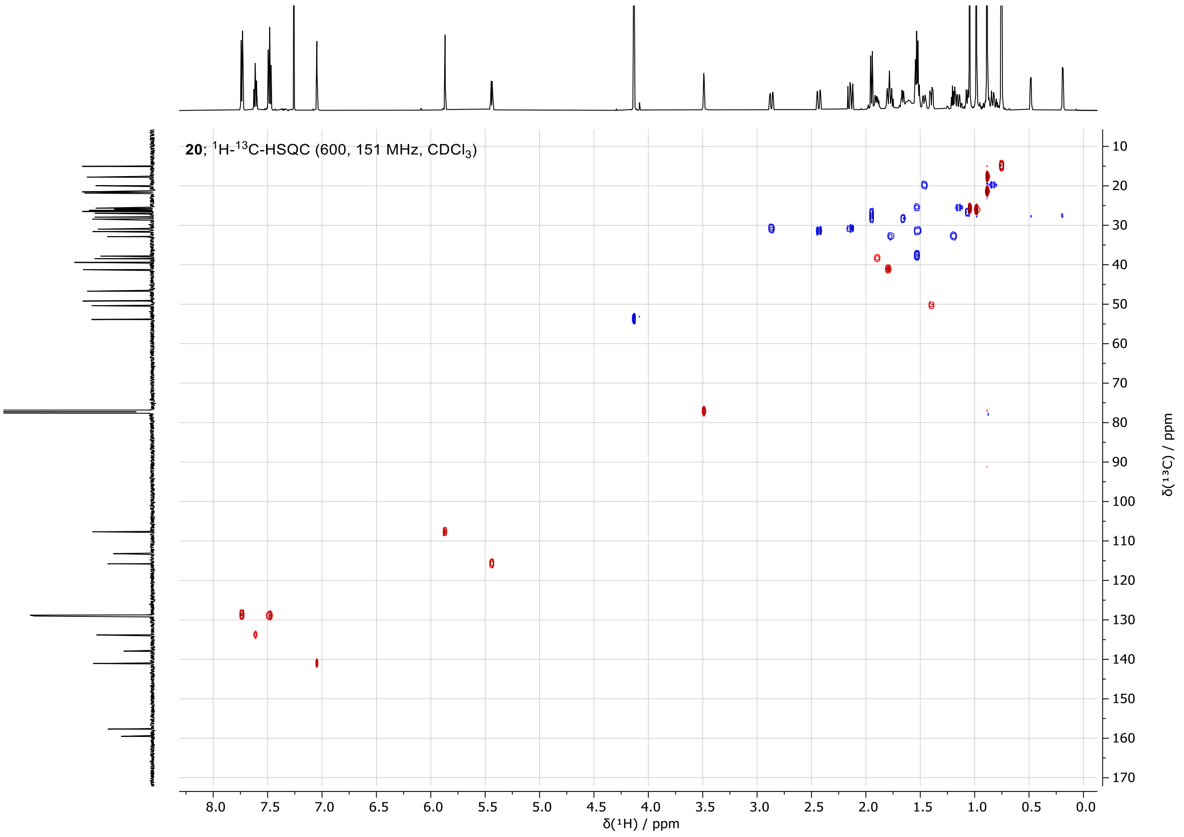

20; ^{1}H-^{13}C-HSQC (600, 151 MHz, $CDCl_3$)
δ(¹H) / ppm
δ(¹³C) / ppm

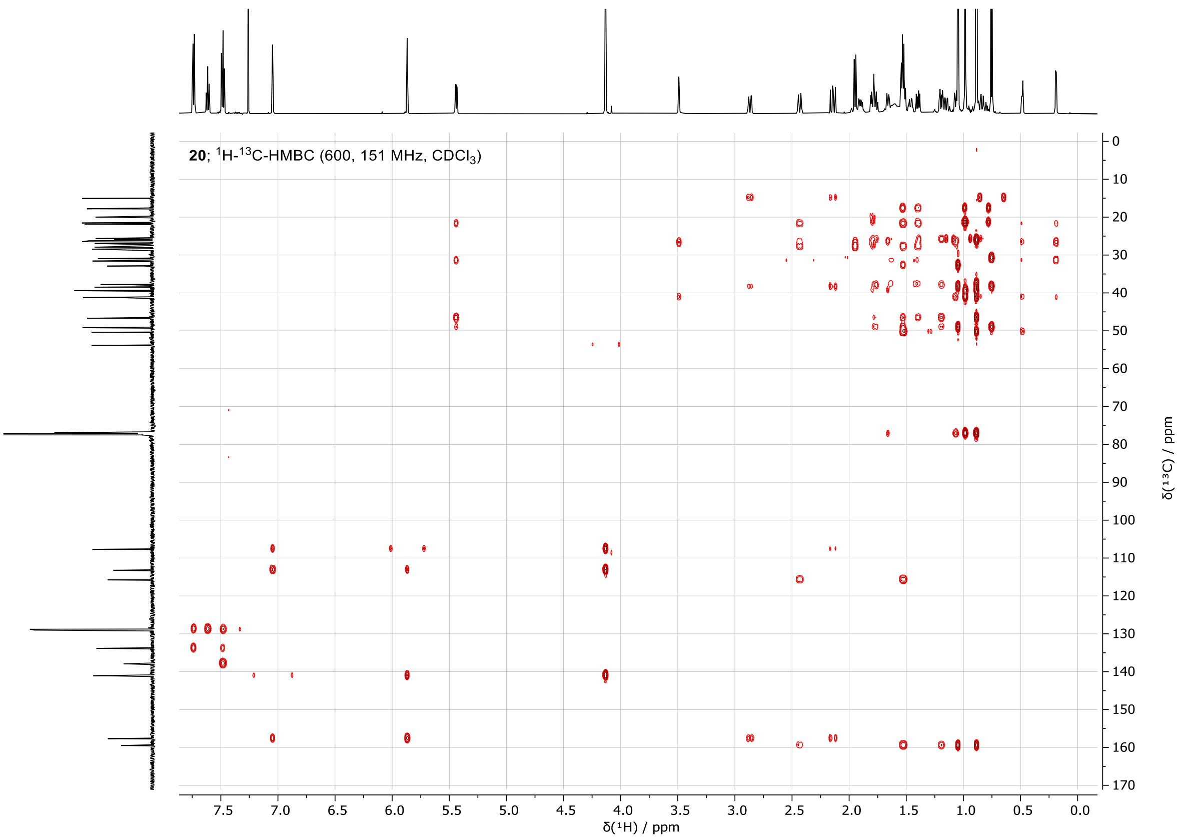
20; ^{1}H-^{13}C-HMBC (600, 151 MHz, $CDCl_3$)
δ(¹H) / ppm
δ(¹³C) / ppm

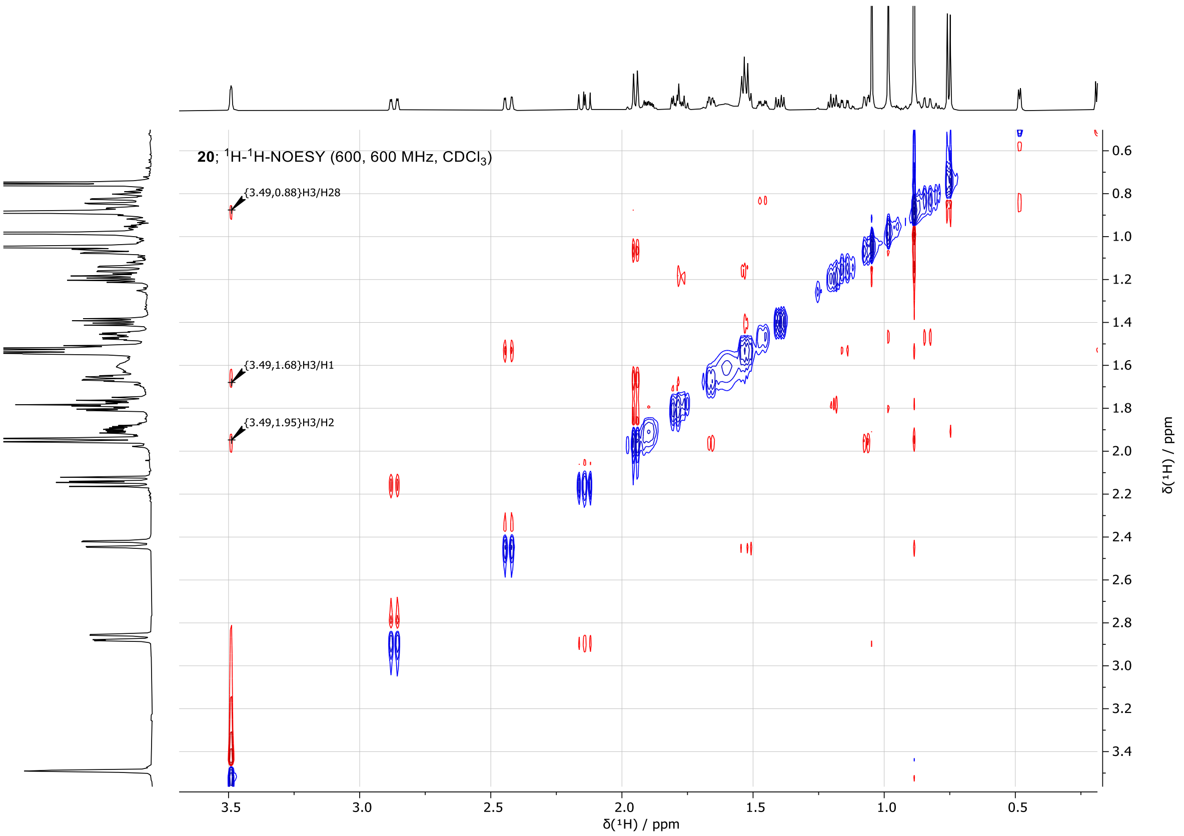
20; ^{1}H-^{1}H-NOESY (600, 600 MHz, $CDCl_3$)
{3.49,0.88}H3/H28
{3.49,1.68}H3/H1
{3.49,1.95}H3/H2
δ(¹H) / ppm
δ(¹H) / ppm

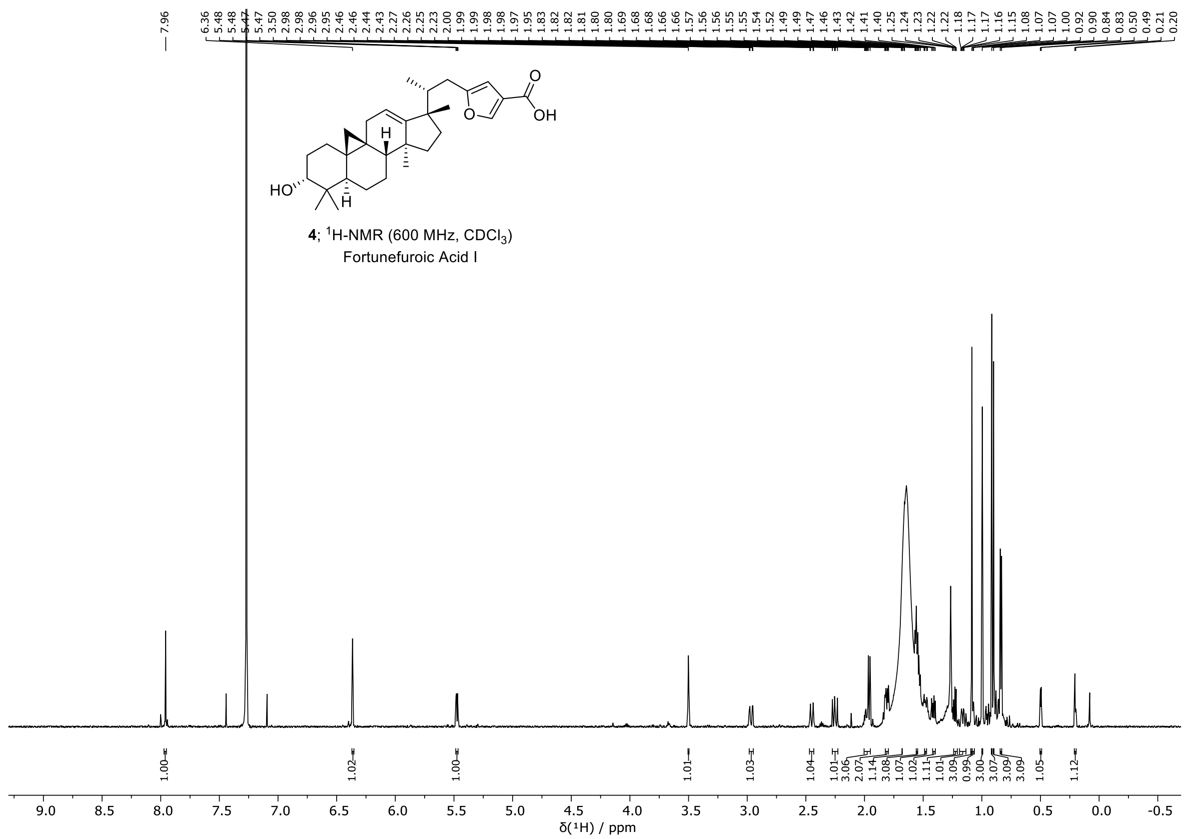
4; 1H-NMR (600 MHz, CDCl3)
Fortunefuroic Acid I
δ(1H) / ppm

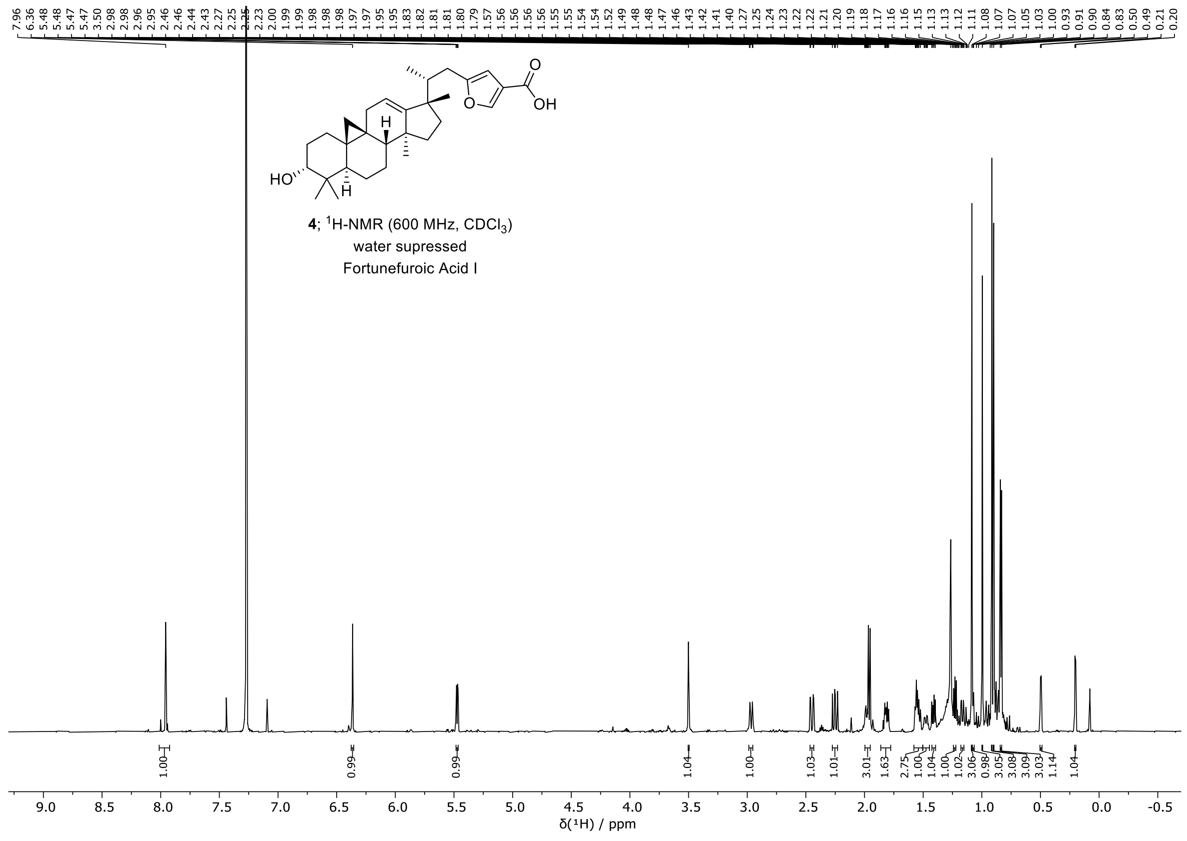
4; 1H-NMR (600 MHz, CDCl3)
water supressed
Fortunefuroic Acid I
δ(1H) / ppm

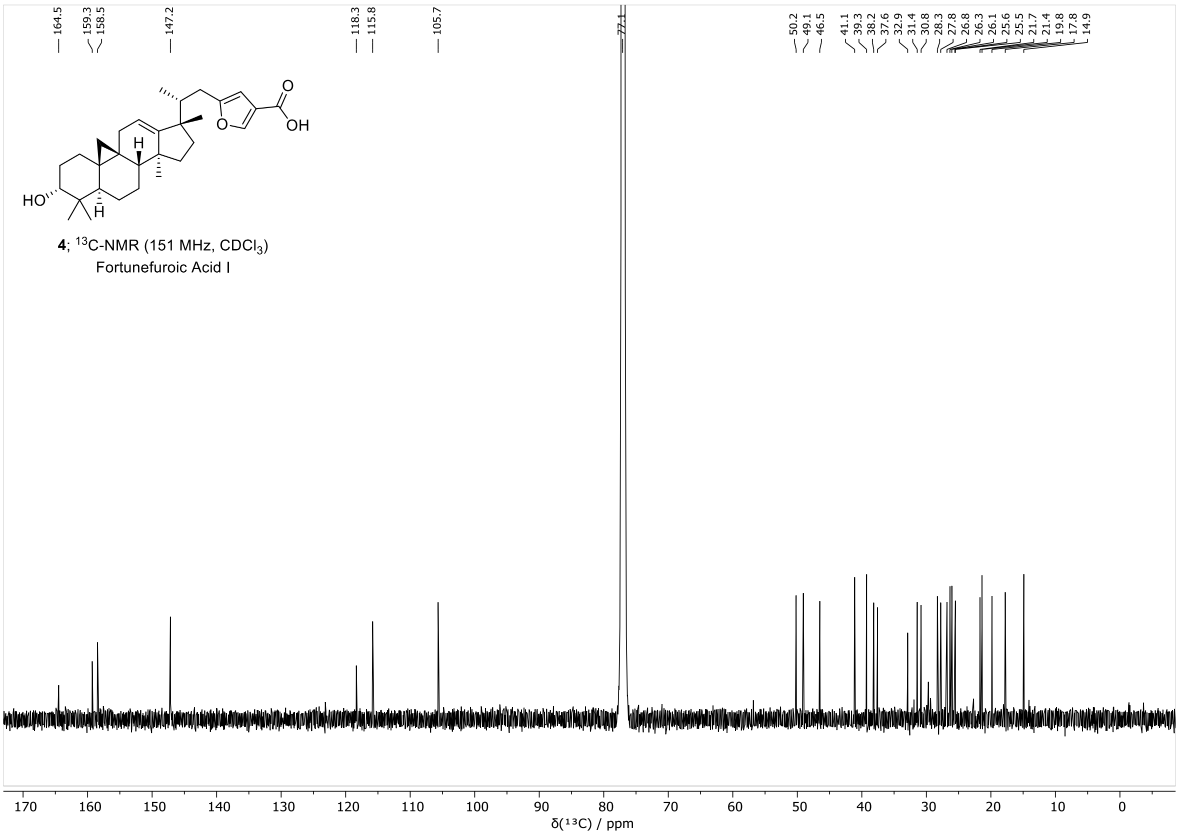
164.5
159.3
158.5
147.2
118.3
115.8
105.7
77.1
50.2
49.1
46.5
41.1
39.3
38.2
37.6
32.9
31.4
30.8
28.3
27.8
26.8
26.3
26.1
25.6
25.5
21.7
21.4
19.8
17.8
14.9
O
OH
O
H
HO
H
4; ^{13}C-NMR (151 MHz, CDCl$_3$)
Fortunefuroic Acid I
δ(¹³C) / ppm

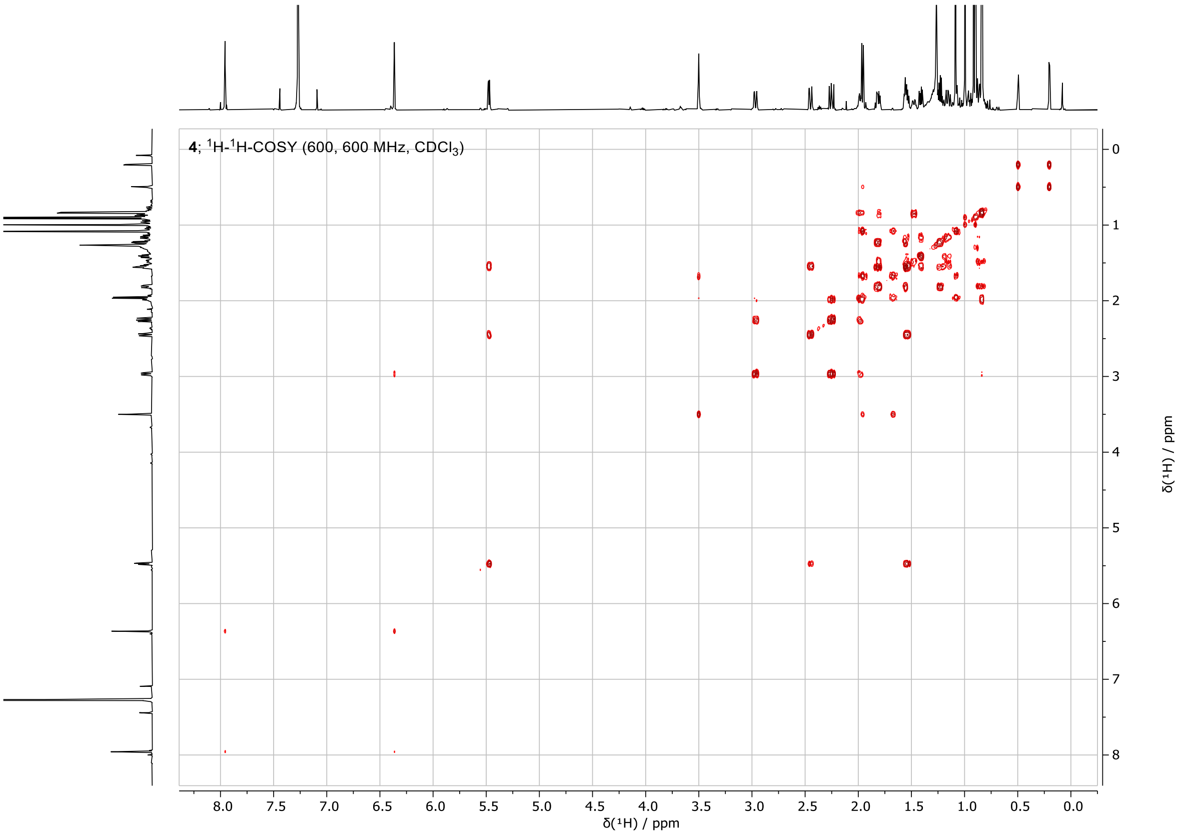
4; ^{1}H-^{1}H-COSY (600, 600 MHz, CDCl$_3$)
δ(¹H) / ppm
δ(¹H) / ppm

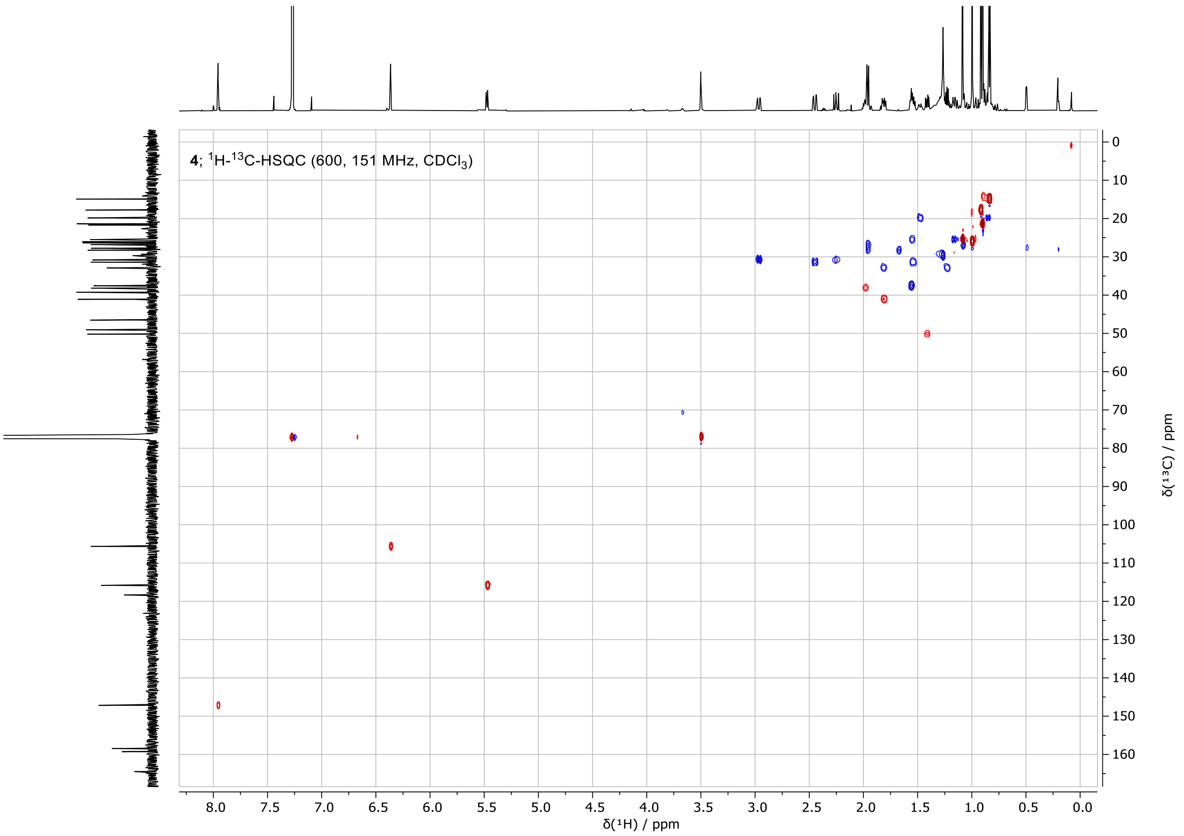
4; ^{1}H-^{13}C-HSQC (600, 151 MHz, $CDCl_3$)
δ(¹H) / ppm
δ(¹³C) / ppm

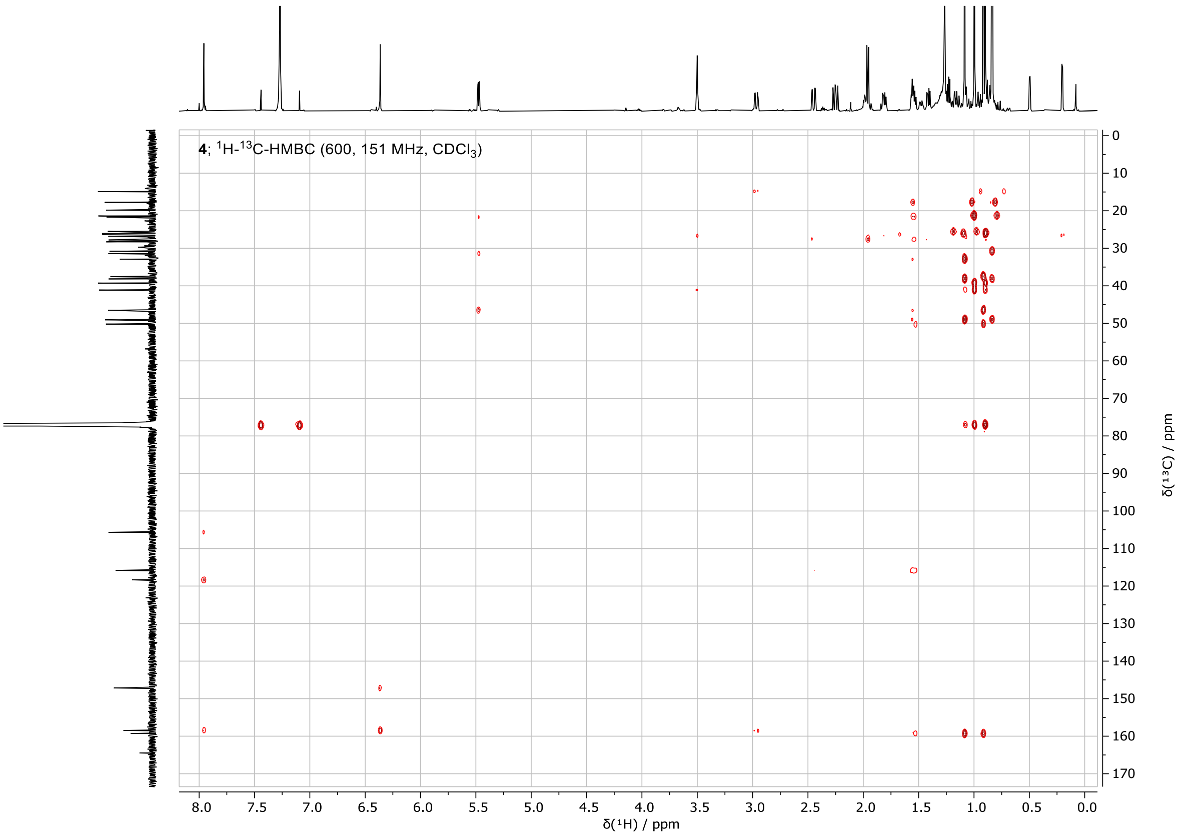
4; ^{1}H-^{13}C-HMBC (600, 151 MHz, $CDCl_3$)
δ(¹H) / ppm
δ(¹³C) / ppm

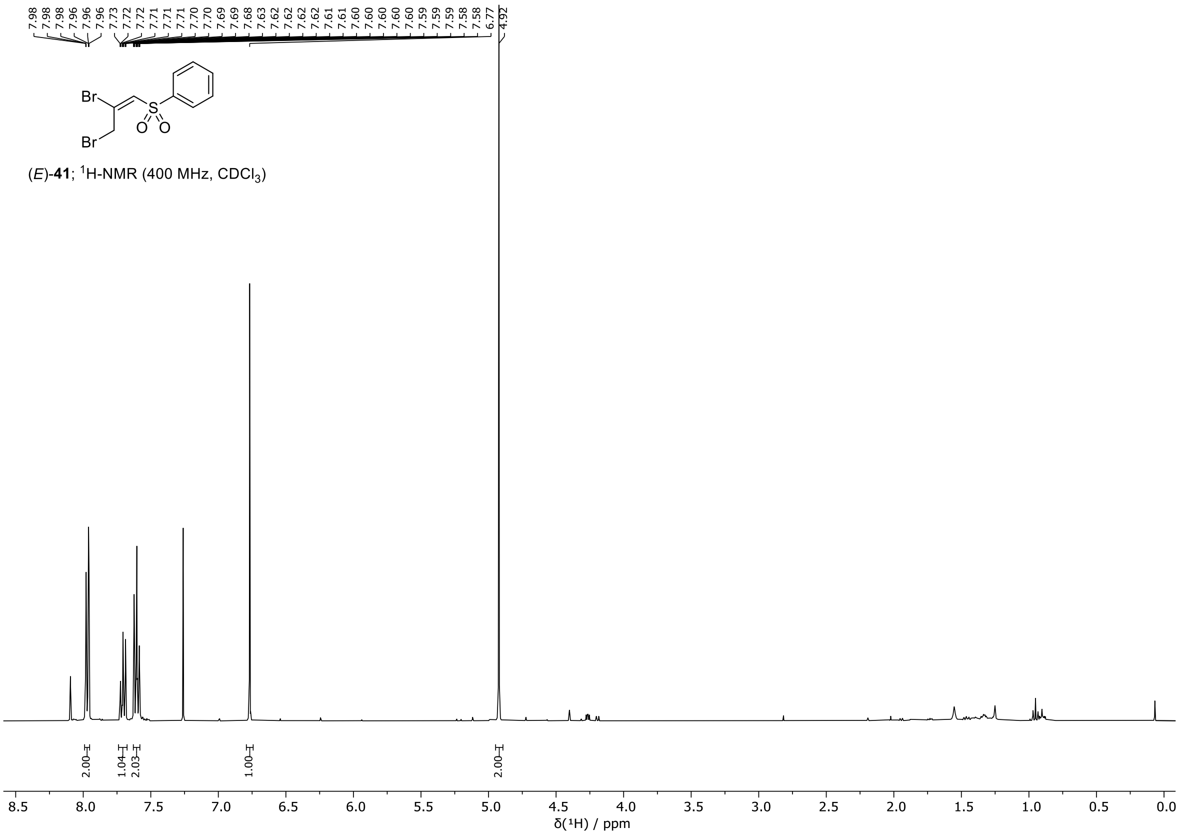

(*E*)-**41**; ^{1}H-NMR (400 MHz, $CDCl_3$)

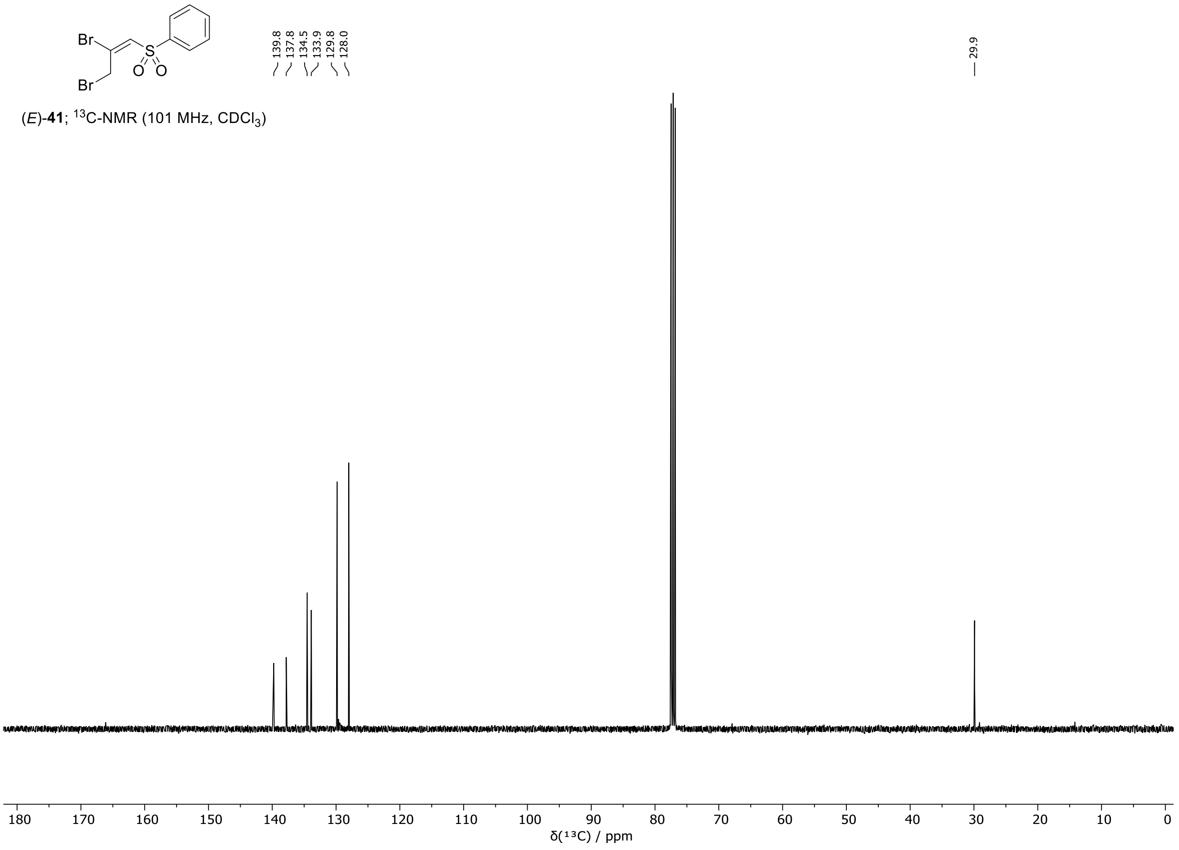

(*E*)-**41**; ^{13}C-NMR (101 MHz, $CDCl_3$)

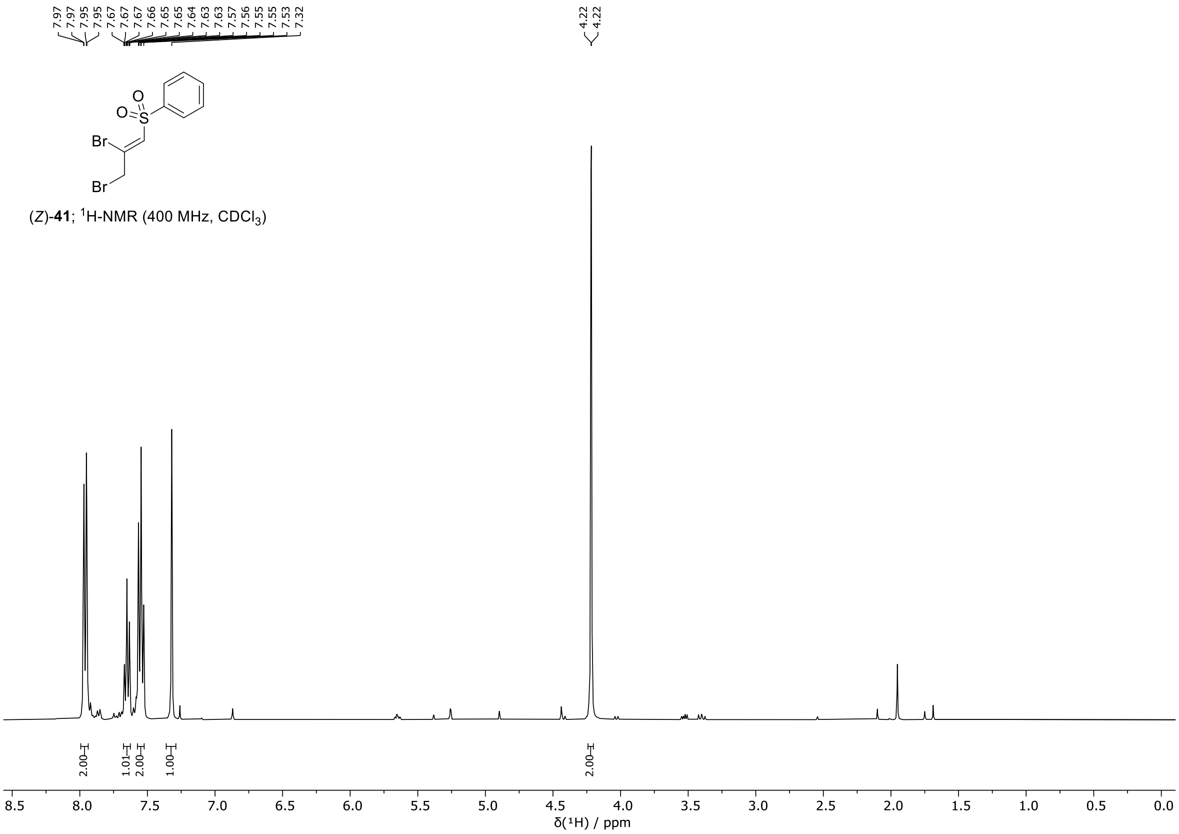

(*Z*)-**41**; ^{1}H-NMR (400 MHz, $CDCl_3$)

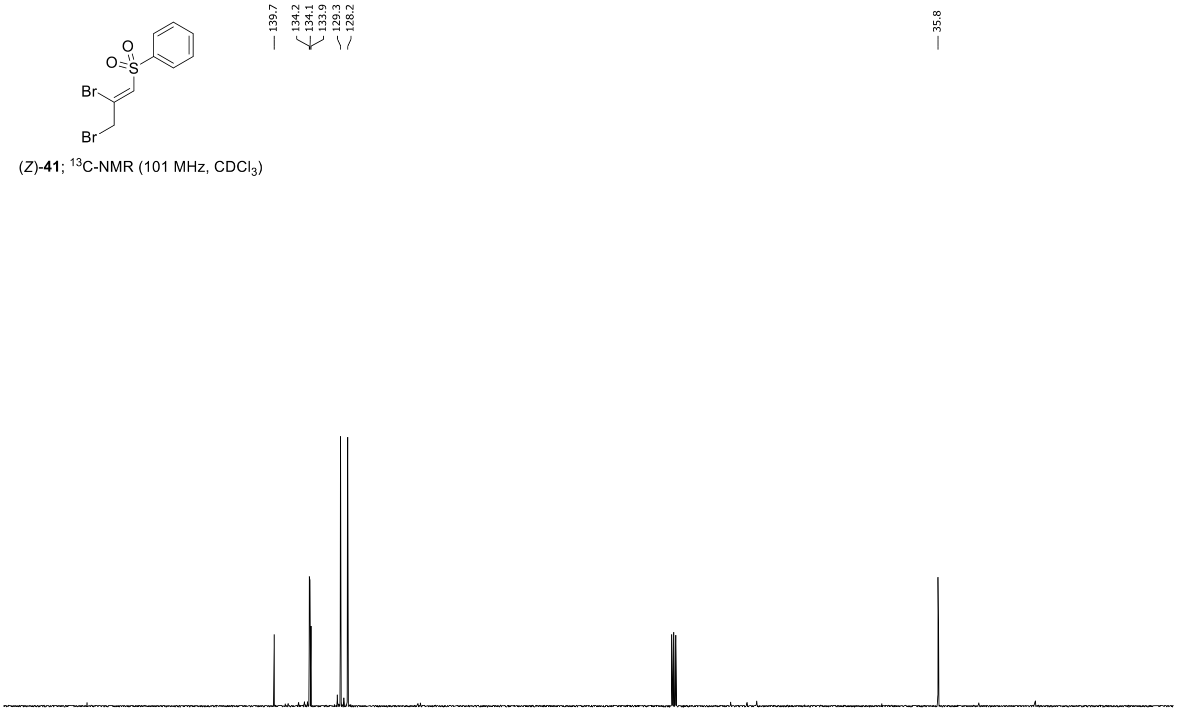

(*Z*)-**41**; ^{13}C-NMR (101 MHz, $CDCl_3$)

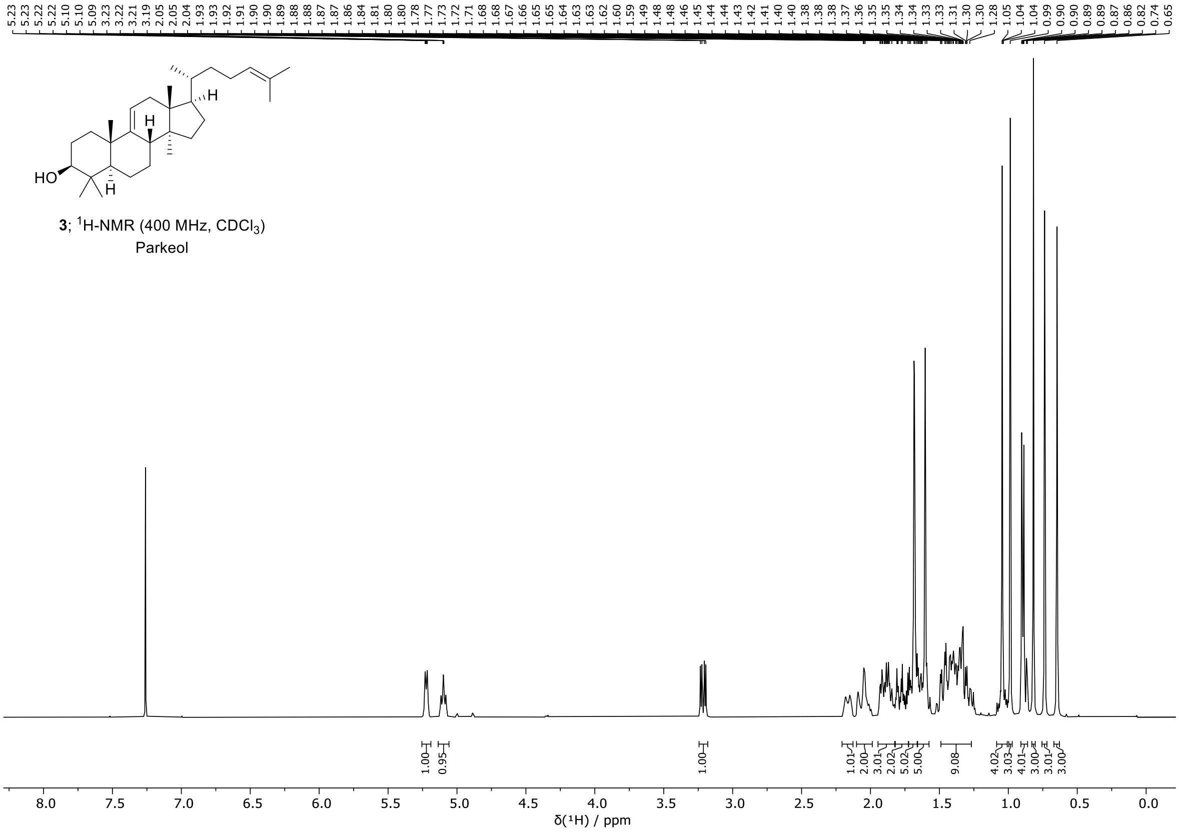
5.23 5.23 5.22 5.10 5.09 3.23 3.22 3.21 3.19 2.05 2.05 2.04 1.93 1.93 1.92 1.91 1.90 1.90 1.89 1.88 1.88 1.87 1.87 1.86 1.84 1.81 1.80 1.80 1.78 1.77 1.73 1.72 1.71 1.68 1.68 1.67 1.66 1.65 1.65 1.64 1.63 1.63 1.62 1.60 1.59 1.49 1.48 1.48 1.46 1.45 1.44 1.44 1.43 1.42 1.41 1.40 1.40 1.38 1.38 1.38 1.37 1.36 1.35 1.35 1.35 1.34 1.34 1.33 1.33 1.31 1.30 1.30 1.30 1.28 1.05 1.04 1.04 0.99 0.90 0.90 0.89 0.89 0.87 0.86 0.82 0.74 0.65
HO
H
3; ^{1}H-NMR (400 MHz, $CDCl_3$)
Parkeol
1.00 0.95 1.00 1.01 2.00 3.01 2.02 5.02 5.00 9.08 4.02 3.03 4.01 3.00 3.01 3.00
8.0 7.5 7.0 6.5 6.0 5.5 5.0 4.5 4.0 3.5 3.0 2.5 2.0 1.5 1.0 0.5 0.0
δ(^{1}H) / ppm

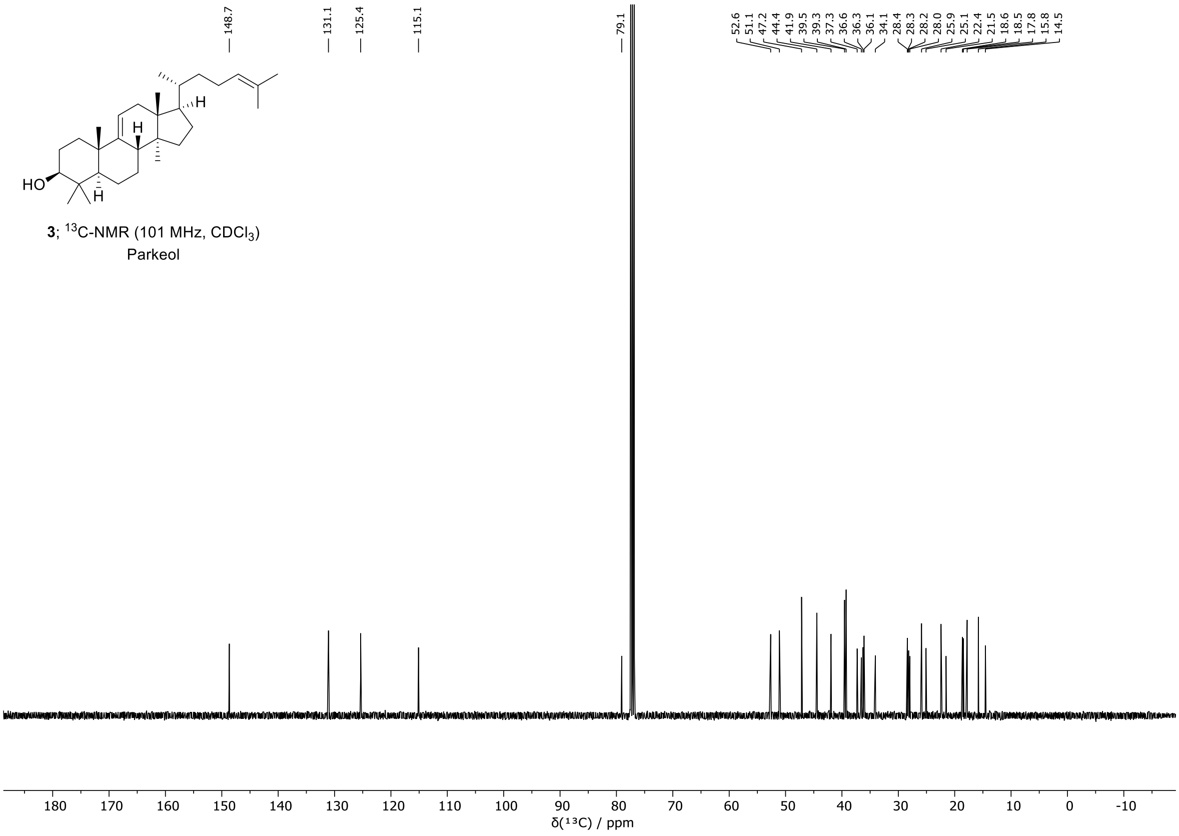
148.7
131.1
125.4
115.1
79.1
52.6 51.1 47.2 44.4 41.9 39.5 39.3 37.3 36.6 36.3 36.1 34.1 28.4 28.3 28.2 28.0 25.9 25.1 22.4 21.5 18.6 18.5 17.8 15.8 14.5
HO
H
3; ^{13}C-NMR (101 MHz, $CDCl_3$)
Parkeol
180 170 160 150 140 130 120 110 100 90 80 70 60 50 40 30 20 10 0 -10
δ(^{13}C) / ppm

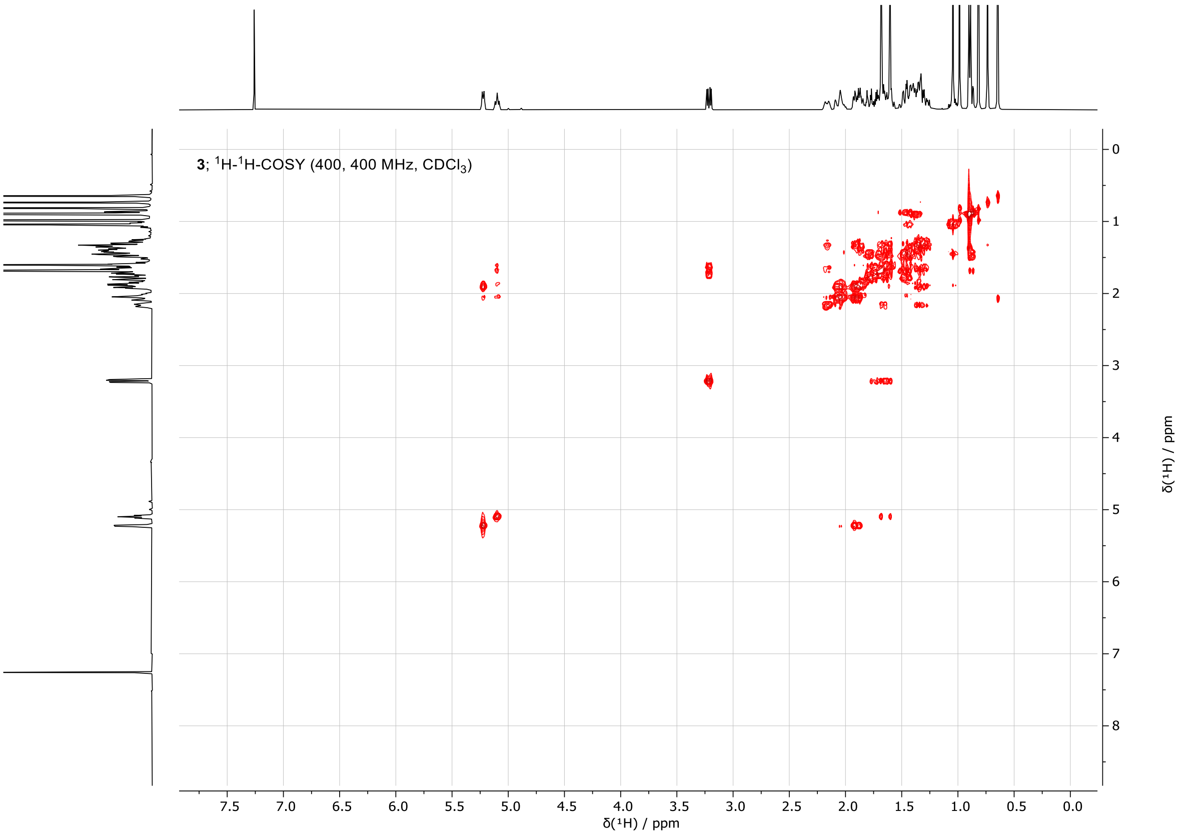
3; ¹H-¹H-COSY (400, 400 MHz, CDCl₃)
δ(¹H) / ppm
δ(¹H) / ppm

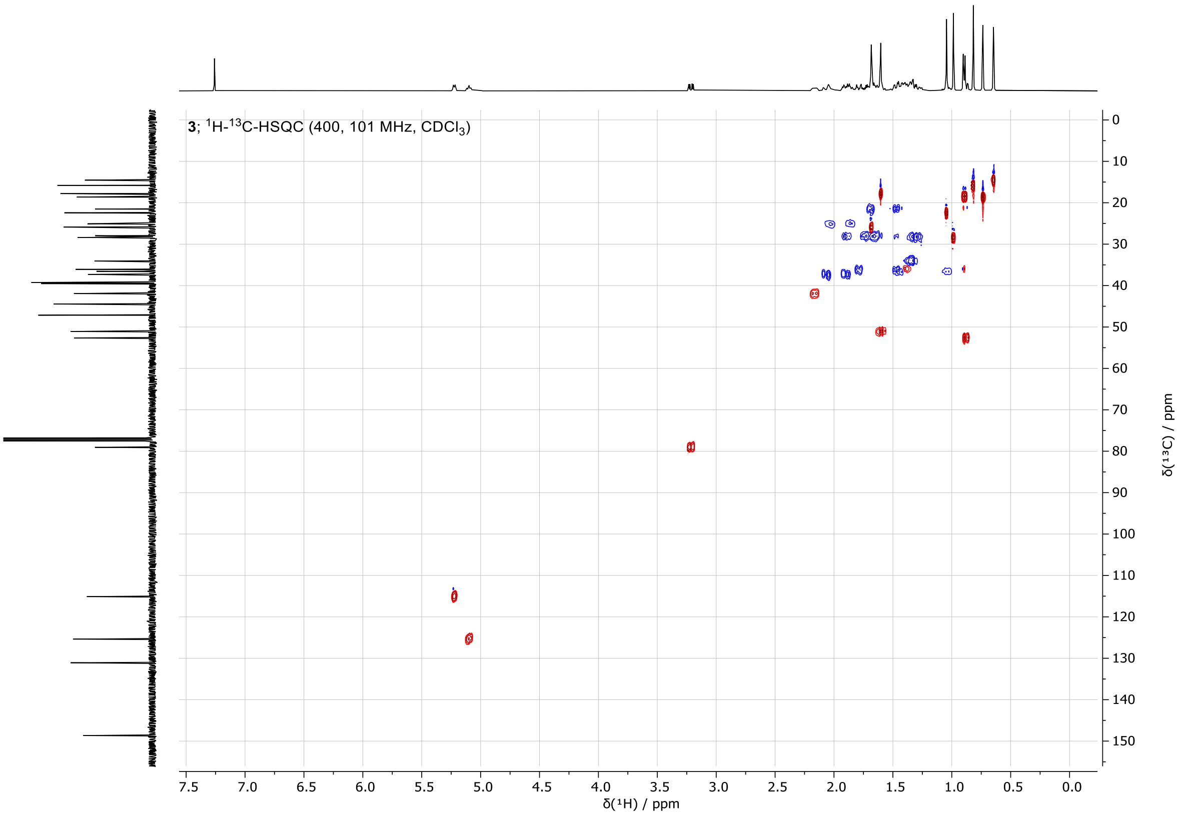
3; ¹H-¹³C-HSQC (400, 101 MHz, CDCl₃)
δ(¹H) / ppm
δ(¹³C) / ppm

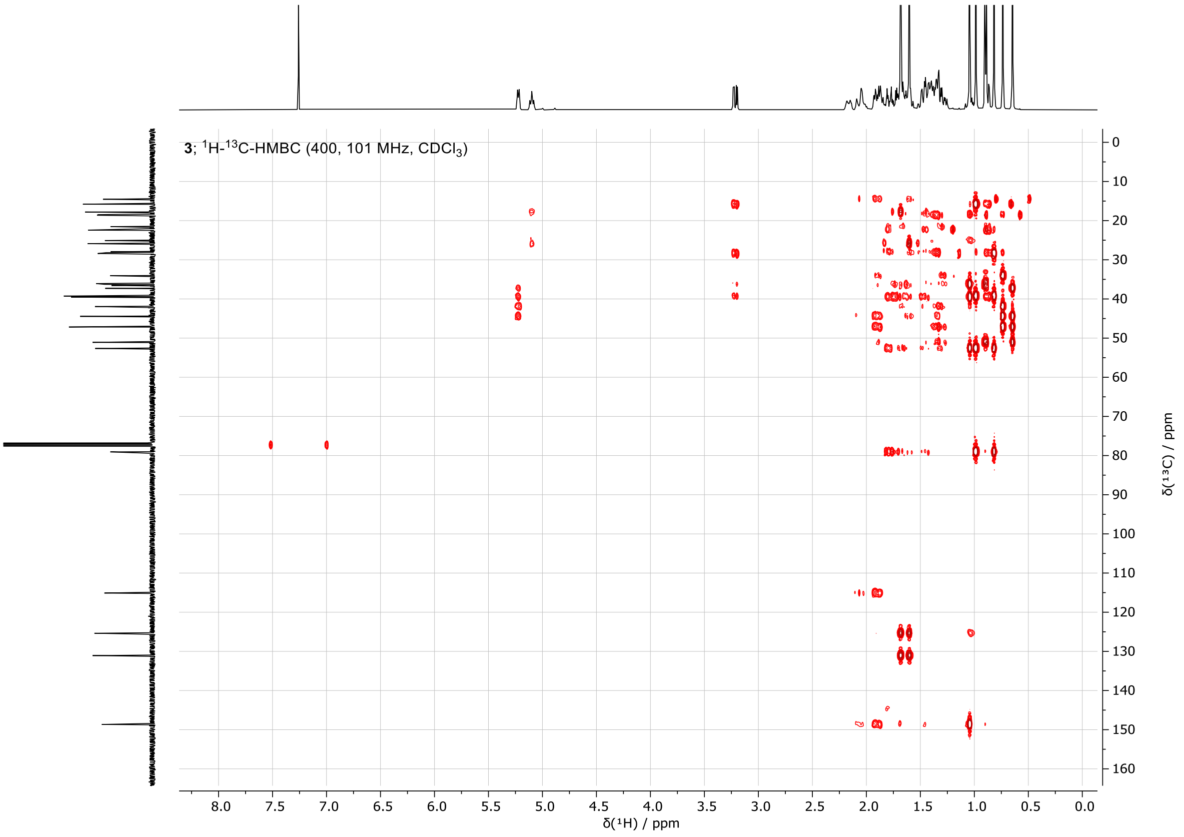

3; ^{1}H-^{13}C-HMBC (400, 101 MHz, CDCl$_3$)
δ(¹H) / ppm
δ(¹³C) / ppm

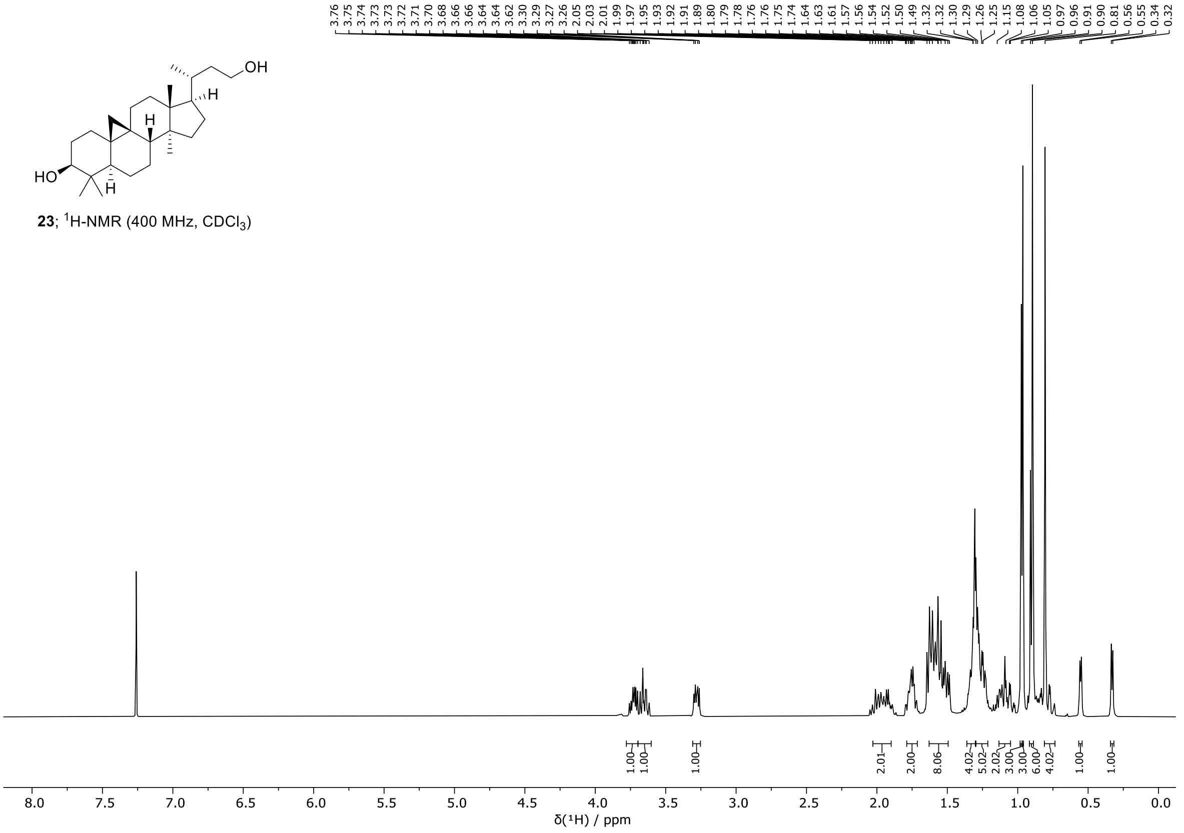

OH
H
H
HO
H
23; ^{1}H-NMR (400 MHz, CDCl$_3$)
δ(¹H) / ppm

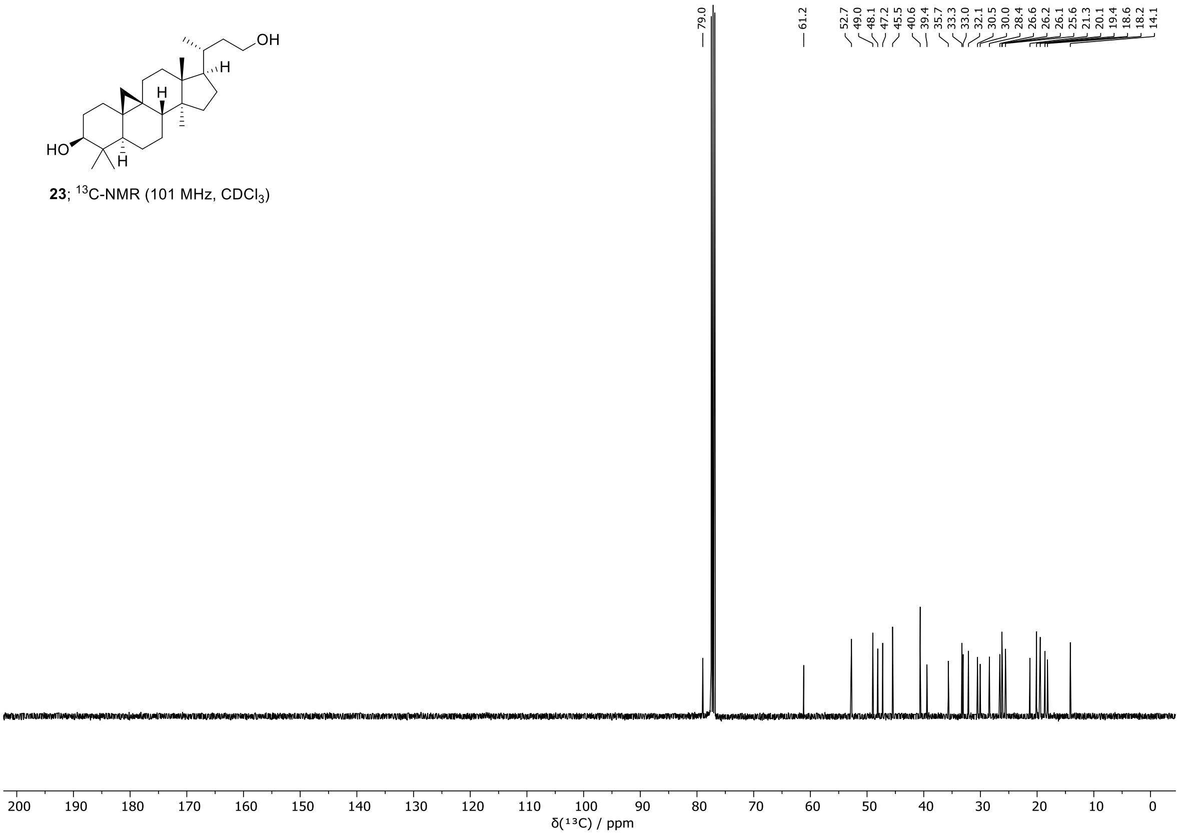

23; ^{13}C-NMR (101 MHz, $CDCl_3$)

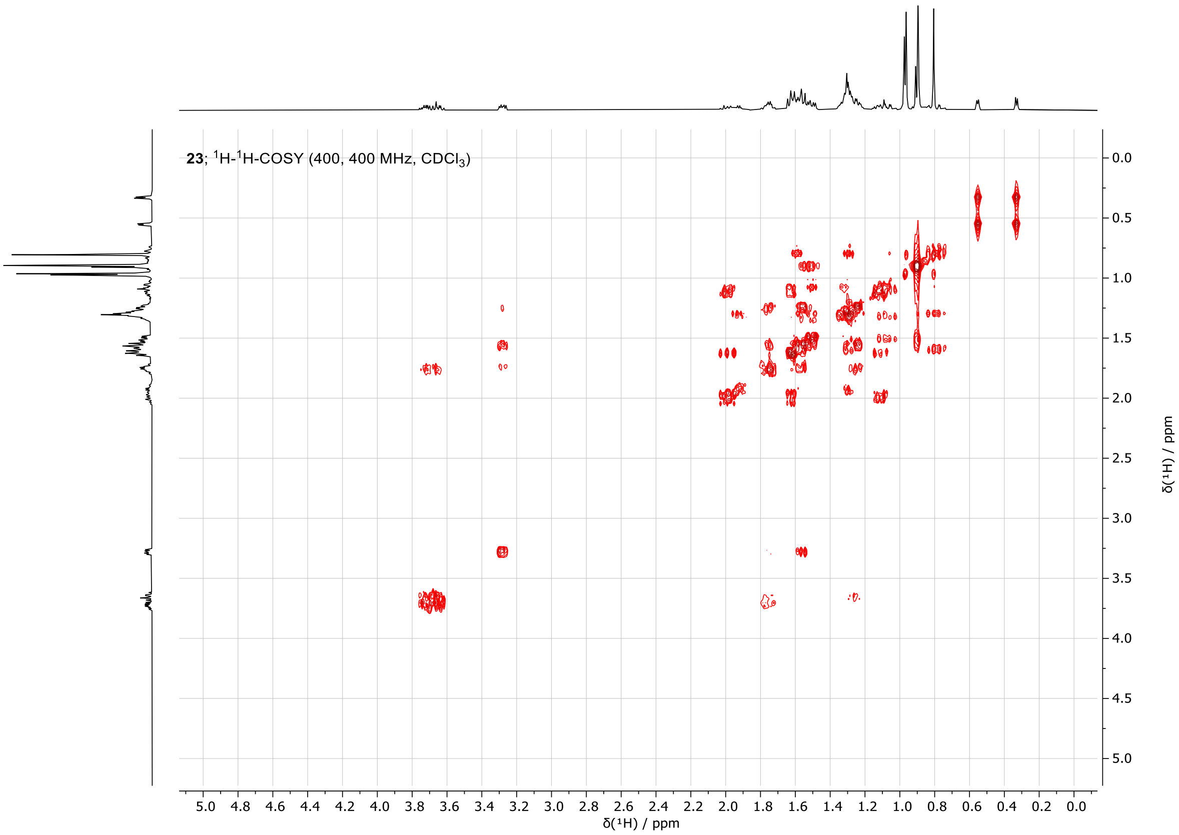

23; ^{1}H-^{1}H-COSY (400, 400 MHz, $CDCl_3$)

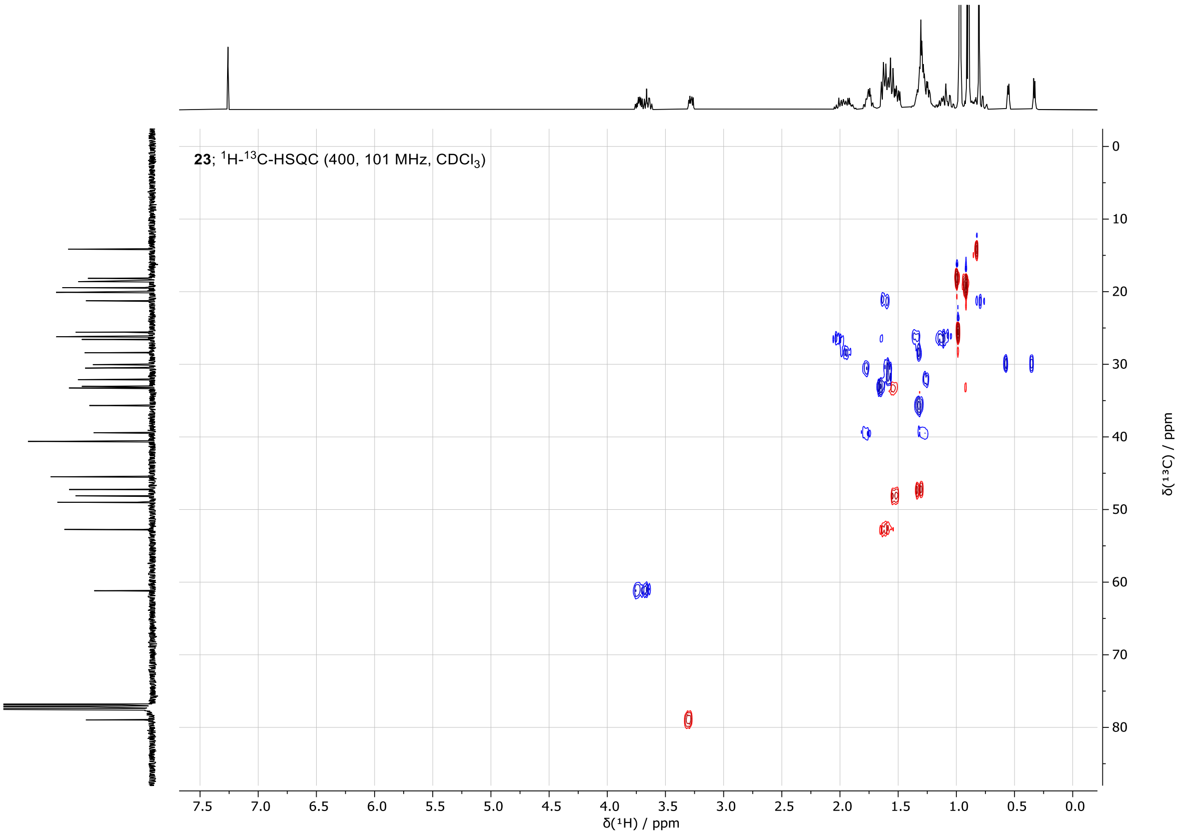
23; 1H-13C-HSQC (400, 101 MHz, CDCl3)
δ(¹H) / ppm
δ(¹³C) / ppm

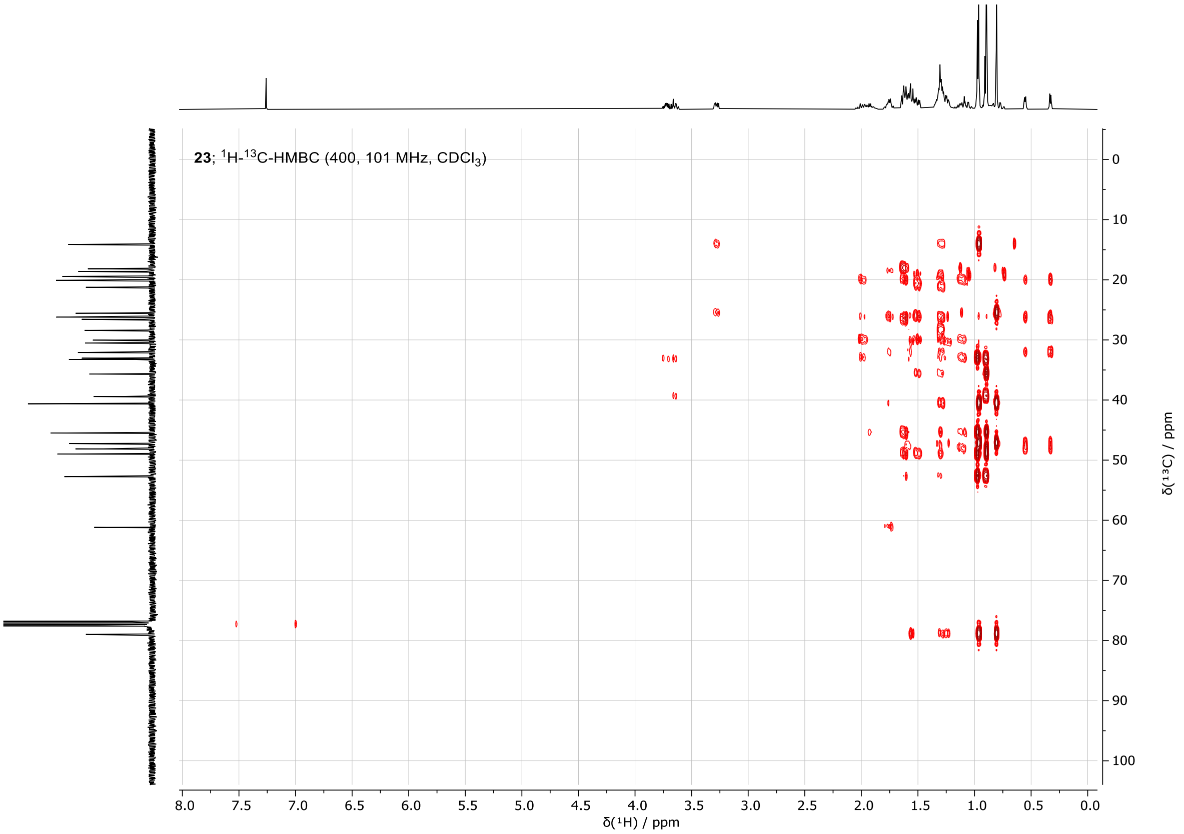
23; 1H-13C-HMBC (400, 101 MHz, CDCl3)
δ(¹H) / ppm
δ(¹³C) / ppm

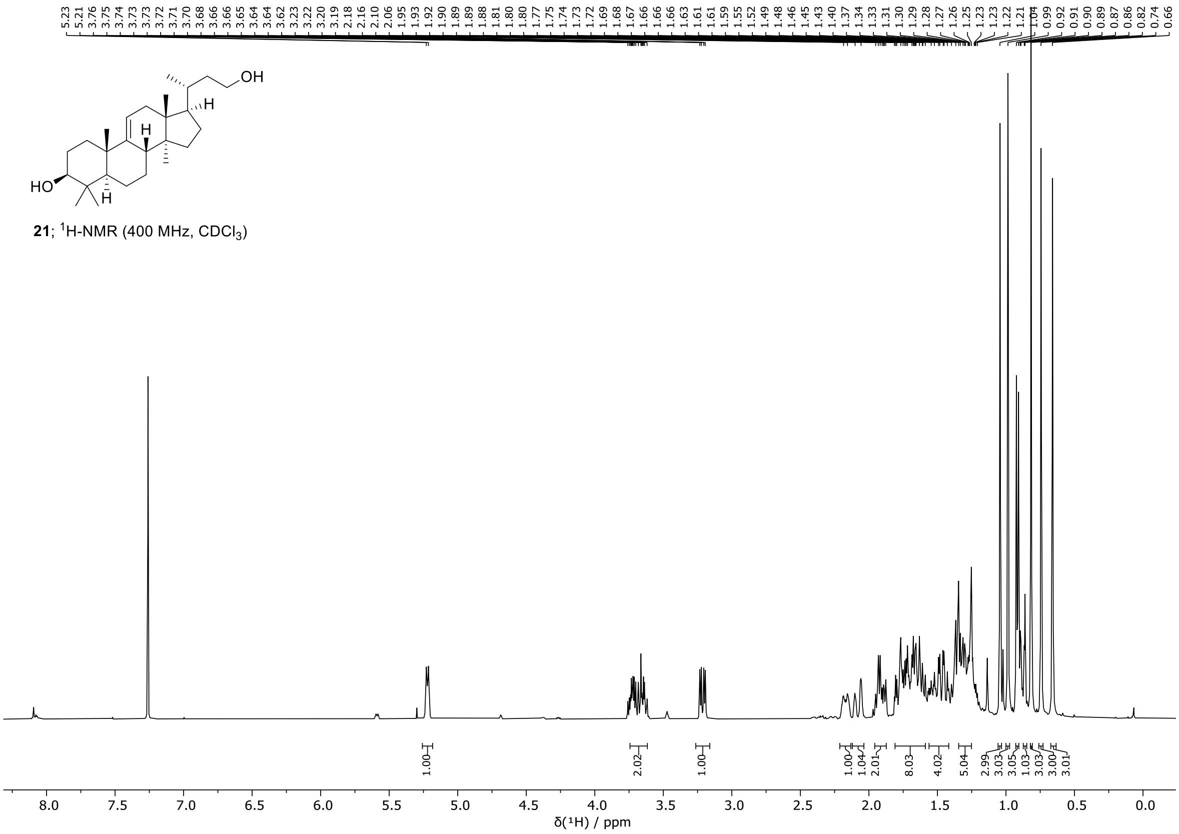

21; ^{1}H-NMR (400 MHz, $CDCl_3$)

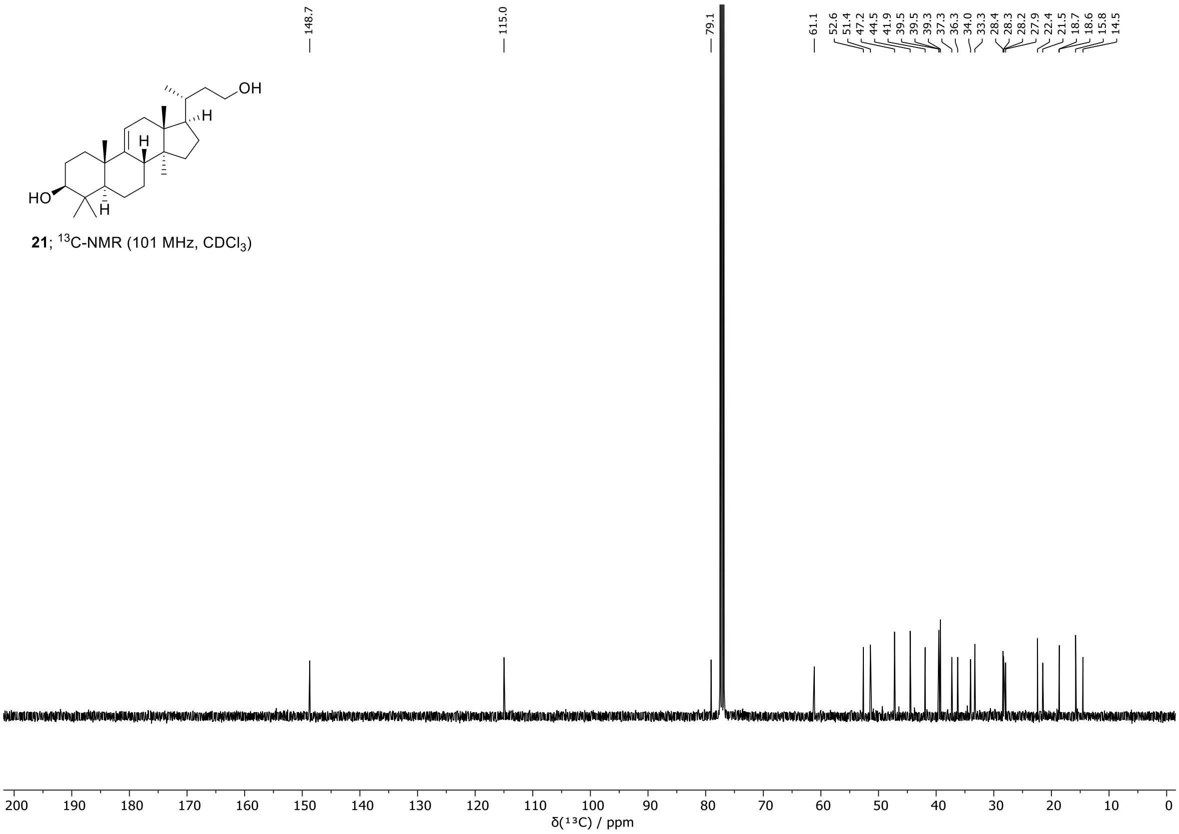

21; ^{13}C-NMR (101 MHz, $CDCl_3$)

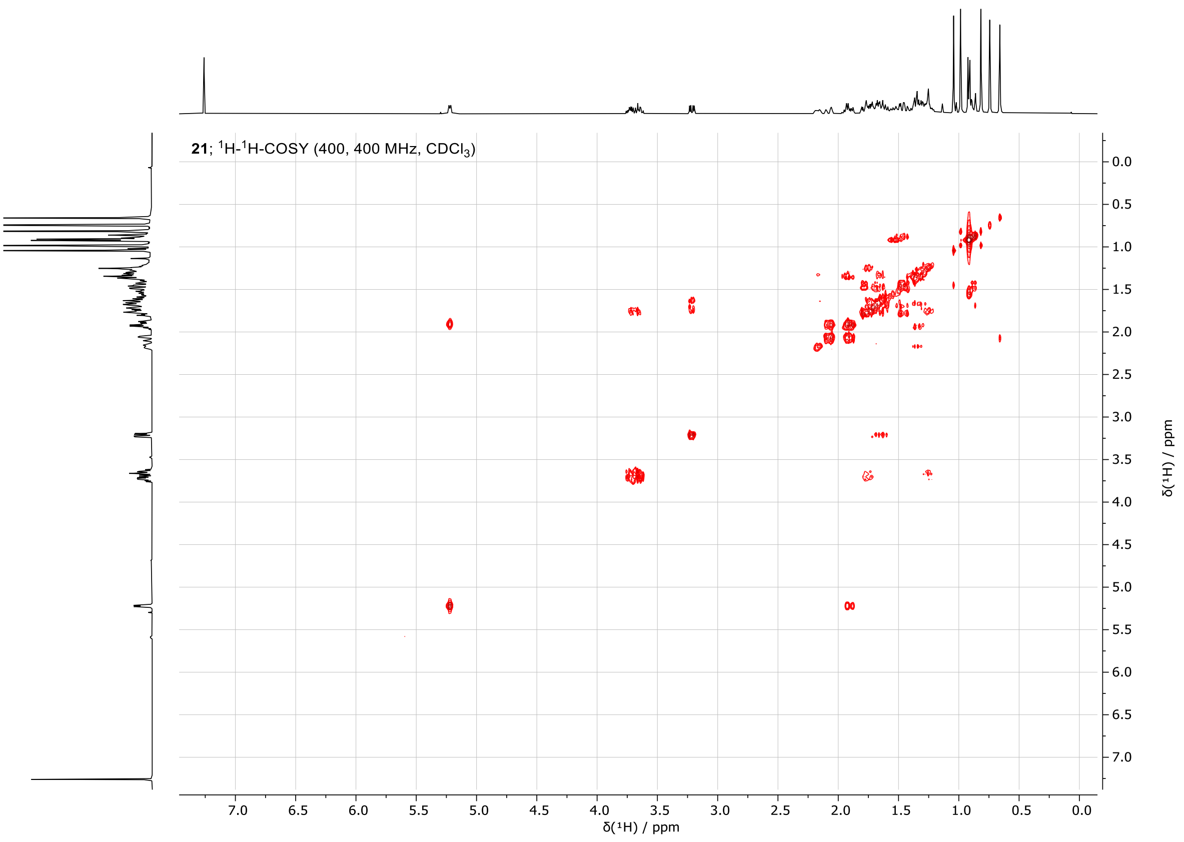

21; ^{1}H-^{1}H-COSY (400, 400 MHz, $CDCl_3$)
δ(¹H) / ppm
δ(¹H) / ppm

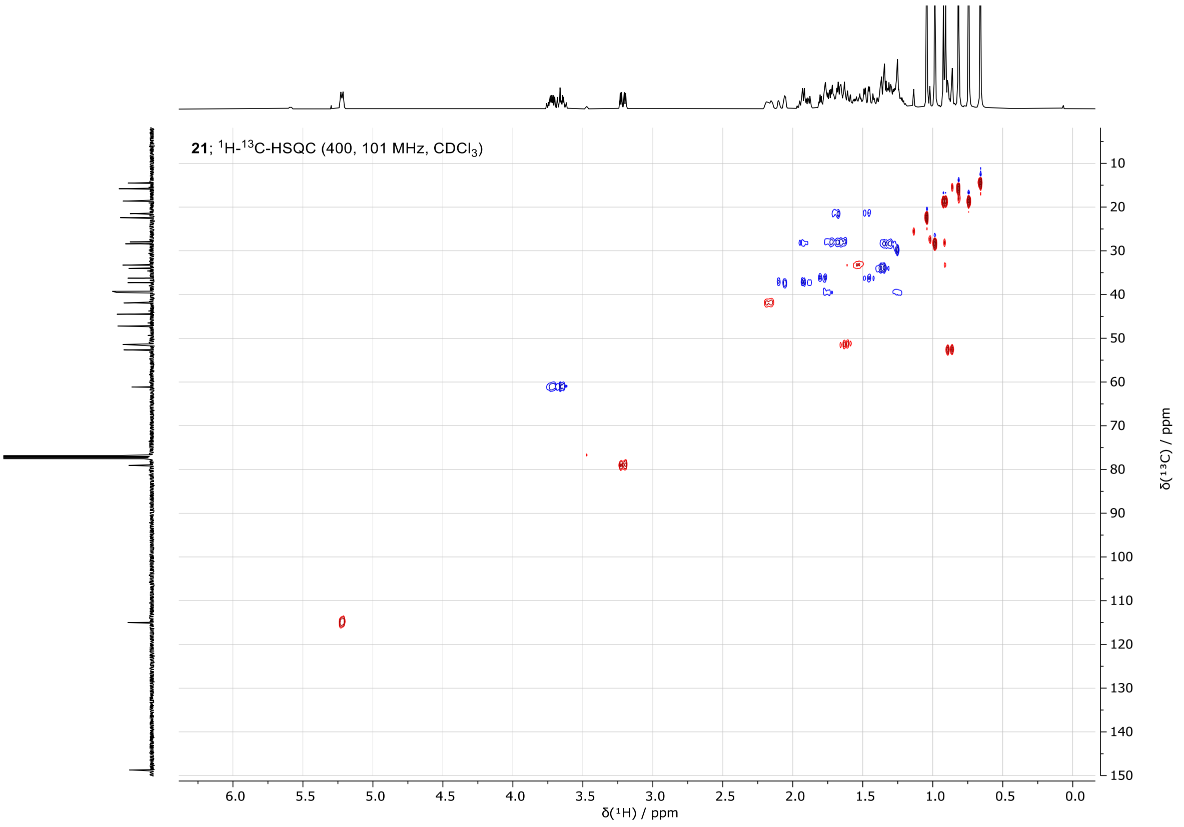

21; ^{1}H-^{13}C-HSQC (400, 101 MHz, $CDCl_3$)
δ(¹H) / ppm
δ(¹³C) / ppm

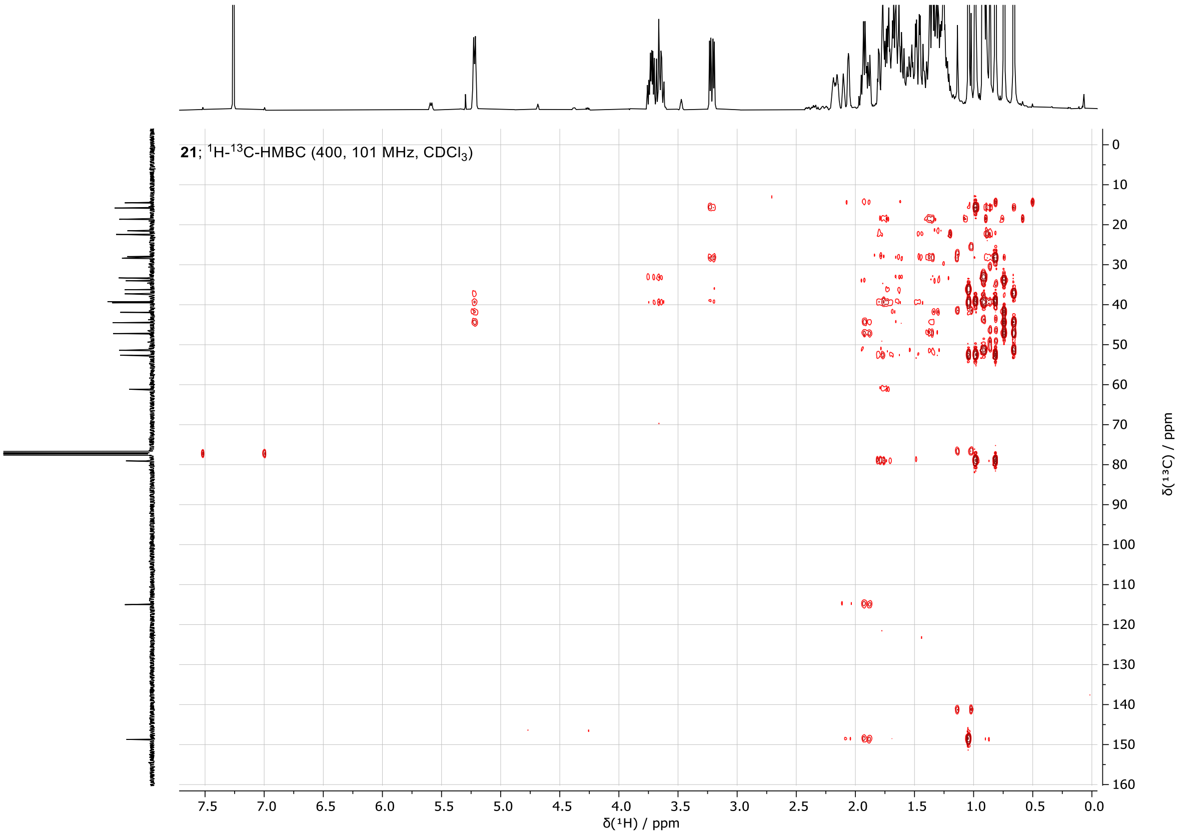

21; ^{1}H-^{13}C-HMBC (400, 101 MHz, $CDCl_3$)

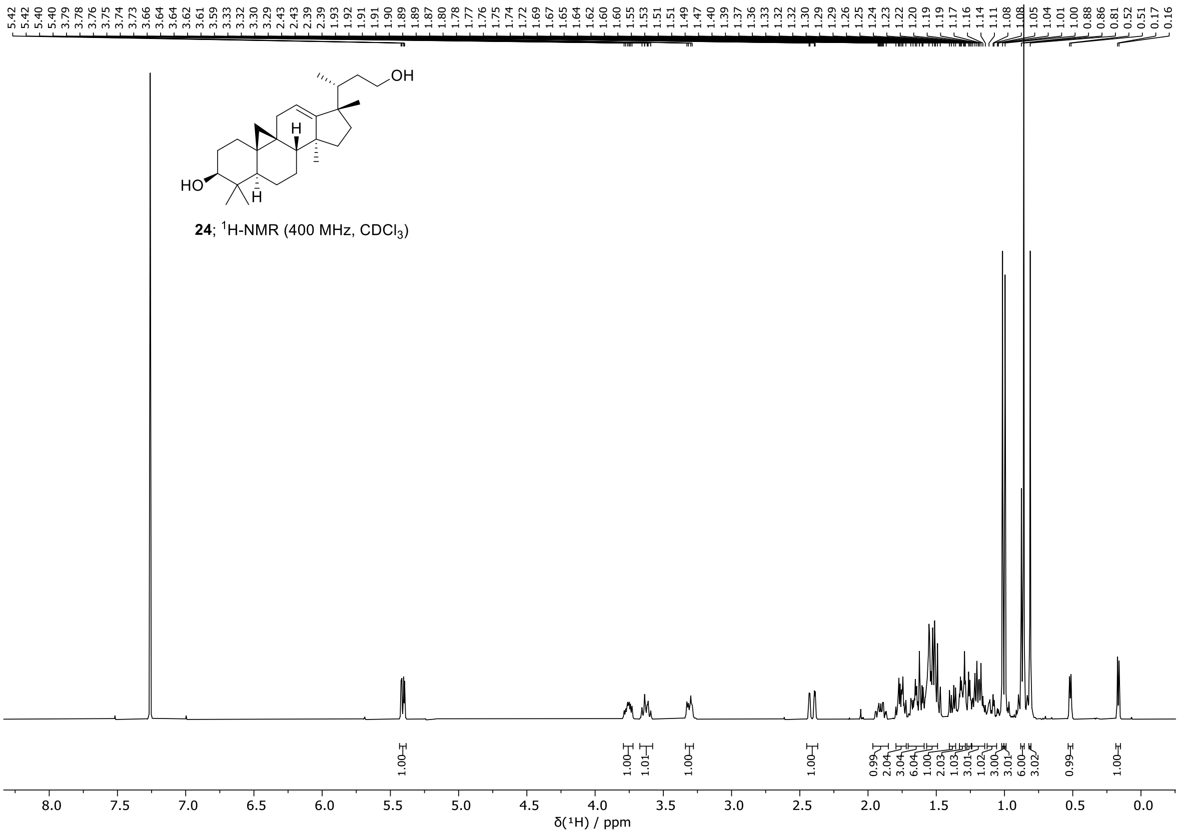

24; ^{1}H-NMR (400 MHz, $CDCl_3$)

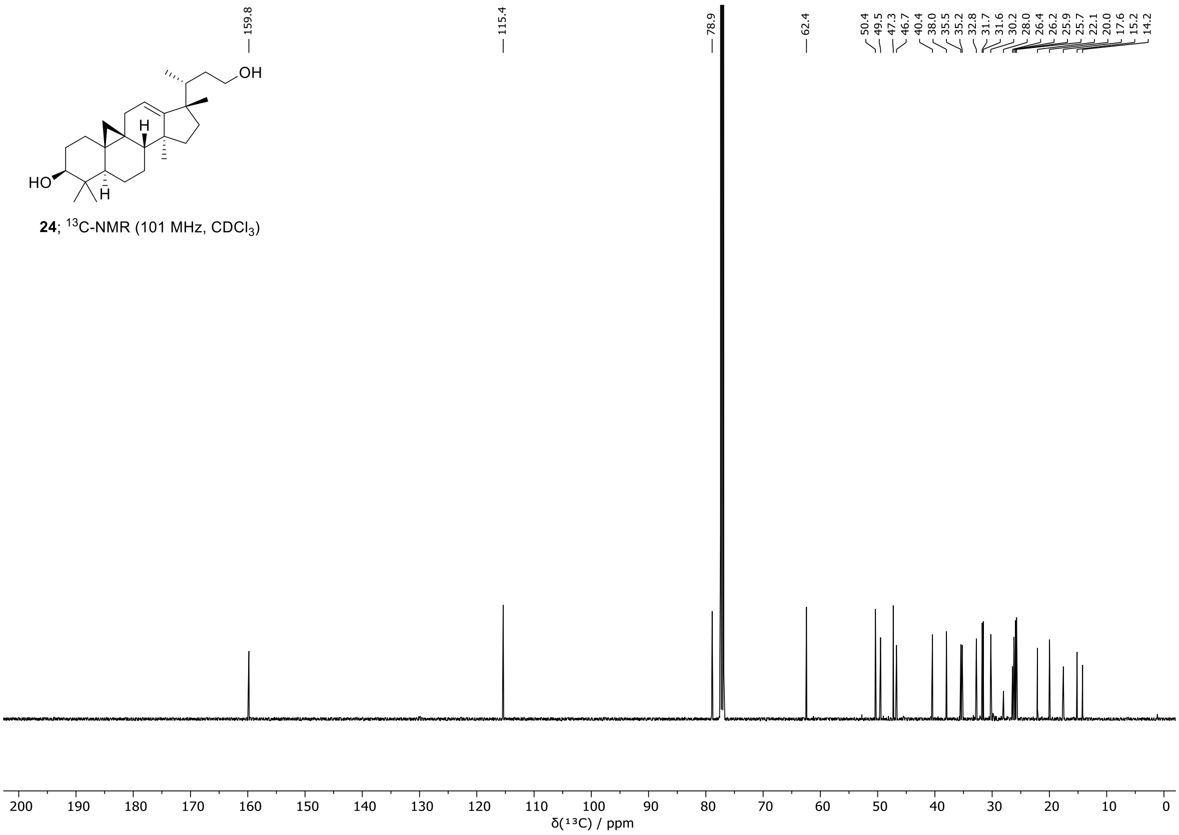
24; ^{13}C-NMR (101 MHz, CDCl$_3$)
HO
H
H
OH
159.8
115.4
78.9
62.4
50.4
49.5
47.3
46.7
40.4
38.0
35.5
35.2
32.8
31.7
31.6
30.2
28.0
26.4
26.2
25.9
25.7
22.1
20.0
17.6
15.2
14.2
δ(^{13}C) / ppm

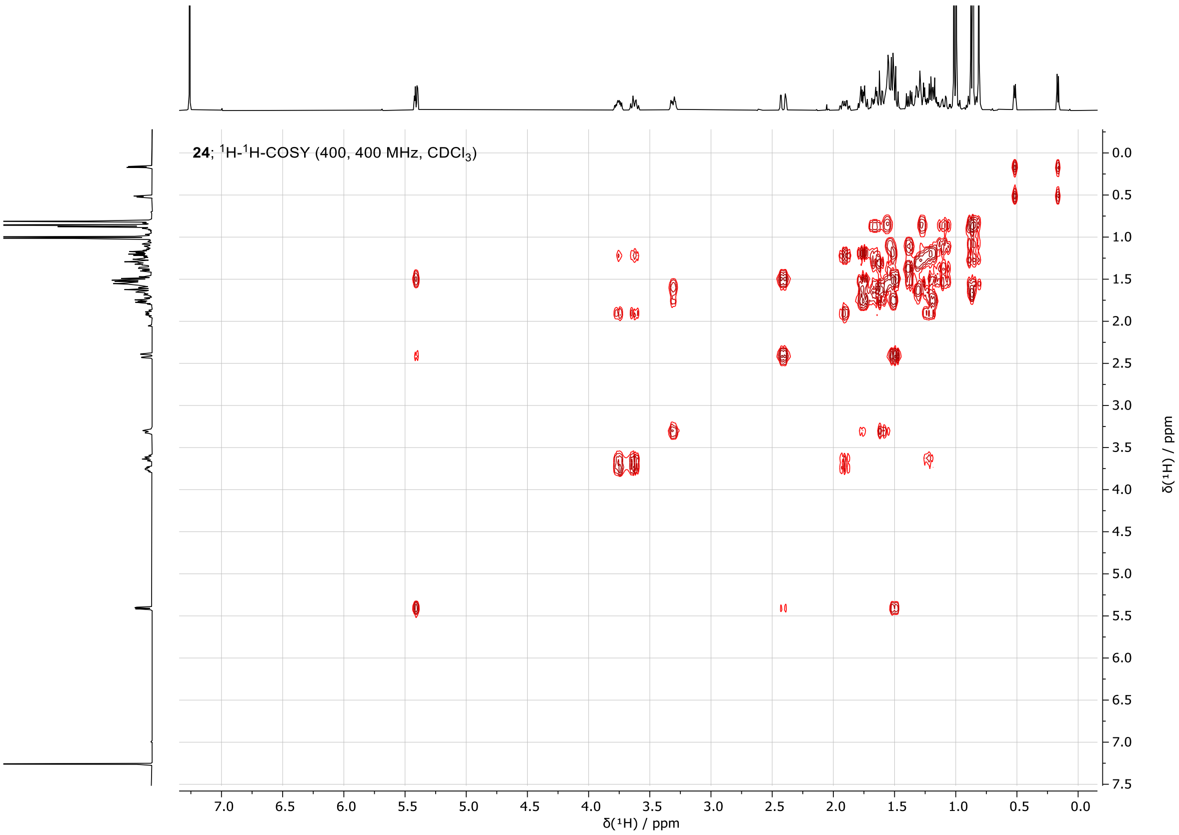
24; ^{1}H-^{1}H-COSY (400, 400 MHz, CDCl$_3$)
δ(^{1}H) / ppm
δ(^{1}H) / ppm

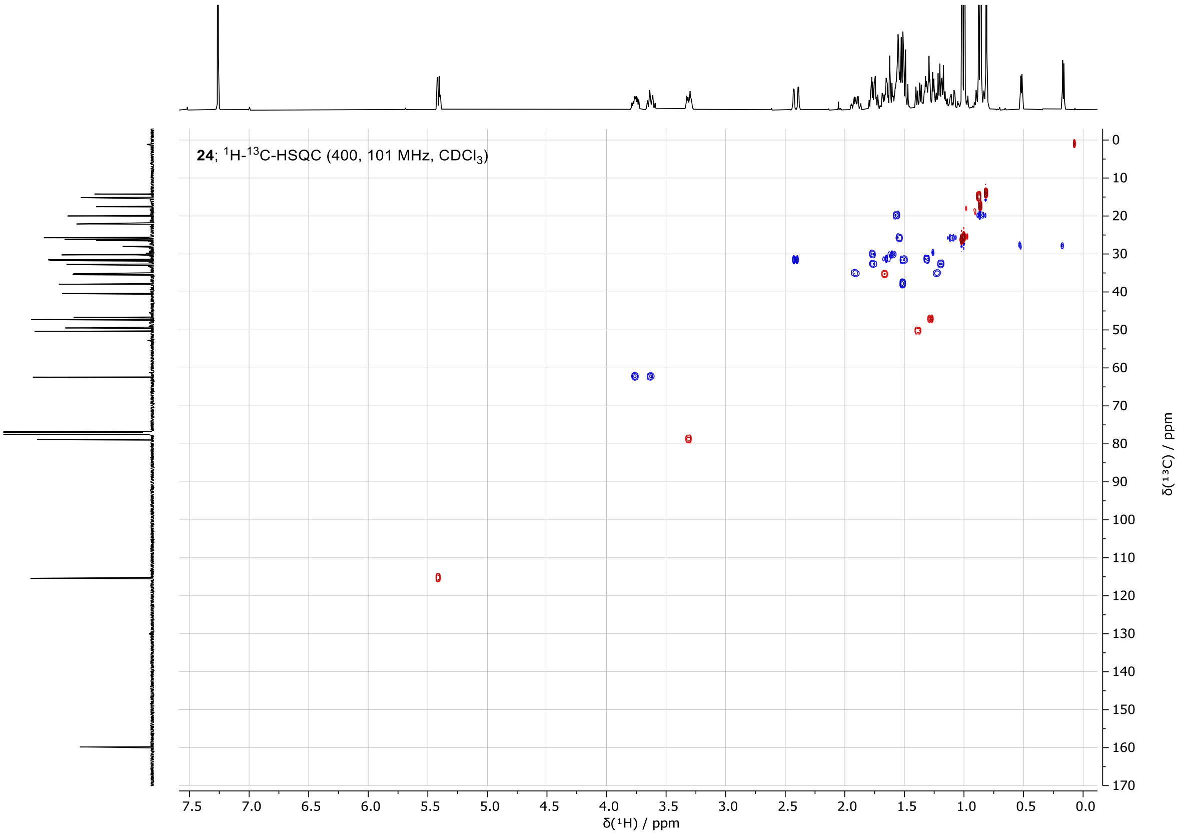
24; ^{1}H-^{13}C-HSQC (400, 101 MHz, CDCl$_3$)
δ(¹H) / ppm
δ(¹³C) / ppm

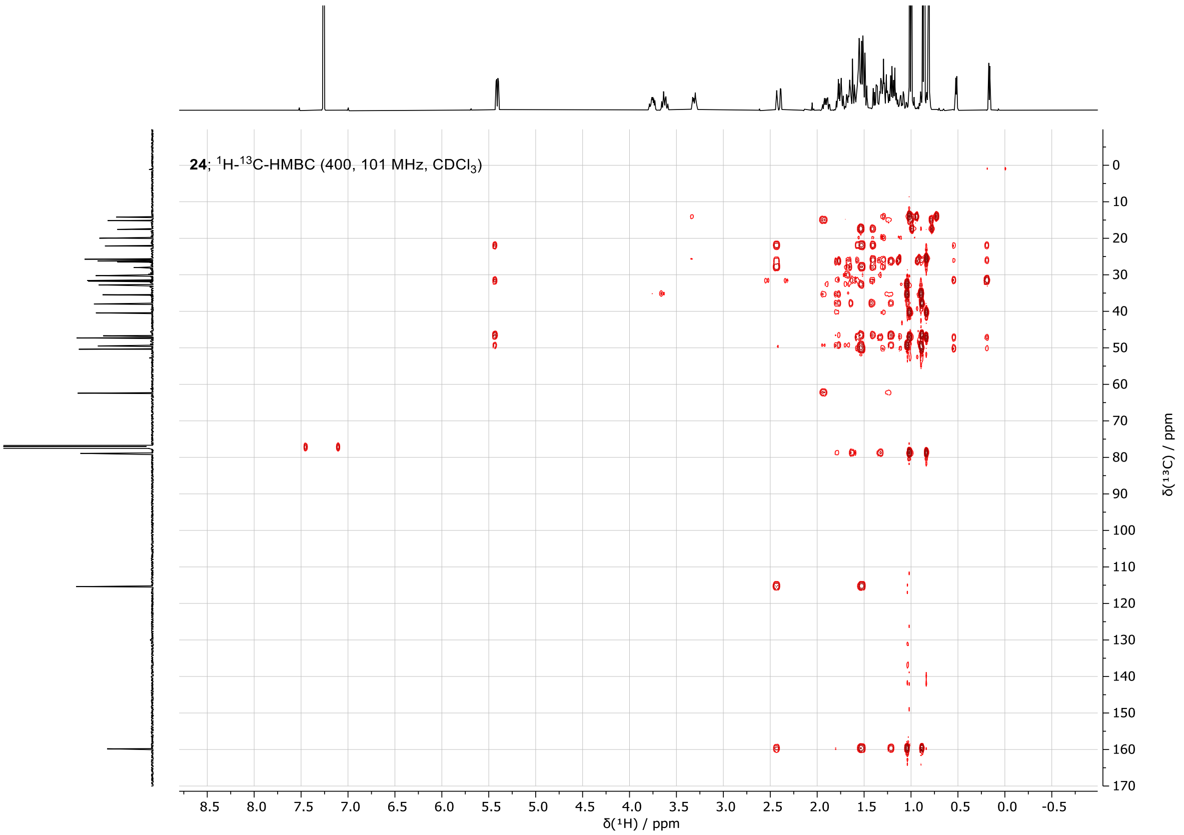
24; ^{1}H-^{13}C-HMBC (400, 101 MHz, CDCl$_3$)
δ(¹H) / ppm
δ(¹³C) / ppm

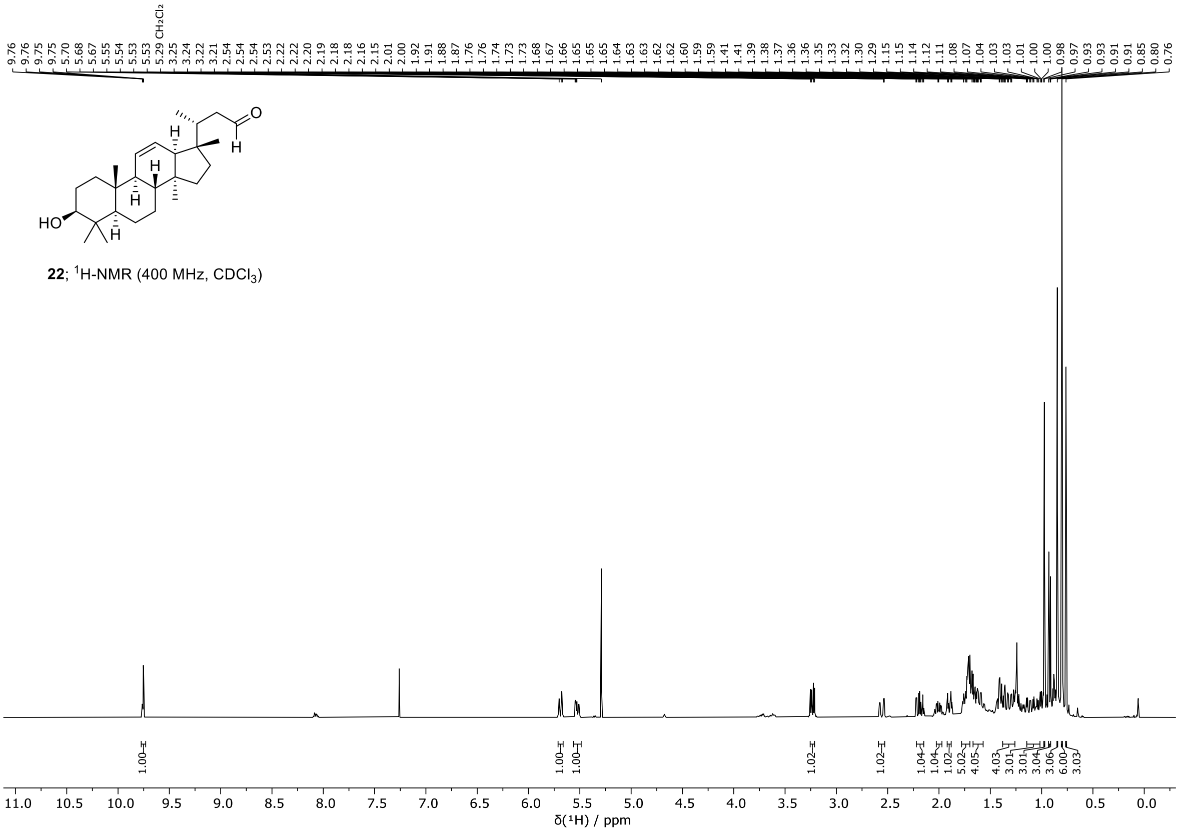

22; ^{1}H-NMR (400 MHz, $CDCl_3$)

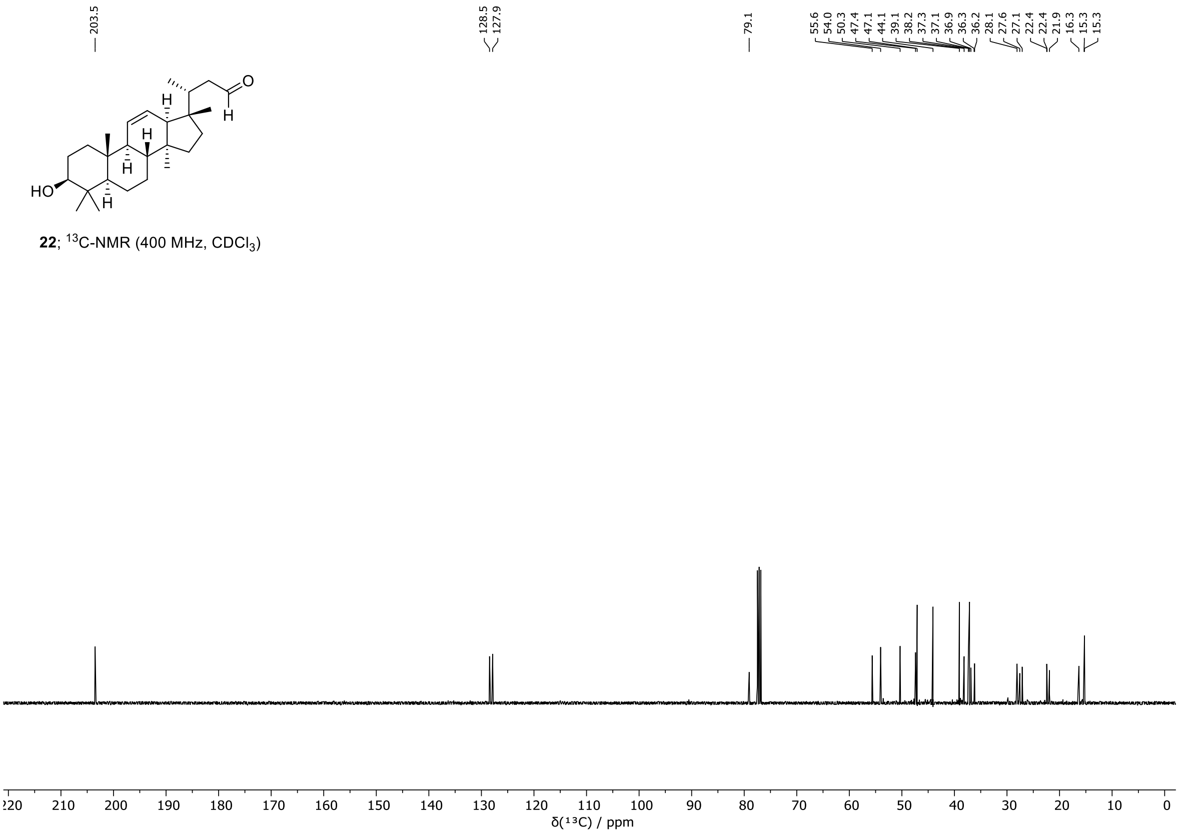

22; ^{13}C-NMR (400 MHz, $CDCl_3$)

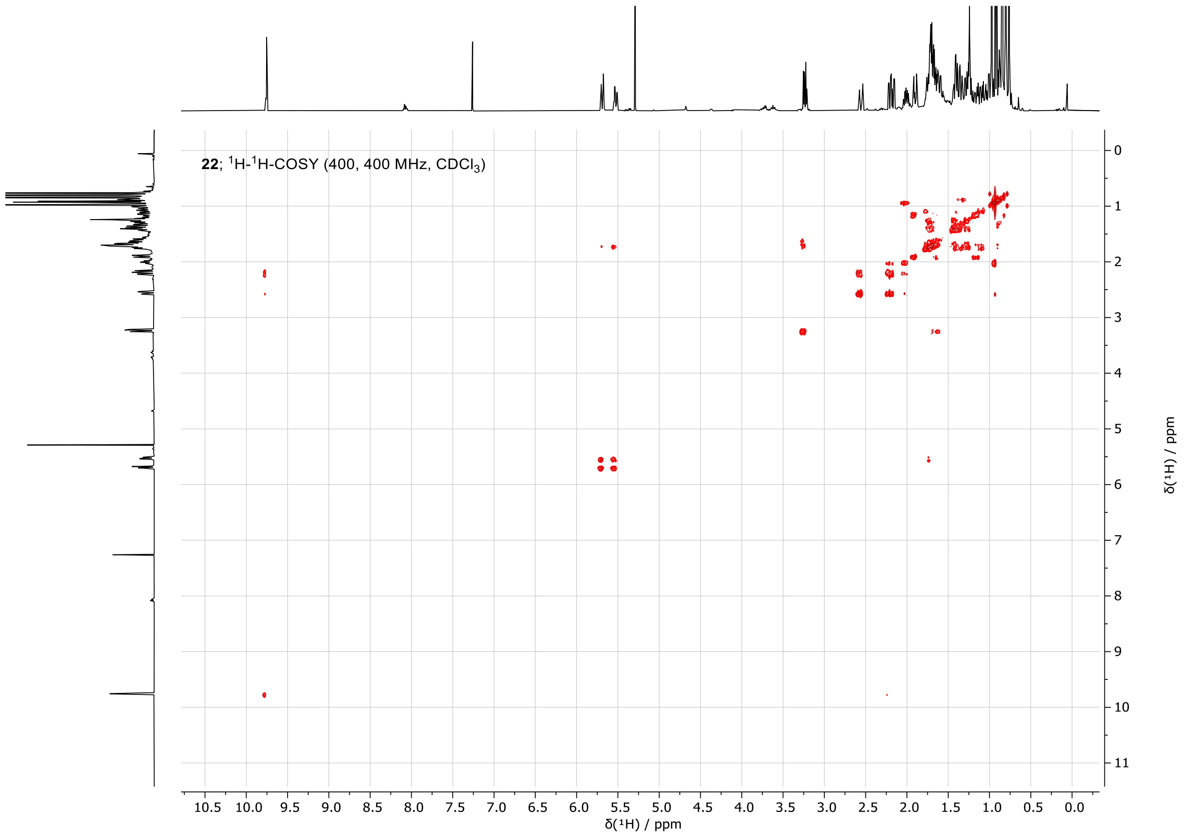
22; 1H-1H-COSY (400, 400 MHz, CDCl3)
δ(¹H) / ppm
δ(¹H) / ppm

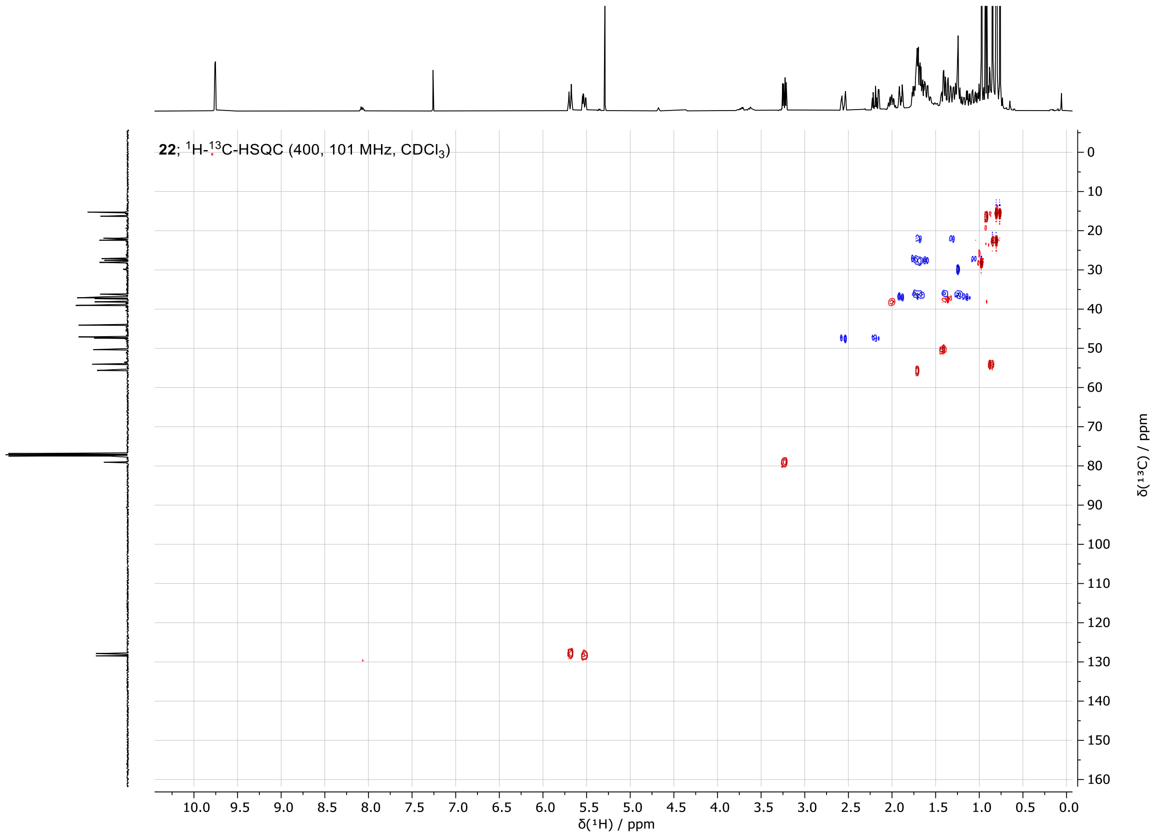
22; 1H-13C-HSQC (400, 101 MHz, CDCl3)
δ(¹H) / ppm
δ(¹³C) / ppm

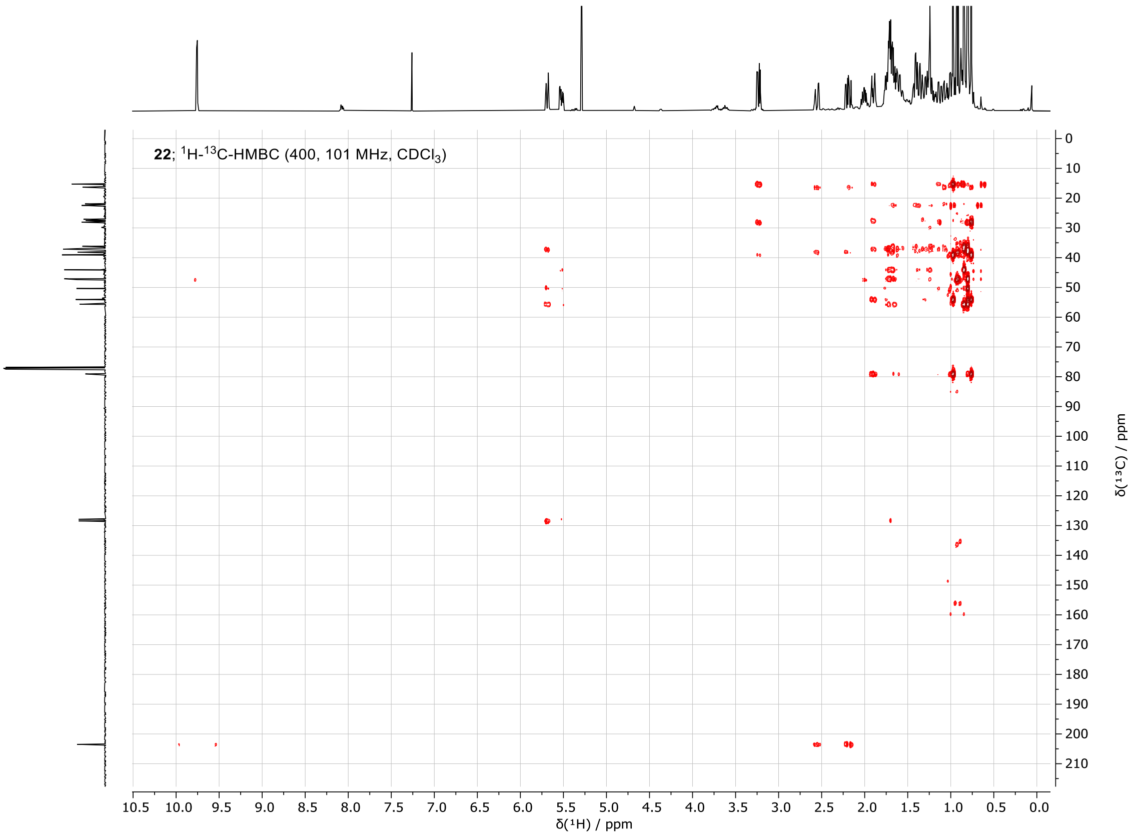

22; 1H-13C-HMBC (400, 101 MHz, CDCl3)
δ(1H) / ppm
δ(13C) / ppm

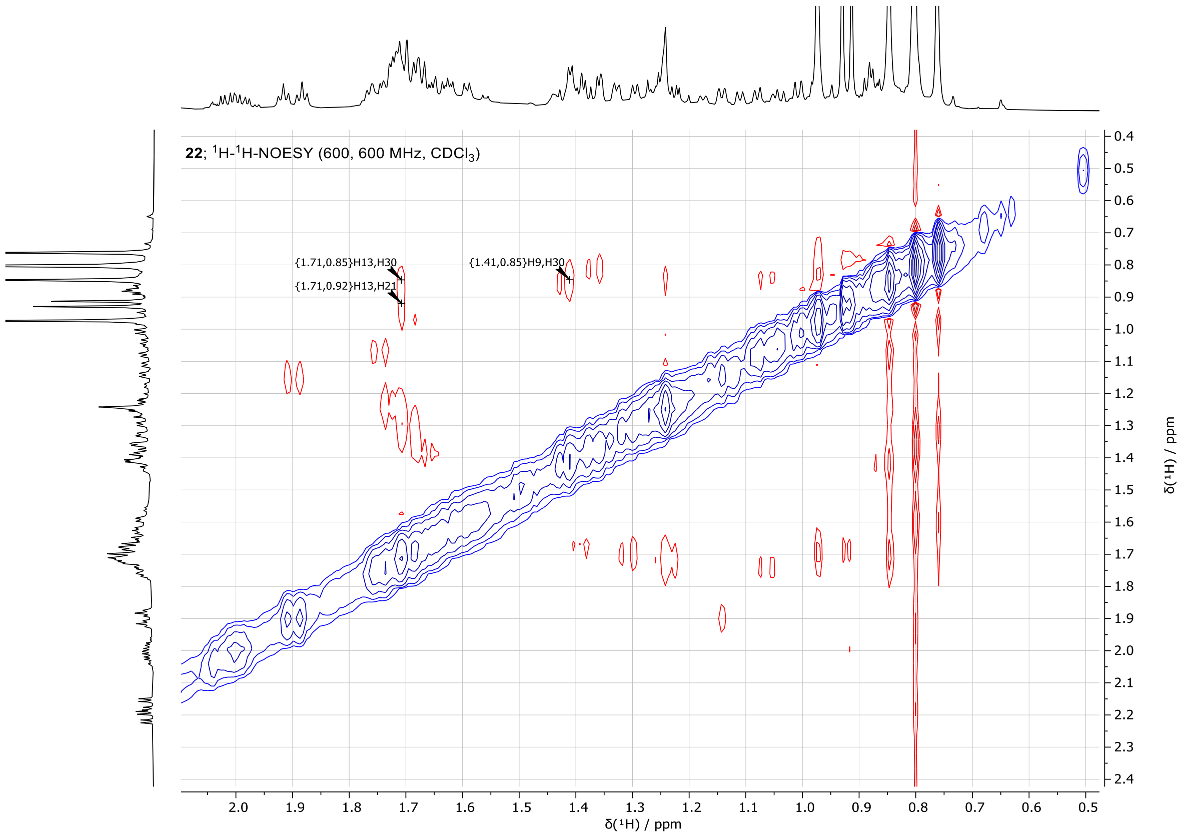

22; 1H-1H-NOESY (600, 600 MHz, CDCl3)
{1.71,0.85}H13,H30
{1.71,0.92}H13,H21
{1.41,0.85}H9,H30
δ(1H) / ppm
δ(1H) / ppm

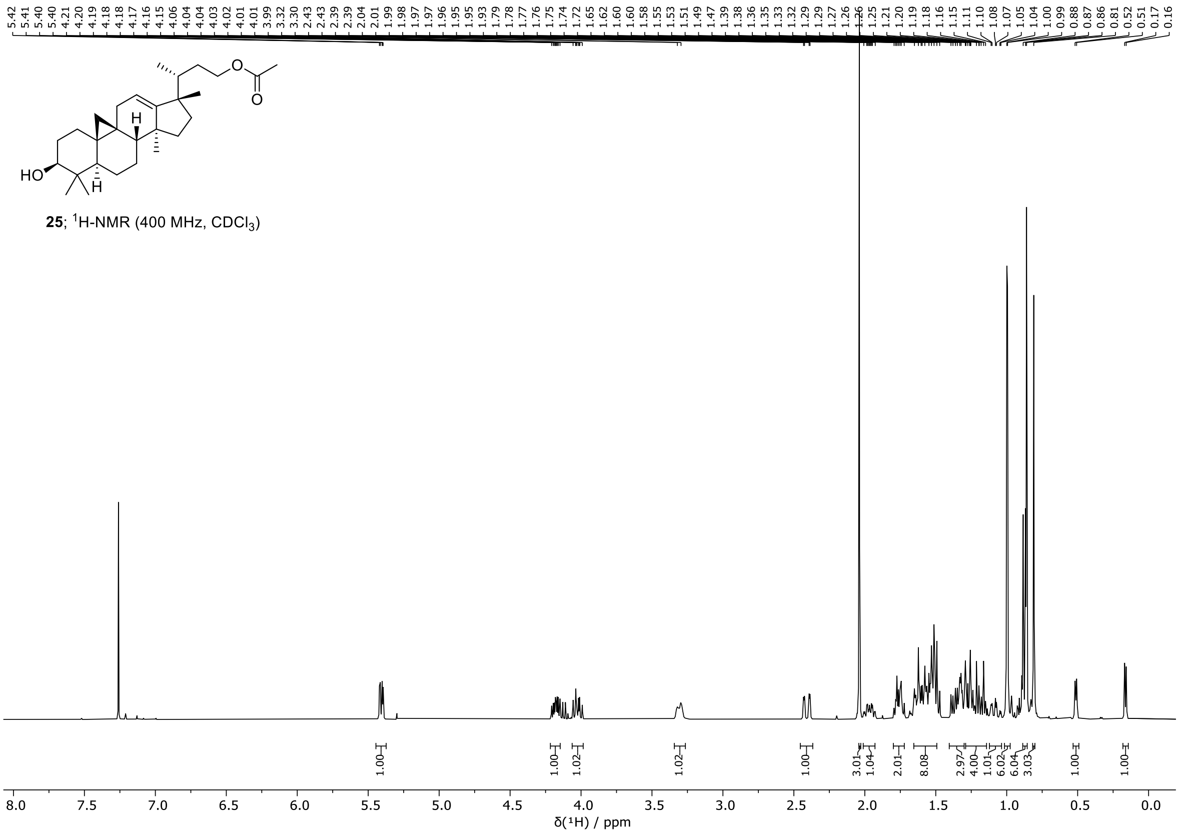

25; ^{1}H-NMR (400 MHz, $CDCl_3$)

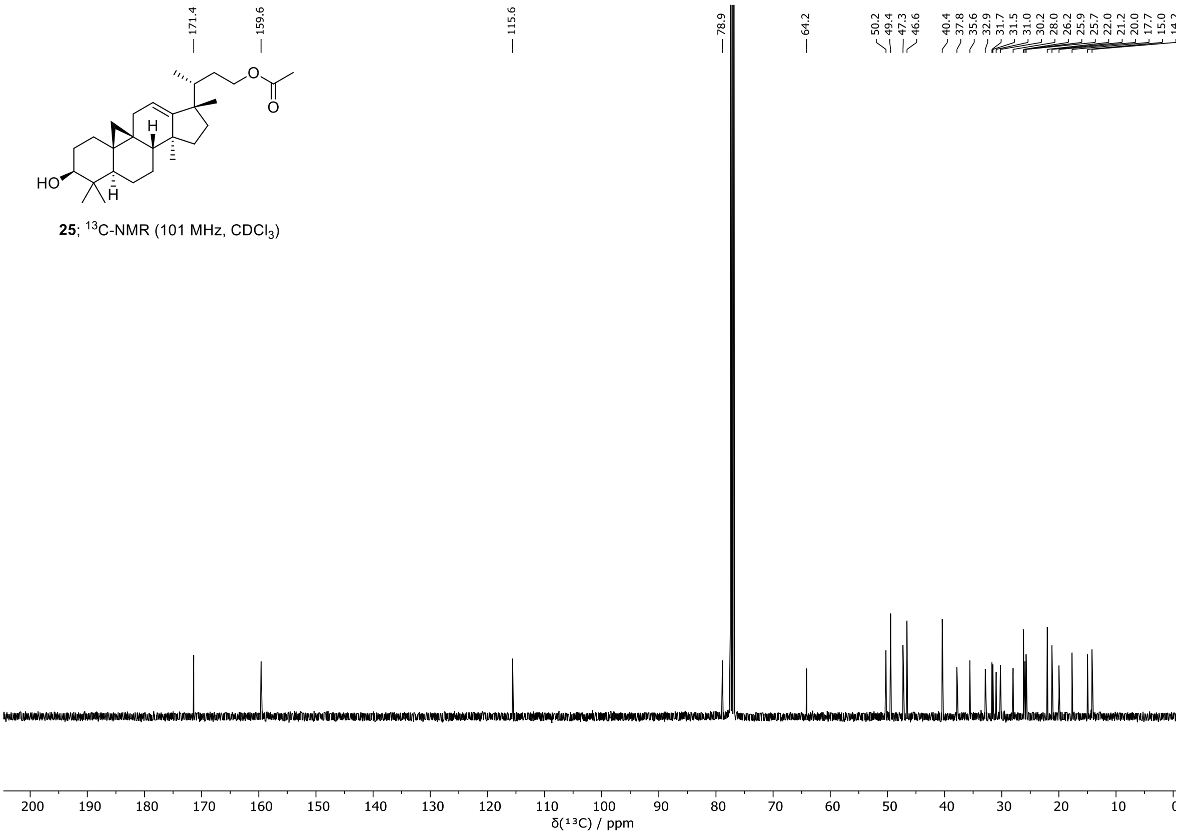

25; ^{13}C-NMR (101 MHz, $CDCl_3$)

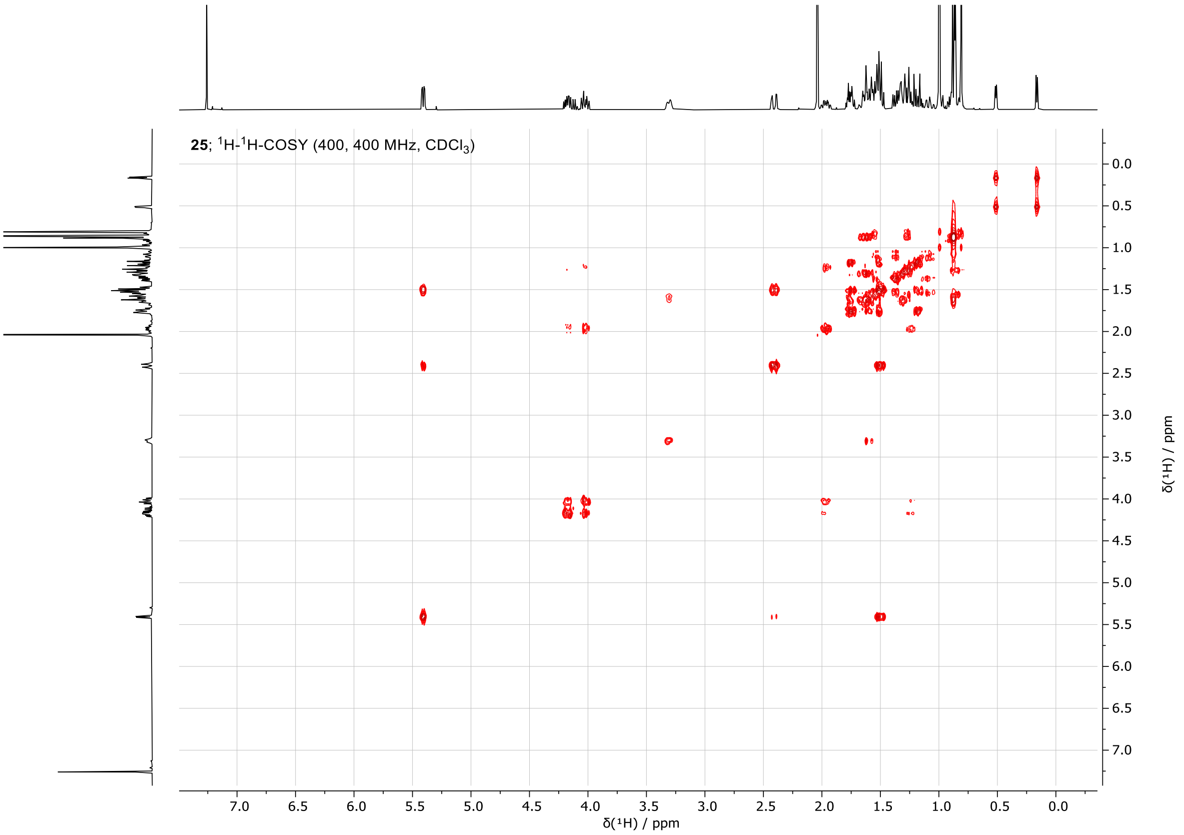

25; ^{1}H-^{1}H-COSY (400, 400 MHz, CDCl$_3$)
δ(¹H) / ppm
δ(¹H) / ppm

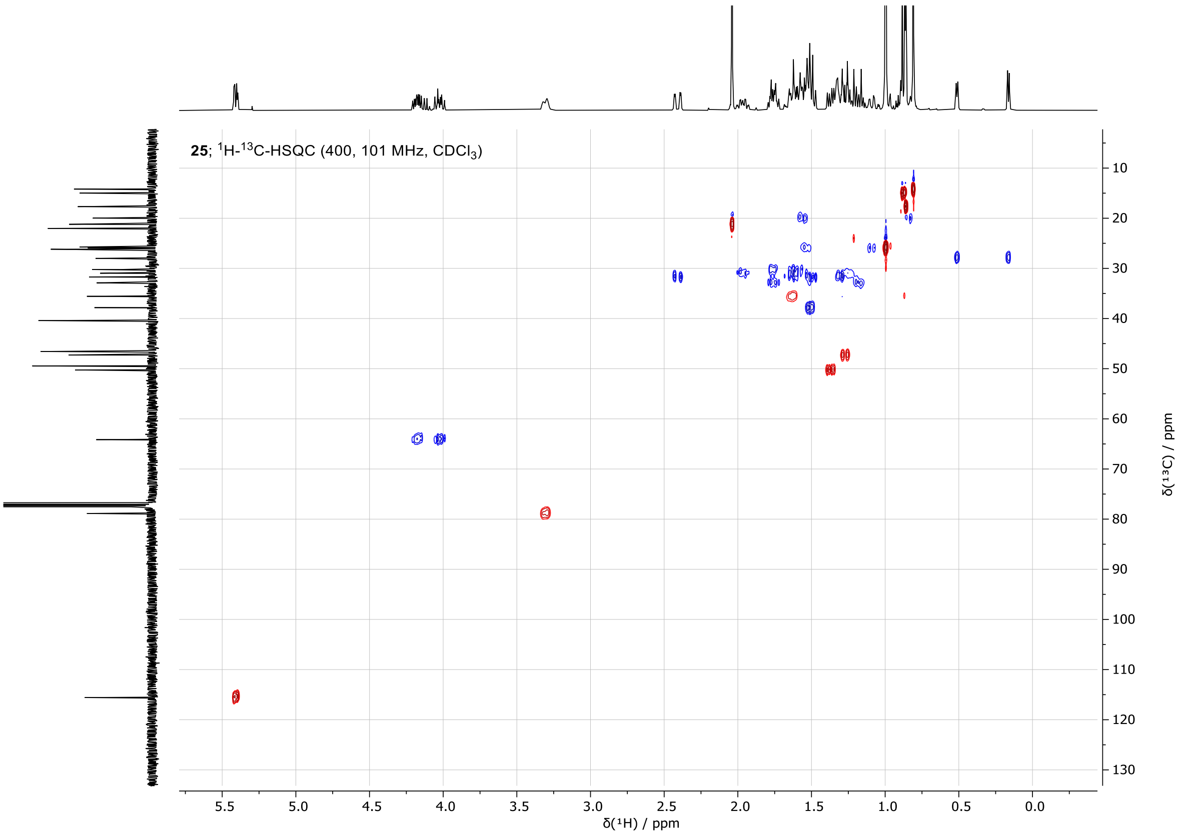

25; ^{1}H-^{13}C-HSQC (400, 101 MHz, CDCl$_3$)
δ(¹H) / ppm
δ(¹³C) / ppm

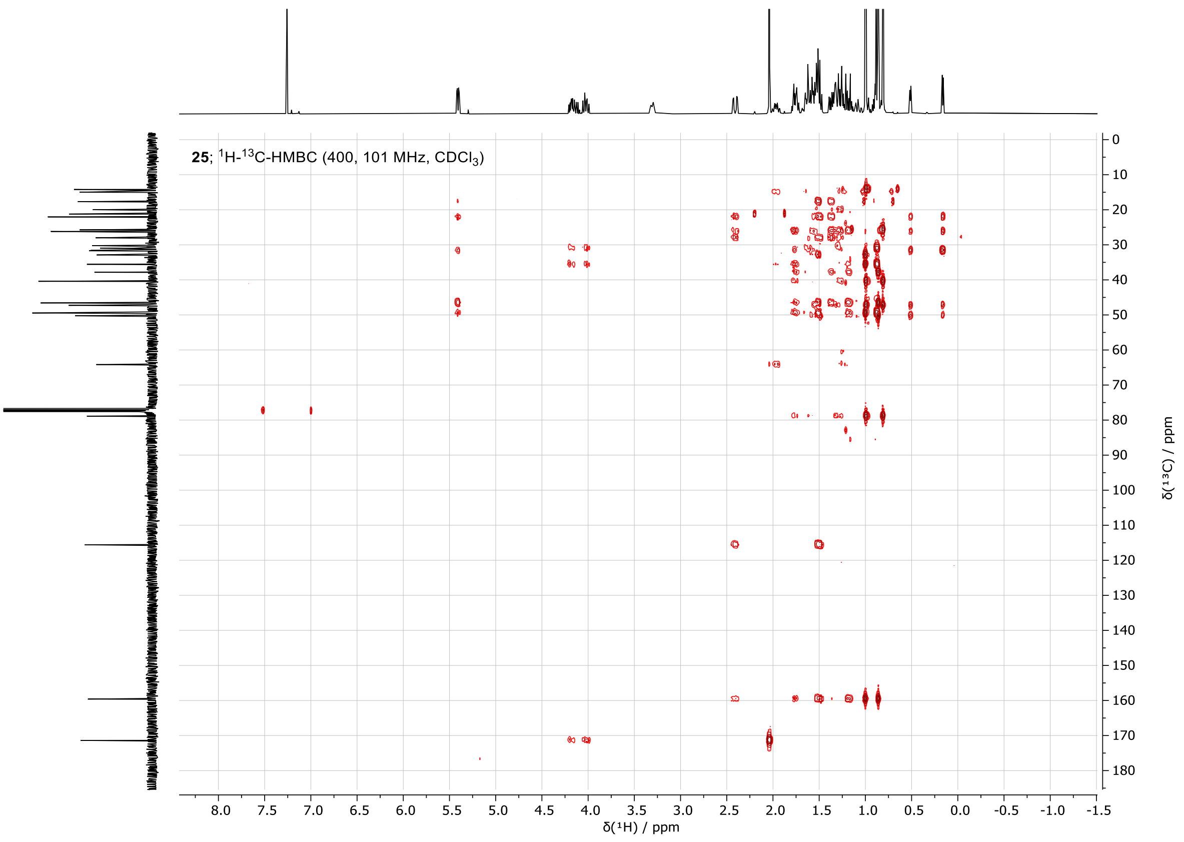

25; ^{1}H-^{13}C-HMBC (400, 101 MHz, CDCl$_3$)

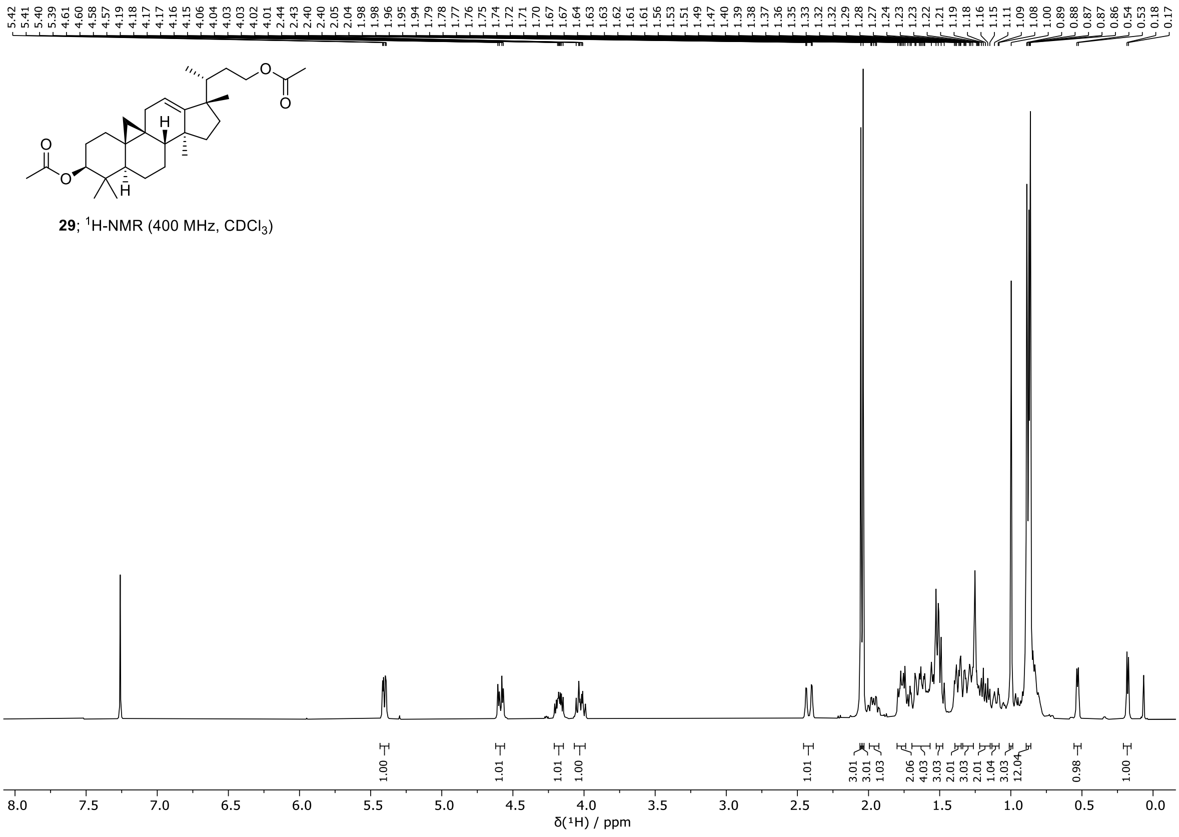

29; ^{1}H-NMR (400 MHz, CDCl$_3$)

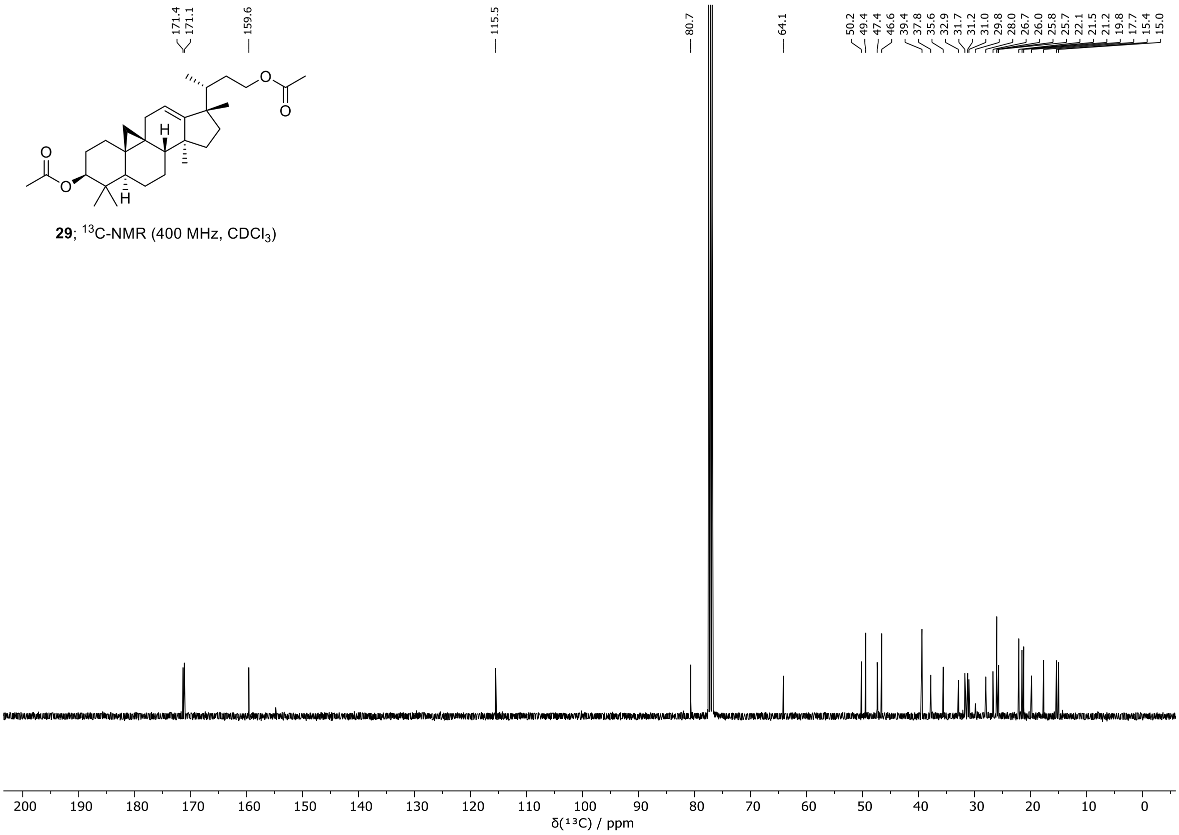
171.4
171.1
159.6
115.5
80.7
64.1
50.2
49.4
47.4
46.6
39.4
37.8
35.6
32.9
31.7
31.2
31.0
29.8
28.0
26.7
26.0
25.8
25.7
22.1
21.5
21.2
19.8
17.7
15.4
15.0
29; 13C-NMR (400 MHz, CDCl3)
200 190 180 170 160 150 140 130 120 110 100 90 80 70 60 50 40 30 20 10 0
δ(13C) / ppm

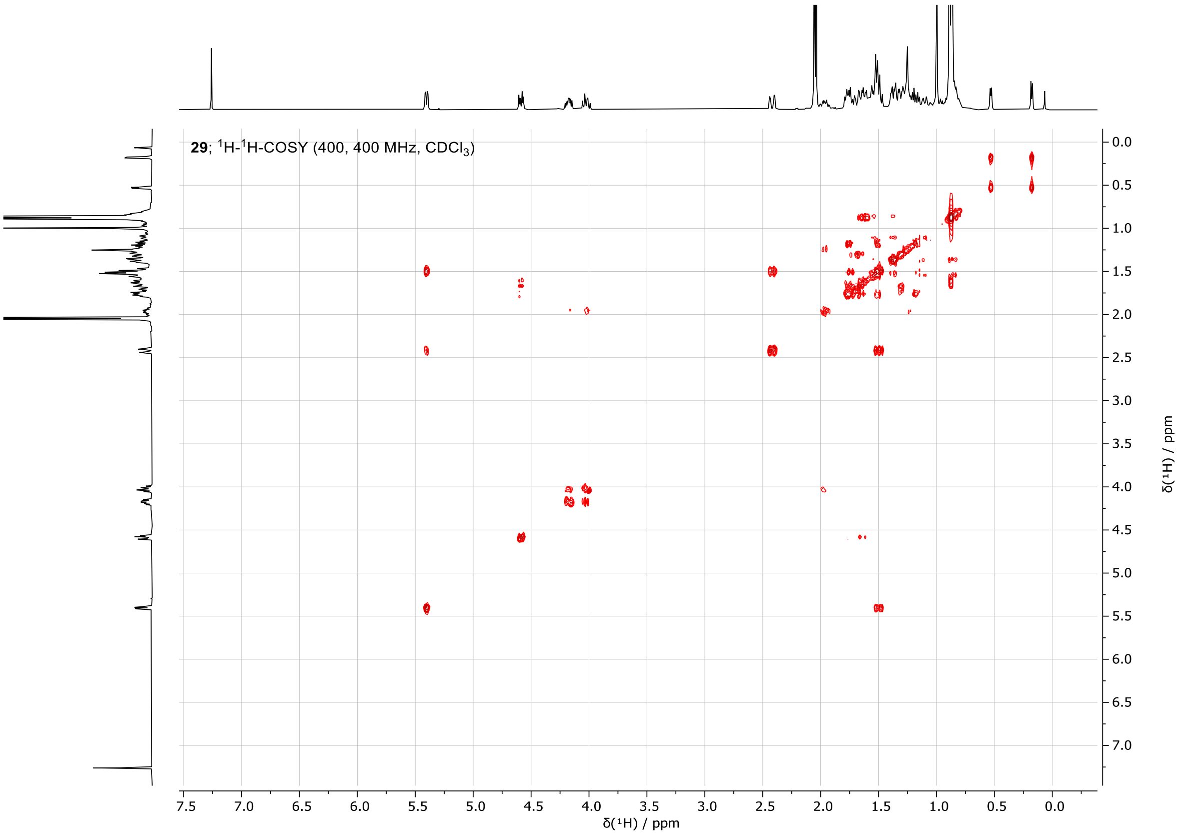
29; 1H-1H-COSY (400, 400 MHz, CDCl3)
7.5 7.0 6.5 6.0 5.5 5.0 4.5 4.0 3.5 3.0 2.5 2.0 1.5 1.0 0.5 0.0
δ(1H) / ppm
0.0 0.5 1.0 1.5 2.0 2.5 3.0 3.5 4.0 4.5 5.0 5.5 6.0 6.5 7.0
δ(1H) / ppm

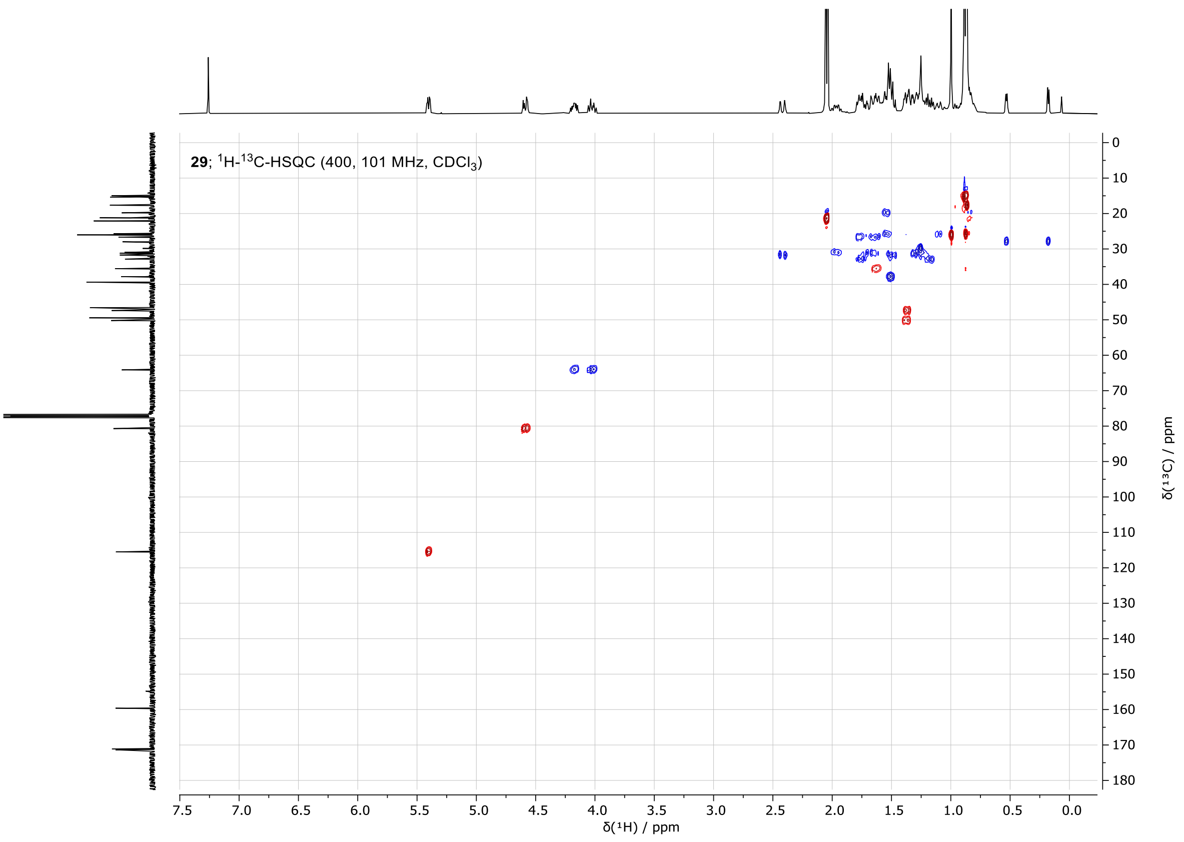
29; 1H-13C-HSQC (400, 101 MHz, CDCl3)
δ(¹H) / ppm
δ(¹³C) / ppm

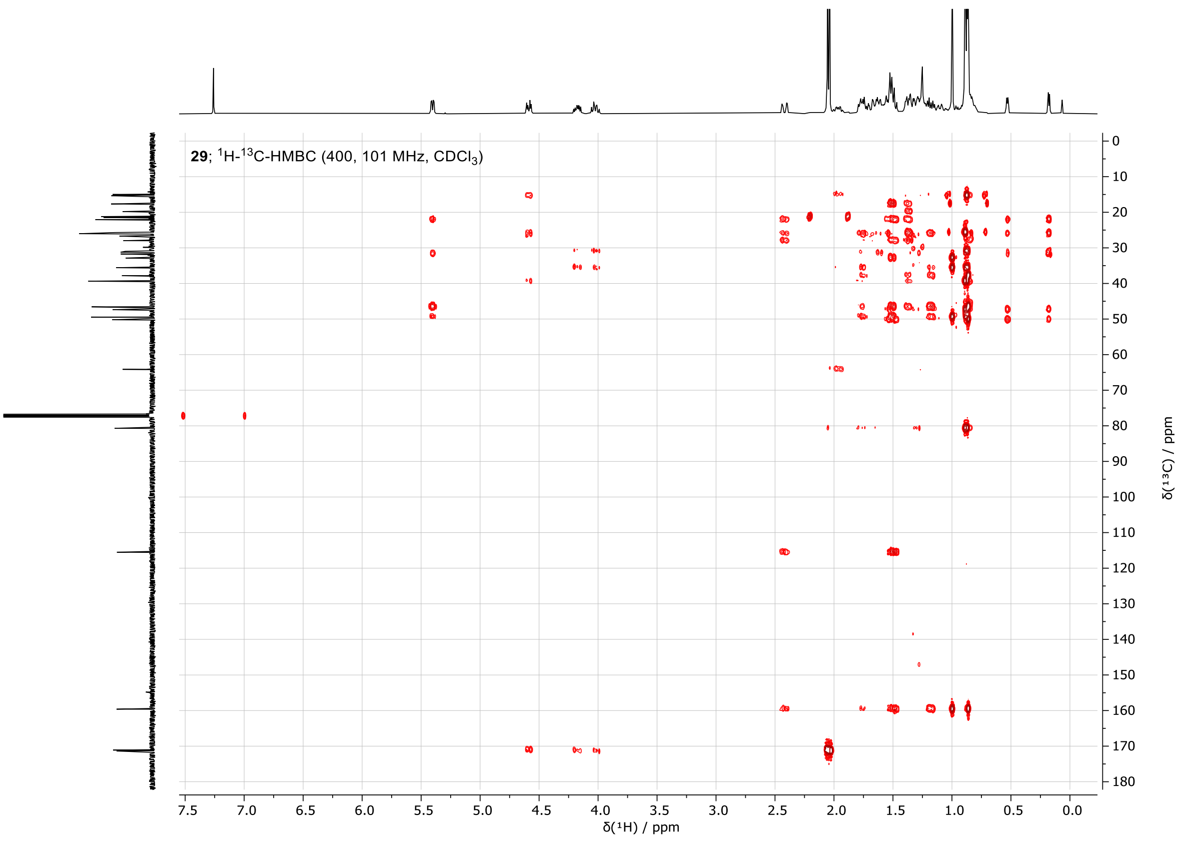
29; 1H-13C-HMBC (400, 101 MHz, CDCl3)
δ(¹H) / ppm
δ(¹³C) / ppm

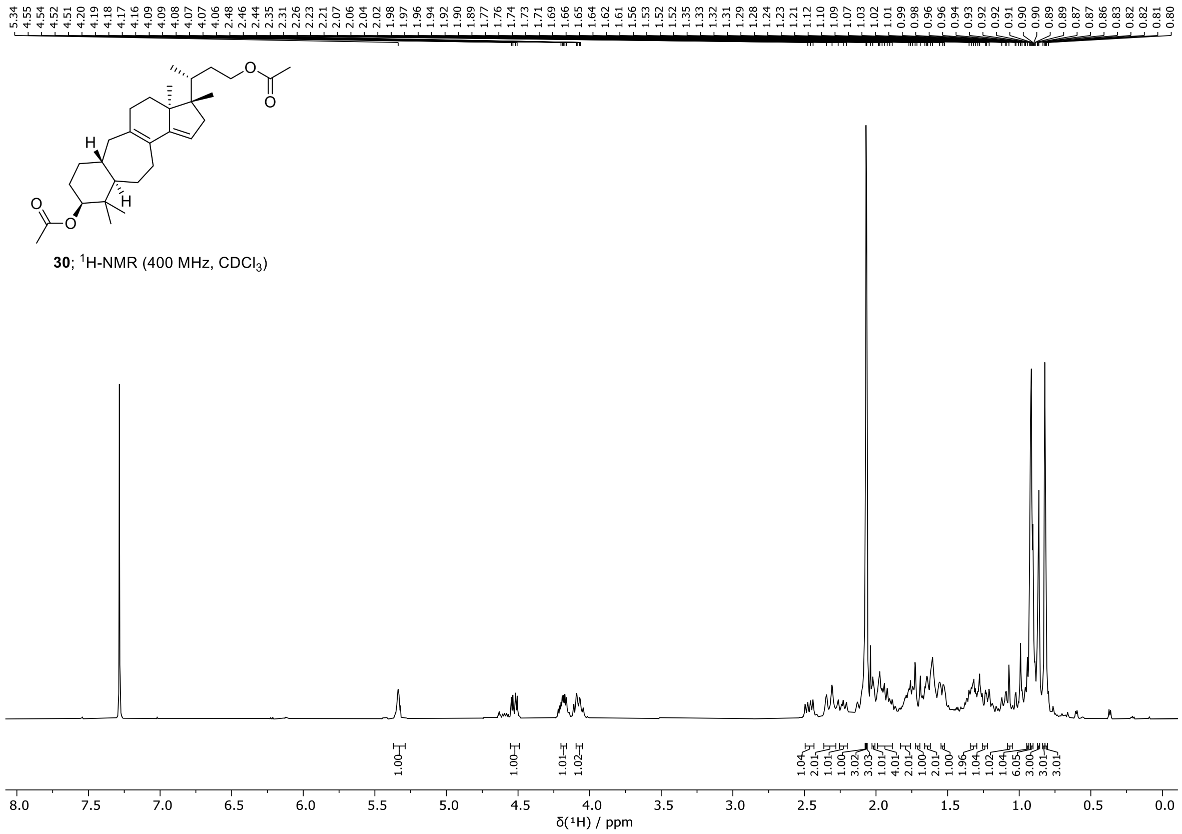
30; ^{1}H-NMR (400 MHz, $CDCl_3$)
δ(¹H) / ppm

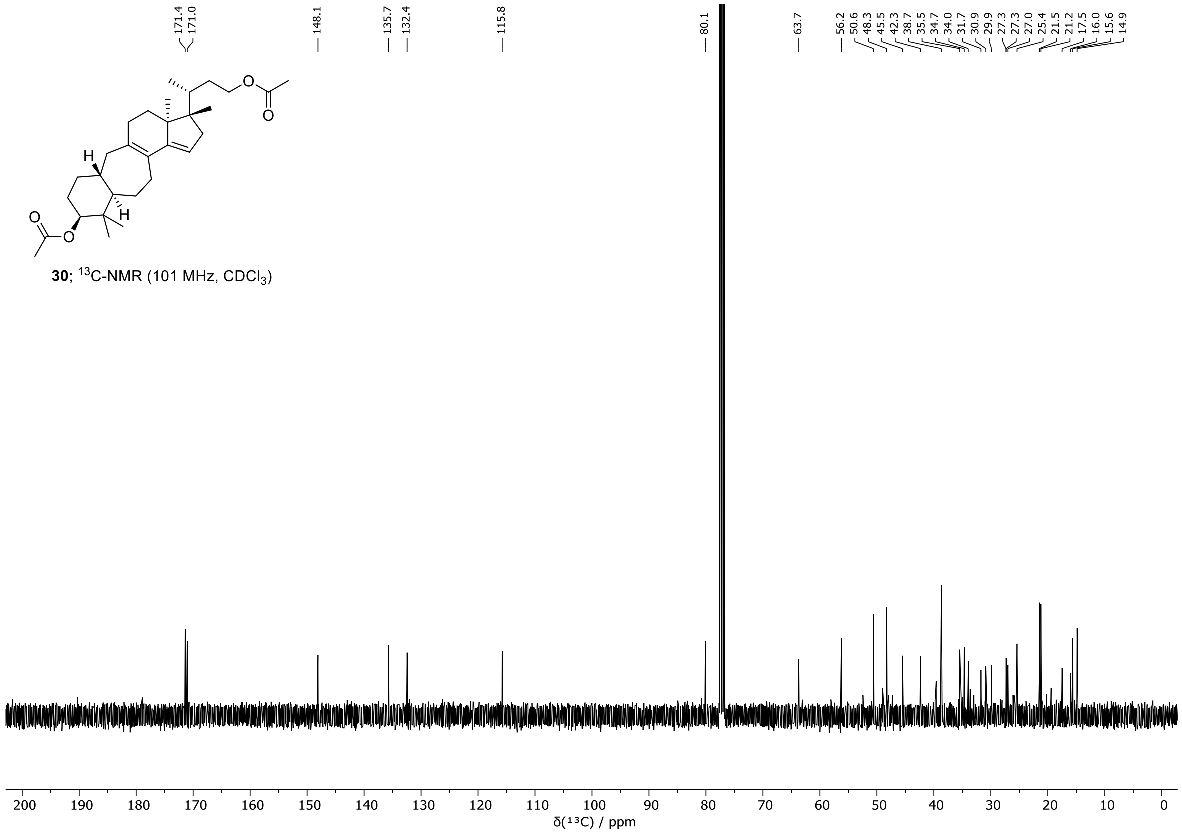
30; ^{13}C-NMR (101 MHz, $CDCl_3$)
δ(¹³C) / ppm

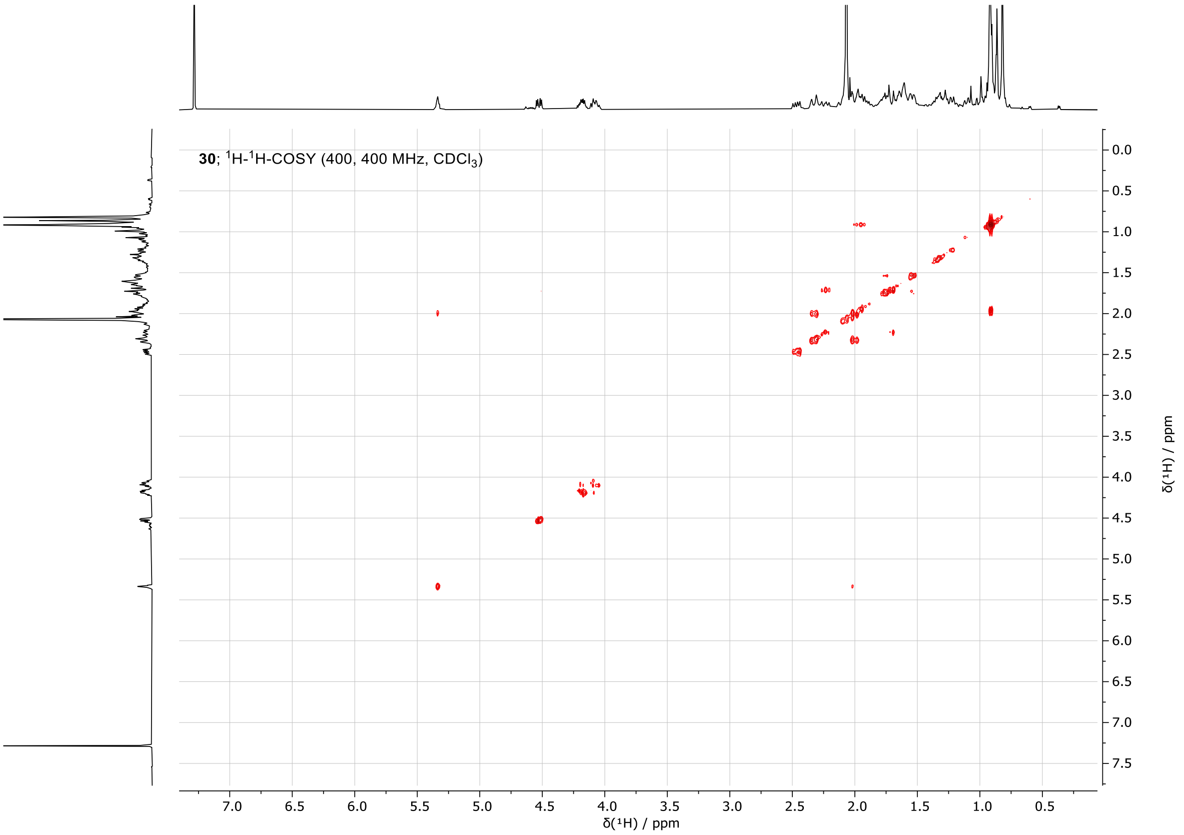
30; ¹H-¹H-COSY (400, 400 MHz, CDCl₃)
δ(¹H) / ppm
δ(¹H) / ppm

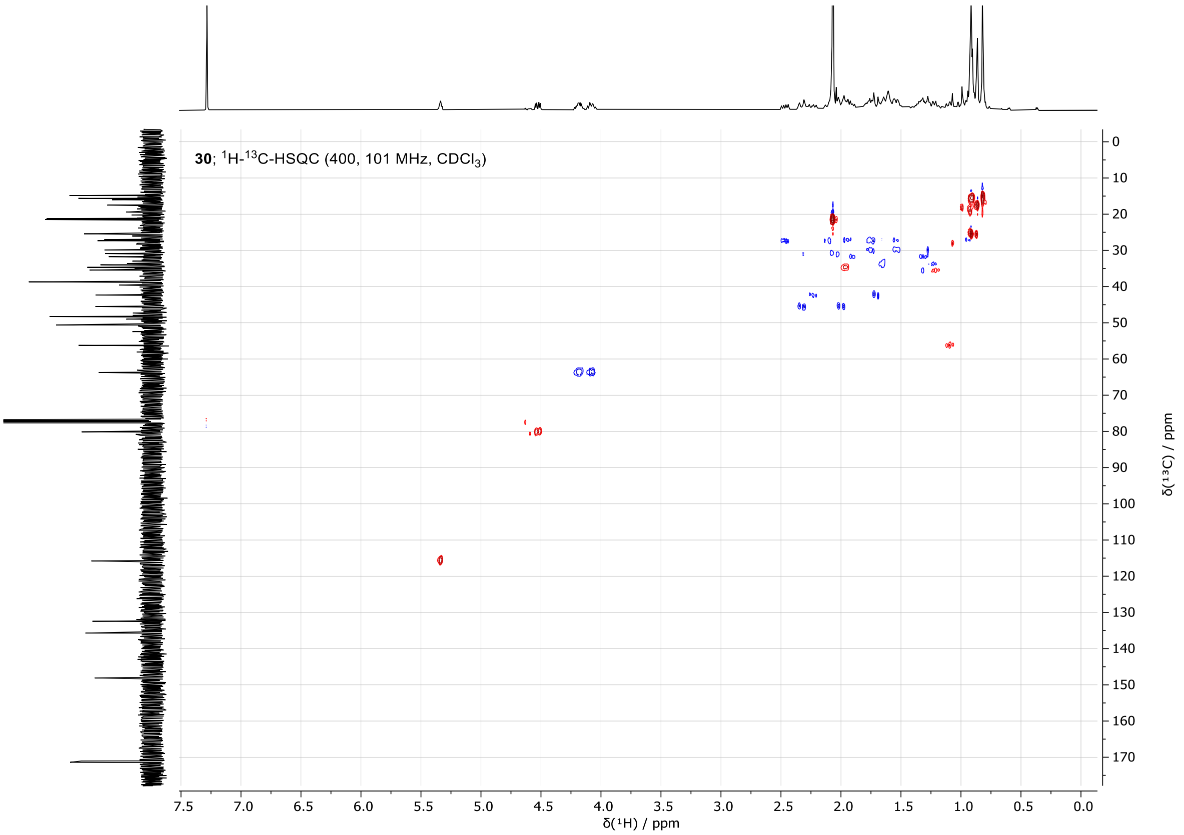
30; ¹H-¹³C-HSQC (400, 101 MHz, CDCl₃)
δ(¹H) / ppm
δ(¹³C) / ppm

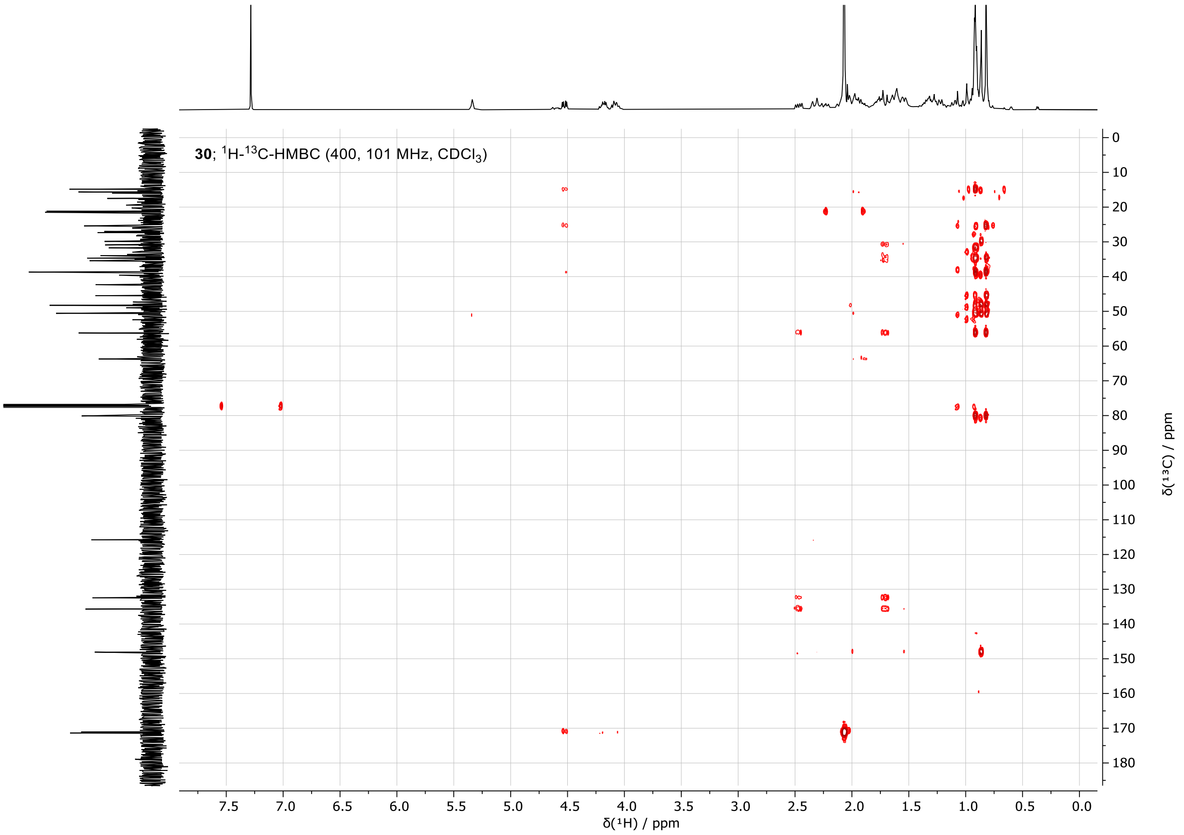
30; ¹H-¹³C-HMBC (400, 101 MHz, CDCl3)
δ(¹H) / ppm
δ(¹³C) / ppm

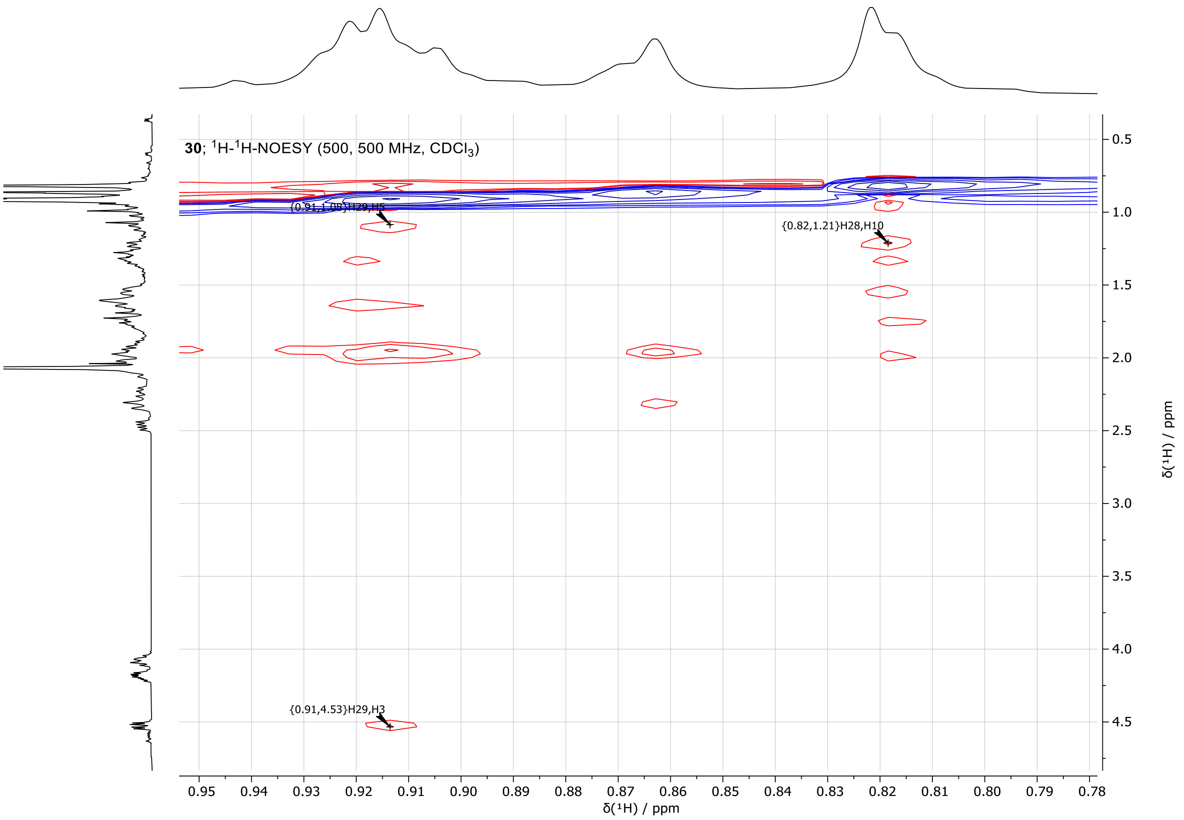
30; ¹H-¹H-NOESY (500, 500 MHz, CDCl3)
{0.91,1.09}H29,H5
{0.82,1.21}H28,H10
{0.91,4.53}H29,H3
δ(¹H) / ppm
δ(¹H) / ppm

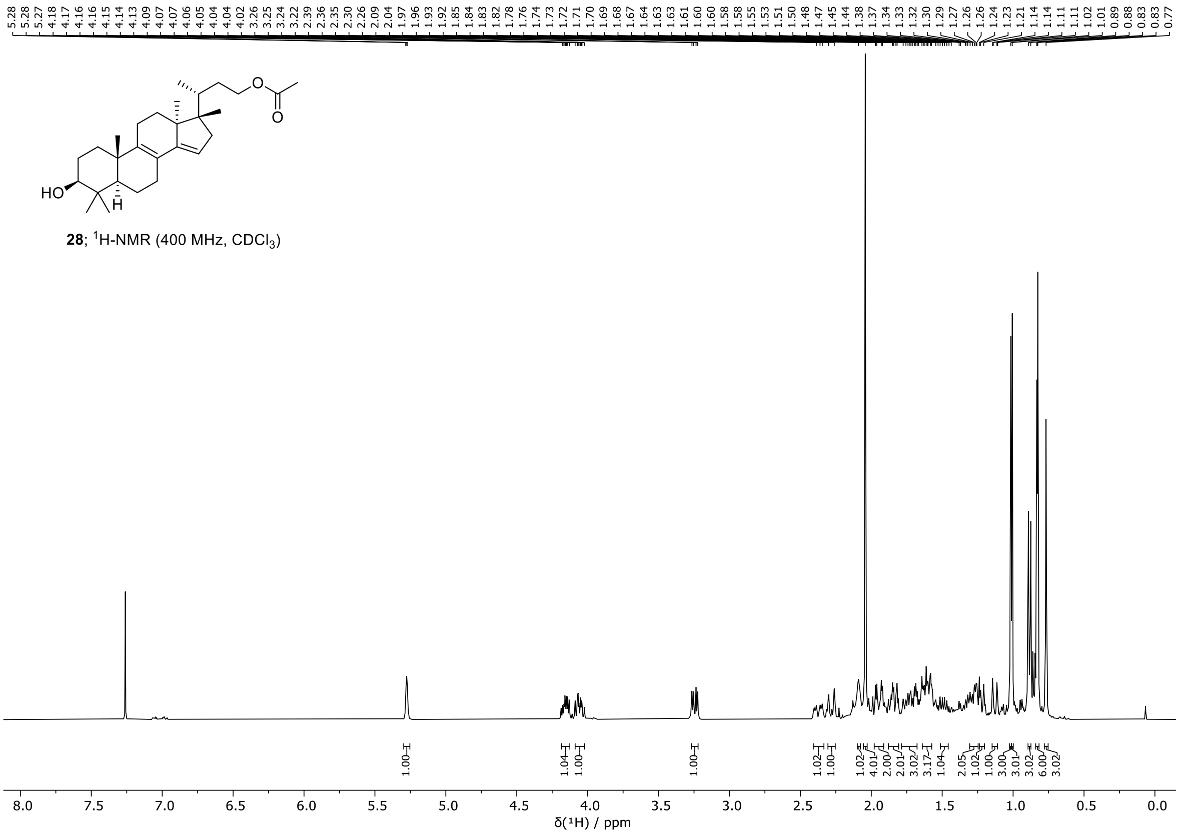

HO
H
O
O
28; ¹H-NMR (400 MHz, CDCl3)
8.0 7.5 7.0 6.5 6.0 5.5 5.0 4.5 4.0 3.5 3.0 2.5 2.0 1.5 1.0 0.5 0.0
δ(¹H) / ppm

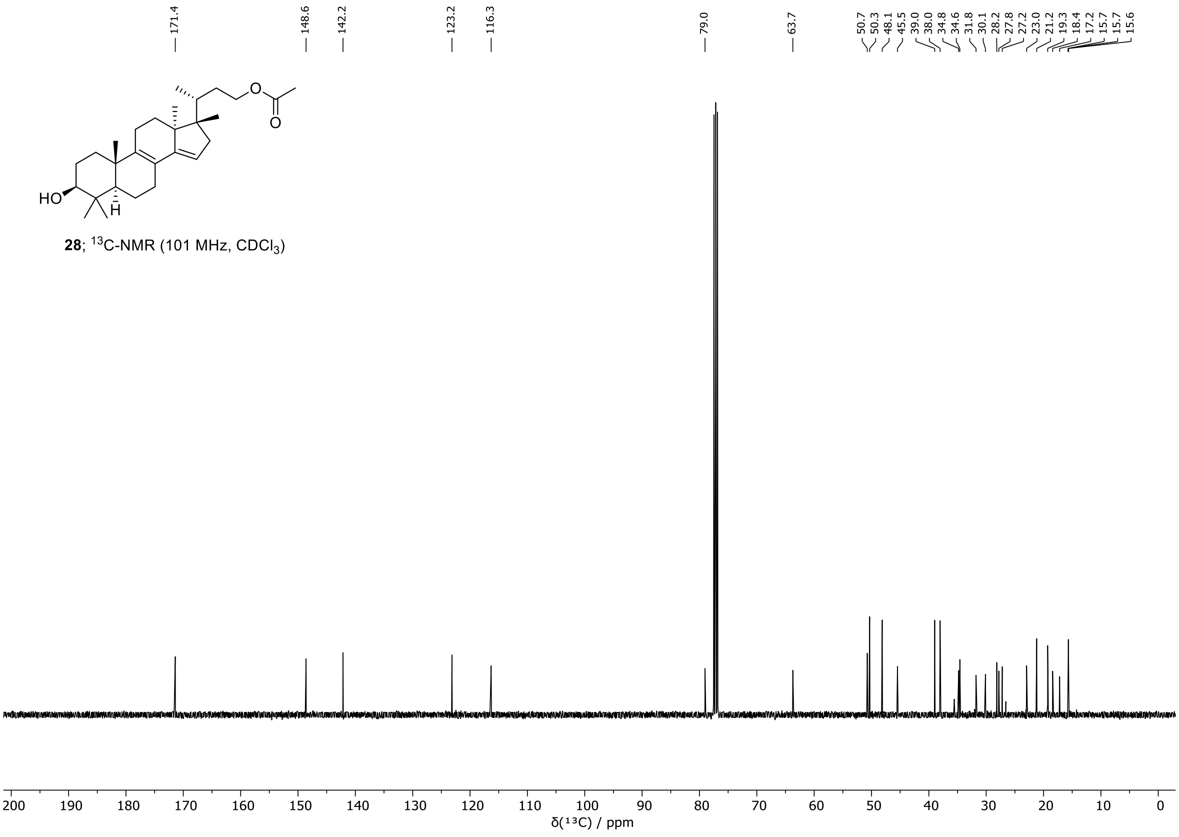

171.4
148.6
142.2
123.2
116.3
79.0
63.7
50.7 50.3 48.1 45.5 39.0 38.0 34.8 34.6 31.8 30.1 28.2 27.8 27.2 23.0 21.2 19.3 18.4 17.2 15.7 15.6
HO
H
O
O
28; ¹³C-NMR (101 MHz, CDCl3)
200 190 180 170 160 150 140 130 120 110 100 90 80 70 60 50 40 30 20 10 0
δ(¹³C) / ppm

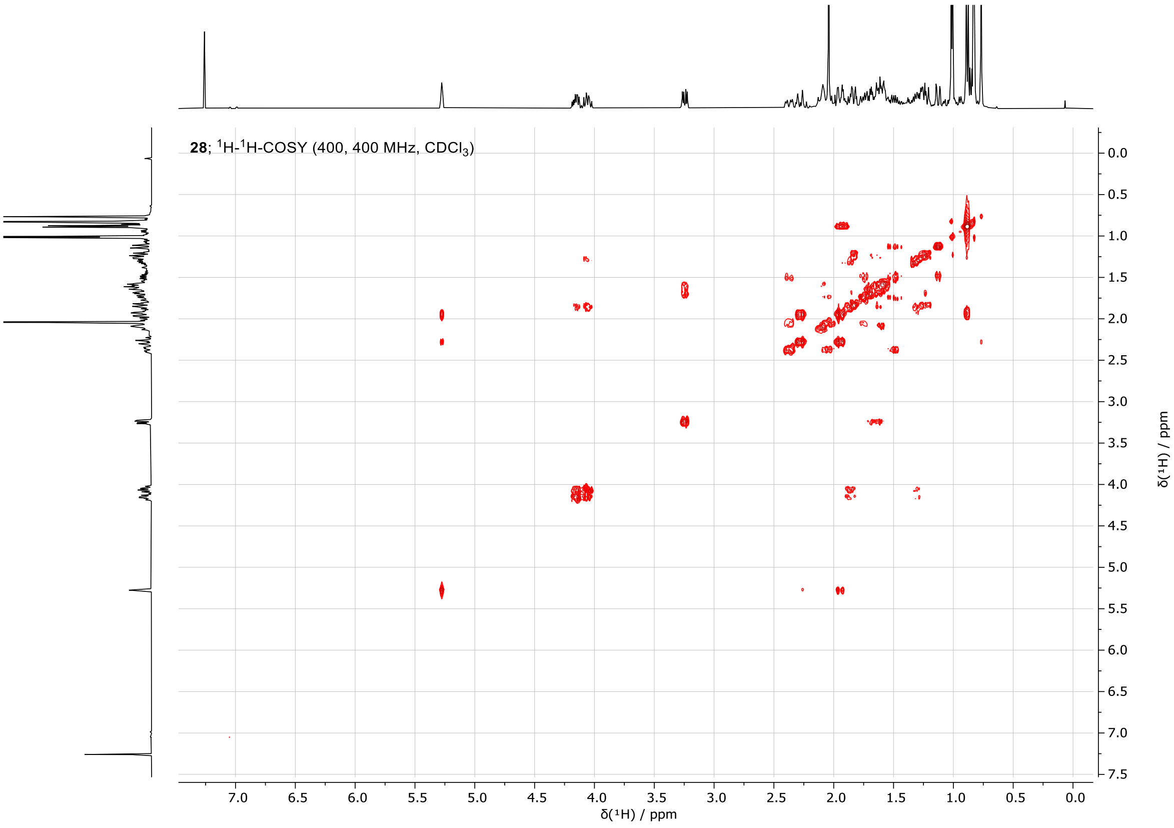
28; ¹H-¹H-COSY (400, 400 MHz, CDCl3)
δ(¹H) / ppm
δ(¹H) / ppm

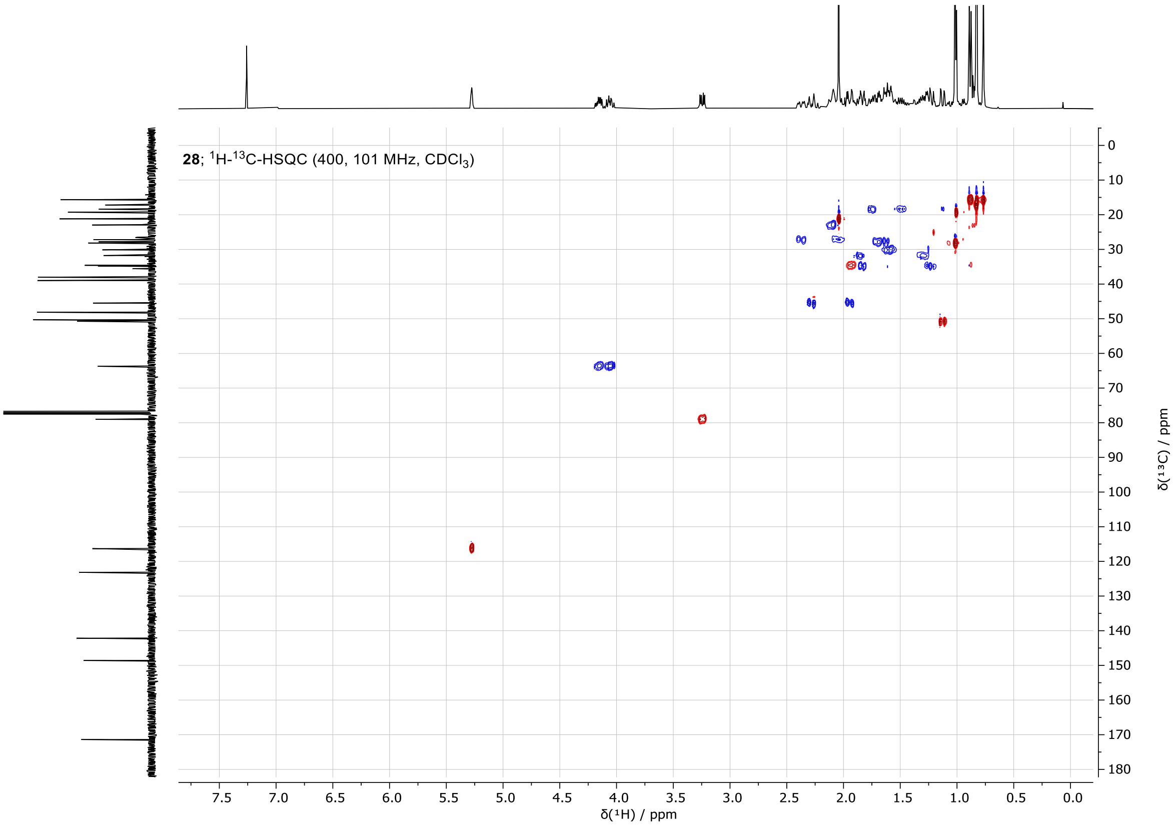
28; ¹H-¹³C-HSQC (400, 101 MHz, CDCl3)
δ(¹H) / ppm
δ(¹³C) / ppm

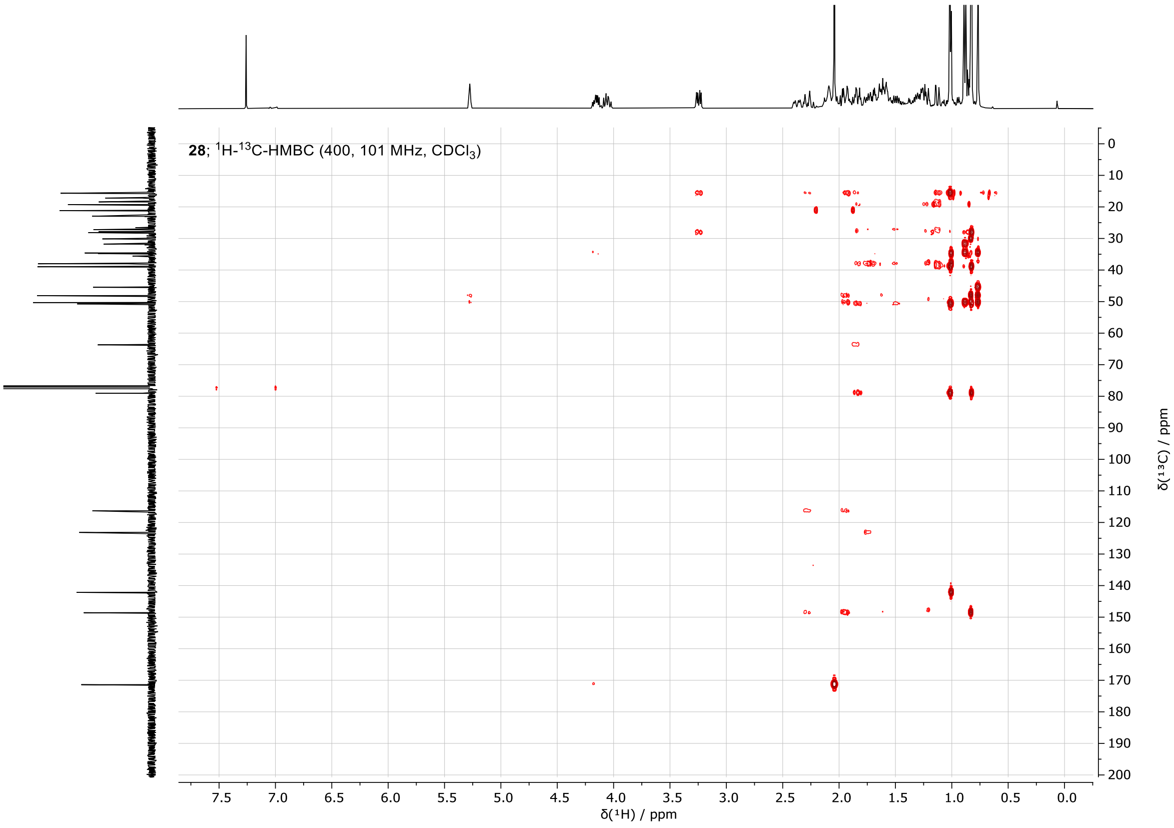
28; ¹H-¹³C-HMBC (400, 101 MHz, CDCl₃)
δ(¹³C) / ppm
δ(¹H) / ppm

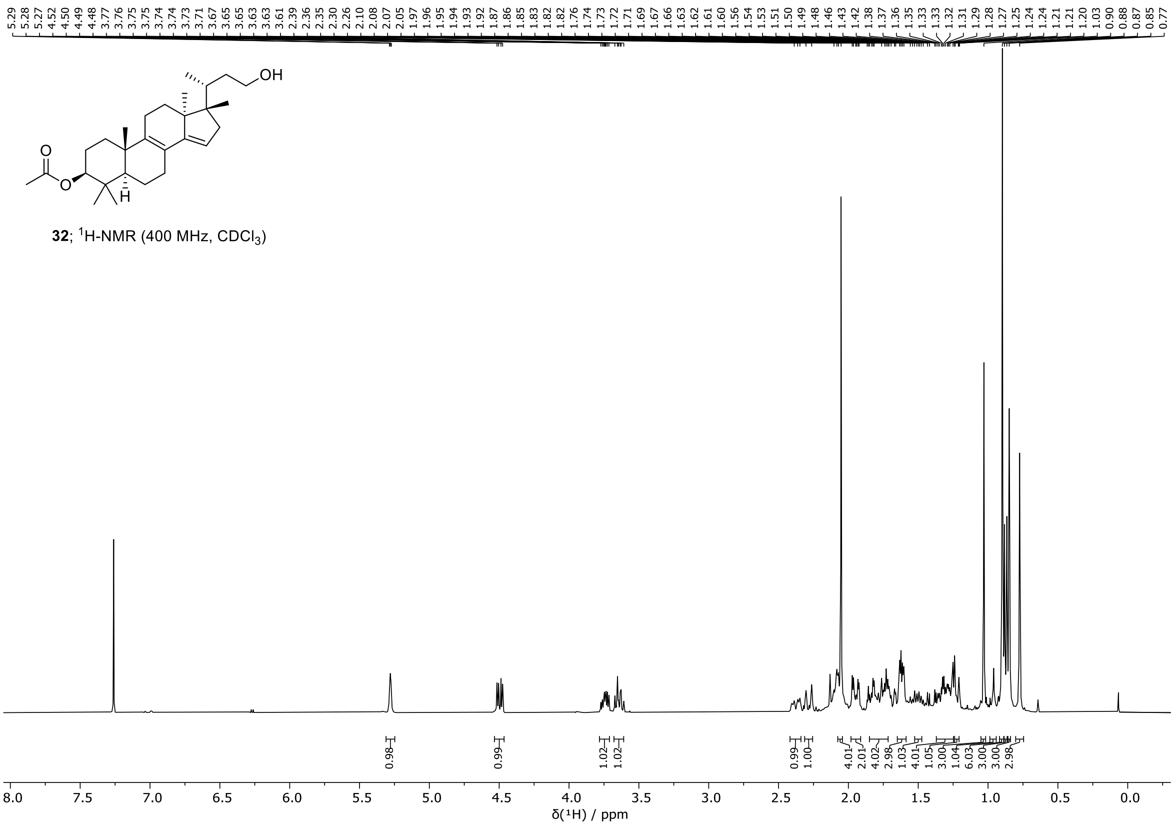
OH
O
O
H
32; ¹H-NMR (400 MHz, CDCl₃)
δ(¹H) / ppm

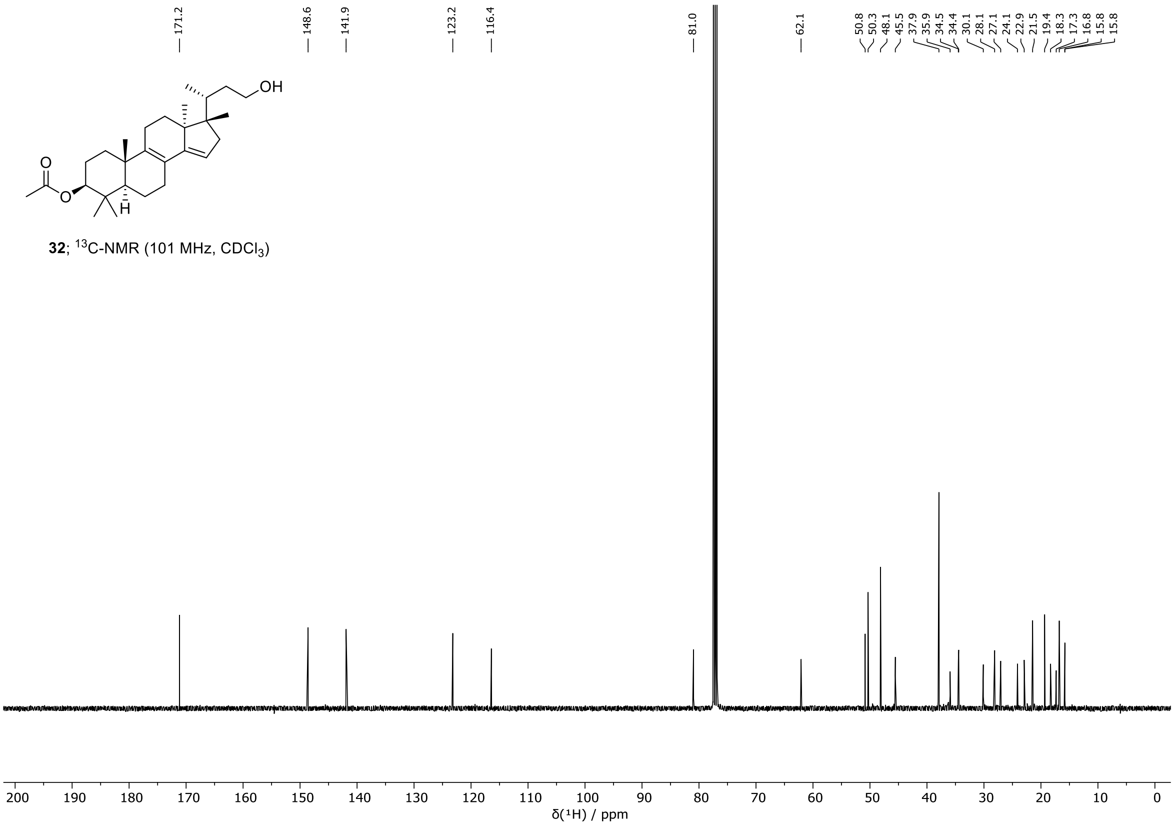

32; ^{13}C-NMR (101 MHz, $CDCl_3$)

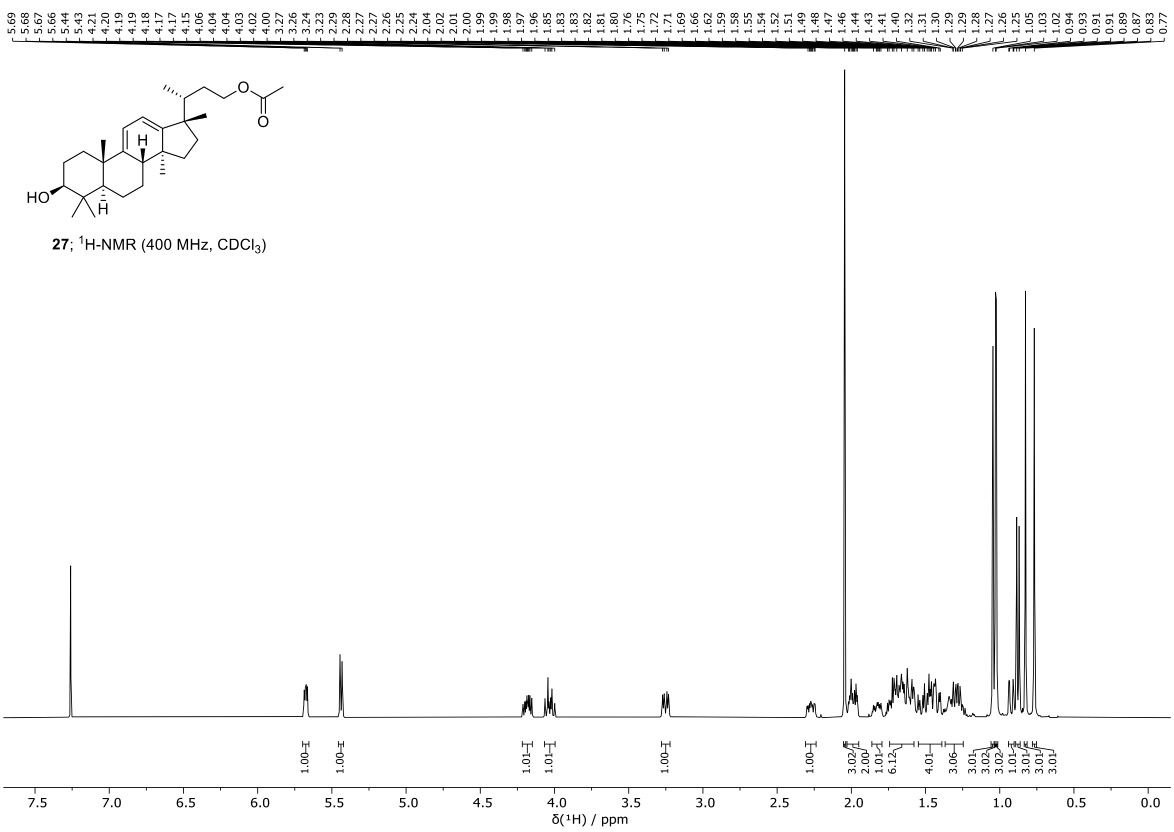

27; ^{1}H-NMR (400 MHz, $CDCl_3$)

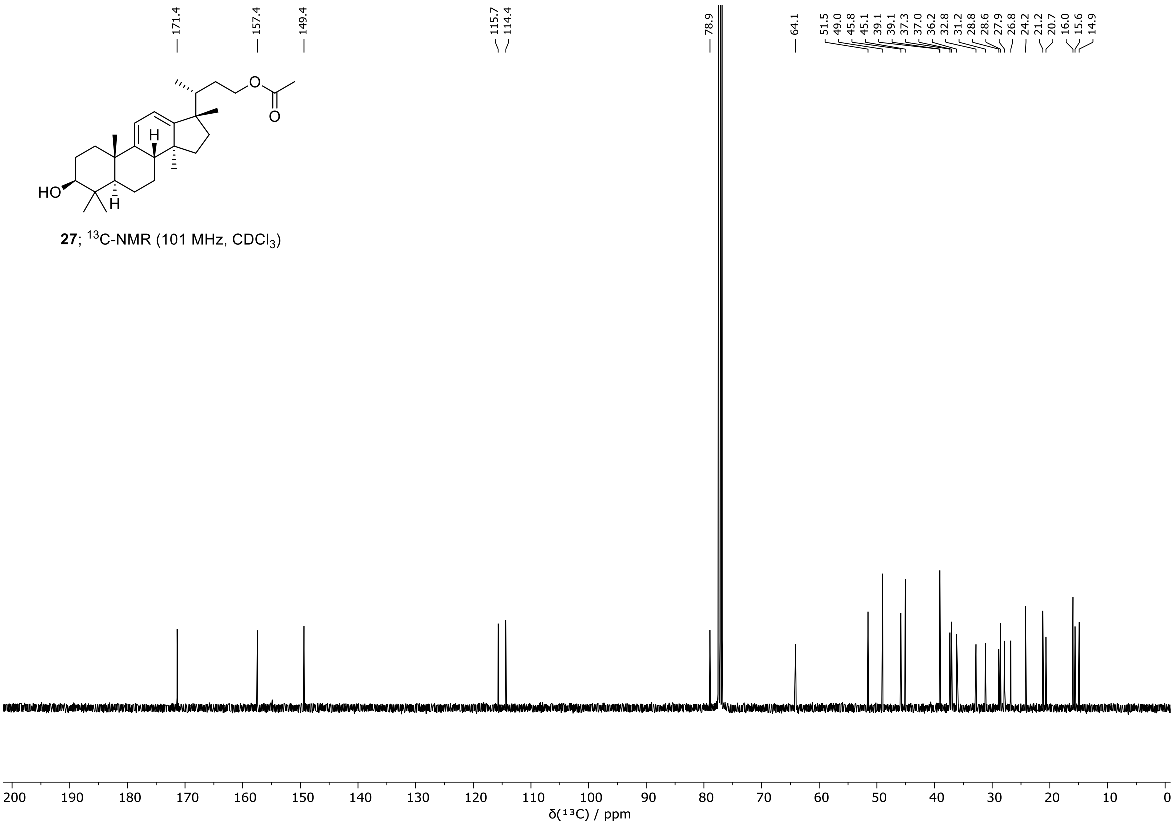

171.4
157.4
149.4
115.7
114.4
78.9
64.1
51.5
49.0
45.8
45.1
39.1
39.1
37.3
37.0
36.2
32.8
31.2
28.8
28.6
27.9
26.8
24.2
21.2
20.7
16.0
15.6
14.9
HO
H
H
O
O
27; ^{13}C-NMR (101 MHz, CDCl$_3$)
δ(¹³C) / ppm

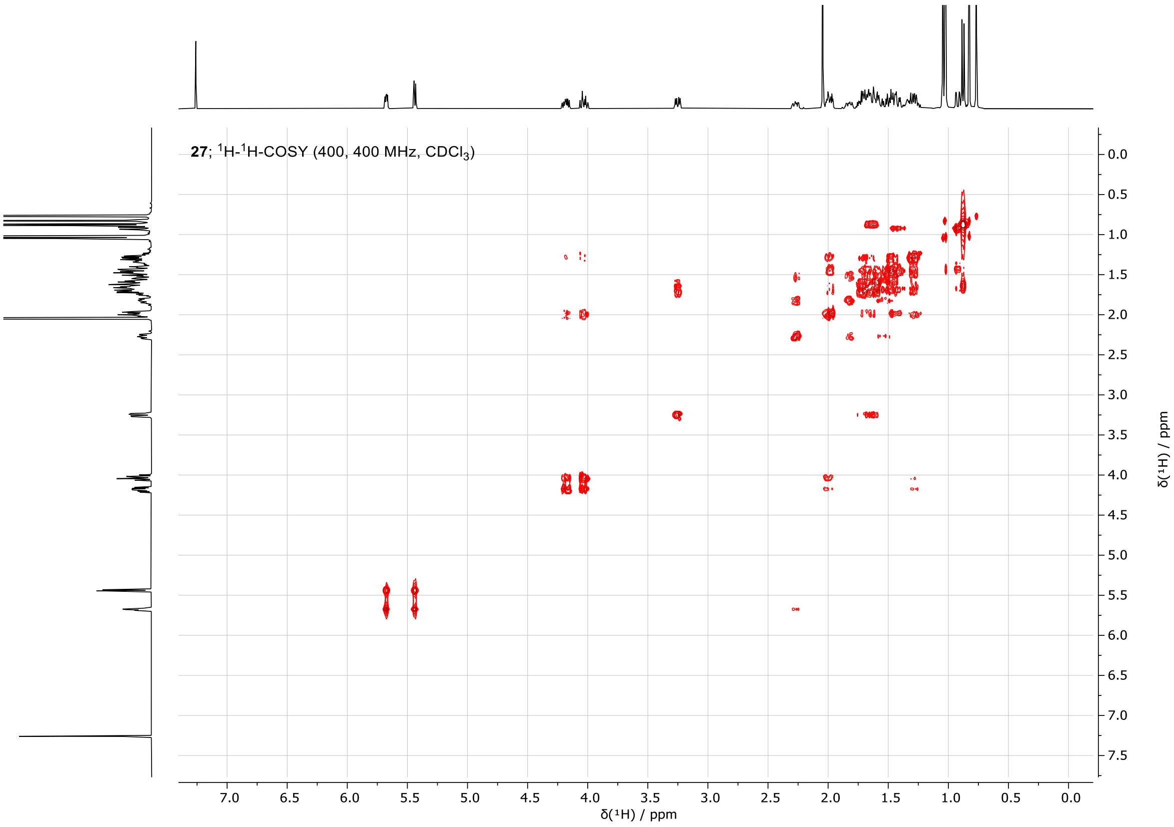

27; ^{1}H-^{1}H-COSY (400, 400 MHz, CDCl$_3$)
δ(¹H) / ppm
δ(¹H) / ppm

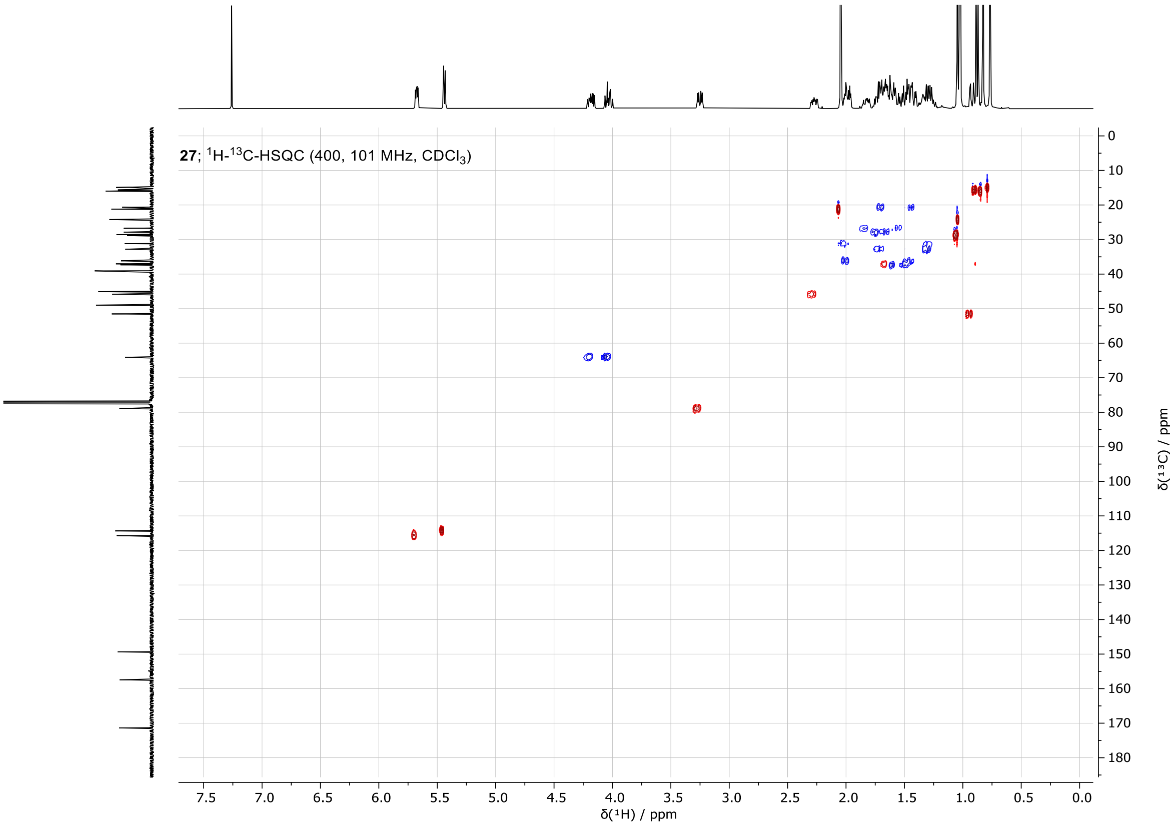

27; ¹H-¹³C-HSQC (400, 101 MHz, CDCl₃)
δ(¹H) / ppm
δ(¹³C) / ppm

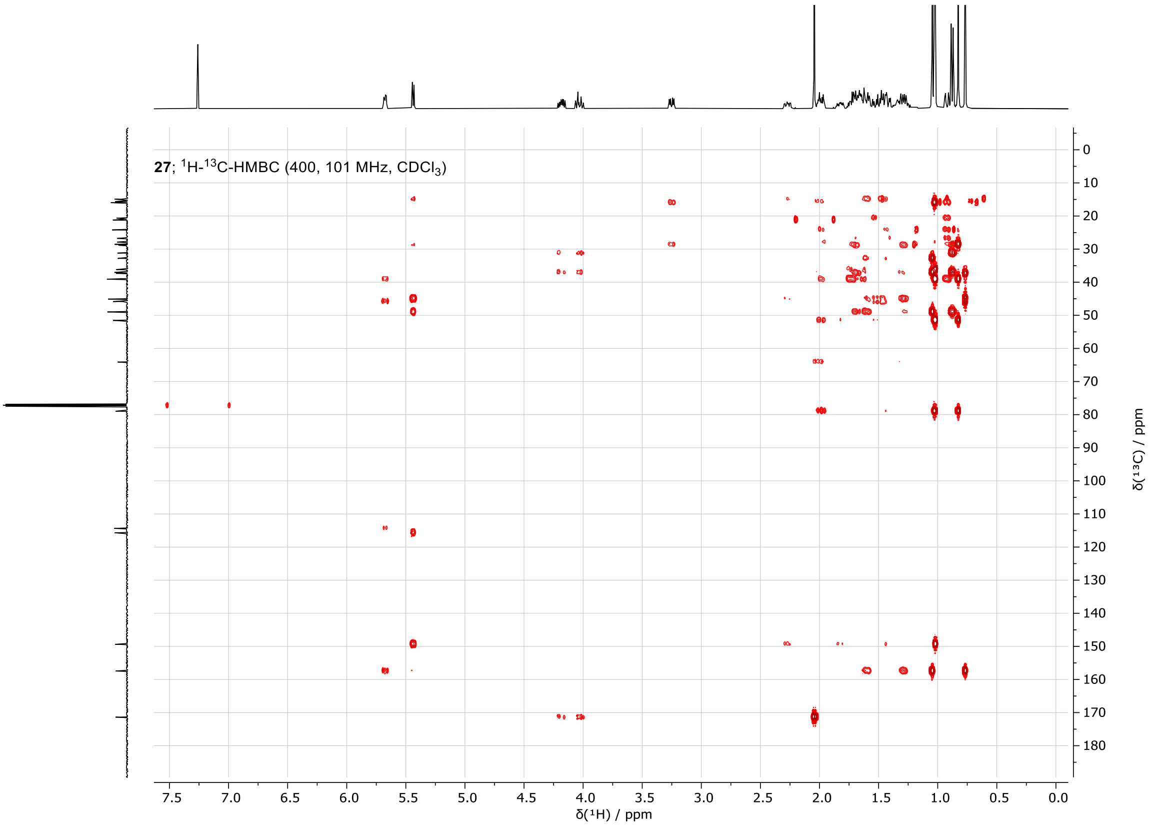

27; ¹H-¹³C-HMBC (400, 101 MHz, CDCl₃)
δ(¹H) / ppm
δ(¹³C) / ppm

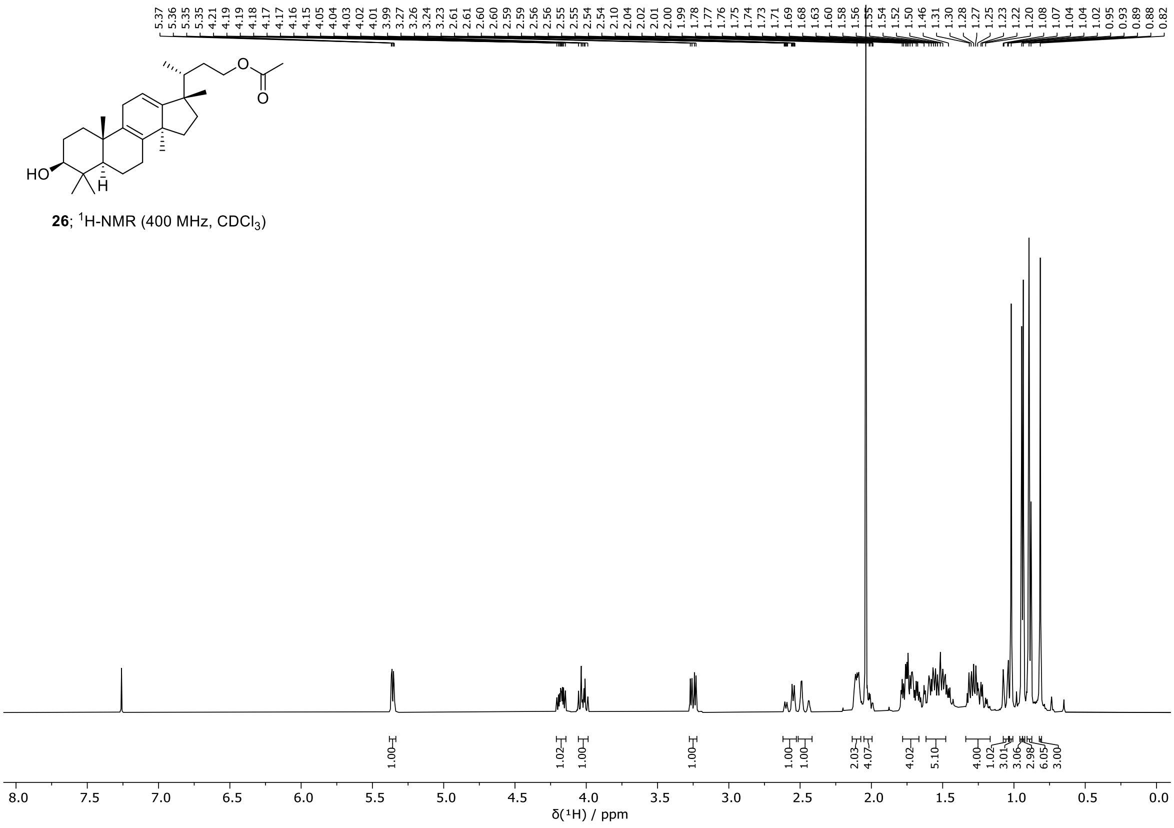

26; ^{1}H-NMR (400 MHz, $CDCl_3$)

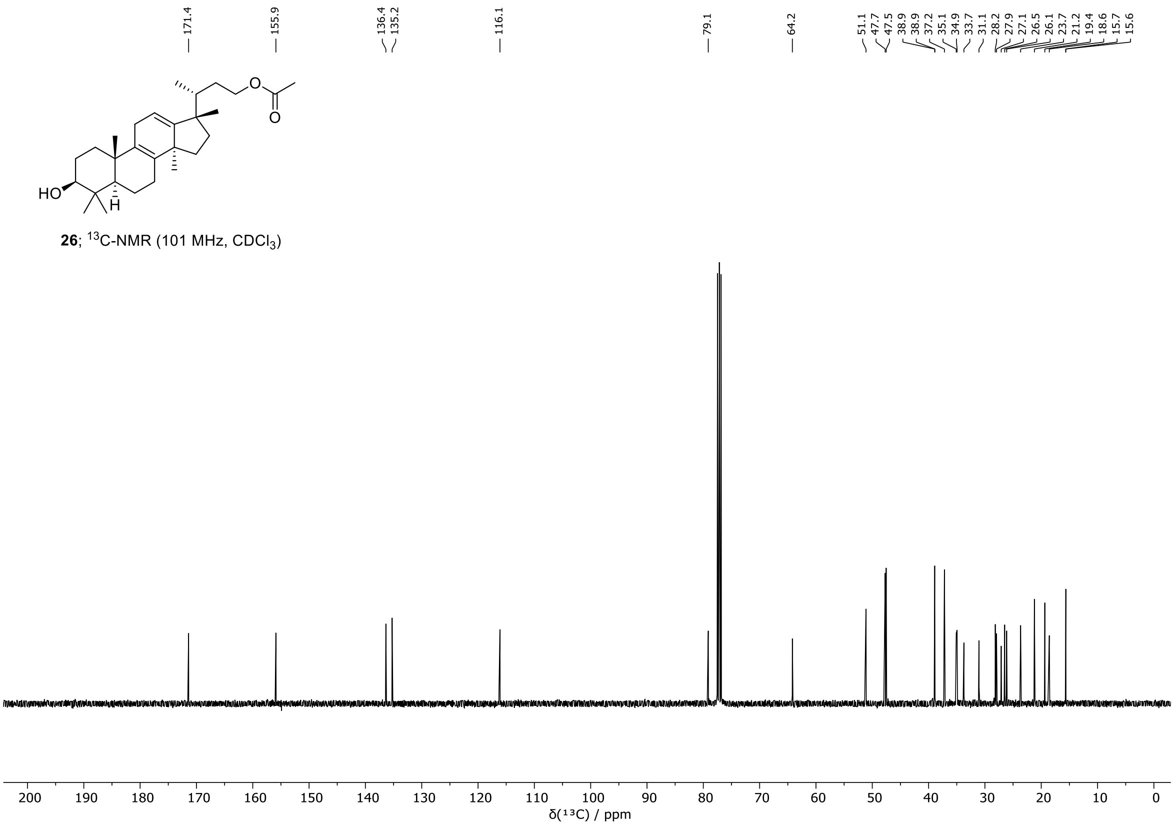

26; ^{13}C-NMR (101 MHz, $CDCl_3$)

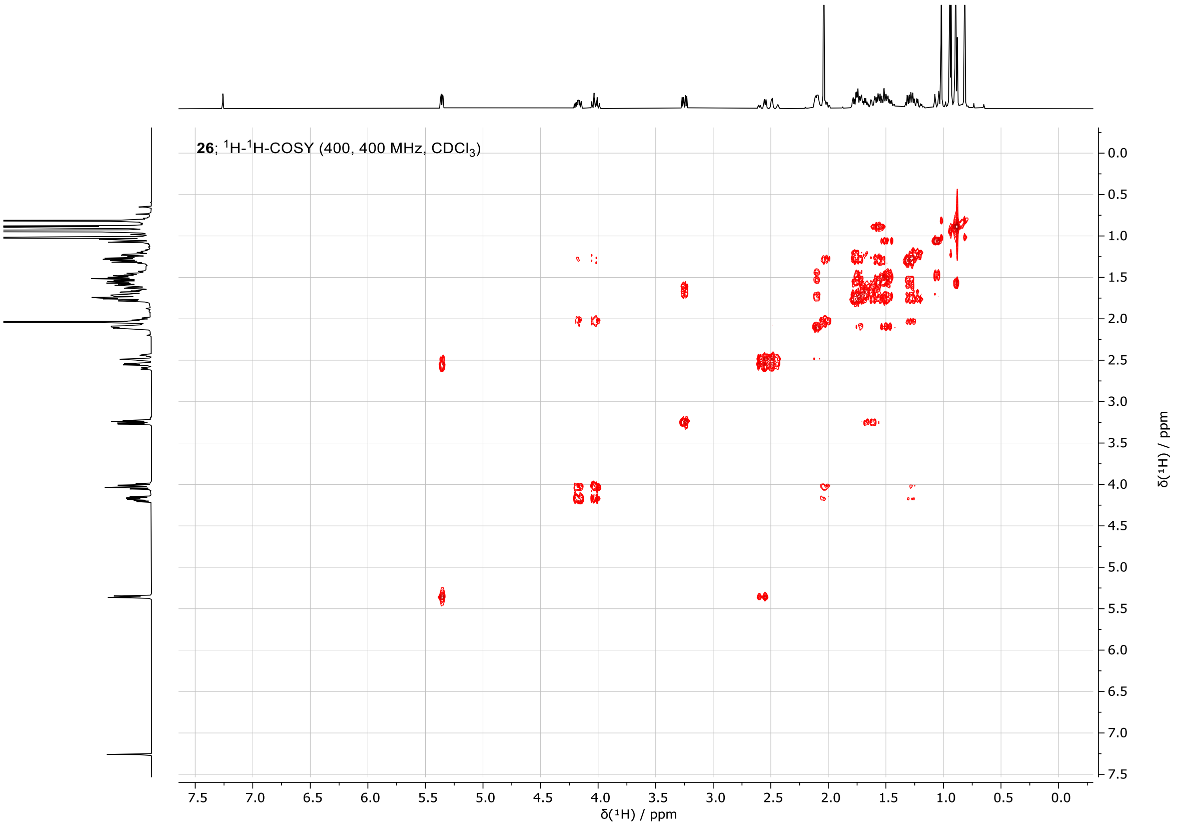

26; ¹H-¹H-COSY (400, 400 MHz, CDCl₃)
δ(¹H) / ppm
δ(¹H) / ppm

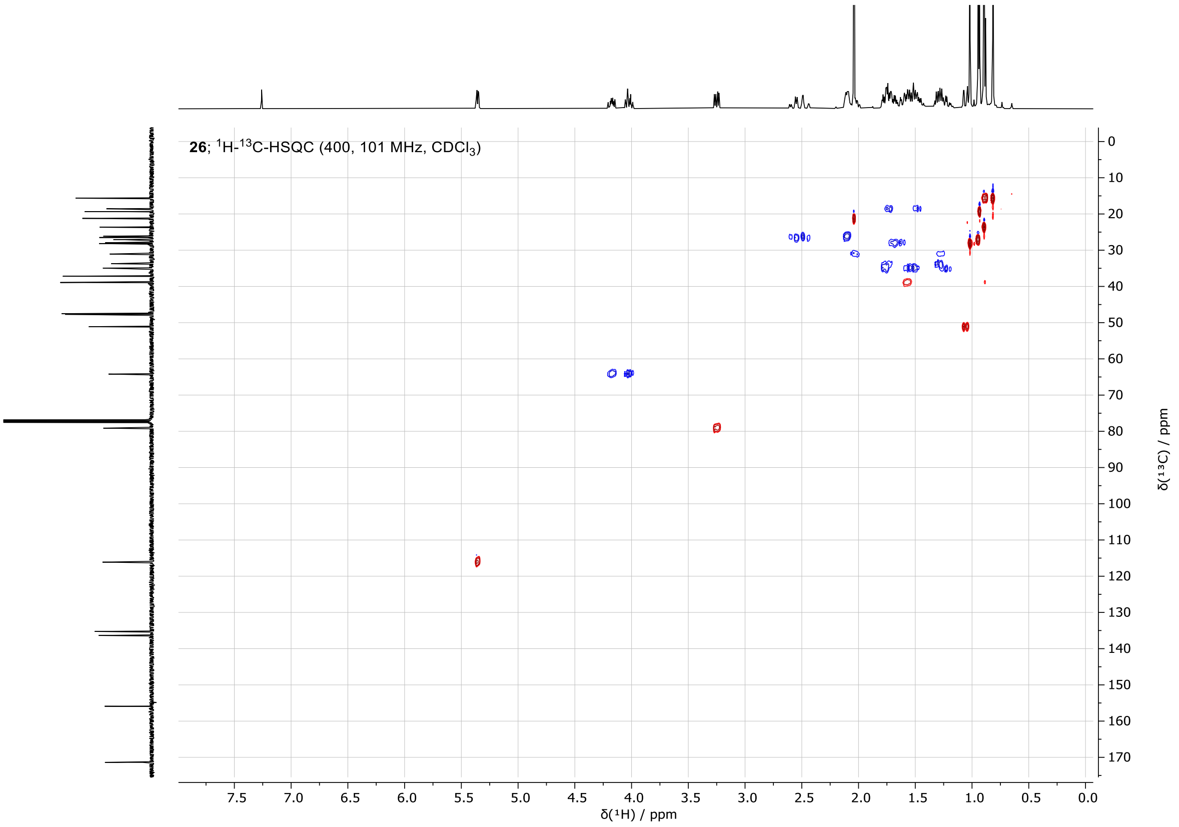

26; ¹H-¹³C-HSQC (400, 101 MHz, CDCl₃)
δ(¹H) / ppm
δ(¹³C) / ppm

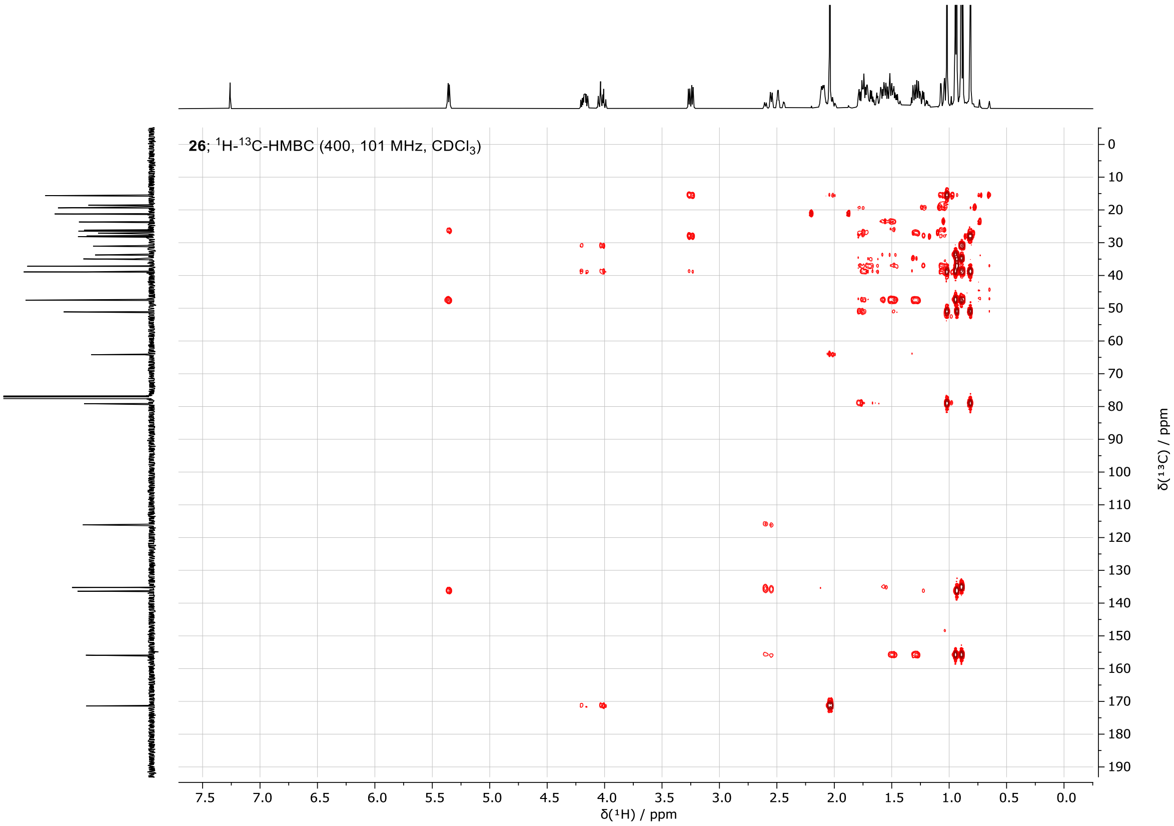
26; ¹H-¹³C-HMBC (400, 101 MHz, CDCl₃)
δ(¹H) / ppm
δ(¹³C) / ppm

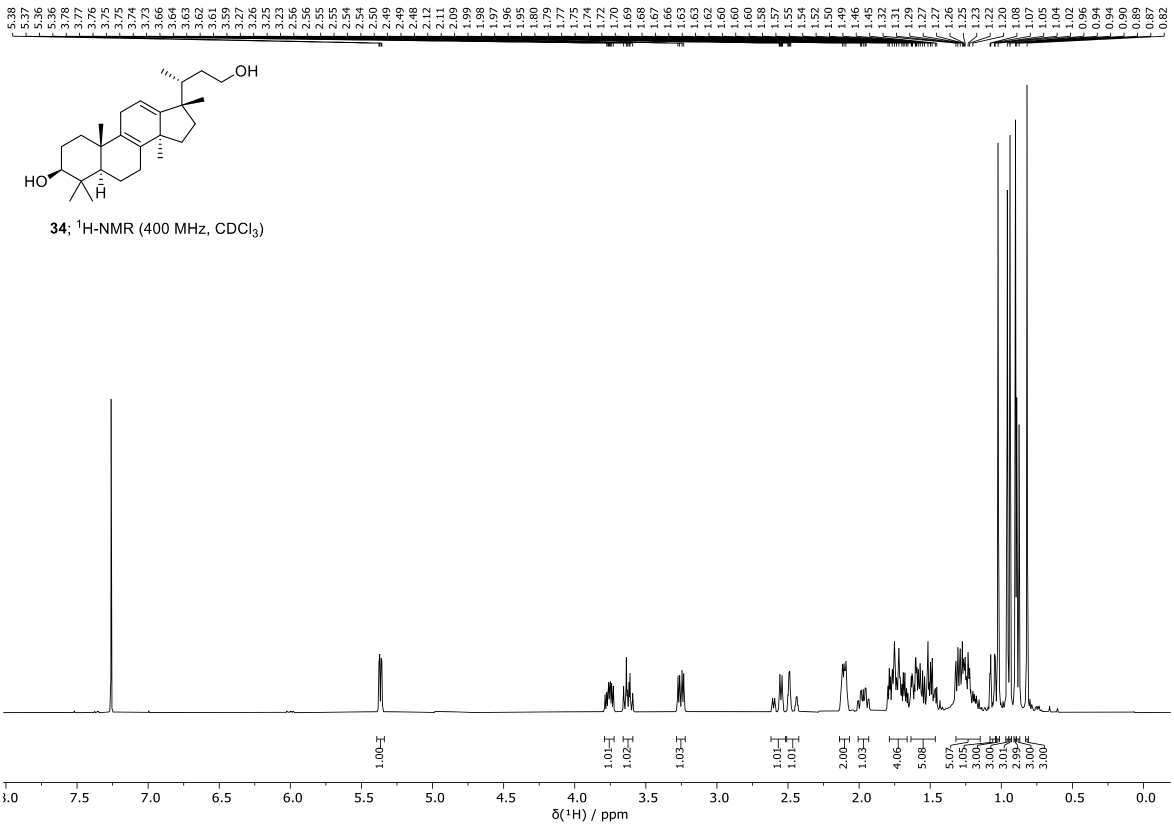
OH
HO
H
34; ¹H-NMR (400 MHz, CDCl₃)
δ(¹H) / ppm

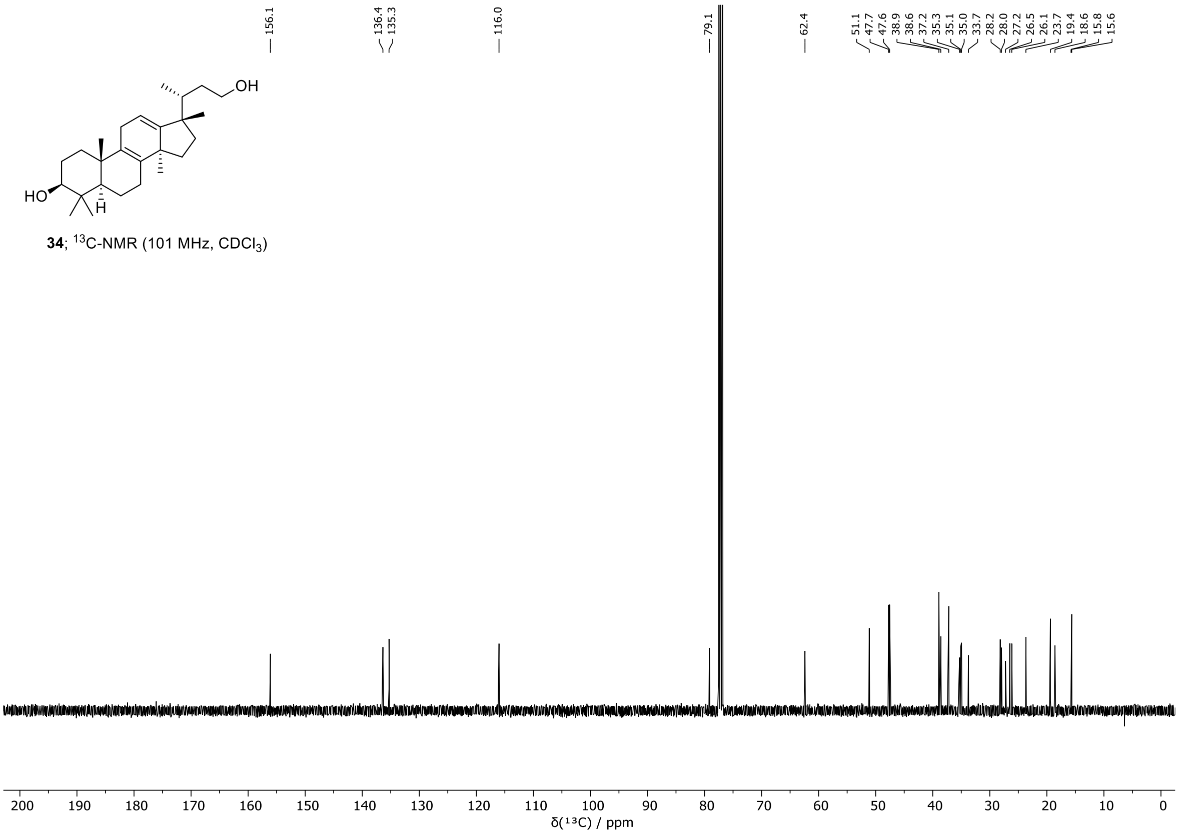

34; ^{13}C-NMR (101 MHz, $CDCl_3$)

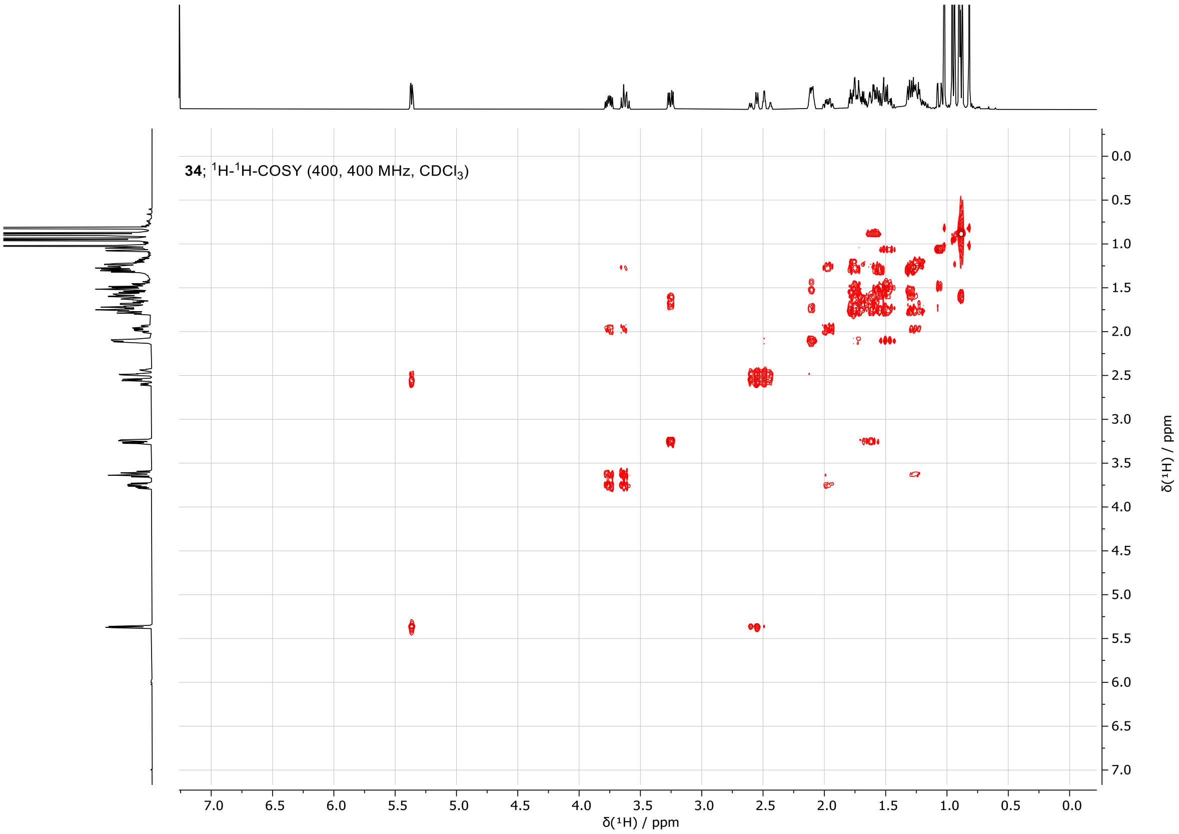

34; ^{1}H-^{1}H-COSY (400, 400 MHz, $CDCl_3$)

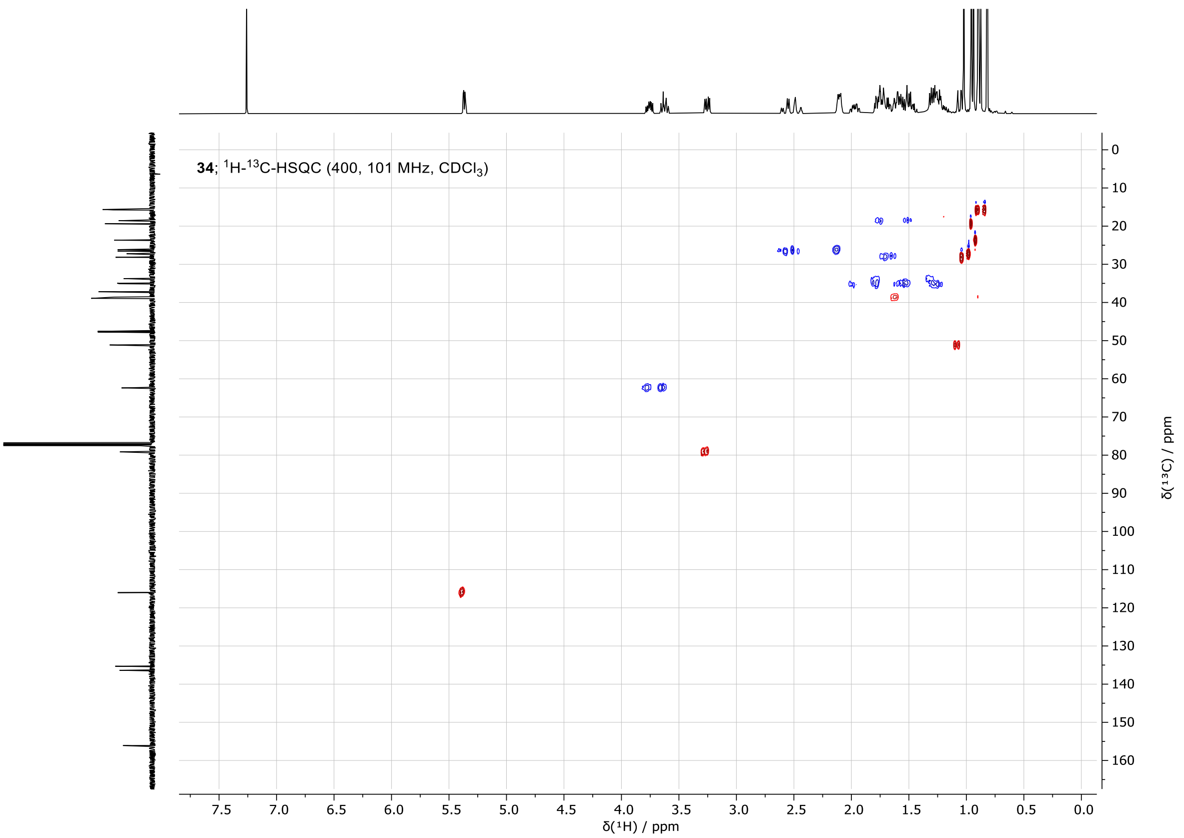
34; ¹H-¹³C-HSQC (400, 101 MHz, CDCl₃)
δ(¹H) / ppm
δ(¹³C) / ppm

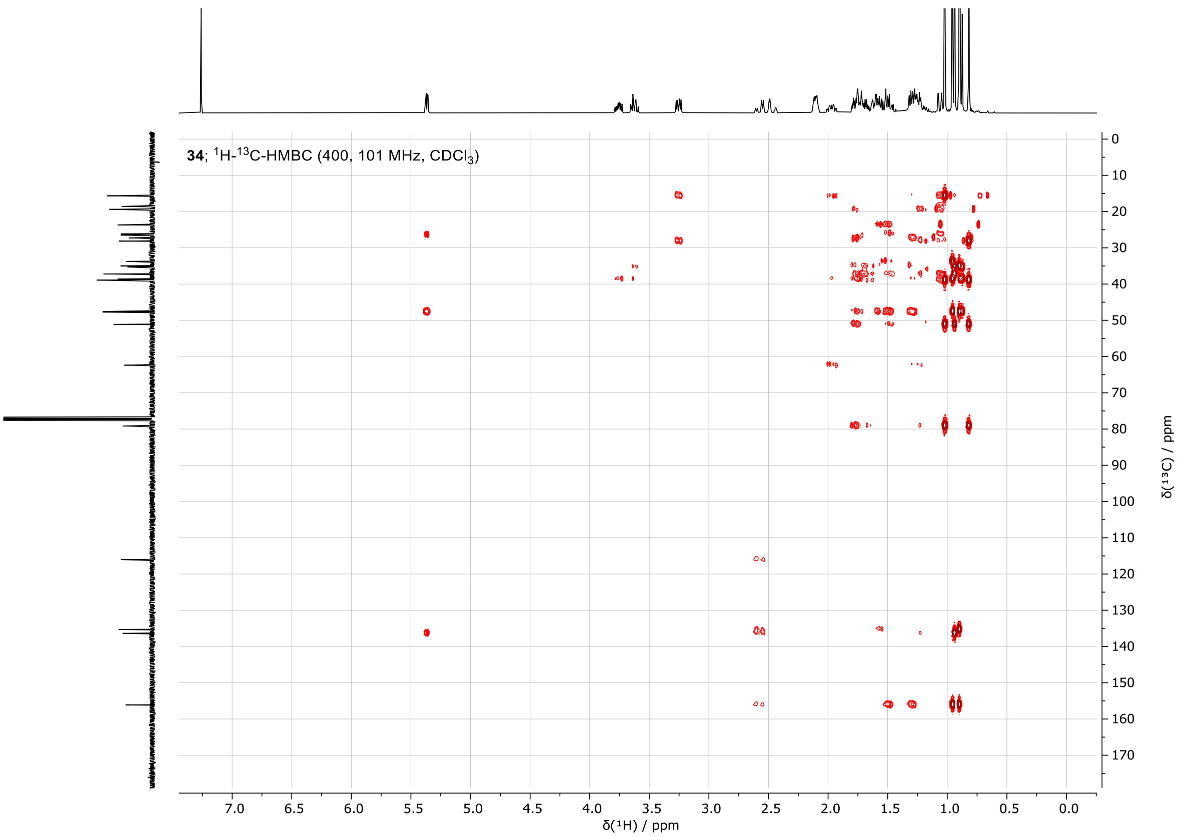
34; ¹H-¹³C-HMBC (400, 101 MHz, CDCl₃)
δ(¹H) / ppm
δ(¹³C) / ppm

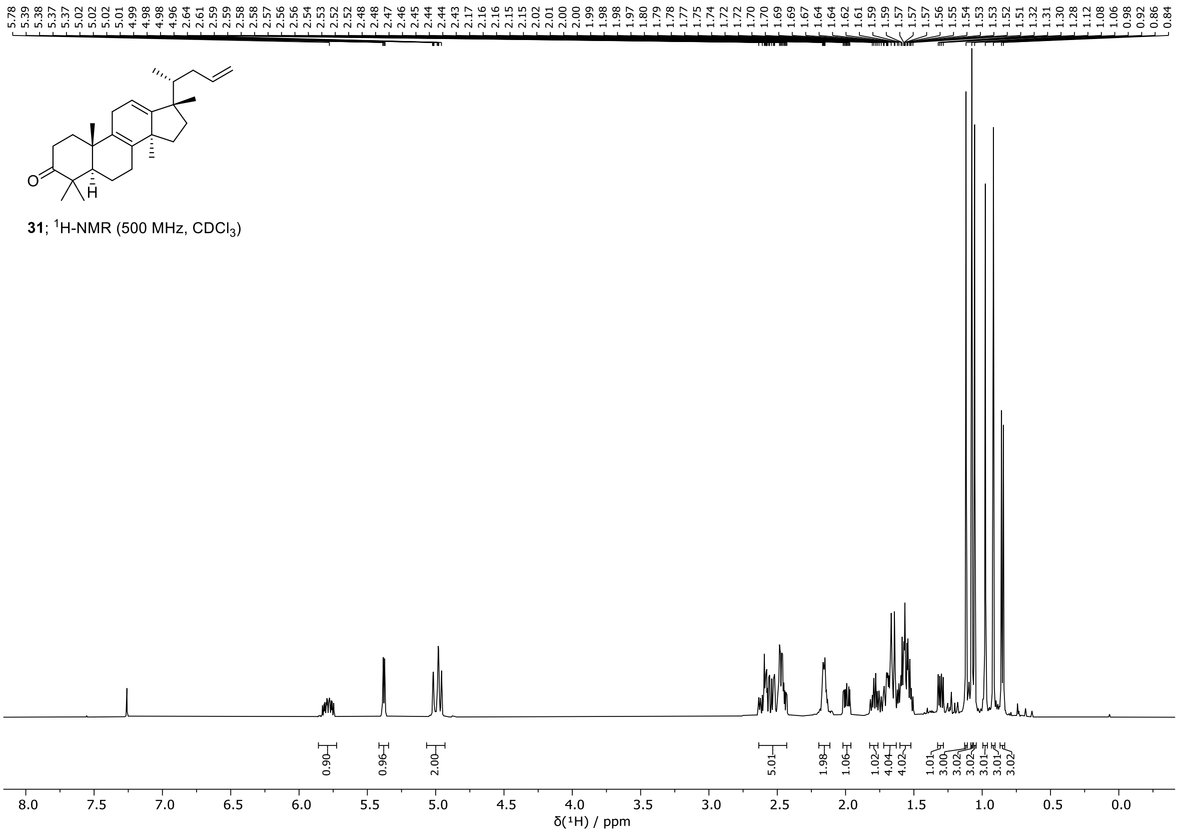

31; ^{1}H-NMR (500 MHz, $CDCl_3$)

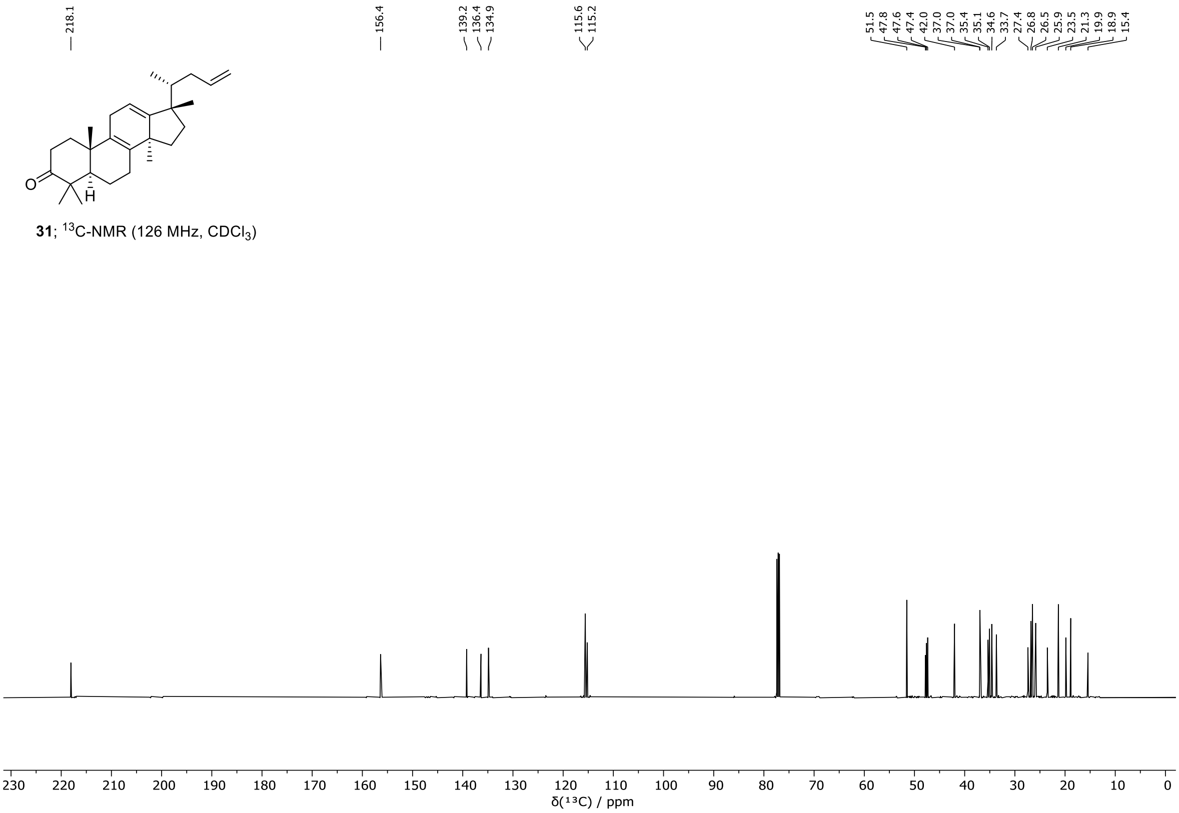

31; ^{13}C-NMR (126 MHz, $CDCl_3$)

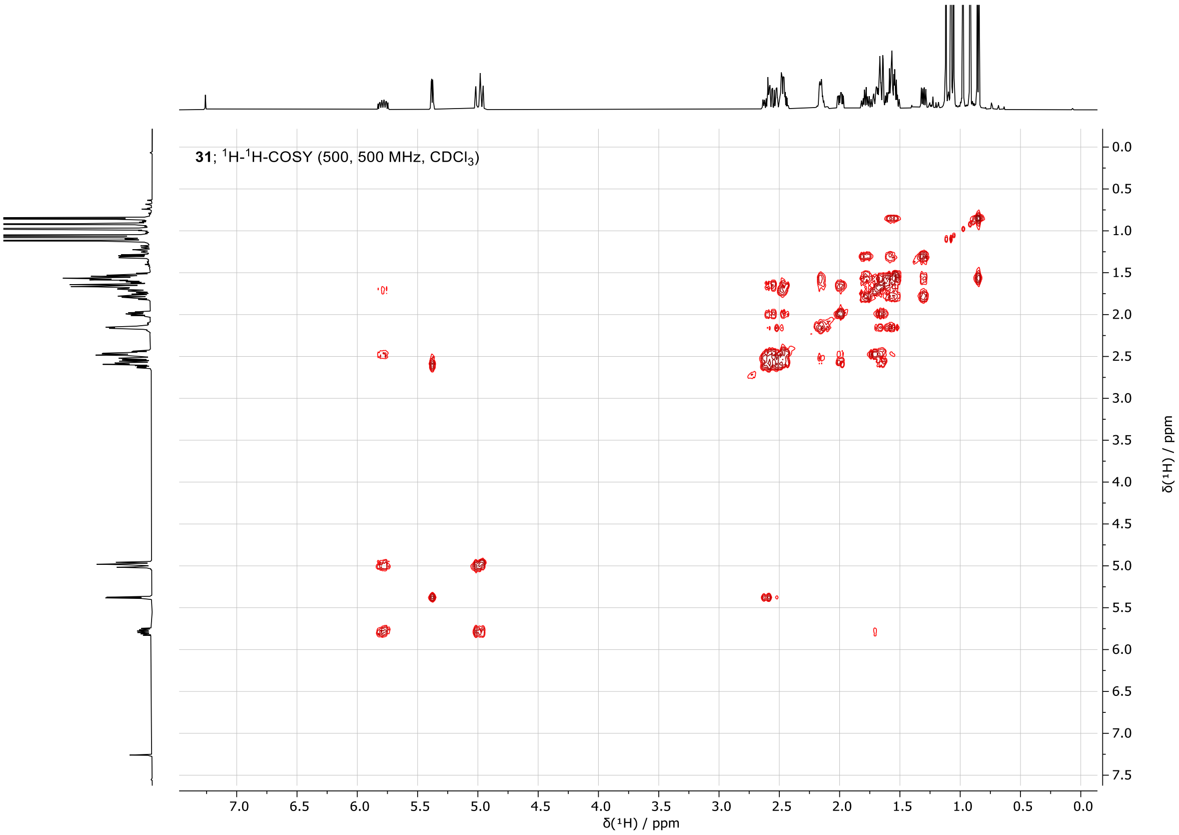
31; 1H-1H-COSY (500, 500 MHz, CDCl3)
δ(¹H) / ppm
δ(¹H) / ppm

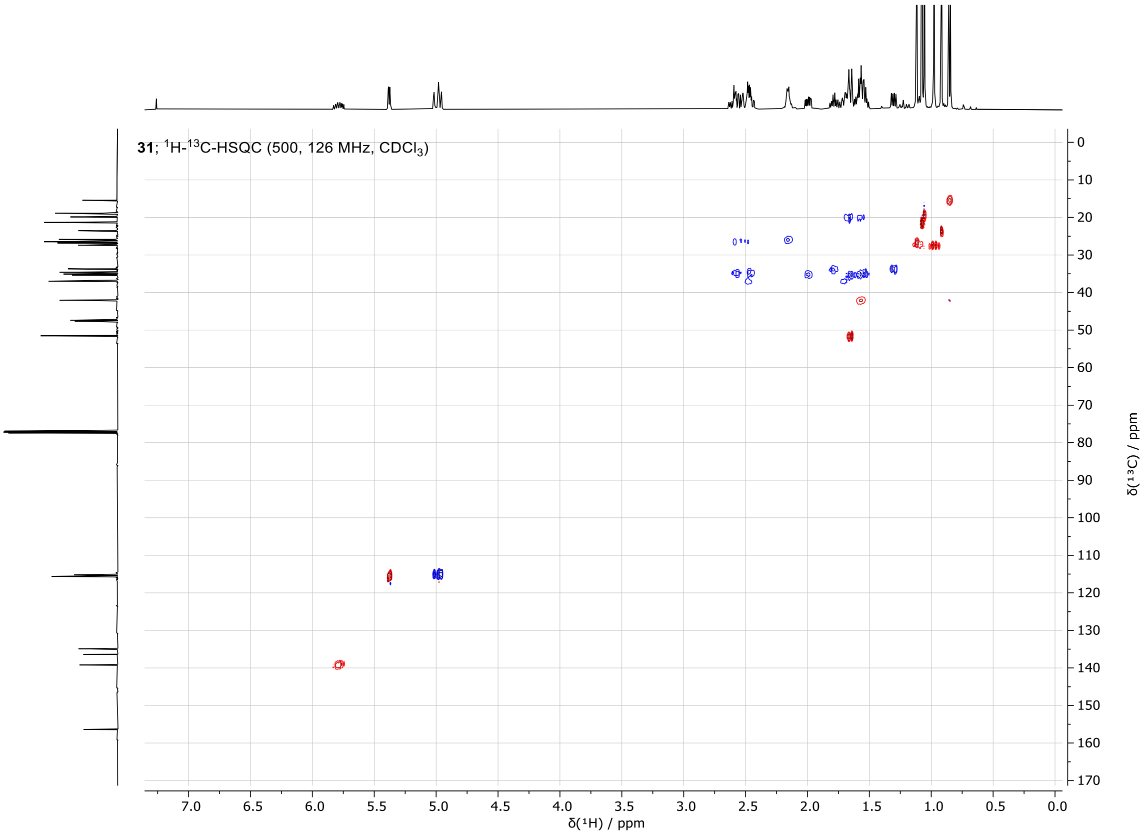
31; 1H-13C-HSQC (500, 126 MHz, CDCl3)
δ(¹H) / ppm
δ(¹³C) / ppm

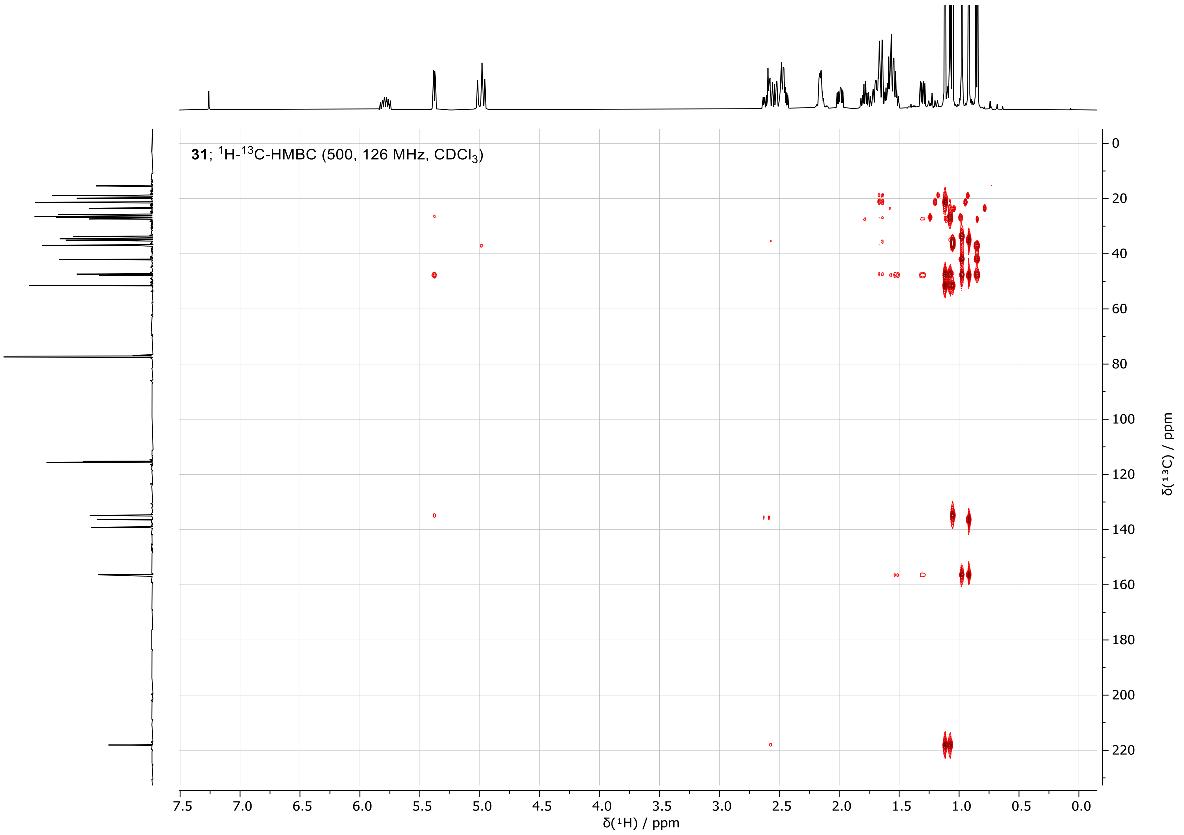
31; ^{1}H-^{13}C-HMBC (500, 126 MHz, CDCl$_3$)
δ(¹³C) / ppm
δ(¹H) / ppm

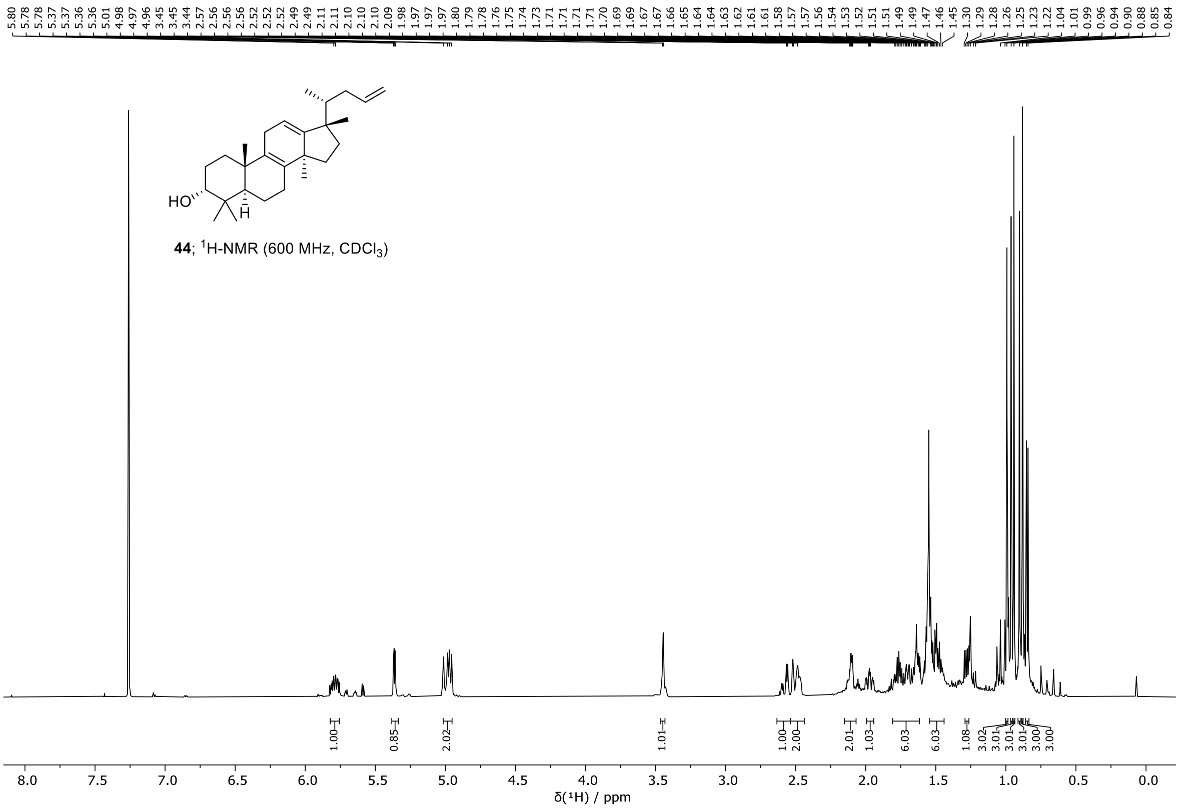
5.80 5.78 5.78 5.37 5.37 5.36 5.36 5.01 4.98 4.97 4.96 3.45 3.45 3.44 2.57 2.56 2.56 2.56 2.52 2.52 2.52 2.49 2.49 2.11 2.10 2.10 2.10 2.09 1.98 1.97 1.97 1.97 1.80 1.79 1.78 1.76 1.75 1.74 1.73 1.71 1.71 1.71 1.71 1.70 1.69 1.69 1.67 1.67 1.66 1.65 1.64 1.64 1.63 1.62 1.61 1.61 1.58 1.57 1.57 1.56 1.54 1.53 1.52 1.51 1.51 1.49 1.49 1.47 1.46 1.45 1.30 1.29 1.28 1.26 1.25 1.23 1.22 1.04 1.01 0.99 0.96 0.94 0.90 0.88 0.85 0.84
HO
H
44; ^{1}H-NMR (600 MHz, CDCl$_3$)
1.00 0.85 2.02 1.01 1.00 2.00 2.01 1.03 6.03 6.03 1.08 3.02 3.01 3.01 3.01 3.00 3.00
δ(¹H) / ppm

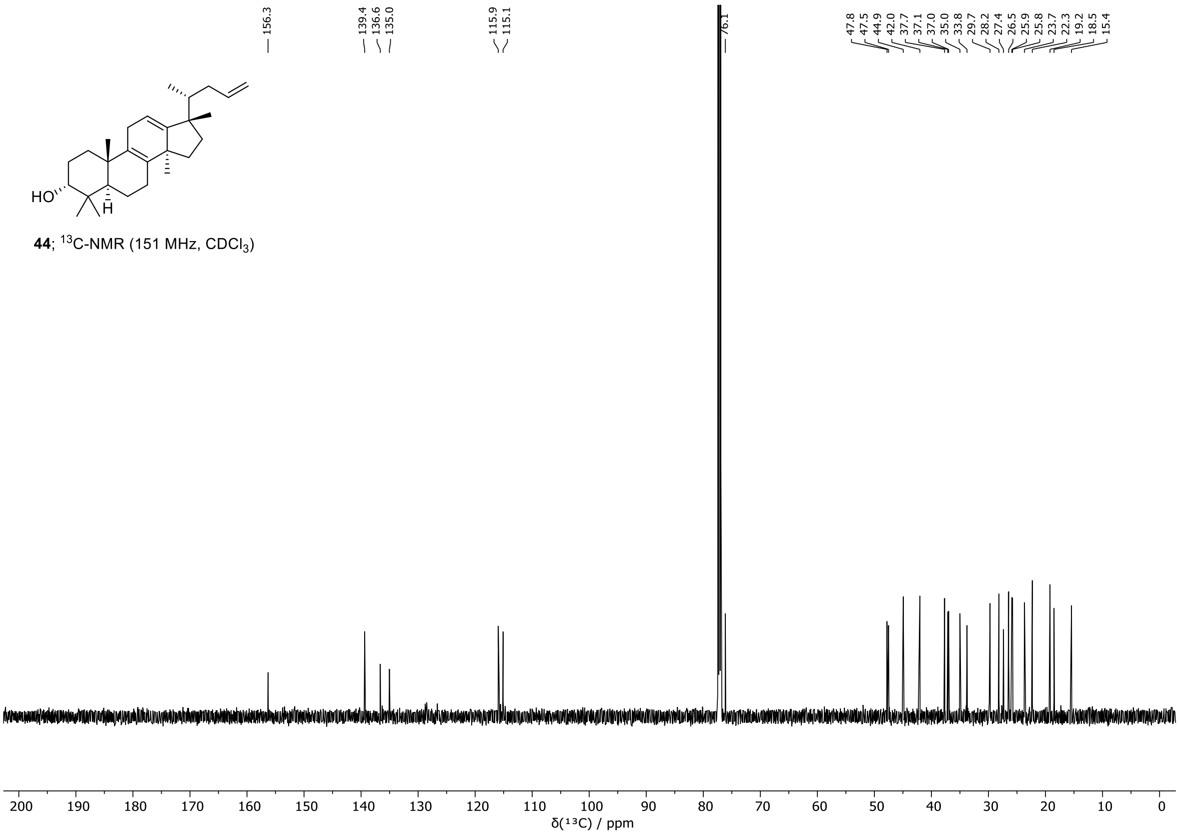
44; ^{13}C-NMR (151 MHz, $CDCl_3$)
HO
H
156.3
139.4
136.6
135.0
115.9
115.1
76.1
47.8
47.5
44.9
42.0
37.7
37.1
37.0
35.0
33.8
29.7
28.2
27.4
26.5
25.9
25.8
23.7
22.3
19.2
18.5
15.4
δ(^{13}C) / ppm

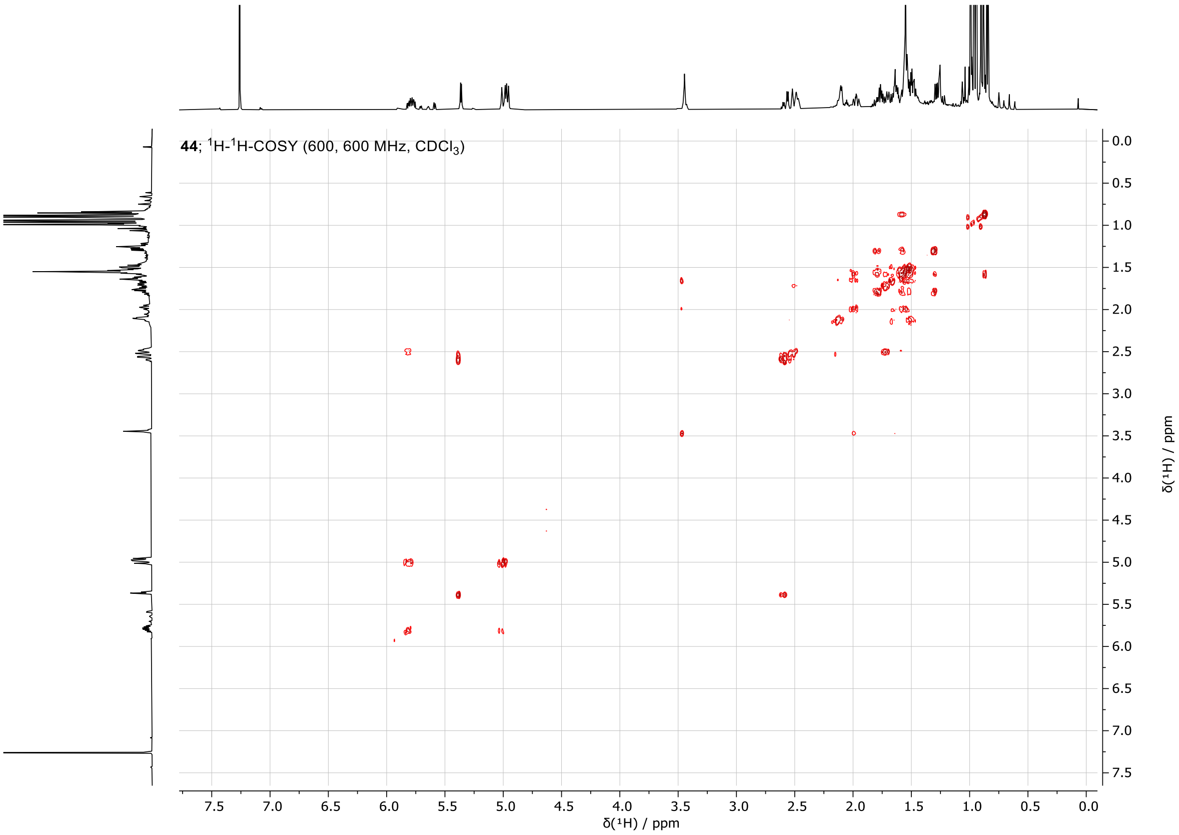
44; ^{1}H-^{1}H-COSY (600, 600 MHz, $CDCl_3$)
δ(^{1}H) / ppm
δ(^{1}H) / ppm

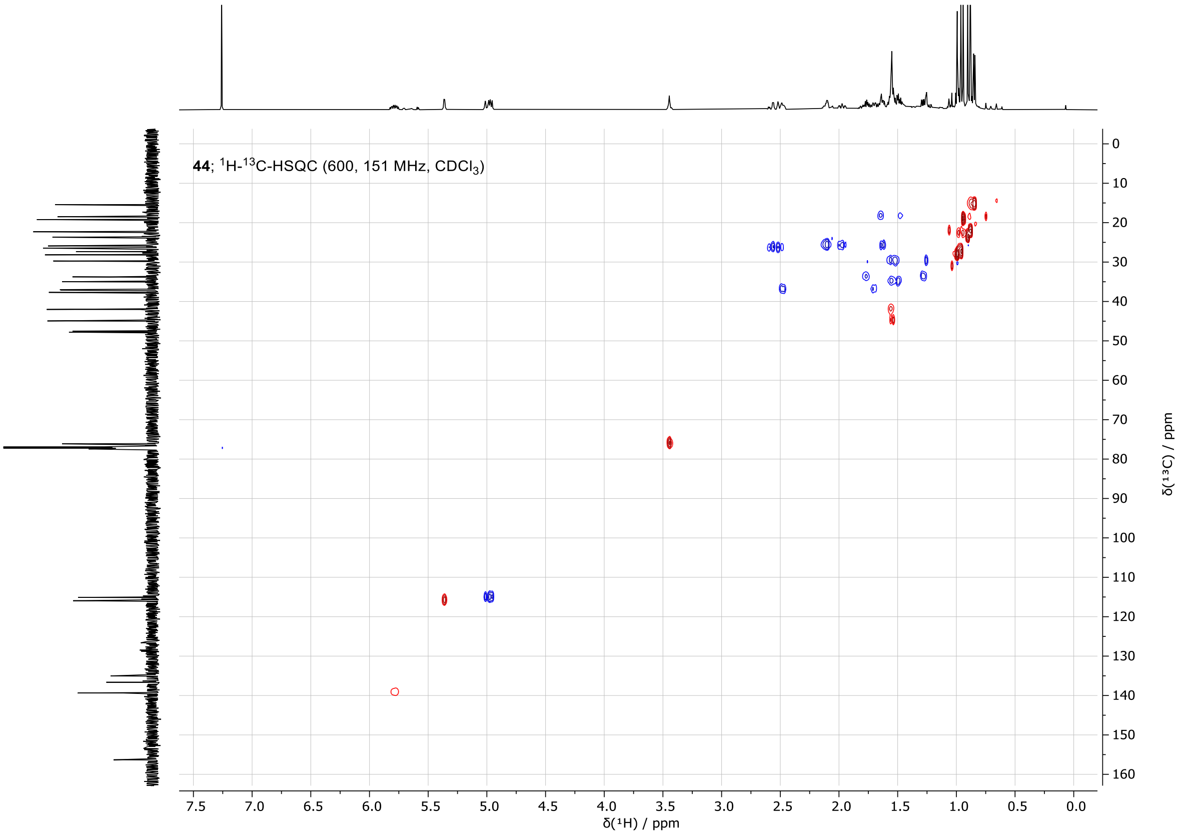

44; ^{1}H-^{13}C-HSQC (600, 151 MHz, $CDCl_3$)
δ(¹H) / ppm
δ(¹³C) / ppm

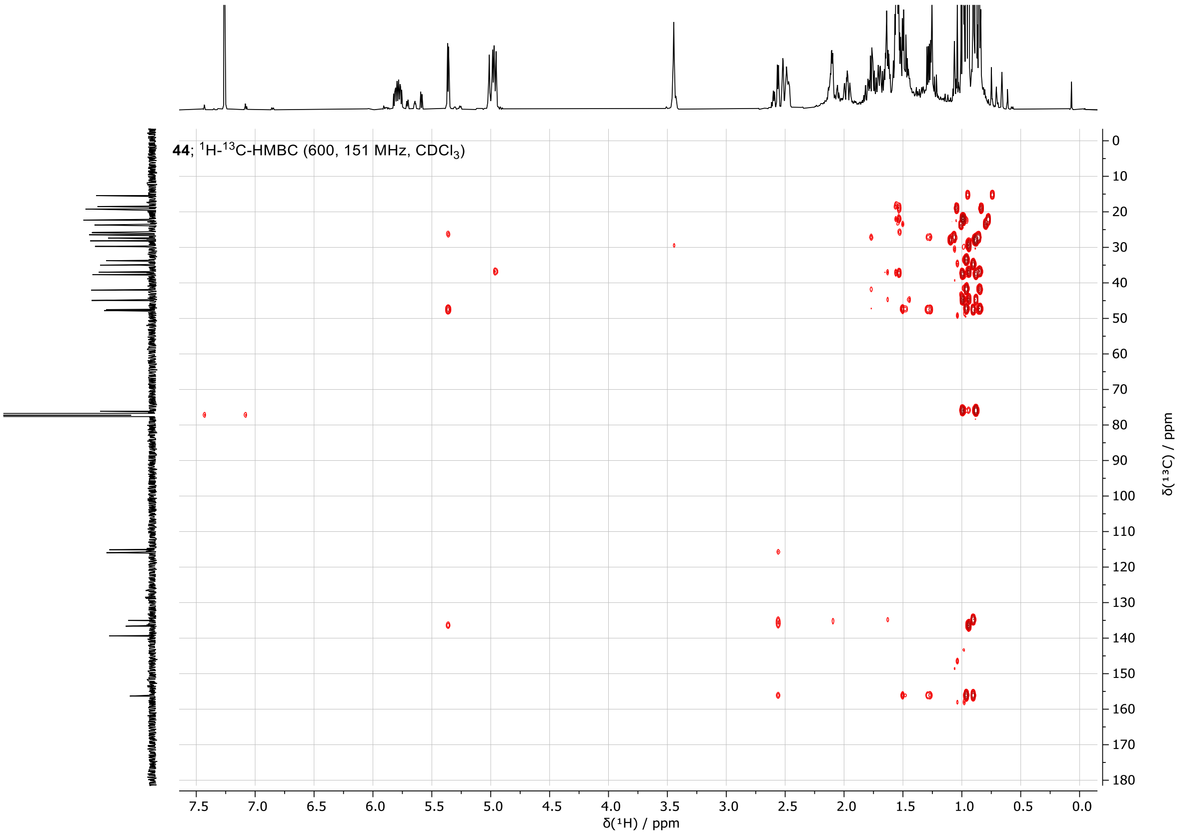

44; ^{1}H-^{13}C-HMBC (600, 151 MHz, $CDCl_3$)
δ(¹H) / ppm
δ(¹³C) / ppm

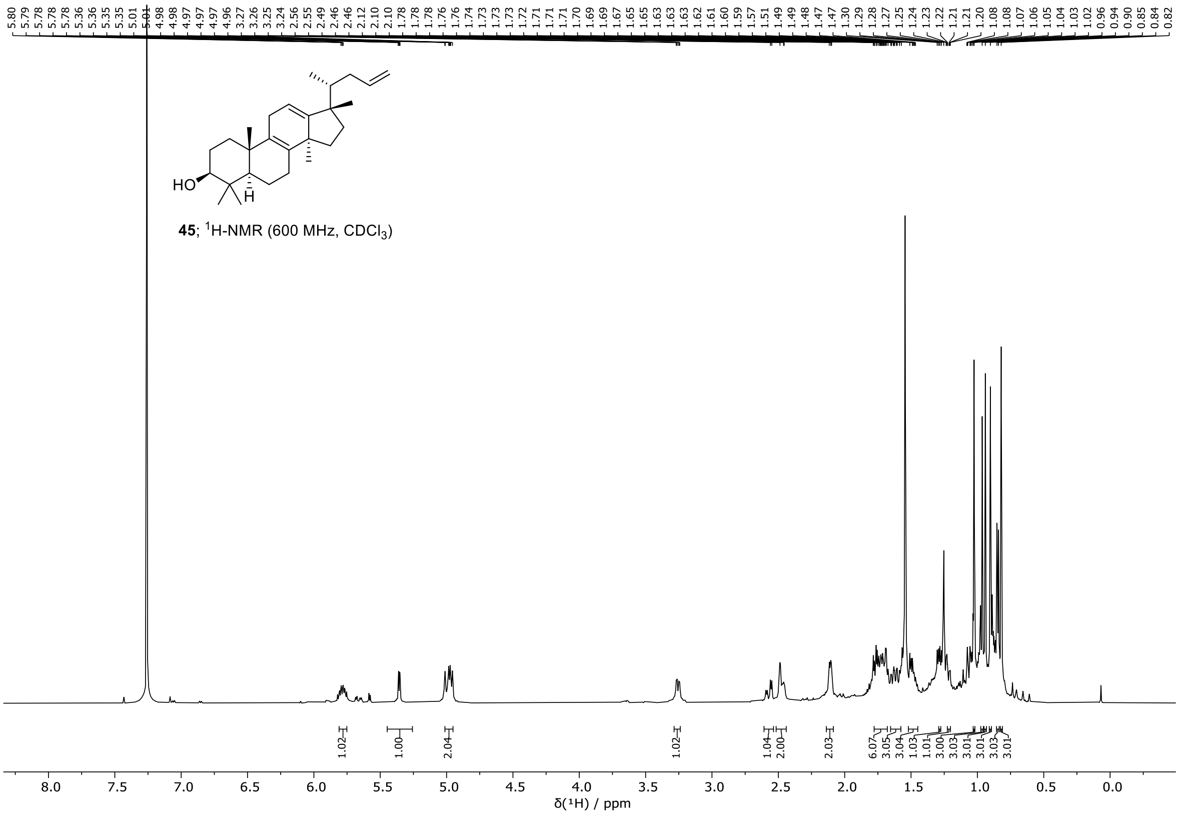
5.80 5.79 5.78 5.78 5.78 5.36 5.36 5.35 5.35 5.01 5.01 4.98 4.98 4.97 4.97 4.96 3.27 3.26 3.25 3.24 2.56 2.55 2.49 2.46 2.46 2.12 2.10 2.10 1.78 1.78 1.78 1.76 1.76 1.74 1.73 1.73 1.73 1.72 1.71 1.71 1.71 1.70 1.69 1.69 1.67 1.65 1.65 1.63 1.63 1.62 1.61 1.60 1.59 1.57 1.51 1.49 1.48 1.47 1.47 1.30 1.29 1.28 1.27 1.25 1.24 1.23 1.22 1.21 1.21 1.20 1.08 1.08 1.07 1.06 1.05 1.04 1.03 1.02 0.96 0.94 0.90 0.85 0.84 0.82
HO
H
45; ¹H-NMR (600 MHz, CDCl₃)
1.02 1.00 2.04 1.02 1.04 2.00 2.03 6.07 3.05 3.04 1.03 1.01 3.00 3.03 3.01 3.01 3.03 3.01
8.0 7.5 7.0 6.5 6.0 5.5 5.0 4.5 4.0 3.5 3.0 2.5 2.0 1.5 1.0 0.5 0.0
δ(¹H) / ppm

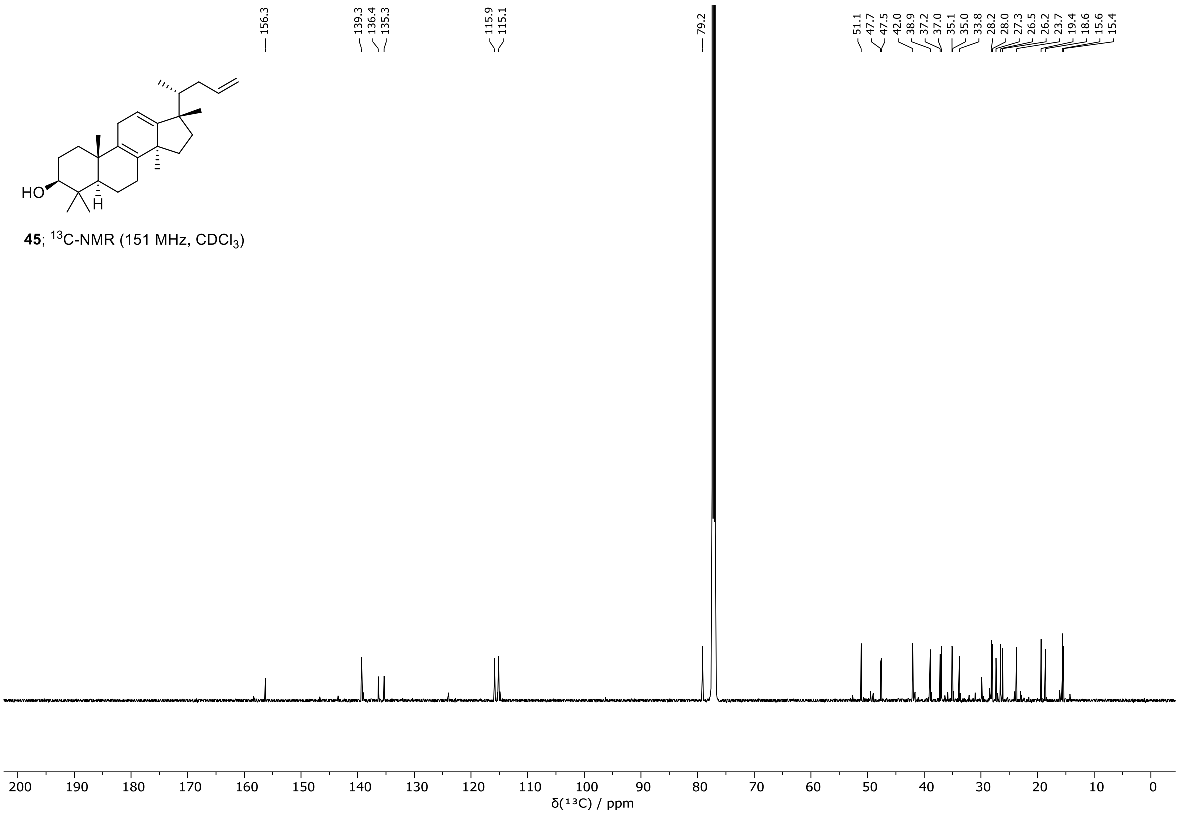
156.3
139.3 136.4 135.3
115.9 115.1
79.2
51.1 47.7 47.5 42.0 38.9 37.2 37.0 35.1 35.0 33.8 28.2 28.0 27.3 26.5 26.2 23.7 19.4 18.6 15.6 15.4
HO
H
45; ¹³C-NMR (151 MHz, CDCl₃)
200 190 180 170 160 150 140 130 120 110 100 90 80 70 60 50 40 30 20 10 0
δ(¹³C) / ppm

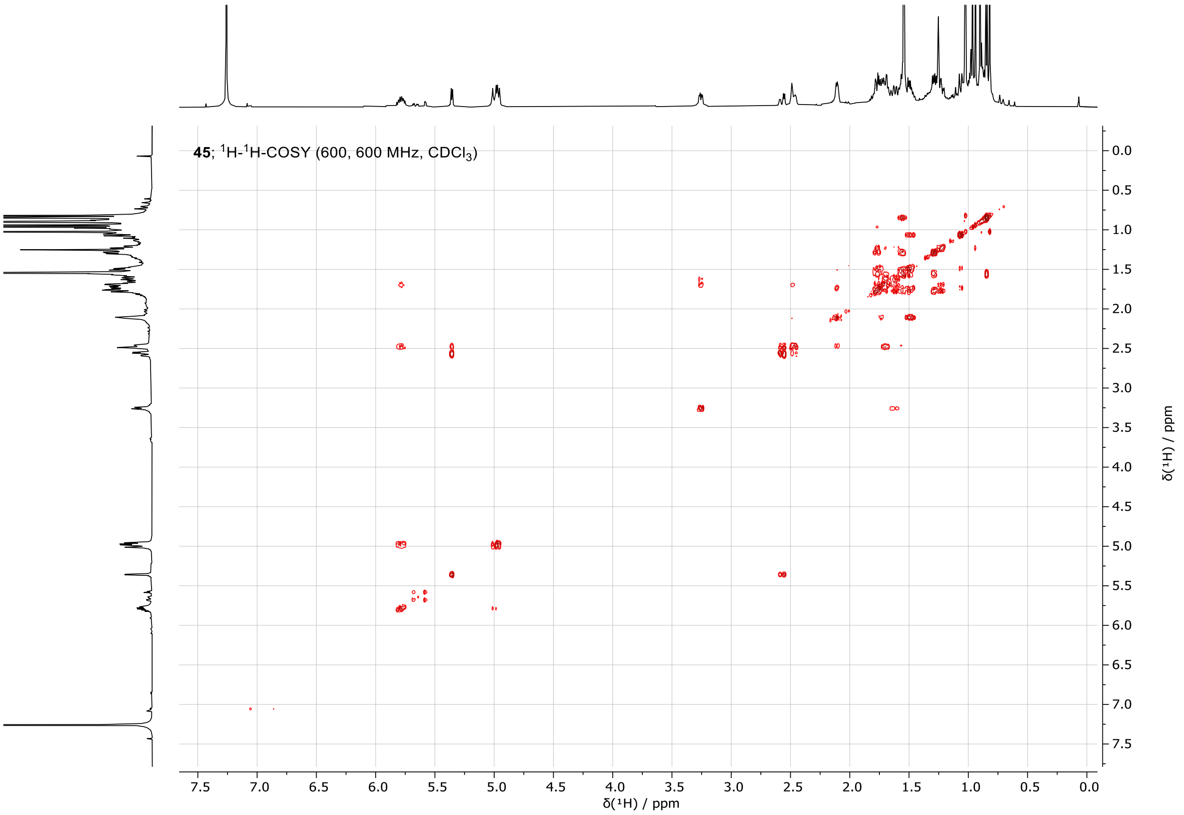

45; ¹H-¹H-COSY (600, 600 MHz, CDCl₃)
δ(¹H) / ppm
δ(¹H) / ppm

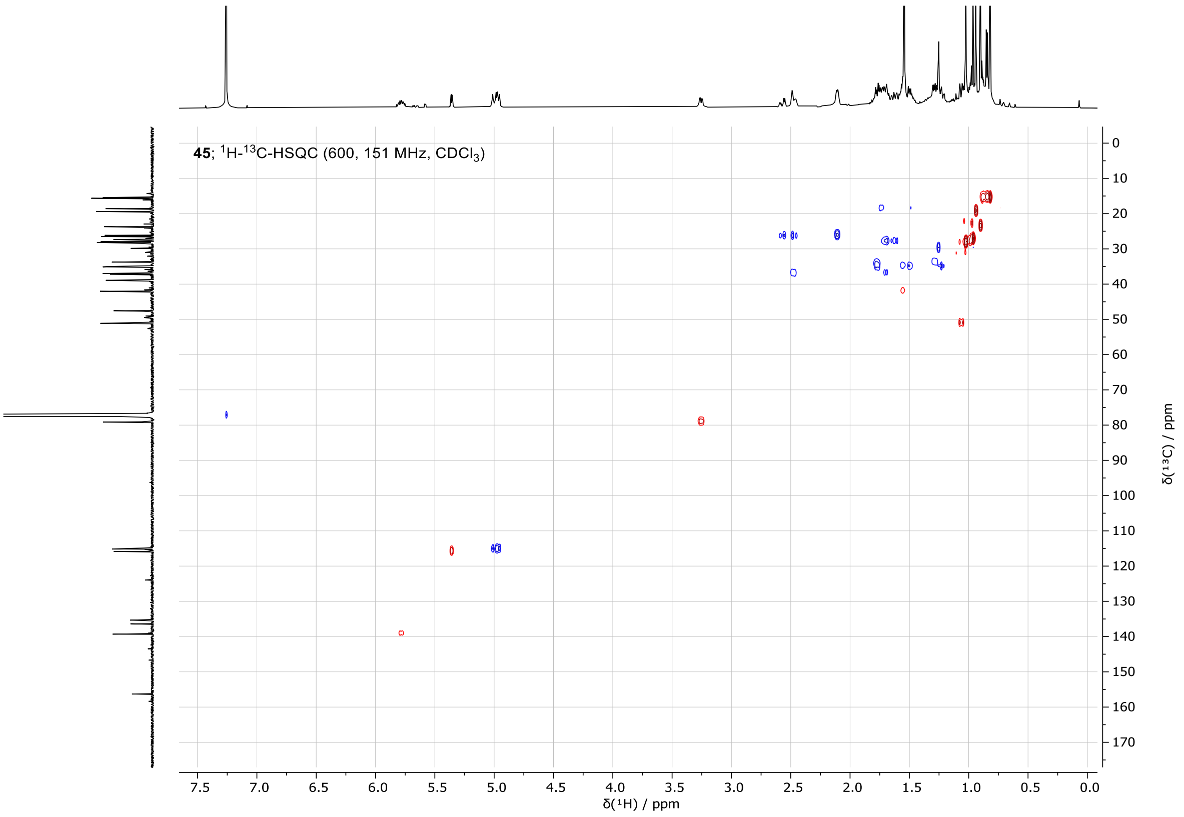

45; ¹H-¹³C-HSQC (600, 151 MHz, CDCl₃)
δ(¹H) / ppm
δ(¹³C) / ppm

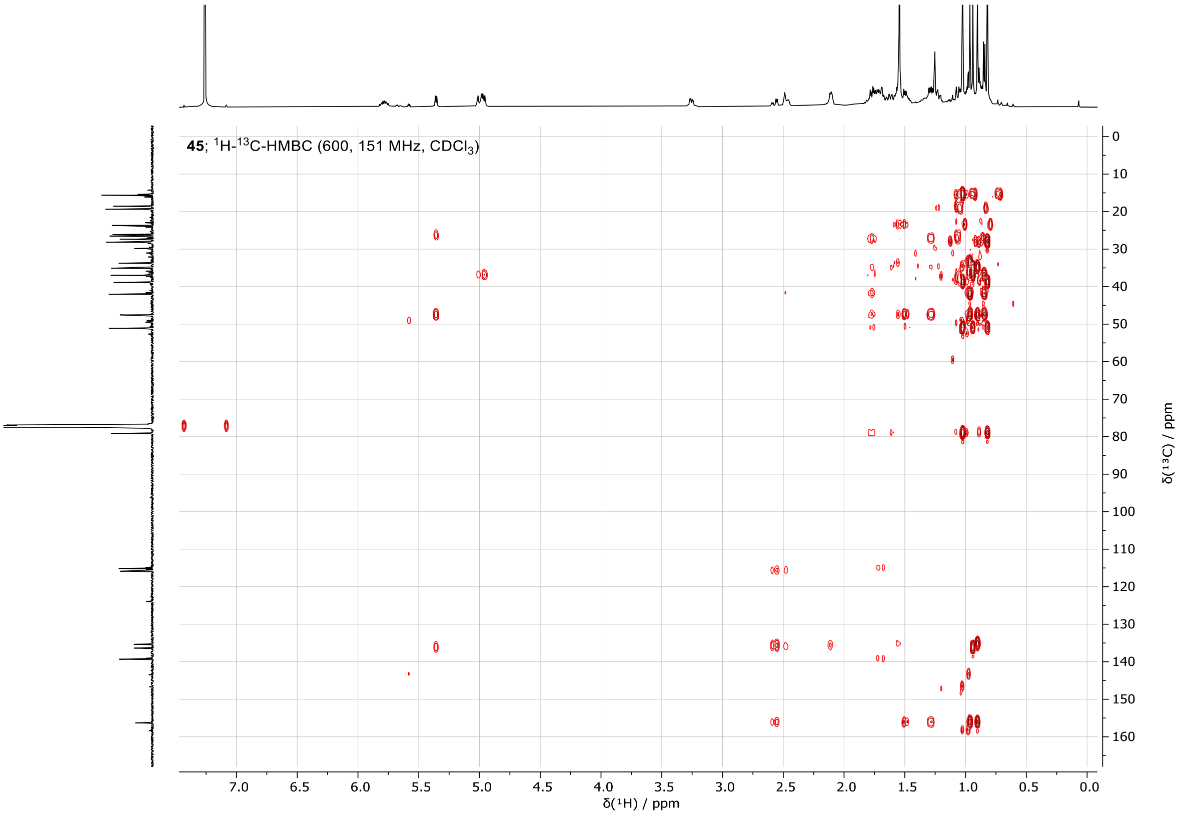
45; ^{1}H-^{13}C-HMBC (600, 151 MHz, CDCl$_3$)
δ(¹H) / ppm
δ(¹³C) / ppm

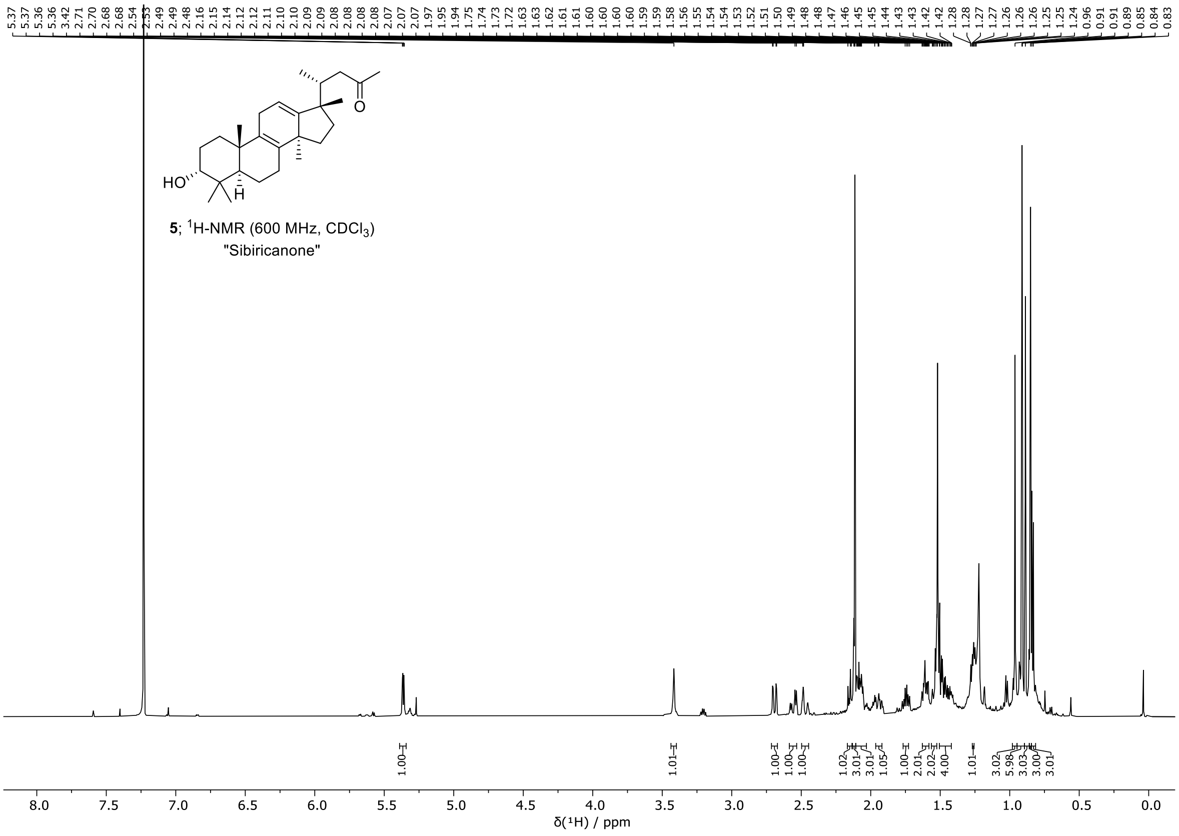
HO
H
O
5; ^{1}H-NMR (600 MHz, CDCl$_3$)
"Sibiricanone"
δ(¹H) / ppm

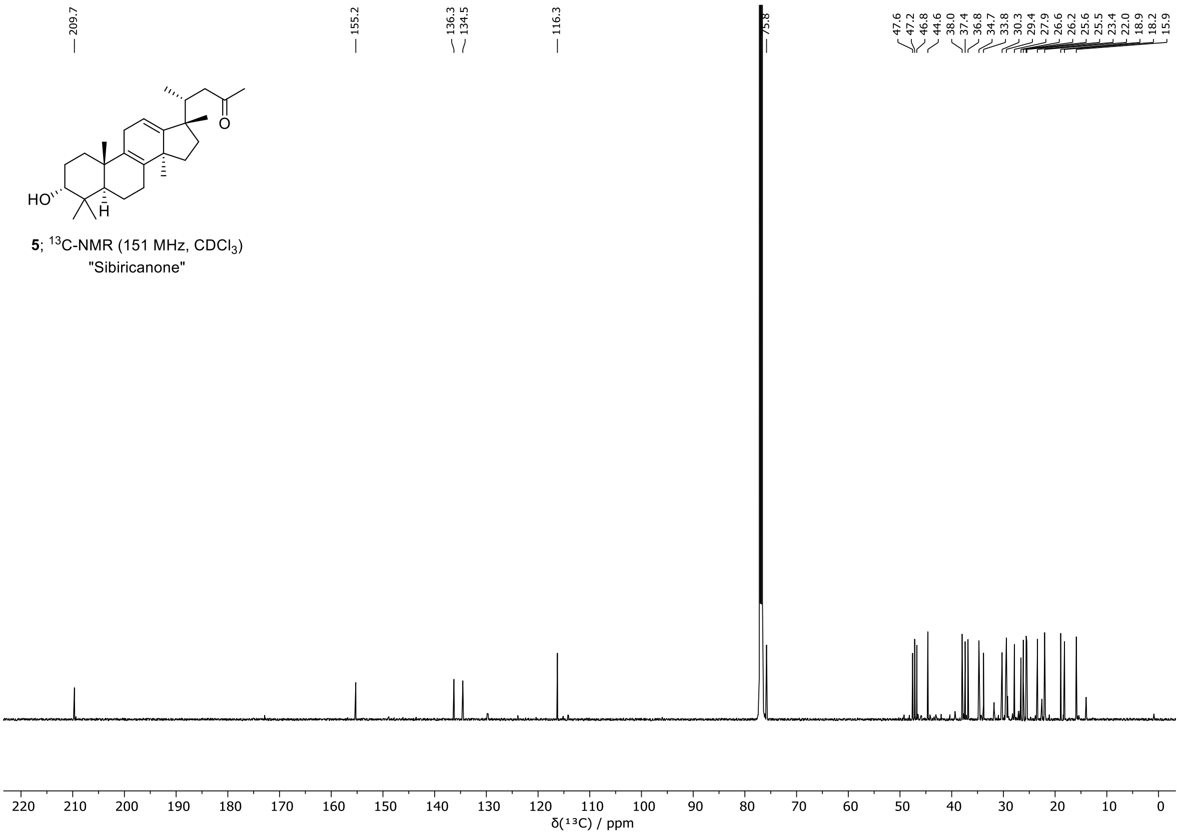
209.7
155.2
136.3
134.5
116.3
75.8
47.6 47.2 46.8 44.6 38.0 37.4 36.8 34.7 33.8 30.3 29.4 27.9 26.6 26.2 25.6 25.5 23.4 22.0 18.9 18.2 15.9
HO
H
O
5; 13C-NMR (151 MHz, CDCl3)
"Sibiricanone"
δ(13C) / ppm

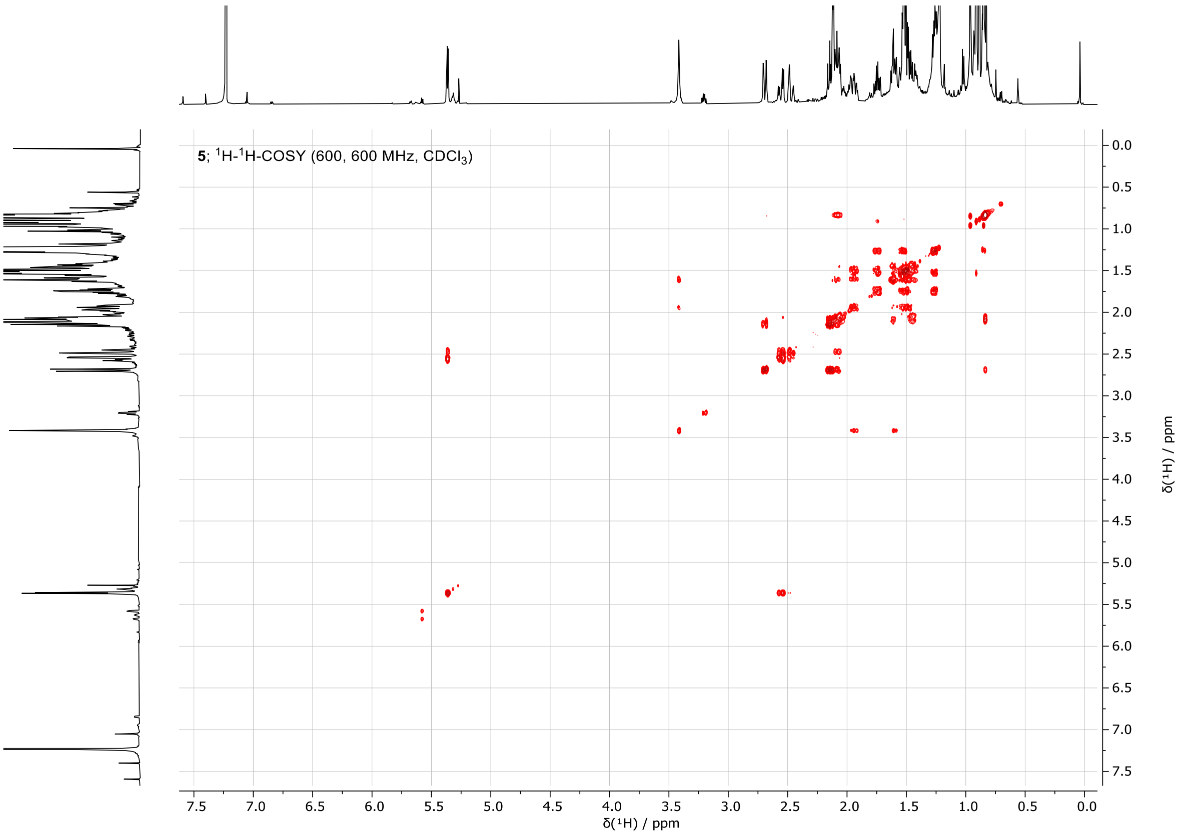
5; 1H-1H-COSY (600, 600 MHz, CDCl3)
δ(1H) / ppm
δ(1H) / ppm

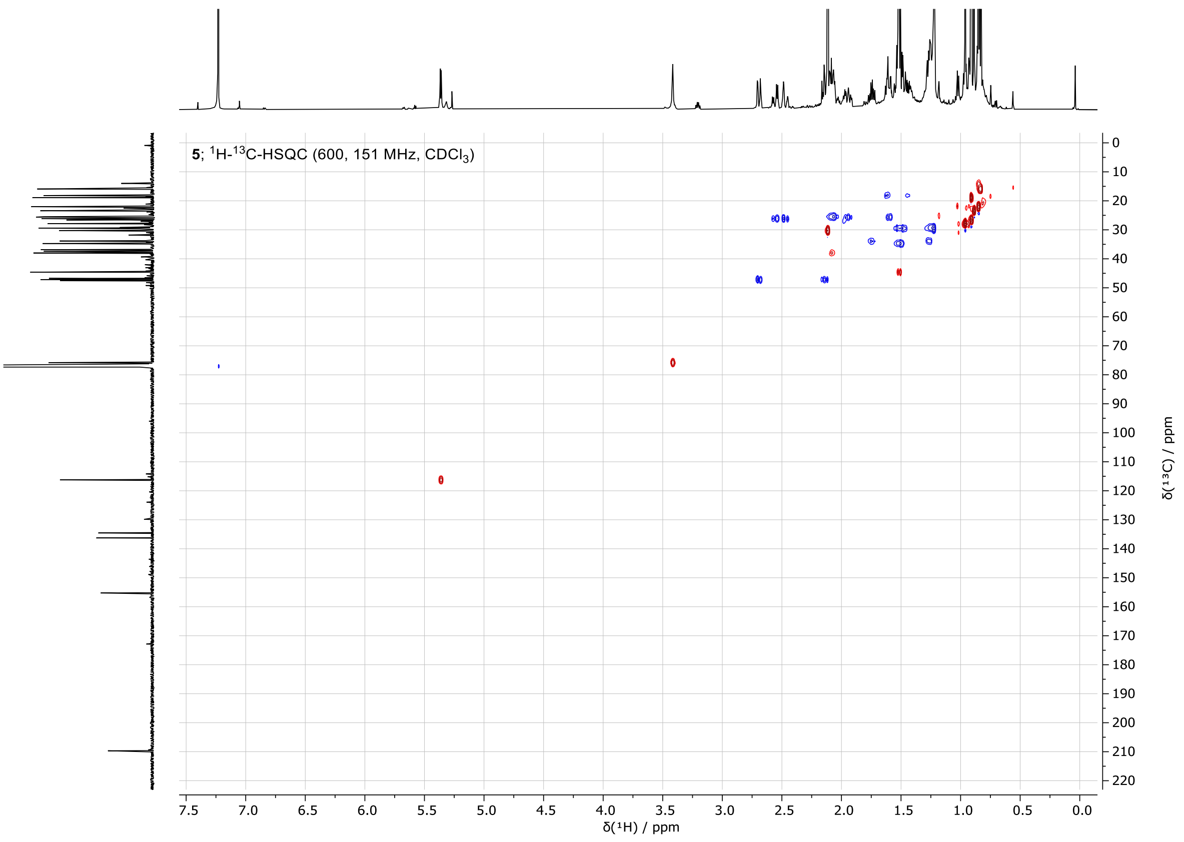
5; ¹H-¹³C-HSQC (600, 151 MHz, CDCl₃)
δ(¹H) / ppm
δ(¹³C) / ppm

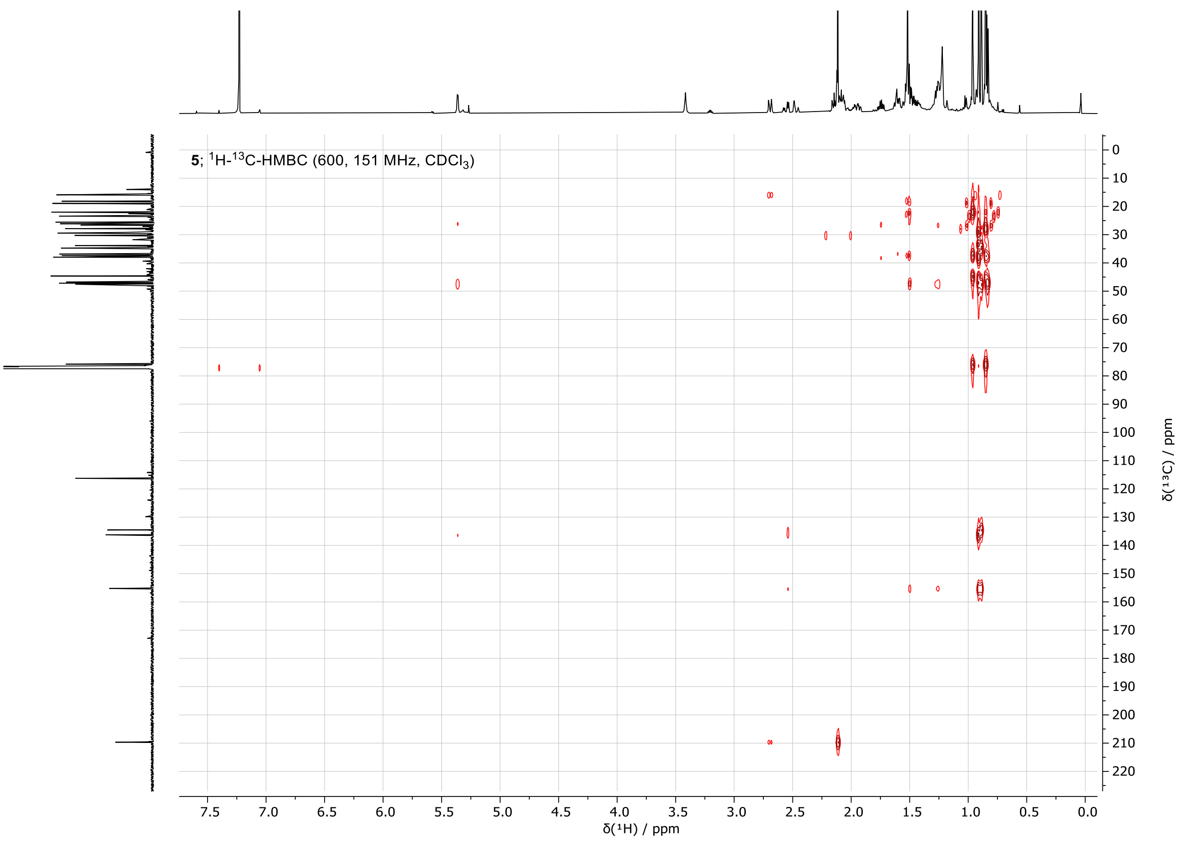
5; ¹H-¹³C-HMBC (600, 151 MHz, CDCl₃)
δ(¹H) / ppm
δ(¹³C) / ppm
